# Correction: Presentations of children to emergency departments across Europe and the COVID-19 pandemic: A multinational observational study

**DOI:** 10.1371/journal.pmed.1004126

**Published:** 2022-11-09

**Authors:** The PLOS Medicine Staff

Fig 3 is incorrect. The authors have provided a corrected version here. The publisher apologises for the error.

**Fig 3:**
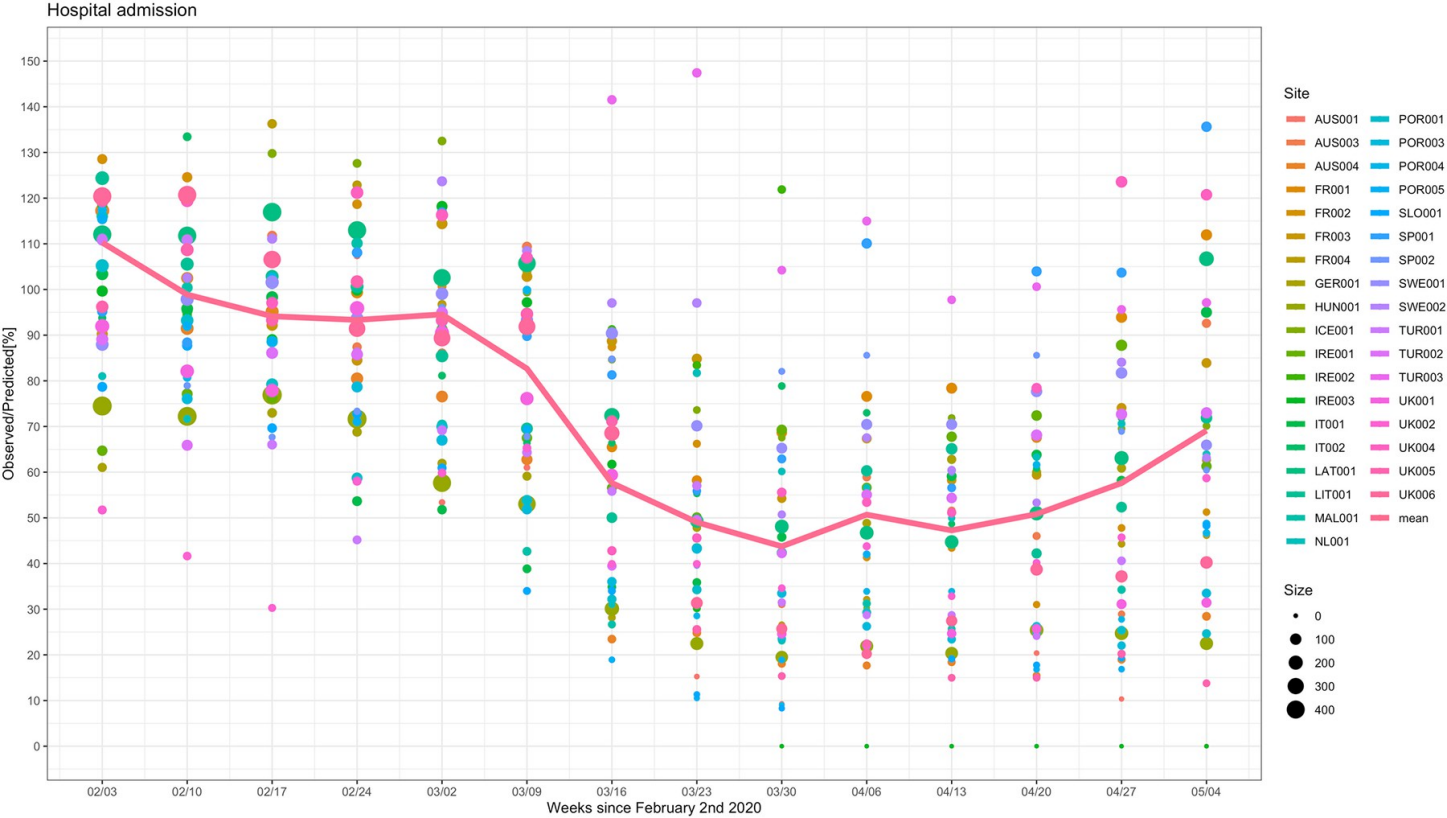
Observed versus predicted hospital admissions for patients attending the ED (%). The observed versus predicted number of children admitted to hospital from the ED in countries across Europe in the weeks following February 2, 2020 until May 11, 2020, for all sites combined. The color and the size of the dots reflect the actual number of ED attendances for each site and for each time window. The line connects the mean of the observed vs. predicted point estimates for each of the individual sites for each time window.
