# Supplementary material for: Presentations of children to emergency departments across Europe and the COVID-19 pandemic: A multinational observational study
Source: PLoS Med. 2022 Aug 26;19(8):e1003974. doi: 10.1371/journal.pmed.1003974 (PMC9467376; doi:10.1371/journal.pmed.1003974)
Supplement: S1 Appendix — (PDF) [file pmed.1003974.s001.pdf]

The EPISODES study group

Paediatric Emergency Outpatient Clinic, Clinical Division of Paediatric Pulmonology, Allergology and Endocrinology, Department of Paediatrics and Adolescent Medicine, Medical University Vienna, Vienna, Austria
Susanne Greber-Platzer[1], Julia Lischka[1]

[1] Clinical Division of Paediatric Pulmonology, Allergology and Endocrinology, Department of Paediatrics and Adolescent Medicine, Comprehensive Centre for Paediatrics, Medical University of Vienna, Vienna, Austria

Department of Pediatrics, Paracelsus Medical University, Salzburg, Austria
Daniel Weghuber, Corinne Vasilico, Jonas Thüminger[1], Matthias Schaffert[1]

[1] Department of Paediatric and Adolescent Surgery, Paracelsus Medical University, Salzburg, Austria

Medical University of Graz, Department of General Paediatrics, Graz, Austria
Daniela Kohlfuerst, Christoph Zurl

Paediatric Emergency Department, Hopital Universitaire Robert-Debre, Paris, France
Luigi Titomanlio[1,2], Alexis Rybak[1,3,4]

[1] Assistance Publique - Hôpitaux de Paris, Pediatric Emergency Department, Robert Debré university hospital, Université de Paris, Paris, France
[2] University of Paris, FHU I2-D2 - INSERM U1141, Paris, France
[2] ACTIV, Association Clinique et Thérapeutique Infantile du Val-de-Marne, Créteil, France
[3] INSERM, ECEVE, UMRS 1123, Université de Paris, Paris, France

Paediatric Emergency Department, Louis Mourier Hospital, Colombes, France
Romain Basmaci, Thibault de Groc

Paediatric Emergency Department, Armand Trousseau Hospital, Paris, France
Romain Guedj

Paediatric Emergency Department, Jean Verdier Hospital, Bondy, France
Camille Aupiais

Paediatric emergency department, Dr. von Hauner Children's Hospital, Ludwig-Maximilians-University Munich, Munich, Germany
Florian Hoffman, Florian Hey

Paediatric Emergency Department, Heim Pal National Paediatric Institute, Budapest, Hungary
Zsolt Bognar, Petra Salamon

Paediatric Emergency Department, Szent Gyorgy University Teaching Hospital of Fejer County, Szekesfehervar, Hungary
Laszlo Fodor, Gábor Simon

Children´s Hospital, Barnaspitali Hringsins, Reykjavik, Iceland
Valtýr Stefánsson Thors, Asgeir Haraldsson, Urdur Jonsdottir

Paediatric Emergency Department, Children's Health Ireland at Crumlin, Ireland
Michael Barrett[1,2,3], Fiona Leonard [1]

[1]Department of Emergency Medicine and Business Intelligence Unit , Children's Health Ireland at Crumlin, Dublin, Ireland
[2]Women's and Children's Health, School Of Medicine, University College Dublin, Dublin, Ireland
[3]National Children's Research Centre, Crumlin, Dublin, Ireland

Paediatric Emergency Department, Children's Health Ireland at Temple Street, Ireland
Paddy Fitzpatrick

Paediatric Emergency Department, Children's Health Ireland at Tallaght, Ireland
Michael Bennett

Division of Paediatric Emergency Medicine, Department of Women's and Children's Health – University Hospital of Padova, Italy
Silvia Bressan, Liviana Da Dalt

Department of Woman and Child Health and Public Health, Fondazione Policlinico Universitario A. Gemelli IRCCS, Rome, Italy
Danilo Buonsenso, Antonio Chiaretti, Rosa Morello

Paediatric emergency department, Children's Clinical University Hospital, Riga Stradins University, Riga, Latvia
Zanda Pučuka

Hospital of Lithuanian University of Health Sciences Kauno Klinikos, Lithuania
Lina Jankauskaite, Vytenis Masilionis

Department of Child and Adolescent Health, Mater Dei Hospital, Msida, Malta
Ruth Farrugia

Department General Paediatrics, ErasmusMC – Sophia, Rotterdam, The Netherlands
Rianne Oostenbrink, Henriette A Moll

Emergency department, Medisch Centrum Alkmaar, Noordwest Ziekenhuisgroep, Alkmaar, The Netherlands
Dorine Borensztajn

Pediatric Emergency Service, Hospital Pediátrico, Centro Hospitalar e Universitário de Coimbra, Portugal
Patrícia Mação[1], Ana Sofia Simões, Nuno Serra de Almeida

[1] University Clinic of Pediatrics, Faculty of Medicine, University of Coimbra, Portugal

Centro Hospitalar e Universitário de São João, Porto, Portugal
Vanessa Gorito, João Viana

Hospital Dona Estefania, Centro Hospitalar de Lisboa Central, Portugal
Susana Castanhinha, Lia Mano

Departamento da Criança e do Jovem- Urgencia Pediatrica, Hospital Prof. Doutor Fernando da Fonseca, Amadora, Portugal
Vanda Anacleto, Ines Mascarenhas, Francisca Saraiva

Paediatric Department, Centro Hospitalar Tondela-Viseu, Viseu, Portugal
Sofia Reis, Sandra Soares Cardoso, Sonia Andrade Santos

University Medical Centre Ljubljana, Univerzitetni Klinični Center, Department of Infectious Diseases, Ljubljana, Slovenia
Mojca Kolnik, Katarina Vincek

Paediatric emergency department, Cruces University Hospital, Barakaldo, Spain
Borja Gomez[1]

[1] Biocruces Bizkaia Health Research Institute, Cruces University Hospital, Barakaldo, Spain

Paediatric emergency unit, Hospital Universitario Río Hortega, Valladolid, Spain
Roberto Velasco

Paediatric emergency department, Astrid Lindgrens Children's hospital, Karolinska University, Sweden
Malin Ryd Rinder

Paediatric emergency department, Sachs' Children and Youth Hospital, Stockholm, Sweden
Tobias Alfven, Samuel Rhedin

Paediatric Emergency Department, Ondokuz Mayıs University, Samsun, Turkey
Esra Akyüz Özkan

Division of Pediatric Emergency Medicine, Department of Pediatrics, Hacettepe University School of Medicine, Ankara, Turkey
Ozlem Teksam, Orkun Aydın

Department of Pediatrics, Division of Emergency Medicine, Mersin City Training and Research Hospital, Toroslar, Mersin, Turkey
Caner Turan, Merve Havan

Paediatric Emergency Medicine Leicester Academic Group, Children's Emergency Department, Leicester Hospitals, Leicester, UK
Damian Roland[1]

[1] SAPPHIRE Group, Health Sciences, Leicester University, Leicester, UK

Department of Paediatric Emergency Medicine, Division of Medicine, St. Mary's hospital - Imperial College NHS Healthcare Trust, London, UK
Ruud G Nijman[12], Ian K Maconochie[1], Tisham De[2], Kate Honeyford[3], Katy Rose[4], Rohan Mongru[2], Danielle Rose[3], Naomi Lin

[1] Centre for Paediatrics and Child Health, Faculty of Medicine, Imperial College London, London, UK
[2] Section of Paediatric Infectious Diseases, Department of Infectious Diseases, Faculty of Medicine, Imperial College London, London, UK
[3] Global Digital Health Unit, School of Public Health, Imperial College London, London, UK
[4] Division of Emergency Medicine - Paediatrics, University College London NHS Foundation Trust, London, UK

Department of paediatric emergency medicine, St. Thomas' hospital, Guy's and St. Thomas' NHS Foundation Trust, London, UK
Sylvester Gomes, Sofia Rapti

Paediatric emergency department, Birmingham women's and children's NHS Foundation Trust, Birmingham, UK
Stuart Hartshorn

Emergency Department, Bristol Royal Hospital for Children, Bristol, UK
Mark D Lyttle[1], Clarissa Barber

[1] Faculty of Health and Applied Sciences, University of the West of England, Bristol, UK

Paediatric emergency department, Alder Hey Children's NHS Foundation Trust, Liverpool, UK
Shrouk Messahel, John Jensen
