# Supplementary material for: Presentations of children to emergency departments across Europe and the COVID-19 pandemic: A multinational observational study
Source: PLoS Med. 2022 Aug 26;19(8):e1003974. doi: 10.1371/journal.pmed.1003974 (PMC9467376; doi:10.1371/journal.pmed.1003974)
Supplement: S1 File — (PDF) [file pmed.1003974.s002.pdf]

EPISODES study

Epidemiology, severity and outcomes of children presenting to emergency departments across Europe during the SARS-CoV-2 pandemic

Protocol version 3, 13-05-2020

MAIN SPONSOR: Imperial College London

FUNDERS: This study is not externally funded. RGN is funded for his research via NIHR ACL award (CL-2018-21-007).

STUDY COORDINATION CENTRE: Imperial College London

IRAS Project ID: 284008
REC reference: n/a

Protocol authorised by:

Name & Role	Date	Signature

STUDY MANAGEMENT GROUP

CHIEF INVESTIGATOR:

Ruud G. Nijman
NIHR Academic clinical lecturer

- Department of Paediatric Emergency Medicine, Division of Medicine, St. Mary's hospital - Imperial College NHS Healthcare Trust, London, London, London, UK
- Faculty of Medicine, Department of Infectious Diseases, Section of Paediatric Infectious Diseases, Imperial College London, UK

CORRESPONDENCE ADDRESS:

Imperial College London
St. Mary's Campus
Medical School
Room 235
Norfolk Place
W2 1PG
Paddington, London

telephone: (0044) 07442360730
e-mail: ruud.nijman@nhs.net
Website: https://www.imperial.ac.uk/people/r.nijman

CO-INVESTIGATORS:

EPISODES study steering group:

Silvia Bressan, silviabress@gmail.com [1],
Danilo Buonsenso, danilobuonsenso@gmail.com [2,3],
Ruth Farrugia, ruth.b.farrugia@gov.mt [4],
Niccolo Parri, niccoparri@gmail.com [5],
Rianne Oostenbrink, r.oostenbrink@erasmusmc.nl [6],
Luigi Titomanlio, luigi.titomanlio@aphp.fr [7],
Damian Roland, dr98@leicester.ac.uk [8,9],
Ian Maconochie, i.maconochie@imperial.ac.uk [10],
Liviana Da Dalt, liviana.dadalt@unipd.it [1],
Zsolt Bognar, bognarzs@heimpalkorhaz.hu [11]
Prof Henriette A Moll, h.a.moll@erasmusmc.nl

[1]Division of Paediatric Emergency Medicine, Department of Women's and Children's Health – University Hospital of Padova, Italy;
[2]Department of Woman and Child Health and Public Health, Fondazione Policlinico Universitario A. Gemelli IRCCS, Rome, Italy

[3]Università Cattolica del Sacro Cuore, Roma, Italia
[4] Department of Child and Adolescent Health, Mater Dei Hospital, Msida, Malta
[5] Emergency Department & Trauma Center, Ospedale Paediatrico Meyer Firenze, Florence, Italy.
[6] Department general Paediatrics, ErasmusMC – Sophia, Rotterdam, The Netherlands
[7] Paediatric Emergency Department, Hopital Universitaire Robert-Debre, Paris, France
[8] SAPPHIRE Group, Health Sciences, Leicester University, Leicester, UK
[9] Paediatric Emergency Medicine Leicester Academic (PEMLA) Group, Leicester Hospitals, Leicester, UK
[10] Department of Paediatric Emergency Medicine, Division of Medicine, St. Mary's hospital - Imperial College NHS Healthcare Trust, London, London, London, UK
[11] Department of Paediatric Emergency Medicine, Heim Pal National Paediatric Institute, Budapest, Hungary

Statistician: Kate Honeyford, k.honeyford@imperial.ac.uk; Global Digital Health Unit, Imperial College London

Study Management: Ruud G Nijman

CLINICAL QUERIES

Clinical queries should be directed to **Ruud G Nijman** who will direct the query to the appropriate person

SPONSOR

Imperial College London is the main research Sponsor for this study. For further information regarding the sponsorship conditions, please contact the Head of Regulatory Compliance at:

Joint Research Compliance Office
Imperial College London and Imperial College Healthcare NHS Trust
Room 215, Level 2, Medical School Building
Norfolk Place
London, W2 1PG
Tel: **0207 594 9459/ 0207 594 1862**
http://www3.imperial.ac.uk/clinicalresearchgovernanceoffice

FUNDER

This study is not externally funded. RGN is funded for his research via NIHR ACL award (CL-2018-21-007).

This protocol describes the epidemiology, severity and outcomes of children presenting to emergency department across Europe during the SARS-CoV-2 pandemic study and provides information about procedures for entering participants. Every care was taken in its drafting, but corrections or amendments may be necessary. These will be circulated to investigators in the study. Problems relating to this study should be referred, in the first instance, to the Chief Investigator.

This study will adhere to the principles outlined in the UK Policy Frame Work for Health and Social Care Research. It will be conducted in compliance with the protocol, the Data Protection Act and other regulatory requirements as appropriate.

Table of Contents

STUDY MANAGEMENT GROUP 2

CHIEF INVESTIGATOR: 2

CORRESPONDENCE ADDRESS: 2

CO-INVESTIGATORS: 2

CLINICAL QUERIES 3

SPONSOR 3

FUNDER 3

GLOSSARY OF ABBREVIATIONS 5

KEYWORDS 5

STUDY SUMMARY 6

1. INTRODUCTION 6

1.1 BACKGROUND AND RATIONALE FOR CURRENT STUDY 7

2. STUDY OBJECTIVES 7

3. STUDY DESIGN 7

3.1 DESIGN 7
3.2 DATA COLLECTION 8
3.3 DATA STORAGE AND DATA MANAGEMENT 8
3.4 STUDY OUTCOME MEASURES 9

4. PARTICIPANT ENTRY 9

4.1 INCLUSION CRITERIA 9
4.2 EXCLUSION CRITERIA 9

5. ASSESSMENT AND FOLLOW-UP 9

6. STATISTICS AND DATA ANALYSIS 10

6.1 STATISTICAL ANALYSIS 10
6.2 PATICIPATING CENTRES AND SAMPLE SIZE 10

7. REGULATORY ISSUES 10

7.1 ETHICS APPROVAL 10
7.2 CONFIDENTIALITY 10
7.3 INDEMNITY 11
7.4 SPONSOR 11
7.5 FUNDING 11
7.6 AUDITS 11
7.7 STUDY REGISTRATION 11

8. STUDY MANAGEMENT 11

9. PUBLICATION POLICY 11

9.1 AUTHORSHIP AGREEMENT 11
9.2 DISSEMINATION 12
10. REFERENCES 13
APPENDIX 1. CLINICAL REPORT FORM, V5, 26-05-2020 .... **ERROR! BOOKMARK NOT DEFINED.**
APPENDIX 2. COUNTRY LEADS, V1, 13-05-2020 .... **ERROR! BOOKMARK NOT DEFINED.**
APPENDIX 3. SURVEY ON STUDY SITE SPECIFIC CHARACTERISTICS, V2, 13-05-2020 . **ERROR! BOOKMARK NOT DEFINED.**
APPENDIX 4. PARTICIPATING SITES AND SITE CODES, V1, 13-05-2020 .... **ERROR! BOOKMARK NOT DEFINED.**
APPENDIX 5. GANT CHART OF EPISODES-STUDY, V1, 13-05-2020 .... **ERROR! BOOKMARK NOT DEFINED.**

GLOSSARY OF ABBREVIATIONS

(P)ED	(P)aediatric emergency department
PIMS-TS	Paediatric Inflammatory Multisystem Syndrome - temporally associated with SARS-CoV-2

KEYWORDS

Paediatric emergency medicine, SARS-CoV-2, children, epidemiology, Europe

STUDY SUMMARY

**TITLE**	The epidemiology, severity and outcomes of children presenting to emergency departments across Europe during the SARS-CoV-2 pandemic (EPISODES study)
**DESIGN**	Retrospective data analysis of aggregated routinely collected and cross-sectional clinical data from (paediatric) emergency departments across Europe
**AIMS**	We aim to describe current patterns of children presenting to (paediatric) emergency departments across Europe and compare these with historical data, to understand the timeliness of their presentations in relation to the disease severity, and to monitor for emerging disease entities.
**OUTCOME MEASURES**	Absolute numbers of children presenting to (paediatric) emergency departments across Europe; for all children and children with different typologies (ie: working diagnosis, age)<br>Severity of illness as defined by: 1) percentage of children with abnormal vital parameters, 2) high triage urgency, 3) a composite outcome of: need for emergency medications, need for hospital admission >24 hours, PICU, and death.<br>Change of relative incidence of children with specific diagnoses of interest and the severity of their presentation as a proxy for timeliness of presentations.
**POPULATION**	All children aged 0 – 16 years presenting to emergency departments across Europe
**ELIGIBILITY**	All children presenting to the emergency department during the specified period, aged 0 – < 18 years, for unscheduled health care [Upper age band being determined by the upper age of children being seen in an institution]<br>That undergo a formal clinical assessment by advanced nurse practitioner (or equivalent) or clinician in the emergency department<br>With all or part of the data of the triaging process (including vital signs), consultation, management (including diagnostics and treatment) and outcomes (including working diagnosis and disposition) routinely documented in the electronic patient record<br>The upper age of children being included will be determined by the upper age bracket of the local institution of children being assessed and seen in the emergency department.
**DURATION**	4 months, 17 days

1. INTRODUCTION

1.1 BACKGROUND AND RATIONALE FOR CURRENT STUDY

Ever since the first cases of SARS-CoV-2 were reported in Europe and since the initial outbreak in Italy in February 2020, the pandemic has caused significant challenges for health care systems and the societies at large across Europe. One of the few reassuring aspects of this pandemic might be that children don't appear to get infected as often as adults, that severe disease in children is rare, and that children appear to play a limited role in the transmission of the virus.(Munro and Faust 2020) As a result, numbers of children attending the paediatric emergency departments have been reported to have fallen drastically.(Isba et al. 2020) However, the reduced numbers appear to be out of keeping with what was to be expected as a result of the government 'lockdown' policies. It is hypothesised that, following the imposed restrictions on free movements by governments, children are not cross-infecting one another with other common childhood diseases with the closure of day care facilities and schools, that they are less exposed to air pollution triggering respiratory disease, and that they are less often involved in high velocity, traffic related trauma. Also, as an unwanted effect of the pandemic, frontline clinicians are anecdotally reporting an increase in delayed presentations of children with serious illness.(Lazzerini et al. 2020) Furthermore, cases of children presenting with an emerging Paediatric Inflammatory Multisystem Syndrome - temporally associated with SARS-CoV-2 (PIMS-TS) have been reported, with some of these children testing positive and some testing negative for SARS-CoV-2.(Jones et al. 2020; RCPCH 2020; Riphagen et al. 2020) At present, no data exist to verify these findings across multiple European countries. Hence, it is important to describe current patterns of children presenting to paediatric emergency department across Europe and compare these with historical data, to understand the timeliness of their presentations in relation to the disease severity, and to monitor for emerging disease entities.

2. STUDY OBJECTIVES

- To describe the change in absolute numbers and the spectrum of type of problems in children presenting to paediatric emergency departments across Europe during the SARS-CoV-2 pandemic in comparison with historical data. [Primary objective]
- To assess the severity and the outcomes of children presenting to paediatric emergency departments across Europe during the SARS-CoV-2 pandemic and the relative change in comparison with historical data. [Secondary objective]

3. STUDY DESIGN

3.1 DESIGN

- Retrospective data analysis of routinely collected clinical data of children presenting to emergency departments across Europe.
- Aggregated, anonymous data will be entered on a monthly basis for each individual participating centre; For the period spanning the Covid pandemic [starting February 2020], this will be aggregated, anonymous data on a weekly basis [Monday through to Sunday].
- Each month or each week will start at the first Monday (00:00 am) of that time period, through to the last Sunday (11:59 pm) of that time period.
- Time period of interest will be January 1st 2018 – May 1st 2020 to allow for collection of historical data for comparison.

3.2 DATA COLLECTION

- A clinical report form has been constructed to capture the anonymised and aggregated data for each participating centre, in a standardized and harmonised manner
- Prior to participation, each centre will indicate which data collection system is being used and which data are routinely available for extraction from their electronic health records
- Minimum requirement of available data for participation include: denominator data of all children presenting, age and gender, date and time of presentation, working diagnosis, hospital admission; and completion of site specific survey.
- For this study, manual data extraction by the participating institutions has been discouraged to ensure timely, standardized and efficient collection of the data.
- The digital data entry system will account for the availability of data of participating centres using conditional linking and centres will only be asked to entre data that is routinely available in their institution
- The clinical report forms will collect data on: (appendix 1)
    - Day and time of presentations
    - Gender, age
    - Referral and mode of arrival
    - Triage urgency
    - Vital signs, including heart rate, respiratory rate, O2 saturation levels, level of consciousness, temperature
    - Diagnostic tests performed in the emergency department; including imaging, blood test, microbiology and virology
    - Treatment; including treatment with bronchodilators, steroids, antibiotics, anti-Covid drugs, other parenteral medications, other nebulised medications, resuscitation drugs.
    - Disposition; including admission to hospital, admission to PICU, death. Time in the emergency department, duration of hospital
- All data are routinely collected clinical variables as part of current best clinical care recommendations
- All data can be extracted automatically from the electronic health records; no data were collected for study purposes only; no manual data extraction was performed.
- Each participating institution will be asked to complete a survey detailing on hospital specific and emergency care specific details, as well as outlining changes to the local healthcare pathways triggered by the Covid outbreak that might have affected the number and type of presentations to the emergency department (appendix 3).

3.3 DATA STORAGE AND DATA MANAGEMENT

- Local data will only be accessed by members of the local clinical team; no one outside the local clinical team will have access to any person identifiable data or any data at a patient individual level.
- The aggregated and anonymised data will be entered using the validated online data entry software Redcap (Harris et al. 2009)
  (website portal: http://redcap.euclids-ci.eu/redcap/index.php?action=myprojects)
- The site specific survey will use the same platform and storing of data. The site will only be identifiable by a specific site code; the PI has a list of the institutions and their site code; all sites will be given a unique site code for all data entry.

- Data will be stored in a data storage cloud alongside data from other data entry projects of the Section of paediatric infectious diseases at Imperial College.
- Data custodian: Dr. Tisham De, tisham.de08@imperial.ac.uk, data manager, room 235, Medical School building, St Mary's campus, Imperial College

3.4 STUDY OUTCOME MEASURES

Primary outcome measure:

- Absolute numbers of children presenting to paediatric emergency department; for all children and children with different typologies (ie: working diagnosis, age)

Secondary outcome measures:

- Severity of illness as defined by: 1) percentage of children with abnormal vital parameters, 2) high triage urgency, 3) a composite outcome of: need for emergency medications, need for hospital admission >24 hours, PICU, and death.
- Change of relative incidence of children with specific diagnoses of interest and the severity of their presentation as a proxy for timeliness of presentations.

4. PARTICIPANT ENTRY

4.1 INCLUSION CRITERIA

- All children presenting to the emergency department during the specified period, aged 0 – < 18 years, for unscheduled health care [Upper age band being determined by the upper age of children being seen in an institution]
- That undergo a formal clinical assessment by advanced nurse practitioner (or equivalent) or clinician in the emergency department
- With all or part of the data of the triaging process (including vital signs), consultation, management (including diagnostics and treatment) and outcomes (including working diagnosis and disposition) routinely documented in the electronic patient record
- The upper age of children being included will be determined by the upper age bracket of the local institution of children being assessed and seen in the emergency department.

4.2 EXCLUSION CRITERIA

- Children visiting the emergency department, who are then streamed to a primary care service for initial consultation.
- Children presenting to the emergency department for scheduled health care or a planned follow-up visit; children who have an unscheduled re-visit to the emergency department within one disease episode are not excluded.

5. ASSESSMENT AND FOLLOW-UP

This study will only collect cross-sectional data and the data will only reflect data from the duration of a patient's stay in the emergency department. There will not be any follow-up or linking of longitudinal data as part of this study.

6. STATISTICS AND DATA ANALYSIS

6.1 STATISTICAL ANALYSIS

- Descriptive analysis, using absolute numbers and percentages, per centre and for all centres combined
- Changes in time will be assessed per centre, for all centres combined, or for clusters of participating centres, and will be described as the change in (pooled) absolute numbers and percentages. Time series analysis will be conducted to show significant changes in outcomes of interests.
- No subgroup analysis for individual countries or institutions will be performed; explorative analysis might identify clusters of participating centres with similar trends in time.

6.2 PATICIPATING CENTRES AND SAMPLE SIZE

We will aim to have 2-4 participating institutions per country, using a quota sampling design, aiming for a total of 50 participating institutions. We have identified country leads for 23 EU countries in a previous collaboration (Appendix 2) [Bressan et al., 2020, Ann Emerg Med]; the country sites will be responsible for approaching additional national sites. Each site will contribute data on approximately 6,000 to 60,000 patients per year for the study period of interest (January 2018 – May 2020). Each site will be assigned a unique site code for data entry purposes (Appendix 4).

Data and all appropriate documentation will be stored for a minimum of 10 years after the completion of the study, including the follow-up period.

7. REGULATORY ISSUES

7.1 ETHICS APPROVAL

The Study Coordination Centre has obtained approval from the Health Regulator Authority (HRA). The study must also receive confirmation of capacity and capability from each participating NHS Trust before accepting participants into the study or any research activity is carried out. The study will be conducted in accordance with the recommendations for physicians involved in research on human subjects adopted by the 18th World Medical Assembly, Helsinki 1964 and later revisions.

A single survey with data on hospital factors and local urgent and emergency care delivery will be completed by every site leads once; this survey will not include any person identifiable data. In any research output, sites will be named by using their site codes only and not their institutional names. Participating hospitals will be encouraged to register their project as a local quality improvement project.

7.2 CONFIDENTIALITY

The Chief Investigator will preserve the confidentiality of participants taking part in the study and is registered under the Data Protection Act.

7.3 INDEMNITY

Imperial College London holds negligent harm and non-negligent harm insurance policies which apply to this study

7.4 SPONSOR

Imperial College London will act as the main Sponsor for this study. Delegated responsibilities will be assigned to the NHS trusts taking part in this study.

7.5 FUNDING

This study did not receive external funding.
RGN was funded by an academic clinical lectureship award from the National Institute of Health research (CL-2018-21-007)

7.6 AUDITS

The study may be subject to inspection and audit by Imperial College London under their remit as sponsor and other regulatory bodies to ensure adherence to GCP and the UK Policy Frame Work for Health and Social Care Research.

7.7 STUDY REGISTRATION

The study and its designs will be registered on the website of the European Society of Emergency Medicine (www.eusem.org) and the network of Research in European Pediatric emergency medicine (REPEM, affiliated with the PEM section of EUSEM: http://repem.net).

8. STUDY MANAGEMENT

The day-to-day management of the study will be co-ordinated through Ruud G Nijman.

9. PUBLICATION POLICY

9.1 Authorship agreement

1. Named authors on any manuscript and other research output originating from the EPISODES study data will be derived from the original study group
2. Any manuscript or other research output derived from the EPISODE study data will be published on behalf of the EPISODES-group, with institutions named in alphabetical order.
3. Additional authors may be considered as long as all the international criteria for authorship contribution have been fulfilled; additional authors should be proposed early to the lead investigators by the site lead.
4. Authors are responsible for providing their correct affiliations and titles, and on checking this for any final version of any manuscript or other research output, such as conference abstracts, that will be submitted
5. The lead investigators are responsible for disseminating any research output to the other members of the EPISODES-group as early as possible for their valued input, checking of correctness and completeness, and their final approval for submission.

6. Authors are responsible for signing their (electronic) conflict of interest forms (ICMJE forms, http://www.icmje.org/conflicts-of-interest/) within 72 hours after having been requested to do so. Failure to do so might lead to removal from the authorship list by the lead author.
7. Authors can nominate people to be acknowledged for their contribution to the EPISODES study; the responsibility of informing these people and gaining their consent to be mentioned in the acknowledgement section lies with the site lead of each institution. This is not equivalent to an authorship, and these people will not be listed in the EPISODES study contributors list.
8. Sequence of the main authors will be alphabetical, except for the first – second – second last – last author that will be decided by the lead investigators of the study.

Ad 1. Original study group:

Silvia Bressan[1], Danilo Buonsenso[2,3], Ruth Farrugia[4], Niccolo' Parri[5], Rianne Oostenbrink[6], Henriette A Moll[6], Luigi Titomanlio[7], Damian Roland[8], Ruud G. Nijman[10,11], Ian Maconochie[10], Liviana Da Dalt[1], Zsolt Bognar [12]

[1]Division of Paediatric Emergency Medicine, Department of Women's and Children's Health – University Hospital of Padova, Italy
[2]Department of Woman and Child Health and Public Health, Fondazione Policlinico Universitario A. Gemelli IRCCS, Rome, Italy
[3]Università Cattolica del Sacro Cuore, Roma, Italia
[4] Department of Child and Adolescent Health, Mater Dei Hospital, Msida, Malta
[5] Emergency Department & Trauma Center, Ospedale Paediatrico Meyer Firenze, Florence, Italy.
[6] Department of General Paediatrics, ErasmusMC – Sophia, Rotterdam, The Netherlands
[7] Paediatric Emergency Department, Hopital Universitaire Robert-Debre, Paris, France
[8] SAPPHIRE Group, Health Sciences, Leicester University, Leicester, UK
[9] Paediatric Emergency Medicine Leicester Academic (PEMLA) Group, Leicester Hospitals, Leicester, UK
[10] Department of Paediatric Emergency Medicine, Division of Medicine, St. Mary's hospital - Imperial College NHS Healthcare Trust, London, London, London, UK
[11]Faculty of Medicine, Department of Infectious Diseases, Section of Paediatric Infectious Diseases, Imperial College London, UK
[12] Department of Paediatric Emergency Medicine, Heim Pal National Paediatric Institute, Budapest, Hungary

Ad 8.

1st author: RG Nijman; 2nd author S Bressan; second-last R Oostenbrink; last L Titomanlio

9.2 Dissemination

Our data will show the impact of the SARS-CoV-2 pandemic on the numbers of children presenting to paediatric emergency department across Europe. We will also be able to describe any changes in severity and the different typologies of children needing emergency care, comparing the data from during the outbreak with historical data. This will provide strong data on the issues of the timeliness children presenting to EDs and the potential increase of incidence of children with Kawasaki -like and hyperinflammatory conditions across Europe. We have carefully balanced the

need for large scale epidemiologic data against the need for in depth patient individual data. The steering group has extensive experience in this type of research and the use of the European network of paediatric emergency care physicians.(Mintegi et al. 2008; Van de Voorde et al. 2013; van de Maat et al. 2019) This type of research fits in well with the outlined research priorities of the Research in Paediatric Emergency Medicine (REPEM) Network.(Bressan et al. 2019) Our study design will also allow for future studies looking into more detail in specific typologies.
We expect to publish our main manuscript in a leading international peer reviewed journal, and to present this work at international conferences. We also expect to produce short papers on 1) difficulties on harmonising routinely collected clinical data from European paediatric emergency departments, 2) changes in health care pathways across Europe amongst participants of the EPISODES-study. Secondary analysis of the data may be performed with approval of the EPISODES steering group after review of a study proposal by any member of the EPISODES study group.
Furthermore, the EPISODES study will position us in a unique position to respond rapidly to a potential second wave of SARS-CoV-2 infections and to collect data on epidemiological issues in paediatric emergency medicine.

10. REFERENCES

Bressan, Silvia, Luigi Titomanlio, Borja Gomez, Santiago Mintegi, Alain Gervaix, Niccolo Parri, Liviana Da Dalt, et al. 2019. "Research Priorities for European Paediatric Emergency Medicine." *Archives of Disease in Childhood*, April, archdischild-2019-316918. https://doi.org/10.1136/archdischild-2019-316918.

Harris, Paul A, Robert Taylor, Robert Thielke, Jonathon Payne, Nathaniel Gonzalez, and Jose G Conde. 2009. "Research Electronic Data Capture (REDCap)—A Metadata-Driven Methodology and Workflow Process for Providing Translational Research Informatics Support." *Journal of Biomedical Informatics* 42 (2): 377–81. https://doi.org/https://doi.org/10.1016/j.jbi.2008.08.010.

Isba, Rachel, Rhiannon Edge, Rachel Jenner, Emily Broughton, Natalie Francis, and Jim Butler. 2020. "Where Have All the Children Gone? Decreases in Paediatric Emergency Department Attendances at the Start of the COVID-19 Pandemic of 2020." *Archives of Disease in Childhood*, May, archdischild-2020-319385. https://doi.org/10.1136/archdischild-2020-319385.

Jones, Veena G, Marcos Mills, Dominique Suarez, Catherine A Hogan, Debra Yeh, J Bradley Segal, Elizabeth L Nguyen, Gabrielle R Barsh, Shiraz Maskatia, and Roshni Mathew. 2020. "COVID-19 and Kawasaki Disease: Novel Virus and Novel Case." *Hospital Pediatrics*, May, hpeds.2020-0123. https://doi.org/10.1542/hpeds.2020-0123.

Lazzerini, Marzia, Egidio Barbi, Andrea Apicella, Federico Marchetti, Fabio Cardinale, and Gianluca Trobia. 2020. "Delayed Access or Provision of Care in Italy Resulting from Fear of COVID-19." *The Lancet Child & Adolescent Health* 4 (5): e10–11. https://doi.org/10.1016/S2352-4642(20)30108-5.

Maat, Josephine van de, Elles van de Voort, Santiago Mintegi, Alain Gervaix, Daan Nieboer, Henriette Moll, and Rianne Oostenbrink. 2019. "Antibiotic Prescription for Febrile Children in European Emergency Departments: A Cross-Sectional, Observational Study." *The Lancet. Infectious Diseases*, February. https://doi.org/10.1016/S1473-3099(18)30672-8.

Mintegi, Santiago, Mark D Lyttle, Ian K Maconochie, Javier Benito, Alain Gervaix, Henriette Moll, Itai Shavit, et al. 2008. "Pediatric Emergency Care in Europe: A Descriptive Survey of 53 Tertiary Medical Centers." *European Journal of Pediatrics* 172 (5): 667–74. https://doi.org/10.1007/s00431-013-1930-x.

Munro, Alasdair P S, and Saul N Faust. 2020. "Children Are Not COVID-19 Super Spreaders: Time to Go Back to School." *Archives of Disease in Childhood*, May, archdischild-2020-319474. https://doi.org/10.1136/archdischild-2020-319474.

RCPCH. 2020. “Royal College of Paediatrics and Child Health - Guidance - Paediatric Multisystem Inflammatory Syndrome Temporally Associated with COVID-19.” 2020. https://www.rcpch.ac.uk/resources/guidance-paediatric-multisystem-inflammatory-syndrome-temporally-associated-covid-19.

Riphagen, Shelley, Xabier Gomez, Carmen Gonzalez-Martinez, Nick Wilkinson, and Paraskevi Theocharis. 2020. “Hyperinflammatory Shock in Children during COVID-19 Pandemic.” *The Lancet*, May. https://doi.org/10.1016/S0140-6736(20)31094-1.

Voorde, P Van de, B Emerson, B Gomez, J Willems, D Yildizdas, I Iglowstein, E Kerkhof, et al. 2013. “Paediatric Community-Acquired Septic Shock: Results from the REPEM Network Study.” *European Journal of Pediatrics* 172 (5): 667–74. https://doi.org/10.1007/s00431-013-1930-x.
