# Supplementary material for: Presentations of children to emergency departments across Europe and the COVID-19 pandemic: A multinational observational study
Source: PLoS Med. 2022 Aug 26;19(8):e1003974. doi: 10.1371/journal.pmed.1003974 (PMC9467376; doi:10.1371/journal.pmed.1003974)
Supplement: S2 File — (PDF) [file pmed.1003974.s003.pdf]

ISRCTN registry
ISRCTN91495258 https://doi.org/10.1186/ISRCTN91495258
[https://doi.org/10.1186/ISRCTN91495258]

Investigating the impact of the coronavirus (COVID-19) pandemic on children presenting to emergency departments across Europe

Submission date
06/07/2020
Registration date
14/07/2020
Last edited
01/04/2021

Recruitment status
No longer recruiting
Overall trial status
Completed
Condition category
Not Applicable

✓ Retrospectively registered
? Protocol not yet available
? SAP not yet available
? Results not yet available and trial completed for less than 1 year
? Raw data not yet available
✓ Study completed

Plain English Summary

Background and study aims
COVID-19 is a condition caused by the coronavirus (called SARS-CoV-2) that was first identified in late 2019. This virus can infect the respiratory (breathing) system. Ever since the first cases of SARS-CoV-2 were reported in Europe, and since the initial outbreak in Italy in February 2020, the pandemic has caused significant challenges for health care systems and the societies at large across Europe.

One of the few reassuring aspects of this pandemic might be that children don't appear to get infected as often as adults, that severe disease in children is rare, and that children appear to play a limited role in the transmission of the virus. As a result, numbers of children attending hospital emergency departments have been reported to have fallen drastically. However, the reduced numbers appear to be out of keeping with what was to be expected as a result of the government 'lockdown' policies. It is thought that, as a result of the imposed restrictions on free movements by governments, children are not cross-infecting one another with other common childhood diseases with the closure of daycare facilities and schools, that they are less exposed to air pollution triggering the respiratory disease, and that they are less often involved in high velocity, traffic-related trauma.

Also, as an unwanted effect of the pandemic, frontline clinicians are noticing an increase in delayed presentations of children with serious illness. Furthermore, cases of children presenting with an

emerging Paediatric Inflammatory Multisystem Syndrome - temporally associated with Sars-Cov-2 (PIMS-TS) have been reported, with some of these children testing positive and some testing negative for SARS-CoV-2. At present, no there is no evidence to confirm these findings across multiple European countries. Therefore, it is important to describe current patterns of children presenting to paediatric emergency departments across Europe and compare these with historical data. The aim of this is to provide evidence for changes to attendance to emergency departments for children; to monitor for possible new diseases; and to understand the timeliness of their presentations in relation to the disease severity, to confirm if children are attending emergency departments later than normal during the pandemic and therefore have more severe symptoms by the time they are first seen by healthcare staff.

This study will be performed by the EPISODES study steering group, in collaboration with the European Society of Emergency Medicine and the Research in European Paediatric Emergency Medicine network.

Who can participate?
The collective data of all children presenting to the emergency departments of the participating centres during the period between January 1st, 2018 and May 1st, 2020 will be included in this trial.

What does the study involve?
This study will involve analysis of routinely collected clinical data of all children presenting to emergency departments across Europe over a 2 and half year period. The data will not be identifiable and will be collected on a monthly basis for each individual participating centre during the period spanning the COVID-19 pandemic (beginning February 2020). The historical data (from January 2018 and prior to February 2020) will be collected to serve as a comparison.

What are the possible benefits and risks of participating?
As this study does not involve any change to the care of the children whose data is included and that no individual patient data or identifiable data will be collected, there are not thought to be any risks involved in this study. It is hoped that data will show the impact of the COVID-19 pandemic on the numbers of children presenting to emergency departments across Europe and may, therefore, be used to provide advice on emergency department attendance for children, and to respond rapidly to a potential second wave of the pandemic.

Where is the study run from?
Imperial College London (UK). There are currently 40 confirmed participating sites (1-4 centres per European country) providing data for the study.

When is the study starting and how long is it expected to run for?
From June to December 2021

Who is funding the study?
The study is investigator-initiated and funded.

Who is the main contact?
Dr Ruud Nijman
r.nijman@imperial.ac.uk

Trial website

https://www.eusem.org/news/564-help-research-in-paediatric-emergency-medicine-moving-forward [https://www.eusem.org/news/564-help-research-in-paediatric-emergency-medicine-moving-forward]

Contact information

Type

Scientific

Primary contact

Dr Ruud Nijman

ORCID ID

https://orcid.org/0000-0001-9671-8161 [https://orcid.org/0000-0001-9671-8161]

Contact details

St Marys Campus
Medical School Building
Room 235
Norfolk Place
London
W2 1PG
United Kingdom
+44 (0)7442360730
r.nijman@imperial.ac.uk [mailto:r.nijman@imperial.ac.uk]

Additional identifiers

EudraCT number

Nil known

IRAS number

284008

ClinicalTrials.gov number

Nil known

Protocol/serial number

IRAS 284008

Study information

Scientific title

The epidemiology, severity, and outcomes of children presenting to emergency departments across Europe during the SARS-COV-2 pandemic: the EPISODES study

Acronym

EPISODES

Study hypothesis

This study aims to describe current patterns of children presenting to paediatric emergency departments across Europe during the SARS-CoV-2 pandemic and compare these with historical data, to understand the timeliness of their presentations in relation to the disease severity, and to monitor for emerging disease entities.

Ethics approval

Approved 18/06/2020, UK HRA, Imperial College Research Governance and Integrity Team (Joint Research Compliance Office Office Room 221, Medical School Building, St Mary's Campus, Imperial College London W2 1NY; n.shaikh@imperial.ac.uk; +44 (0)20 7594 9484), ref: 20SM6003

Study design

Retrospective analysis of routinely collected clinical data

Primary study design

Observational

Secondary study design

Epidemiological study

Trial setting

Hospitals

Trial type

Diagnostic

Patient information sheet

No participant information sheet available

Condition

Paediatric emergency department presentation

Intervention

Current interventions as of 01/04/2021:
This study will involve retrospective analysis of routinely collected clinical data of all children presenting to emergency departments across Europe over a 2 and half year period. Aggregated, anonymous data will be entered on a monthly basis for each individual participating centre during the

period spanning the COVID-19 pandemic (beginning February 2020). All data will be extracted from electronic health care records by the local clinical teams. Monthly aggregated data will be entered on a validated and secure online platform (RedCap). Aggregated, anonymous data will be presented on a weekly/monthly basis where each week period will start on the first Monday (00:00 am) of that time period, through to the last Sunday (11:59 pm) of that time period. The total time period of interest will be January 1st, 2018 to May 17th, 2020 to allow for the collection of historical data (prior to February 2020) for comparison. Once the data is collected it will be analysed after the end of the period of interest.

A quota sampling design will be used to select from which 1-4 institutions from each participating European countries data will be collected. Every site lead will complete a site-specific survey to inform on hospital-specific factors and local changes to healthcare pathways induced by the SARS-CoV-2 pandemic. No data with personally identifiable data will be collected, nor any data on a patient individual level. Data will be analysed by comparing absolute numbers and percentages of children presenting to emergency departments, the severity of their presenting problems, their working diagnoses, and the patient outcomes, over time during the study period.

We will use historic datasets to calibrate time series auto-regressive integrated moving average (ARIMA) forecasting models, in order to predict the expected number of ED attendances for different conditions using national-level and local data pre- and during the COVID-19 pandemic. We will compare the forecasted trends to the observed data for the same periods of time. Site-specific surveys detailing local health care pathways, and COVID-19 related changes to these pathways, will allow for unique local mediation analysis, and the ARIMA models will be adjusted for local policy interventions on social distancing and other lockdown measures.

An extension of the study period will allow data until May 2021 to be collected.

Previous interventions:
This study will involve retrospective analysis of routinely collected clinical data of all children presenting to emergency departments across Europe over a 2 and half year period. Aggregated, anonymous data will be entered on a monthly basis for each individual participating centre during the period spanning the COVID-19 pandemic (beginning February 2020). All data will be extracted from electronic health care records by the local clinical teams. Monthly aggregated data will be entered on a validated and secure online platform (RedCap). Aggregated, anonymous data will be presented on a weekly basis where each month or each week period will start at the first Monday (00:00 am) of that time period, through to the last Sunday (11:59 pm) of that time period. The total time period of interest will be January 1st, 2018 to May 1st, 2020 to allow for the collection of historical data (prior to February 2020) for comparison. Once the data is collected it will be analysed after the end of the period of interest.

A quota sampling design will be used to select from which 1-4 institutions from each participating European countries data will be collected. Every site lead will complete a site-specific survey to inform on hospital-specific factors and local changes to healthcare pathways induced by the SARS-CoV-2 pandemic. No data with personally identifiable data will be collected, nor any data on a patient individual level. Data will be analysed by comparing absolute numbers and percentages of children presenting to emergency departments, the severity of their presenting problems, their working diagnoses, and the patient outcomes, over time during the study period.

Intervention type

Other

Phase

Drug names

Primary outcome measure

Absolute numbers of children presenting to the paediatric emergency department over the period of interest; for all children and children with different typologies (i.e. working diagnosis, age)

Secondary outcome measures

1. The severity of illness of children presenting to the paediatric emergency department over the period of interest as defined by the following criteria: percentage of children with abnormal vital parameters; high triage urgency; a composite outcome of the need for emergency medications, the need for hospital admission for >24 h, the need for PICU admission, and death
2. Change of relative incidence of children with specific diagnoses of interest and the severity of their presentation as a proxy for timeliness of presentations. Calculated from: absolute numbers of children presenting to the paediatric emergency department; the percentage of children with abnormal vital parameters; the number of cases with high triage urgency; a composite outcome of the need for emergency medications, the need for hospital admission for >24 h, the need for PICU admission, and death; over the period of interest and over an equivalent historical time period for comparison

Overall trial start date

14/06/2020

Overall trial end date

31/12/2021

Reason abandoned (if study stopped)

Eligibility

Participant inclusion criteria

1. All children presenting to the emergency department during the period of interest for unscheduled health care
2. Aged between 0 and 18 years (upper age limit determined by the upper age bracket for children being assessed at the local participating centre)
3. Undergo a formal clinical assessment by advanced nurse practitioner (or equivalent) or clinician in the emergency department
4. All or part of the data of the triaging process (including vital signs), consultation, management (including diagnostics and treatment) and outcomes (including working diagnosis and disposition) routinely documented in the electronic patient record

Participant type

Patient

Age group

Child

Gender

Both

Target number of participants

Between 6,000 and 60,000 per participating centre, 40 confirmed participating centres at the time of submission

Participant exclusion criteria

1. Children visiting the emergency department who are then streamed to a primary care service for the initial consultation.
2. Children presenting to the emergency department for scheduled health care or a planned follow-up visit (children who have an unscheduled re-visit to the emergency department within one disease episode are not excluded)

Recruitment start date

01/01/2018

Recruitment end date

01/05/2021

Locations

Countries of recruitment

Austria, England, France, Germany, Hungary, Iceland, Ireland, Italy, Latvia, Lithuania, Malta, Netherlands, Portugal, Slovenia, Spain, Sweden, Turkey, United Kingdom

Trial participating centre

Imperial College
London
W2 1NY
United Kingdom

Trial participating centre

Medical University Vienna
Paediatric Emergency Outpatient Clinic
Clinical Division of Pediatric Pulmonology, Allergology and Endocrinology
Department of Pediatrics and Adolescent Medicine
Währinger Gürtel 18-20
Vienna
1090
Austria

Trial participating centre

Paracelsus Medical University
Paediatric Emergency Department and Paediatric surgery Department
Müllner Hauptstrasse 48
Salzburg
5020
Austria

Trial participating centre

Medical University of Graz
Department of General Paediatrics
Auenbruggerplatz 2
Graz
8036
Austria

Trial participating centre

Hopital Universitaire Robert-Debre
Paediatric Emergency Department
Bd Sérurier
Paris
75019
France

Trial participating centre

Louis Mourier Hospital
Paediatric Emergency Department
178 Rue des Renouillers
Colombes
92700
France

Trial participating centre

Armand Trousseau Hospital
Paediatric Emergency Department
26 avenue du Dr-Arnold-Netter
Paris
75012
France

Trial participating centre

Jean Verdier Hospital
Paediatric Emergency Department
3 Rue Arthur Groussier
Bondy
93140
France

Trial participating centre

Dr. von Hauner Children's Hospital
Paediatric emergency department
Ludwig-Maximilians-University Munich
Lindwurmstraße 4
Munich
80337
Germany

Trial participating centre

Heim Pal National Paediatric Institute
Paediatric Emergency Department
Ulloi ut 86
Budapest
1089
Hungary

Trial participating centre

Szent Gyorgy University Teaching Hospital of Fejer County
Paediatric Emergency Department
Szekesfehervar
8000
Hungary

Trial participating centre

Barnaspitali Hringsins
Hringbraut 101
Reykjavík
101
Iceland

Trial participating centre

Children's Health Ireland at Crumlin
Paediatric Emergency Department
Cooley Rd
Crumlin
Dublin
D12 N512
Ireland

Trial participating centre

Children's Health Ireland at Temple Street
Paediatric Emergency Department
Temple St
Rotunda
Dublin

D01 XD99
Ireland

Trial participating centre

Children's Health Ireland at Tallaght
Paediatric Emergency Department
Tallaght
Dublin
D24 NR0A
Ireland

Trial participating centre

University Hospital of Padova
Division of Paediatric Emergency Medicine
Department of Women's and Children's Health
Via Giustiniani, 3
Padova
35128
Italy

Trial participating centre

ondazione Policlinico Universitario A. Gemelli IRCCS
Department of Woman and Child Health and Public Health
Via della Pineta Sacchetti, 217
Rome
00168
Italy

Trial participating centre

Children's Clinical University Hospital
Paediatric emergency department
Riga Stradins University
Vienības gatve 45
Riga
-
Latvia

Trial participating centre

Hospital of Lithuanian University of Health Sciences Kauno Klinikos
Eivenių g. 2
Kaunas
50161
Lithuania

Trial participating centre

Mater Dei Hospital

Department of Child and Adolescent Health
Msida
MSD 2090
Malta

Trial participating centre

Erasmus MC Sophia
Department General Paediatrics
Dr. Molewaterplein 40
Rotterdam
3015 GD
Netherlands

Trial participating centre

Medisch Centrum Alkmaar, Noordwest Ziekenhuisgroep
Emergency department
Wilhelminalaan 12
Alkmaar
1815 JD
Netherlands

Trial participating centre

Hospital Pediátrico, Centro Hospitalar e Universitário de Coimbra
Pediatric Emergency Service
Avenida, R. Dr. Afonso Romão
Coimbra
3000-602
Portugal

Trial participating centre

Centro Hospitalar e Universitário de São João
Alameda Prof. Hernâni Monteiro
Porto
4200-319
Portugal

Trial participating centre

Hospital Dona Estefania
Centro Hospitalar de Lisboa Central
Alameda Santo António dos Capuchos
Lisboa
1169-050
Portugal

Trial participating centre

Hospital Prof. Doutor Fernando da Fonseca

Departamento da Criança e do Jovem- Urgencia Pediatrica
IC19
Amadora
2720-276
Portugal

Trial participating centre

Centro Hospitalar Tondela-Viseu
Paediatric Department
Av. Rei Dom Duarte
Viseu
3504-509
Portugal

Trial participating centre

University Medical Centre Ljubljana
Univerzitetni Klinični Center
Department of Infectious Diseases
Zaloška cesta 7
Ljubljana
1000
Slovenia

Trial participating centre

Cruces University Hospital
Paediatric emergency department
Cruces Plaza, S/N
Barakaldo
48903
Spain

Trial participating centre

Hospital Universitario Río Hortega
Paediatric emergency unit
Calle Dulzaina, 2
Valladolid
47012
Spain

Trial participating centre

Astrid Lindgrens Children's hospital
Paediatric emergency department
Karolinska University
Anna Stecksséns gata 35
Solna
171 64
Sweden

Trial participating centre

Sachs' Children and Youth Hospital
Paediatric emergency department
Sjukhusbacken 10
Stockholm
118 83
Sweden

Trial participating centre

Faculty of Medicine, Ondokuz Mayıs University
Paediatric Emergency Department
Körfez
19 Mayıs University
Samsun
55270
Turkey

Trial participating centre

Hacettepe University School of Medicine
Division of Pediatric Emergency Medicine
Department of Pediatrics
Hacettepe
A.Adnan Saygun Cd
Ankara
06230
Turkey

Trial participating centre

Mersin City Training and Research Hospital
Department of Pediatrics
Division of Emergency Medicine
Korukent Mah. 96015 Sok. Mersin Entegre Sağlık Kampüsü
Toroslar
Mersin
33240
Turkey

Trial participating centre

Leicester Children's Hospital
Paediatric Emergency Medicine Leicester Academic Group
Children's Emergency Department
Leicester Royal Infirmary
Infirmary Square
Leicester
LE1 5UE
United Kingdom

Trial participating centre

St. Mary’s Hospital
Department of Paediatric Emergency Medicine
Division of Medicine
Imperial College NHS Healthcare Trust
Praed Street
London
W2 NY1
United Kingdom

Trial participating centre

St. Thomas' Hospital
Department of paediatric emergency medicine
Guy's and St. Thomas' NHS Foundation Trust
Westminster Bridge Rd
South Bank
London
SE1 7EH
United Kingdom

Trial participating centre

Birmingham Children's Hospital
Paediatric emergency department
Birmingham women's and children's NHS Foundation Trust
Steelhouse Ln
Birmingham
B4 6NH
United Kingdom

Trial participating centre

Bristol Royal Hospital for Children
Emergency Department
Upper Maudlin St
Bristol
BS2 8BJ
United Kingdom

Trial participating centre

Alder Hey Children's Hospital
Paediatric emergency department
Alder Hey Children's NHS Foundation Trust
E Prescot Rd
Liverpool
L12 2AP
United Kingdom

Sponsor information

Organisation

Imperial College London

Sponsor details

Joint Research Compliance Office
Medical School building
Room 221
Norfolk Place
London
W211PG
England
United Kingdom
+44 (0)207 594 9465
cheuk-fung.wong@imperial.ac.uk [mailto:cheuk-fung.wong@imperial.ac.uk]

Sponsor type

University/education

Website

http://www3.imperial.ac.uk/ [http://www3.imperial.ac.uk/]

GRID

grid.7445.2 [https://www.grid.ac/institutes/grid.7445.2]

Funders

Funder type

Other

Funder name

Investigator initiated and funded

Alternative name(s)

Funding Body Type

Funding Body Subtype

Location

Results and Publications

Publication and dissemination plan

Current publication and dissemination plan as of 01/04/2021:
Expect to publish the main manuscript in a leading international peer-reviewed journal and presentation at international conferences, planned for 01/06/2021 (i.e.: data from original EPISODES study, detailing period January 2018 - May 2020).

Anticipated additional production of short papers on both difficulties on harmonising routinely collected clinical data from European paediatric emergency departments, and changes in health care pathways across Europe amongst participants of the EPISODES study. Secondary analysis of the data may be performed with the approval of the EPISODES steering group after review of a study proposal by any member of the EPISODES study group. Furthermore, the EPISODES study will position the trial group in a unique position to respond rapidly to a potential second wave of Sars-Cov-2 infections and to collect data on epidemiological issues in paediatric emergency medicine.

Previous publication and dissemination plan:
Expect to publication of the main manuscript in a leading international peer-reviewed journal and presentation at international conferences. Anticipated production of short papers on both difficulties on harmonising routinely collected clinical data from European paediatric emergency departments, and changes in health care pathways across Europe amongst participants of the EPISODES study. Secondary analysis of the data may be performed with the approval of the EPISODES steering group after review of a study proposal by any member of the EPISODES study group. Furthermore, the EPISODES study will position the trial group in a unique position to respond rapidly to a potential second wave of Sars-Cov-2 infections and to collect data on epidemiological issues in paediatric emergency medicine.

Intention to publish date

01/06/2021

Individual participant data (IPD) sharing statement

The datasets generated during and/or analysed during the current study are/will be available upon request from r.nijman@imperial.ac.uk. Requests and study proposals will be reviewed by the steering committee. Any of the data needed for any (approved by the steering committee) proposed analysis will be shared. These data will not contain patient individual data and are only available in aggregated and fully anonymised form. UK HRA approval was obtained; no patient informed consent was needed. Data will become available after acceptance of first publication of the main study results. Data will be available for 10 years after study closure.

Participant level data

Available on request

Trial outputs

Output type	Details	Date created	Date added	Peer reviewed?	Patient-facing?

Additional files

Editorial Notes

01/04/2021: The following changes were made to the trial record: 1. The recruitment end date was

changed from 14/05/2020 to 01/05/2021. 2. The overall trial end date was changed from 31/10/2020 to 31/12/2021. 3. The intention to publish date was changed from 01/10/2020 to 01/06/2021. 4. The interventions and publication and dissemination plan were updated. 26/03/2021: The trial participating centres have been added. 16/03/2021: Internal review. 15/07/2020: Internal review. 14/07/2020: Trial’s existence confirmed by HRA and Health and Care Research Wales (HCRW).
