# Supplementary material for: Presentations of children to emergency departments across Europe and the COVID-19 pandemic: A multinational observational study
Source: PLoS Med. 2022 Aug 26;19(8):e1003974. doi: 10.1371/journal.pmed.1003974 (PMC9467376; doi:10.1371/journal.pmed.1003974)
Supplement: S3 File — (PDF) [file pmed.1003974.s004.pdf]

S3 File. Clinical report form

Clinical report form

This form describes the data of children presenting to the emergency department of
Hospital code: UK005

*based on data availability for centre: certain categories will NOT appear if data are not available at all in a setting.

For the month / week [circle which applies]

For Week (February 2020 onwards): period of data entry always describes a period starting on each Monday and finishing on Sunday. For month (January 2018 until February 2020): every first day of the month until last day of the month.

beginning Monday [enter date, DD/MM/YYYY]

ending Sunday [enter date, DD/MM/YYYY]

**Study ID**	[auto generated]		
Local Study ID	[optional: local team to link study ID with month or week of entry]		
Total number of visits[1]	[1]number of visits were seen by physician/ANP or eq. in the PED<br>Include only unplanned urgent and emergency care only; no planned/scheduled medical care.<br>Include 'Left without being seen' in total number of visits.	(n)	
Day of presentation	Monday	(n)	
	Tuesday	(n)	
	Wednesday	(n)	
	Thursday	(n)	
	Friday	(n)	
	Saturday	(n)	
	Sunday	(n)	
	Data not available	*(n)*	
Time of presentation	Day (08.00 – 15.59)	(n)	
	Evening (16.00 – 23.59)	(n)	
	Night (00.00 – 07.59)	(n)	
	Data not available	*(n)*	
Referral	Self referred	(n)	
	General practitioner<br>Or primary care equivalent, such as urgent care centre	(n)	
	Primary paediatrician	(n)	
	Telephone consultation service (e.g. 111 in the UK)	(n)	
	Specialist physician from another or the same hospital	(n)	
	Other	(n)	

	Data not available	*(n)*	
Mode of arrival	Own means of transport<br>This includes public transport, taxi, etc.	(n)	
	Ambulance – emergency services<br>Include air ambulance services	(n)	
	Data not available	*(n)*	
Gender	Male	(n)	
	Female	(n)	
	Data not available	*(n)*	
Age	0 - <14 days	(n)	
	14 days - <3 months	(n)	
	3 months - <1 year	(n)	
	1 – <2 years	(n)	
	2 - <5 years	(n)	
	5 - <12 years	(n)	
	12 - <16 years	(n)	
	16 - <18 years	(n)	
	Data not available	*(n)*	
Triage urgency	Level 1-2<br>Eq: Emergent – very urgent; RED - ORANGE	(n)	
	Level 3<br>Eq: Urgent; YELLOW	(n)	
	Level 4 – 5<br>Eq: Non urgent – standard; GREEN - BLUE	(n)	
	Data not available	*(n)*	
Presenting problem	Trauma<br>Principal presenting problem related to injuries	(n)	
	Medical<br>Principal presenting problem related to acute medical reasons	(n)	
	Other<br>Principal presenting problem related to other reasons	(n)	
	data not available	(n)	
Vital signs	Tachycardia<br>[APLS definition: use 95th centile threshold for age]	(n)	
	Heart rate: Data not available	*(n)*	
	Tachypnoea<br>[APLS definition: use 95th centile threshold for age]	(n)	
	Respiratory rate: Data not available	*(n)*	
	Reduced level of consciousness<br>On AVPU scale: V-P-U; GCS <14	(n)	
	Level of consciousness: Data not available	*(n)*	
	O2 saturations <94%	(n)	
	Oxygen saturations: Data not available	*(n)*	

	Temperature >37.9 °C	(n)	
	Temperature: Data not available	*(n)*	
Diagnostics performed	Blood tests<br>If child had ANY performed, including bloodgas	(n)	
	Microbiology / virology: any	(n)	
	Microbiology / virology: Covid-19	(n)	
	Imaging: CT	(n)	
	Imaging: XR	(n)	
	Imaging: US	(n)	
	Imaging: MRI	(n)	
	Data not available	*(n)*	
Treatment in PED	Any medications prescribed as part of the initial (ED) management plan		
	Antibiotics: oral<br>If presenting on abx, and these are continued or changed PO, include as [yes]	(n)	
	Antibiotics: IM / IV / IO<br>If presenting on abx, and these are changed to IM / IV / IO, include as [yes]	(n)	
	Bronchodilators: per inhaler	(n)	
	Bronchodilators: per nebuliser	(n)	
	Bronchodilators: intravenous<br>Eg: $MgSO_4$, salbutamol, aminophylline	(n)	
	Nebulised medication other	(n)	
	Steroids: inhaled	(n)	
	Steroids: nebulised	(n)	
	Steroids: intravenous	(n)	
	Steroids: oral	(n)	
	Analgesia / Antipyretics: paracetamol PO / IV / PR	(n)	
	Analgesia / Antipyretics / anti-inflammatory: NSAIDs[3] PO	(n)	
	Analgesia: opioids PO / IN / IV	(n)	
	Insulin IV / SC	(n)	
	Inotropes / vasopressors<br>Any iv infusion with adrenaline, noradenaline, dopamine, dobutamine, or eq., started in ED	(n)	
	IV fluid bolus (10 mls.kg; 20 mls.kg; >20 mls.kg))	(n)	
	Covid-19: Hydrochloroquine PO	(n)	
	Covid-19: Chloroquine PO	(n)	
	Covid-19: Azithromycin PO	(n)	
	Other: IVIG		
	Data not available	*(n)*	
Working diagnosis[3]	[3]all patients should have ONE main infectious or non-infectious working diagnosis, as per treating ED clinician at the end of PED consultation<br><br>Every patient should be coded with one diagnosis only.	(n)	

	Infection: Upper respiratory tract/ENT: Otitis Media	(n)	
	Infection: Upper respiratory tract/ENT: Tonsillitis / pharyngitis	(n)	
	Infection: Lower respiratory tract infections	(n)	
	Infection: Gastro-intestinal infections	(n)	
	Infection: Childhood exanthems (e.g.: varicella, roseola)	(n)	
	Infection: Sepsis	(n)	
	Infection: Meningitis / CNS infection	(n)	
	Infection: Inflammatory illness	(n)	
	Infection: Kawasaki	(n)	
	Infection: Undifferentiated fever / fever without focus	(n)	
	Infection: febrile neutropaenia	(n)	
	Infection: Simple febrile seizure	(n)	
	Diabetic keto-acidosis	(n)	
	Testicular torsion	(n)	
	Appendicitis	(n)	
	Intussusception, volvulus, malrotation	(n)	
	Sickle cell crisis / related problems	(n)	
	Mental health illness	(n)	
	Accidental intoxication, ingestion	(n)	
	Alcohol ingestion / Substance misuse	(n)	
	Minor trauma: any radius fracture	(n)	
	Minor trauma: minor head injury	(n)	
	Major trauma<br>includingthose with (pre-arrival) major trauma alert	(n)	
Safeguarding	(Suspected) non-accidental injury<br>Defined as any (suspected) inflicted or any sustained injury with serious safeguarding concerns that triggered involvement of safeguarding team and social services, likely resulting in hospital admission; this excludes cases for information sharing purposes only	(n)	
Outcome	Short stay admission<br>Eq. short stay unit / clinical decision unit / Observation unit [max duration 24 hrs]	(n)	
	Admission to hospital ward	(n)	
	Admission to PICU	(n)	
	Death (in PED)	(n)	
	Left without being seen<br>Patients in (P)ED who did not wait for completion of treatment or consultation; include 'left the department against medical advice'.	(n)	
	Data not available	*(n)*	
	Time in department<br>- 0 - <60 mins<br>- 60-<120 mins	(n)	

	- 120-<240 mins<br>- 240-<480 mins<br>- 480 mins or more<br>From registration in ED to discharge from ED (to any disposition)		
	Duration of admission<br>-<24 hours<br>- 24- <48 hours<br>- 48 - <72 hours<br>- 72 hours or more	(n)	
