# Supplementary material for: Presentations of children to emergency departments across Europe and the COVID-19 pandemic: A multinational observational study
Source: PLoS Med. 2022 Aug 26;19(8):e1003974. doi: 10.1371/journal.pmed.1003974 (PMC9467376; doi:10.1371/journal.pmed.1003974)
Supplement: S1 Table — (PDF) [file pmed.1003974.s006.pdf]

S1 Table. List of time windows for data entry

Time period	Start date	Finish date	Number of days
1	Monday, 1 January 2018	Wednesday, 31 January 2018	31
2	Thursday, 1 February 2018	Wednesday, 28 February 2018	28
3	Thursday, 1 March 2018	Saturday, 31 March 2018	31
4	Sunday, 1 April 2018	Monday, 30 April 2018	30
5	Tuesday, 1 May 2018	Thursday, 31 May 2018	31
6	Friday, 1 June 2018	Saturday, 30 June 2018	30
7	Sunday, 1 July 2018	Tuesday, 31 July 2018	31
8	Wednesday, 1 August 2018	Friday, 31 August 2018	31
9	Saturday, 1 September 2018	Sunday, 30 September 2018	30
10	Monday, 1 October 2018	Wednesday, 31 October 2018	31
11	Thursday, 1 November 2018	Friday, 30 November 2018	30
12	Saturday, 1 December 2018	Monday, 31 December 2018	31
13	Tuesday, 1 January 2019	Thursday, 31 January 2019	31
14	Friday, 1 February 2019	Thursday, 28 February 2019	28
15	Friday, 1 March 2019	Sunday, 31 March 2019	31
16	Monday, 1 April 2019	Tuesday, 30 April 2019	30
17	Wednesday, 1 May 2019	Friday, 31 May 2019	31
18	Saturday, 1 June 2019	Sunday, 30 June 2019	30
19	Monday, 1 July 2019	Wednesday, 31 July 2019	31
20	Thursday, 1 August 2019	Saturday, 31 August 2019	31
21	Sunday, 1 September 2019	Monday, 30 September 2019	30
22	Tuesday, 1 October 2019	Thursday, 31 October 2019	31
23	Friday, 1 November 2019	Saturday, 30 November 2019	30
24	Sunday, 1 December 2019	Tuesday, 31 December 2019	31
25	Wednesday, 1 January 2020	Sunday, 2 February 2020	33
26	Monday, 3 February 2020	Sunday, 9 February 2020	7
27	Monday, 10 February 2020	Sunday, 16 February 2020	7
28	Monday, 17 February 2020	Sunday, 23 February 2020	7
29	Monday, 24 February 2020	Sunday, 1 March 2020	7
30	Monday, 2 March 2020	Sunday, 8 March 2020	7
31	Monday, 9 March 2020	Sunday, 15 March 2020	7

32	Monday, 16 March 2020	Sunday, 22 March 2020	7
33	Monday, 23 March 2020	Sunday, 29 March 2020	7
34	Monday, 30 March 2020	Sunday, 5 April 2020	7
35	Monday, 6 April 2020	Sunday, 12 April 2020	7
36	Monday, 13 April 2020	Sunday, 19 April 2020	7
37	Monday, 20 April 2020	Sunday, 26 April 2020	7
38	Monday, 27 April 2020	Sunday, 3 May 2020	7
39	Monday, 4 May 2020	Sunday, 10 May 2020	7
40	Monday, 11 May 2020	Sunday, 17 May 2020	7
