# Supplementary material for: Presentations of children to emergency departments across Europe and the COVID-19 pandemic: A multinational observational study
Source: PLoS Med. 2022 Aug 26;19(8):e1003974. doi: 10.1371/journal.pmed.1003974 (PMC9467376; doi:10.1371/journal.pmed.1003974)
Supplement: S2 Table — (PDF) [file pmed.1003974.s007.pdf]

S2 Table. ICD-10 Guidance for coding of diagnosis

**PED working diagnosis**	**Examples**	**Corresponding ICD-10 diagnosis codes**
Infection: Upper respiratory/ENT: otitis media	Acute otitis media; otitis externa; mastoiditis	H60.1-H60.9, H62.4 otitis externa<br>H65.0-H67.8 otitis media and similar<br>H70, H75.0 mastoiditis
Infection: Upper respiratory/ENT: tonsillitis / pharyngitis	Tonsillitis, pharyngitis, quinsy / peritonsillar abscess	B08.5 herpangina<br>B00.2 gingivostomatitis / pharingotonsillitis<br>B27.8-B27.9 mononucleosis<br>J02.0-J03.9 tonsillitis, pharyngitis<br>J36, J39.0 peritonsillar abscess
Infection: Lower respiratory tract infections	Bronchiolitis, pneumonia, lower respiratory tract infection, empyema, pleural effusion, pertussis, undefined LRTI, tracheitis	A37.1-A37.9 pertussis<br>B25.0 Cytomegaloviral pneumonitis<br>J04.1-J04.2 tracheitis<br>J09 Influenza due to pandemic influenza virus<br>J10-J12 influenza and other viral pneumonia<br>J13.0-J15.9 bacterial pneumonia<br>J16.0-18.9 other pneumonia<br>J20.1 – J22.0 other LRTIs<br>J85.0-J86.9 empyema and lung abscess<br>J90.0, J91.0 pleural effusion
Infection: Gastro-intestinal infections	Gastro-enteritis, diarrheal illness, episodes of vomiting with other infections ruled out, pancreatitis, hepatitis, mesenteric adenitis	A02.0-A.02.9 Salmonella<br>A04.0-A04.9 bacterial intestinal infection<br>A05.0-A05.9 bacterial foodborn intoxication<br>A06.0-A07.9 protozoal intestinal disease<br>A08.0-A09.0 gastroenteritis with viral or unspecified origin<br>B15.0, B15.9 Hepatitis A w or w/o hepatic coma<br>B16.0-B16.9 Hepatitis B<br>B17.0-B17.8 other viral Hepatitis<br>B18.0-B18.9 chronic viral hepatitis<br>K29.1, K29.7 acute / simple gastritis<br>K71.0-K71.9 hepatic disease with toxic origin<br>K72.0, K72.9 hepatic failure<br>K75.0-K75.9 other inflammatory liver disease (Excl.: acute, subacute, viral or toxic hepatitis)<br>I88.0 mesenteric lymphadenitis<br>K85.0 pancreatitis<br>R11 vomiting<br>R19.5 other fecal abnormalities
Diabetic keto-acidosis	Diabetic keto-acidosis	E10.1 DKA with coma<br>E13.1, E14.1 Other diabetes with DKA
Testicular torsion	Testicular torsion, or torsion testicular appendix	N44.0 Testicular torsion
Volvulus, malrotation, intussusception	Volvulus, malrotation, intussusception	K56.1 Intussusception<br>K56.2 Volvulus<br>Q43.3 Malrotation of colon
Appendicitis	Appendicitis plus/minus peritonitis	K35.0, K35.2 Appendicitis with generalised peritonitis<br>K35.1, K35.3 Appendicitis with localised peritonitis (abscess)<br>K35.8, K35.9, K36.0, K37.0 Appendicitis, other or unspecified
Mental health illness	Mental health illness, non-accidental intoxication, suicidal ideation or attempt(s), depression, manic episode,	X60-X84 intoxications, suicidal attempts<br>F20-F29 psychotic disorders<br>F30-F39 affective disorders

	hallucinations, schizophrenia, anorexia, other eating disorder, self-harm, psychosis, delirium	F40-F48 Neurotic disorders<br>F50.0, F50.1, F50.3 Anorexia<br>R45.8 Other symptoms and signs involving emotional state
Radius fracture		S52.1, S52.3, S52.5, S52.9<br>S59.1, S59.2<br>M84.33, M84.43, M84.53, M84.63
Minor head injury		S00.0-S00.<br>(not: S00.05, S00.06, S00.07, S00.35, S00.36, S00.37, S00.85, S00.86, S00.87, S00.95, S00.96, S00.97)
