# Supplementary material for: Presentations of children to emergency departments across Europe and the COVID-19 pandemic: A multinational observational study
Source: PLoS Med. 2022 Aug 26;19(8):e1003974. doi: 10.1371/journal.pmed.1003974 (PMC9467376; doi:10.1371/journal.pmed.1003974)
Supplement: S3 Table — (PDF) [file pmed.1003974.s008.pdf]

S3 Table. List of approvals

Name of participating hospital	Side code	Date of data sharing agreement	Date of ethics approval	Ethics reference number
Emergency department, Univ. Klinik für Kinder- und Jugendheilkunde, Vienna, Austria	AUS001	19-08-2020	18-09-2020	32-643 ex 19/10
Paediatric Emergency Department and Paediatric surgery Department, Paracelsus Medical University, Salzburg, Austria	AUS003	07-09-2020	18-09-2020	32-643 ex 19/10
Medical University of Graz, Department of General Paediatrics, Graz, Austria	AUS004	28-02-2021	18-09-2020	32-643 ex 19/10
Paediatric Emergency Department, Hopital Universitaire Robert-Debre, Paris, France	FR001	23-07-2020	01-08-2020	N/A
Paediatric Emergency Department, Louis Mourier Hospital, Colombes, France	FR002	24-07-2020	01-08-2020	N/A
Paediatric Emergency Department, Armand Trousseau Hospital, Paris, France	FR003	02-09-2020	01-08-2020	N/A
Paediatric Emergency Department, Jean Verdier Hospital, Bondy, France	FR004	05-10-2020	01-08-2020	N/A
Paediatric emergency department, Dr. von Hauner Children's Hospital, Ludwig-Maximilians-University Munich, Munich, Germany	GER001	26-07-2020	11-08-2020	50-597
Paediatric Emergency Department, Heim Pal National Paediatric Institute, Budapest, Hungary	HUN001	10-08-2020	15-07-2020	KUT-26/2020
Paediatric Emergency Department, Szent Gyorgy University Teaching Hospital of Fejer County, Szekesfehervar, Hungary	HUN002	23-09-2020	31-07-2020	17/2020.07.30
Children´s Hospital, Barnaspitali Hringsins, Reykjavik, Iceland	ICE001	21-08-2020	01-09-2020	VSN-20-140
Paediatric Emergency Department, Children's Health Ireland at Crumlin, Ireland	IRE001	20-08-2020	14-08-2020	20-NREC-COV-089
Paediatric Emergency Department, Children's Health Ireland at Temple Street, Ireland	IRE002	20-08-2020	14-08-2020	20-NREC-COV-089
Division of Paediatric Emergency Medicine, Department of Women's and Children's Health – University Hospital of Padova, Italy	IT001	15-09-2020	24-08-2020	44435
Department of Woman and Child Health and Public Health, Fondazione Policlinico Universitario A. Gemelli IRCCS, Rome, Italy	IT002	16-12-2020	19-11-2020	3497
Paediatric emergency department, Children's Clinical University Hospital, Riga, Latvia	LAT001	13-08-2020	23-07-2020	Nr.6-1/08/11
Hospital of Lithuanian University of Health Sciences Kauno Klinikos, Lithuania	LIT001	19-01-2021	14-01-2021	SUT-211-0112 SUTP-2893
Department of Child and Adolescent Health, Mater Dei Hospital, Msida, Malta	MAL001	19-10-2020	12-10-2020	HEC07.20
Department general Paediatrics, ErasmusMC – Sophia, Rotterdam, The Netherlands	NL001	07-09-2020	23-06-2020	MEC-2020-0471
Emergency department, Medisch Centrum Alkmaar, Noordwest Ziekenhuisgroep, Alkmaar, The Netherlands	NL002	07-09-2020	23-12-2020	L 020 – 100
Hospital Pediátrico, Centro Hospitalar e Universitário de Coimbra, Portugal	POR001	10-09-2020	28-10-2020	CHUC-127-20

Hospital Dona Estefania, Centro Hospitalar de Lisboa Central, Portugal	POR003	11-06-2021	15-10-2020	939/2020
Hospital Prof. Doutor Fernando da Fonseca, Amadora, Portugal	POR004	06-08-2020	03-08-2020	86/2020
Emergency department, Centro Hospitalar Tondela-Viseu, Viseu, Portugal	POR005	05-08-2020	02-07-2020	N/A
University Medical Centre Ljubljana, Univerzitetni Klinični Center, Department of Infectious Diseases, Ljubljana, Slovenia	SLO001	15-12-2020	07-09-2020	0120-359/2020/3
Paediatric emergency department, Cruces University Hospital, Barakaldo, Spain	SP001	28-07-2020	24-09-2020	PI2020161
Paediatric emergency unit, Hospital Universitario Río Hortega, Valladolid, Spain	SP002		19-10-2020	PI185-20
Paediatric emergency department, Astrid Lindgrens Children's hospital, Karolinska University, Sweden	SWE001		07-10-2020	2020-04717
Paediatric emergency department, Sachs' Children and Youth Hospital, Stockholm, Sweden	SWE002	21-05-2021	07-10-2020	2020-04717
Ondokuz Mayıs University Paediatric Emergency Department in Samsun, Turkey	TUR001	18-08-2020	24-07-2020	B.30.2.ODM.0.20.08 /502
Emergency department, Ihsan Dogramaci Children's Hospital, Hacettepe University School of Medicine, Ankara, Turkey	TUR002	22-09-2020	22-09-2020	16969557-1333 GP 20/846
Mersin City Training and Research Hospital, Department of Pediatrics, Division of Emergency Medicine, Toroslar, Mersin, Turkey	TUR003	02-03-2021	03-03-2021	E-66792640-903.07.01
Paediatric Emergency Medicine Leicester Academic Group, Leicester Hospitals, Leicester, UK	UK001	07-07-2020	18-06-2020	284008 – 20SM6003
Department of Paediatric Emergency Medicine, Division of Medicine, St. Mary's hospital - Imperial College NHS Healthcare Trust, London, UK	UK002	N/A	18-06-2020	284008 – 20SM6003
Paediatric emergency department, Birmingham women's and children's NHS Foundation Trust, Birmingham, UK	UK004	23-08-2020	18-06-2020	284008 – 20SM6003
Paediatric emergency department, University Hospitals Bristol NHS Foundation Trust, Bristol, UK	UK005	07-08-2020	18-06-2020	284008 – 20SM6003
Paediatric emergency department, Alder Hey Children's NHS Foundation Trust, Liverpool, UK	UK006	17-09-2020	18-06-2020	284008 – 20SM6003
