# Supplementary material for: Presentations of children to emergency departments across Europe and the COVID-19 pandemic: A multinational observational study
Source: PLoS Med. 2022 Aug 26;19(8):e1003974. doi: 10.1371/journal.pmed.1003974 (PMC9467376; doi:10.1371/journal.pmed.1003974)
Supplement: S4 Table — (PDF) [file pmed.1003974.s009.pdf]

S4 Table. Overview of participating study sites

Hospital Code	Type of hospital	Description of the emergency department	Geographical area	Pediatric services in hospital	Pediatric inpatient beds	PICU and/or HDU Beds	Number of levels in Triage Tool	Upper age limit (years)
AUS001	University/tertiary	Tertiary-care PED in a mixed hospital	Urban	Tertiary care adults and children, most pediatric subspecialties	64	8	5	18
AUS003	University/tertiary	Tertiary-care PED in a mixed hospital	Urban	Tertiary care adults and children, most pediatric subspecialties	104	6	5	18
AUS004	University/tertiary	Tertiary-care PED of a standalone children's hospital	Mixed urban and rural	Stand-alone pediatric hospital, tertiary care, most subspecialties	107	12	5	18
FR001	University/tertiary	Tertiary-care PED of a standalone children's hospital	Urban	Stand-alone pediatric hospital, tertiary care, most subspecialties	120	20	5	16
FR002	University/tertiary	Tertiary-care PED in a mixed hospital	Urban	General pediatrics in a general hospital, no subspecialties	16	NA	4	16
FR003	University/tertiary	Tertiary-care PED of a standalone children's hospital	Urban	Stand-alone pediatric hospital, tertiary care, most subspecialties	50	10	4	18
FR004	University/tertiary	Tertiary-care PED in a mixed hospital	Urban	Tertiary care adults and children, up to five pediatric subspecialties	35	4	5	16
GER001	University/tertiary	Tertiary-care PED of a standalone children's hospital	Urban	Stand-alone pediatric hospital, tertiary care, most subspecialties	84	14	5	18
HUN001	University/tertiary	Tertiary-care PED of a standalone children's hospital	Mixed urban and rural	Stand-alone pediatric hospital, tertiary care, most subspecialties	514	27	5	18
HUN002	University/tertiary	Tertiary-care PED in a mixed hospital	Mixed urban and rural	Tertiary care adults and children, most pediatric subspecialties	50	8	5	18
ICE001	University/tertiary	Tertiary-care PED of a standalone children's hospital	Urban	Stand-alone pediatric hospital, tertiary care, most subspecialties	28	NA	5	18
IRE001	University/tertiary	Tertiary-care PED of a standalone children's hospital	Urban	Stand-alone pediatric hospital, tertiary care, most subspecialties	233	25	5	16
IRE002	University/tertiary	Tertiary-care PED of a standalone children's hospital	Urban	Stand-alone pediatric hospital, tertiary care, most subspecialties	85	9	5	16
IRE003	University/tertiary	Tertiary-care PED in a mixed hospital	Urban	Tertiary care adults and children, up to five pediatric subspecialties	44	3 HDU	5	16
IT001	University/tertiary	Tertiary-care PED in a mixed hospital	Urban	Tertiary care adults and children, most pediatric subspecialties	125	8	4	15

IT002	University/tertiary	Tertiary-care PED in a mixed hospital	Urban	Tertiary care adults and children, most pediatric subspecialties	60	7	5	17
LAT001	University/tertiary	Tertiary-care PED of a standalone children's hospital	Urban	Stand-alone pediatric hospital, tertiary care, most subspecialties	230	11	5	18
LIT001	University/tertiary	Tertiary-care PED in a mixed hospital	Urban	Tertiary care adults and children, most pediatric subspecialties	169	8	4	18
MAL001	University/tertiary	Tertiary-care PED in a mixed hospital	Mixed urban and rural	Tertiary care adults and children, most pediatric subspecialties	50	10	5	16
NL001	University/tertiary	Tertiary-care PED in a mixed hospital	Mixed urban and rural	Tertiary care adults and children, most pediatric subspecialties	80	20	5	18
NL002	Teaching/DGH	General ED for both adults and children (non-university hospital)	Rural	General pediatric in a general hospital, up to five pediatric subspecialties	15	NA	6	18
POR001	University/tertiary	Tertiary-care PED of a standalone children's hospital	Urban	Stand-alone pediatric hospital, tertiary care, most subspecialties	96	12	5	18
POR003	University/tertiary	Tertiary-care PED of a standalone children's hospital	Urban	Stand-alone pediatric hospital, tertiary care, most subspecialties	115	15	5	18
POR004	Teaching/DGH	General ED with a pediatric section and dedicated PED DRs (non-university hospital)	Urban	General pediatric in a general hospital, up to five pediatric subspecialties	38	11	5	18
POR005	Teaching/DGH	General ED with a pediatric section and dedicated PED DRs (non-university hospital)	Mixed urban and rural	General pediatric in a general hospital, up to five pediatric subspecialties	43	NA	5	18
SL0001	University/tertiary	Tertiary-care PED in a mixed hospital	Mixed urban and rural	Tertiary care adults and children, up to five pediatric subspecialties	46	NA	NA	18
SP001	University/tertiary	Tertiary-care PED in a mixed hospital	Urban	Tertiary care adults and children, most pediatric subspecialties	100	12	5	14
SP002	University/tertiary	Tertiary-care PED in a mixed hospital	Mixed urban and rural	Tertiary care adults and children, up to five pediatric subspecialties	26	6	5	14
SWE001	University/tertiary	Tertiary-care PED in a mixed hospital	Urban	Tertiary care adults and children, most pediatric subspecialties	100	19	5	17
SWE002	University/tertiary	Tertiary-care PED in a mixed hospital	Urban	Tertiary care adults and children, up to five pediatric subspecialties	26	2	5	17
TUR001	University/tertiary	Tertiary-care PED in a mixed hospital	Mixed urban and rural	Tertiary care adults and children, most pediatric subspecialties	80	17	3	18
TUR002	University/tertiary	Tertiary-care PED of a standalone children's hospital	Urban	Stand-alone pediatric hospital, tertiary care, most subspecialties	300	16	5	18
TUR003	Teaching/DGH	Tertiary-care PED in a mixed hospital	Urban	Tertiary care adults and children, most pediatric subspecialties	150	30	3	18

UK001	University/tertiary	Tertiary-care PED in a mixed hospital	Urban	Tertiary care adults and children, up to five pediatric subspecialties	87	10	4	15
UK002	University/tertiary	Tertiary-care PED in a mixed hospital	Urban	Tertiary care adults and children, most pediatric subspecialties	34	15	5	16
UK004	University/tertiary	Tertiary-care PED of a standalone children's hospital	Urban	Stand-alone pediatric hospital, tertiary care, most subspecialties	222	62	5	16
UK005	University/tertiary	Tertiary-care PED of a standalone children's hospital	Urban	Stand-alone pediatric hospital, tertiary care, most subspecialties	127	36	4	16
UK006	University/tertiary	Tertiary-care PED of a standalone children's hospital	Urban	Stand-alone pediatric hospital, tertiary care, most subspecialties	251	48	5	16

DGH: district general hospital, HDU: high dependency unit, PED: pediatric emergency department, PICU pediatric intensive care unit
