# Supplementary material for: Presentations of children to emergency departments across Europe and the COVID-19 pandemic: A multinational observational study
Source: PLoS Med. 2022 Aug 26;19(8):e1003974. doi: 10.1371/journal.pmed.1003974 (PMC9467376; doi:10.1371/journal.pmed.1003974)
Supplement: S5 Table — (PDF) [file pmed.1003974.s010.pdf]

S5 Table. List of national social distancing measures

Response Measure	Austria	France	Germany	Hungary	Iceland	Ireland	Italy	Latvia	Lithuania
Education									
Closure of educational institutions (Daycare and nurseries)	2020-03-16 to 2020-05-04	2020-03-16 to 2020-06-01	2020-03-13 to 2020-05-14	2020-03-25 to 2020-06-02		2020-03-12 to 2020-06-29	2020-03-10 to 2020-06-10	-	2020-03-16 to 2020-04-26
Closure of educational institutions (Daycare and nurseries) - Partially relaxed measure	-	2020-06-02 to 2020-06-22	2020-05-15 to 2020-07-01	-		-	2020-03-04 to 2020-03-09; 2020-06-11 ::	2020-03-17 to 2020-06-10	2020-04-27 ::
Closure of educational institutions (Higher education)	2020-03-16 to [n/a]	-		2020-03-25 to 2020-05-18	2020-03-16 to 2020-05-04	2020-03-12 ::	2020-03-10 to 2020-06-10	2020-03-17 ::	2020-03-16 to 2020-05-24
Closure of educational institutions (Higher education) - Partially relaxed measure	-	2020-03-16 to 2020-06-22		-	-	-	2020-03-04 to 2020-03-09; 2020-06-11 ::	-	2020-05-25 to 2020-06-23
Closure of educational institutions (Primary school)	2020-03-16 to 2020-05-18	-	2020-03-13 to 2020-05-03	2020-03-16 to 2020-06-01		2020-03-12 ::	2020-03-11 ::	2020-03-17 ::	2020-03-16 to 2020-05-24
Closure of educational institutions (Primary school) - Partially relaxed measure	-	2020-03-16 to 2020-06-22	2020-05-04 to 2020-06-29	2020-06-02 to 2020-06-26		-	2020-03-04 to 2020-03-10	-	2020-05-25 to 2020-06-23
Closure of educational institutions (Secondary school)	2020-03-16 to 2020-05-03	2020-03-16 to 2020-05-10	2020-03-13 to 2020-05-03	2020-03-16 to 2020-06-01	2020-03-16 to 2020-05-04	2020-03-12 ::	2020-03-04 to 2020-06-16	-	2020-03-16 to 2020-05-31
Closure of educational institutions (Secondary school) - Partially relaxed measure	2020-05-04 to 2020-06-03	2020-05-11 to 2020-06-22	2020-05-04 to 2020-06-15	2020-06-02 to 2020-06-26	-	-	2020-06-17 ::	2020-03-17 to 2020-05-07	2020-06-01 to 2020-06-16
Public spaces and events									
Closure of public spaces (any)	2020-03-16 to 2020-04-13	2020-03-16 to 2020-05-10	2020-03-16 to 2020-05-04	2020-03-13 to 2020-05-18	2020-03-16 to 2020-05-03	2020-03-12 to 2020-03-26; 2020-06-06 to 2020-06-29	2020-03-10 to 2020-03-10	2020-03-17 to 2020-05-11	-
Closure of public spaces (any) - Partially relaxed measure	2020-04-14 to [n/a]	2020-05-11 to 2020-06-22	2020-05-05 to 2020-06-08	-	2020-05-04 to 2020-06-15	2020-03-27 to 2020-06-05	2020-03-11 to 2020-06-15	2020-05-12 to 2020-06-10; 2020-07-11 ::	2020-03-16 to 2020-07-17
Mass gathering cancellations	2020-03-10 to 2020-07-31	2020-02-29 ::	2020-03-08 to 2020-05-10	2020-03-11 to 2020-06-11	2020-03-16 to 2020-05-24; 2020-08-01 ::	2020-03-12 ::	2020-03-10 to 2020-07-30	2020-03-17 to 2020-06-30	2020-03-12 to 2020-05-03; 2020-07-01 ::
Mass gathering cancellations - Partially relaxed measure	-	-	2020-05-11 ::	-	2020-05-25 to 2020-07-31	-	2020-07-31 ::	2020-07-01 ::	2020-05-04 to 2020-06-30

MassGather50 - Partially relaxed measure		-	-	2020-05-11 to 2020-06-29	-	-	-	-	-	2020-05-04 to 2020-06-16
Face masks										
Protective mask use in public spaces/transport (Mandatory for general population)		2020-04-06 to 2020-04-30	2020-05-18 ::	-		-	2020-06-29 ::	2020-04-14 to 2020-07-14	-	2020-04-10 to 2020-04-29
Protective mask use in public spaces/transport (Mandatory for general population) - Partially relaxed measure		2020-05-01 to [n/a]	-	2020-04-27 ::		-	-	2020-07-15 ::	2020-03-17 to 2020-06-30	2020-04-30 to 2020-06-16; 2020-08-01 ::
Protective mask use in public spaces/transport (Voluntary for general population)		-	2020-05-11 to 2020-05-17	2020-04-02 to 2020-04-26		2020-07-31 ::	2020-05-18 to 2020-06-28	-	-	2020-03-23 to 2020-04-09; 2020-06-17 to 2020-07-31
Protective mask use in public spaces/transport (Voluntary for general population) - Partially relaxed measure		2020-03-31 to 2020-04-05	-	-		-	-	-	-	-
Stay-at-home and lockdown										
Stay-at-home recommendations		2020-03-10 to 2020-03-15	-	2020-03-17 to 2020-05-05	2020-03-21 to 2020-03-26		2020-03-24 to 2020-03-26; 2020-05-18 to 2020-06-26	-	2020-03-17 to 2020-05-11	2020-03-16 to 2020-06-16
Stay-at-home recommendations (general population) - Partially relaxed measure		-	-	2020-05-06 to 2020-06-29	-		2020-03-12 to 2020-03-23	-	2020-05-12 to 2020-06-10	-
Stay-at-home orders (enforced)		2020-03-16 to 2020-04-30	2020-03-17 to 2020-05-11	-	2020-03-27 to 2020-05-18		2020-03-27 to 2020-05-17	2020-03-10 to 2020-05-04	-	-
Stay-at-home orders (enforced) - Partially relaxed measure		-	2020-05-12 to 2020-06-02	-	-		-	-	-	-
Stay-at-home recommendations (risk groups)		-	-	-	-	2020-03-16 ::	2020-03-12 ::	2020-03-04 ::	-	-
Stay-at-home recommendations (risk groups) - Partially relaxed measure		-	-	-	-	-	-		-	-
Workspace										
Teleworking recommendations/workplace closures		2020-03-10 to [n/a]	2020-03-17 to 2020-05-10				2020-03-12 to 2020-06-07	2020-03-12 ::	2020-03-17 ::	2020-03-10 to 2020-06-15
Teleworking recommendations/workplace closures - Partially relaxed measure		-	2020-05-11 ::				2020-06-08 ::	-	-	2020-06-16 ::

Response Measure	Malta	Netherlands	Portugal	Slovenia	Spain	Sweden	Turkey	United Kingdom
Education								
Closure of educational institutions (Daycare and nurseries)	2020-03-13 ::	2020-03-16 to 2020-05-10	2020-03-16 to 2020-05-17	2020-03-12 to 2020-05-18	2020-03-12 ::		2020-03-16 to 2020-05-31	2020-03-23 to 2020-05-31
Closure of educational institutions (Daycare and nurseries) - Partially relaxed measure	-	2020-05-11 to 2020-06-08	2020-05-18 to 2020-05-31	-	-		2020-06-01 to 2020-06-19	2020-06-01 ::
Closure of educational institutions (Higher education)	2020-03-13 to 2020-06-30	2020-03-12 to 2020-06-14	2020-03-16 to 2020-05-17	2020-03-12 ::	2020-03-12 ::	2020-03-18 to 2020-06-15	2020-03-16 to (n/a)	
Closure of educational institutions (Higher education) - Partially relaxed measure	-	2020-06-15 ::	2020-05-18 ::	-	-	-	-	
Closure of educational institutions (Primary school)	2020-03-13 to 2020-06-30	2020-03-16 to 2020-05-10	2020-03-16 ::	2020-03-12 to 2020-05-17	2020-03-12 ::		2020-03-16 to 2020 -06-19	2020-03-23 to 2020-05-31
Closure of educational institutions (Primary school) - Partially relaxed measure	-	2020-05-11 to 2020-06-08	-	2020-05-18 to 2020-06-01	-		-	2020-06-01 ::
Closure of educational institutions (Secondary school)	2020-03-13 to 2020-06-30	2020-03-16 to 2020-06-01	2020-03-16 to 2020-05-17	2020-03-12 to 2020-05-31	2020-03-12 ::	2020-03-18 to 2020-06-15	2020-03-16 to 2020 -06-19	2020-03-23 to 2020-06-14
Closure of educational institutions (Secondary school) - Partially relaxed measure	-	2020-06-02 to 2020-06-30	2020-05-18 ::	2020-06-01 ::	-	-	-	2020-06-15 ::
Public spaces and events								
Closure of public spaces (any)	2020-03-18 to 2020-05-21	-	2020-03-13 to 2020-03-17	2020-03-15 to 2020-04-28	2020-03-14 to 2020-05-03		2020-03-11 to 2020-04-26	2020-03-16 to 2020-05-09
Closure of public spaces (any) - Partially relaxed measure	2020-05-22 ::	2020-03-12 ::	2020-03-18 ::	2020-04-29 ::	2020-05-04 ::		2020-04-27 to (N/a)	2020-05-10 ::
Mass gathering cancellations	2020-03-13 ::	2020-03-12 to 2020-06-30	2020-03-15 ::	2020-03-07 to 2020-07-08	2020-03-14 ::	2020-03-12 ::	2020-03-11 to 2020-05-31	2020-03-24 ::
Mass gathering cancellations - Partially relaxed measure	-	2020-07-01 ::	-	-	-	-	2020-06-01 to (n/a)	-
MassGather50 - Partially relaxed measure	-		-	-	-	-	-	-
Face masks								
Protective mask use in public spaces/transport (Mandatory for general population)	2020-05-22 ::	2020-06-01 ::	2020-05-04 ::	2020-03-30 to 2020-05-31; 2020-06-25 ::	2020-05-04 ::		2020-05-11 to (n/a)	2020-07-27 ::

Protective mask use in public spaces/transport (Mandatory for general population) - Partially relaxed measure		-	-	-	2020-06-01 to 2020-06-24	-		-	-
Protective mask use in public spaces/transport (Voluntary for general population)		-	-	-	-	2020-03-13 to 2020-05-03		-	2020-06-09 to 2020-07-26
Protective mask use in public spaces/transport (Voluntary for general population) - Partially relaxed measure		-	-	-	-	-		-	-
Stay-at-home and lockdown									
Stay-at-home recommendations			2020-03-16 to 2020-05-10	2020-03-19 to 2020-05-02	-	2020-03-09 to 2020-03-13		-	2020-03-16 to 2020-03-23
Stay-at-home recommendations (general population) - Partially relaxed measure			2020-05-11 to 2020-06-01	-	-	-		-	-
Stay-at-home orders (enforced)			-	-	2020-03-19 to 2020-05-04	2020-03-14 to 2020-05-03		2020-03-11 to 2020-04-261	2020-03-24 to 2020-05-09
Stay-at-home orders (enforced) - Partially relaxed measure			-	-	-	2020-05-04 to 2020-05-11		2020-04-27 to 2020-05-31	2020-05-10 to 2020-07-04
Stay-at-home recommendations (risk groups)		2020-03-28 ::	2020-03-13 to 2020-06-26	2020-03-18 ::	-	2020-03-12 ::	2020-03-20 ::	2020-03-21 to 2020-06-06	2020-03-16 to 2020-07-05
Stay-at-home recommendations (risk groups) - Partially relaxed measure		-	-	-	-	-	-	2020-06-07 to (n/a)	2020-07-06 to 2020-08-01
Workspace									
Teleworking recommendations/workplace closures		2020-05-08 ::	2020-03-12 to 2020-05-10	2020-03-16 to 2020-05-31	2020-03-04 ::	2020-03-09 to 2020-04-12	2020-03-16 ::	2020-03-22 to 2020-05-31	2020-03-16 to 2020-05-09
Teleworking recommendations/workplace closures - Partially relaxed measure		-	2020-05-11 ::	2020-06-01 ::	-	2020-04-13 ::	-	2020-06-01 to (n/a)	2020-05-10 ::

Legend:

Dates for the introduction of national social distancing measures for the domains: education, public spaces and events, face masks, stay-at-home recommendations, and the workspace for the countries participating in the EPISODES study. The colored circles indicate the degree of social distancing measures with the most stringent in red, to green for the least restrictive. The areas boxed in red indicate domains with no social distancing measures for a country. Source: https://www.ecdc.europa.eu/en/covid-19/ ; for Turkey: https://www.icisleri.gov.tr
