# Supplementary material for: Presentations of children to emergency departments across Europe and the COVID-19 pandemic: A multinational observational study
Source: PLoS Med. 2022 Aug 26;19(8):e1003974. doi: 10.1371/journal.pmed.1003974 (PMC9467376; doi:10.1371/journal.pmed.1003974)
Supplement: S6 Table — The dates and numbers of SARS-CoV02 infections in each of the study sites’ countries participating in the EPISODES study. (PDF) [file pmed.1003974.s011.pdf]

S6 Table. List of national SARS-CoV-2 rates

Country	Date of 1st reported case	Date of 1st 100 cases	Date of Highest 14-day cumulative rate of new cases per 100.000	Highest cumulative 14-day rate of new cases per 100.000
**Austria**	2020-02-26	2020-03-09	2020-04-02	102.33
**France**	2020-01-25	2020-03-01	2020-04-11	86.12
**Germany**	2020-01-28	2020-03-01	2020-04-09	86.36
**Hungary**	2020-03-05	2020-03-21	2020-04-23	13.34
**Iceland**	2020-02-29	2020-03-13	2020-04-03	277.04
**Ireland**	2020-03-01	2020-03-15	2020-04-23	213.02
**Italy**	2020-01-31	2020-02-24	2020-04-02	124.03
**Latvia**	2020-03-03	2020-03-21	2020-04-06	20.52
**Lithuania**	2020-02-28	2020-03-22	2020-04-04	25.12
**Malta**	2020-03-07	2020-03-22	2020-04-12	46.20
**Netherlands**	2020-02-28	2020-03-07	2020-04-19	86.57
**Portugal**	2020-03-03	2020-03-14	2020-04-11	109.02
**Slovenia**	2020-03-05	2020-03-14	2020-04-05	28.55
**Spain**	2020-02-01	2020-03-02	2020-04-05	217.56
**Sweden**	2020-02-01	2020-03-07	2020-05-01	83.60
**Turkey**	2020-03-12	2020-03-19	2020-04-22	74.97
**United Kingdom**	2020-02-01	2020-03-04	2020-05-01	99.25

Legend:
The dates and numbers of SARS-CoV02 infections in each of the study sites' countries participating in the EPISODES study [1].

1. European Centre for Disease Prevention and Control. COVID-19 [Internet]. 2020. Available from: https://www.ecdc.europa.eu/en/covid-19
