# Supplementary material for: Presentations of children to emergency departments across Europe and the COVID-19 pandemic: A multinational observational study
Source: PLoS Med. 2022 Aug 26;19(8):e1003974. doi: 10.1371/journal.pmed.1003974 (PMC9467376; doi:10.1371/journal.pmed.1003974)
Supplement: S7 Table — (PDF) [file pmed.1003974.s012.pdf]

S7 Table. Sensitivity analyses: poisson regression models for ED attendances

	Incidence Rate Ratio (95% Confidence Interval)	*p value*
**Number of COVID-19 measures in hospital$**		
No measures – reference group		
1 measure	0·426 (0·391 to 0·463)	<0·001
2 measures	0·760 (0·717 to 0·806)	<0·001
3 measures	0·454 (0·413 to 0·499)	<0·001
4+ measures	0·130 (0·108 to 0·155)	<0·001
**SARS-CoV-2 prevalence^**		
Low prevalence	2·616 (2·197 to 3·144)	<0·001
**Type of hospital**		
Mixed tertiary hospital – reference group		
Standalone tertiary children's hospital	3·486 (2·891 to 4·237)	<0·001
General non-university teaching hospital	2·728 (2·276 to 3·297)	<0·001
Urban – reference group		
Urban and rural mixed	5·327 (4·434 to 6·452)	<0·001
**Age Group**		
0-<12 months – reference group		
12-<24 months	0·888 (0·859 to 0·917)	<0·001
2-<5 years	0·841 (0·816 to 0·866)	<0·001
5-<12 years	0·736 (0·712 to 0·761)	<0·001
12-18 years	0·744 (0·714 to 0·773)	<0·001
**Triage urgency classification**		
Non-urgent and standard triage categories – reference group		
Urgent	1·193 (1·170 to 1·279)	<0·001
Emergent and very urgent	1·409 (1·370 to 1·449)	<0·001
**Diagnosis I**		
Appendicitis – reference group		
Gastro-intestinal infections	0·295 (0·267 to 0·326)	<0·001
Minor head injury	0·749 (0·678 to 0·829)	<0·001
LRTI	0·339 (0·306 to 0·333)	<0·001
Mental health issues	0·650 (0·573 to 0·738)	<0·001
Otitis media	0·218 (0·193 to 0·246)	<0·001
Radius fracture	0·724 (0·648 to 0·811)	<0·001
Tonsillitis	0·372 (0·337 to 0·412)	<0·001
**Diagnosis II**		
Surgical presentation – appendicitis – reference group		
Communicable diseases	0·316 (0·267 to 0·326)	<0·001
Other	0·727 (0·678 to 0·829)	<0·001
**Outcome**		
Admission – reference group		

Death	1·829 (0·916 to 3·210)	0·077
PICU Admission	1·134 (1·000 to 1·279)	0·045

Legend:

To derive IRRs, the predicted counts for each individual site were used as an offset in the Poisson model to account for case-mix differences between sites.

^ Low prevalence countries were defined as a cumulative 14-day rate of <= 80 new cases per 100,000 of the population as per the European Centre for Disease Prevention and Control (ECDC) [1]

$ The number of changes made in each hospital in response to the pandemic as previously detailed by Rose et al. [2].

LRTI: lower respiratory tract infection, PICU: pediatric intensive care unit

1. European Centre for Disease Prevention and Control. COVID-19 [Internet]. 2020. Available from: https://www.ecdc.europa.eu/en/covid-19
2. Rose K, Bressan S, Honeyford K, Bognar Z, Buonsenso D, Da Dalt L, et al. Responses of paediatric emergency departments to the first wave of the COVID-19 pandemic in Europe: a cross-sectional survey study. Rybak A, Simões AS, Chiaretti A, Haraldsson A, Gomez B, Aupiais C, et al., editors. *BMJ Paediatr Open* [Internet] 2021;**5**(1). Available from: https://bmjpaedsopen.bmj.com/content/5/1/e001269
