# Supplementary material for: Presentations of children to emergency departments across Europe and the COVID-19 pandemic: A multinational observational study
Source: PLoS Med. 2022 Aug 26;19(8):e1003974. doi: 10.1371/journal.pmed.1003974 (PMC9467376; doi:10.1371/journal.pmed.1003974)

S1 Fig. Spiderplot for availability of data

Legend:

Spider plot showing availability of data (in %, with outer ring representing 100%, and each ring showing a 10% increase) for the 10 data domains.

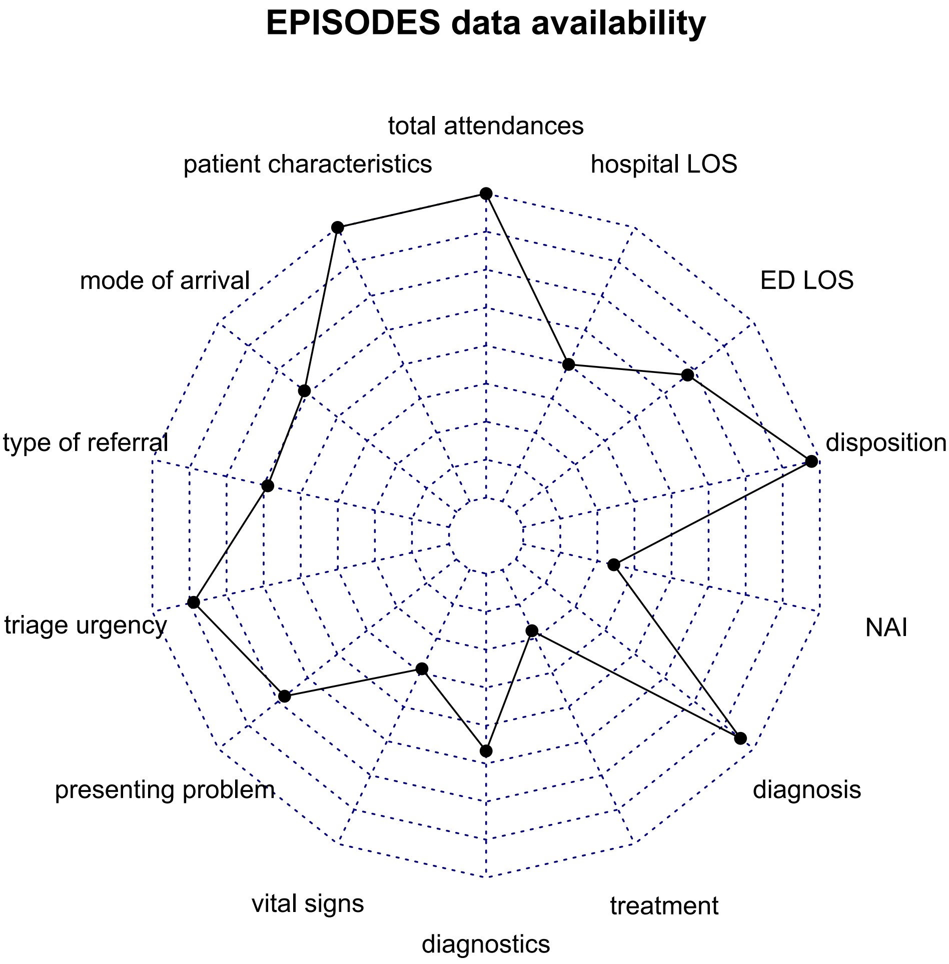

Supplement: S1 Fig — (PDF) [file pmed.1003974.s013.pdf]
