# Supplementary material for: Presentations of children to emergency departments across Europe and the COVID-19 pandemic: A multinational observational study
Source: PLoS Med. 2022 Aug 26;19(8):e1003974. doi: 10.1371/journal.pmed.1003974 (PMC9467376; doi:10.1371/journal.pmed.1003974)

S2 Fig. Map of European with all participating study sites

Legend:

All participating study sites are highlighted with their study site ID; represented countries in red. This map has been generated via 'rnaturalearth' in R, a package built using Natural Earth map data.

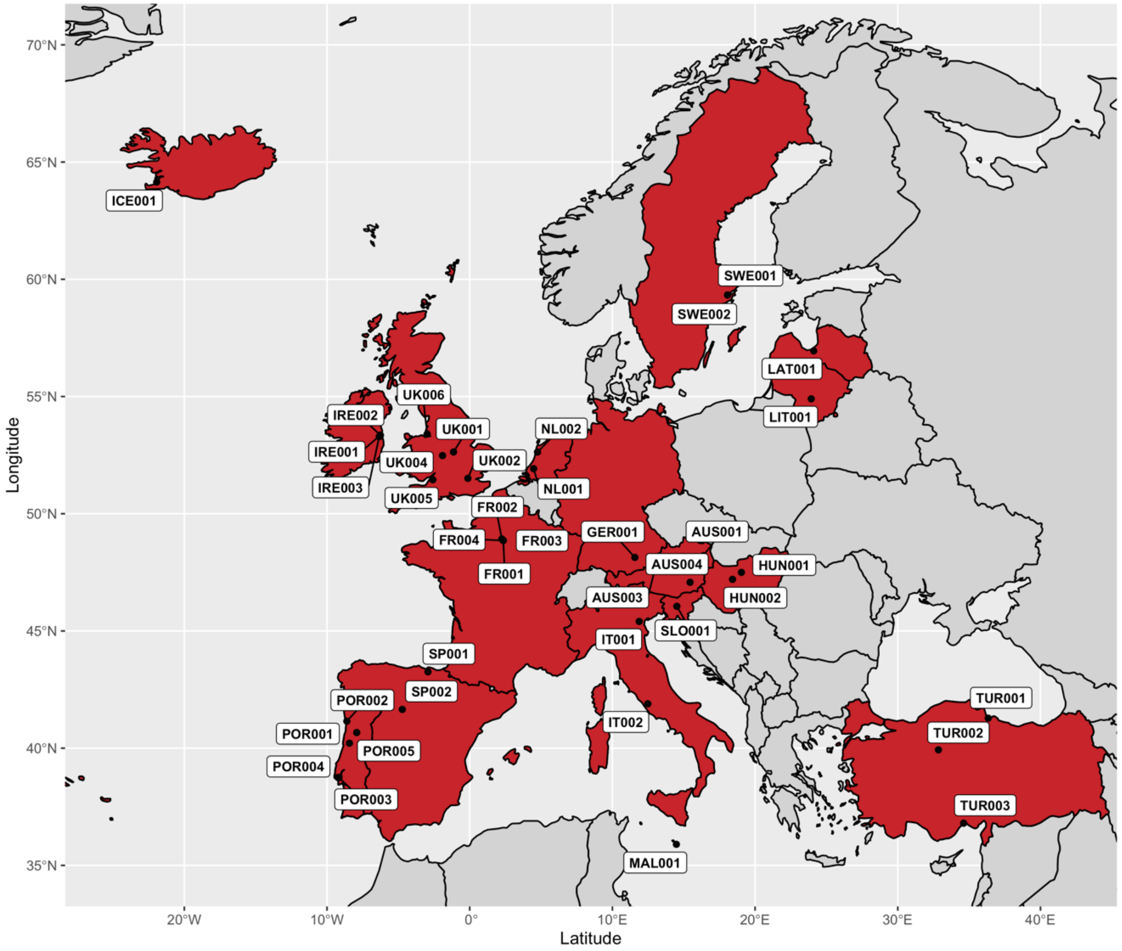

Supplement: S2 Fig — All participating study sites are highlighted with their study site ID; represented countries in red. (PDF) [file pmed.1003974.s014.pdf]
