# Supplementary material for: Presentations of children to emergency departments across Europe and the COVID-19 pandemic: A multinational observational study
Source: PLoS Med. 2022 Aug 26;19(8):e1003974. doi: 10.1371/journal.pmed.1003974 (PMC9467376; doi:10.1371/journal.pmed.1003974)

S3 Fig. Annual emergency department attendance numbers in 2018 and 2019

Legend:

The total number of pediatric emergency departments attendances for each of the study sites for 2018 and 2019 (pre-COVID-19). Overall, the numbers of ED attendances in 2018 and 2019 were similar for each of the study sites, with considerable diversity between the study sites and TUR003 seeing more children in their ED than the other study sites.

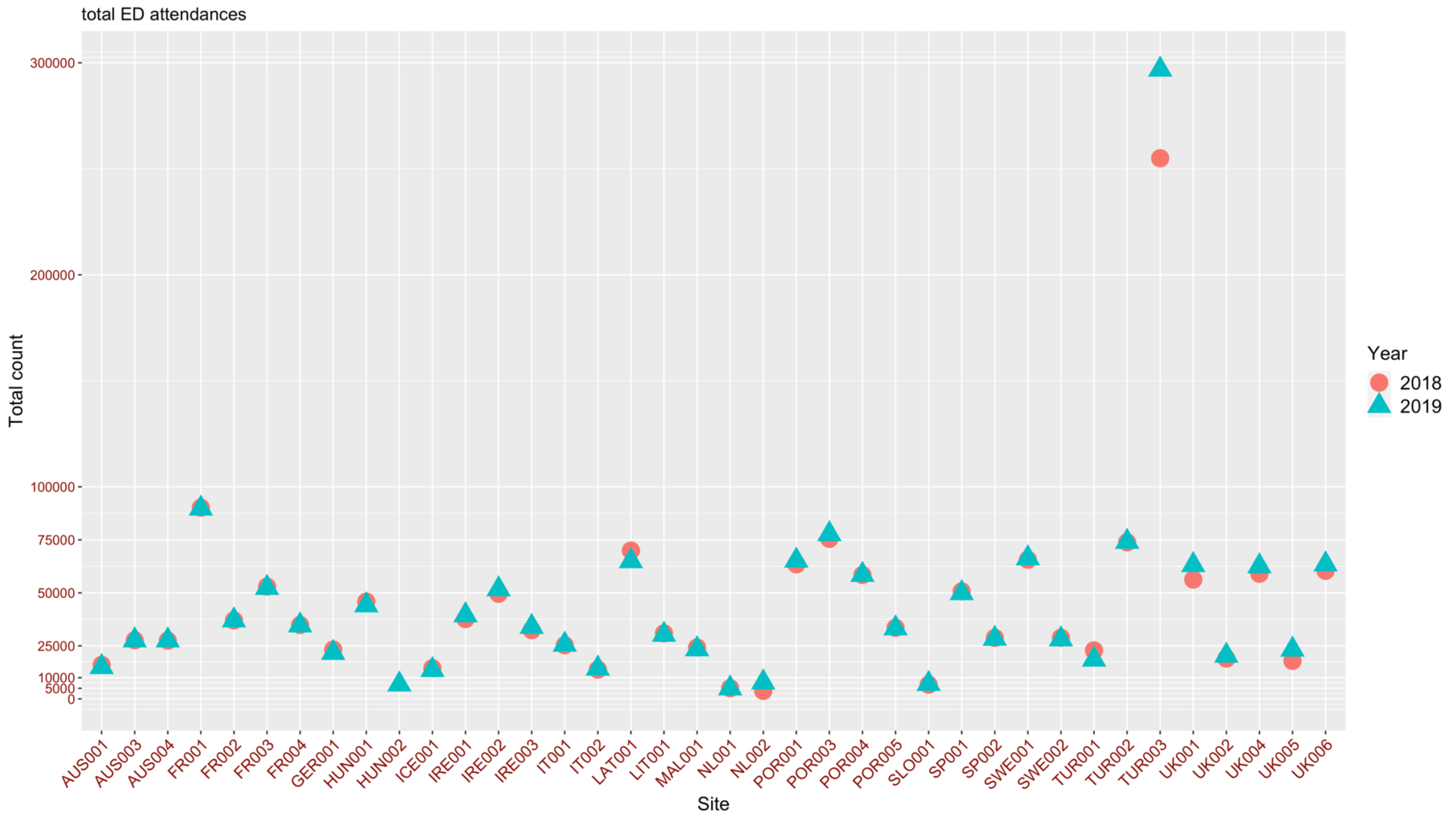

Supplement: S3 Fig — The total number of pediatric ED attendances for each of the study sites for 2018 and 2019 (pre-COVID-19). Overall, the numbers of ED attendances in 2018 and 2019 were similar for each of the study sites, with considerable diversity between the study sites and TUR003 seeing more children in their ED than the other study sites. (PDF) [file pmed.1003974.s015.pdf]
