# Supplementary material for: Presentations of children to emergency departments across Europe and the COVID-19 pandemic: A multinational observational study
Source: PLoS Med. 2022 Aug 26;19(8):e1003974. doi: 10.1371/journal.pmed.1003974 (PMC9467376; doi:10.1371/journal.pmed.1003974)

S4 Fig. Annual emergency department attendance numbers 2018 to 2020 for all sites separately

Legend:

The total number of pediatric emergency departments attendances for each of the study sites for the entire study duration (January 2018 – May 2020). The y-axis is depicted in log scale.

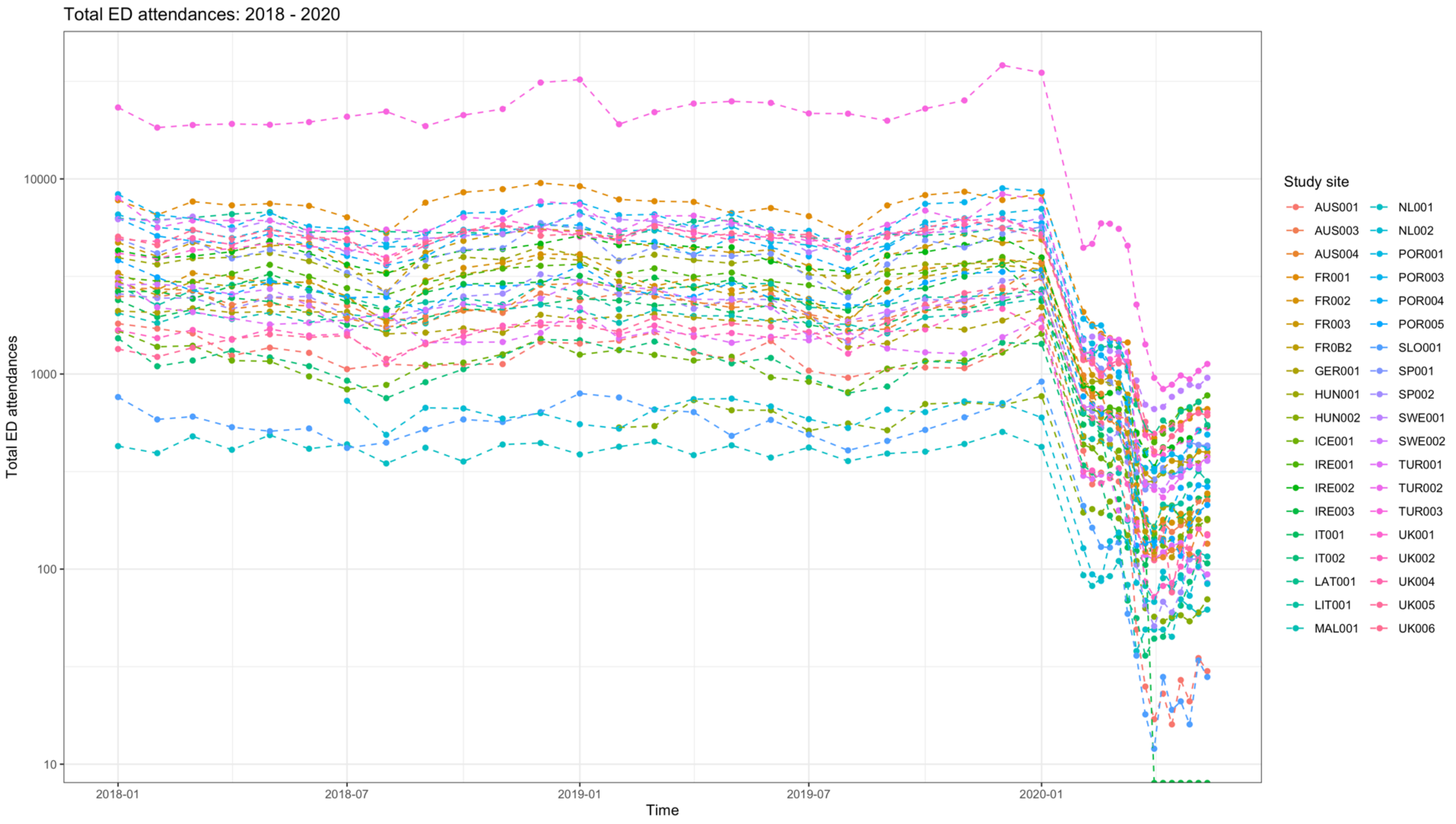

Supplement: S4 Fig — The total number of pediatric ED attendances for each of the study sites for the entire study duration (January 2018–May 2020). The y-axis is depicted in log scale. (PDF) [file pmed.1003974.s016.pdf]
