# Supplementary material for: Presentations of children to emergency departments across Europe and the COVID-19 pandemic: A multinational observational study
Source: PLoS Med. 2022 Aug 26;19(8):e1003974. doi: 10.1371/journal.pmed.1003974 (PMC9467376; doi:10.1371/journal.pmed.1003974)
Supplement: S5 Fig — The observed versus predicted number of children presenting to EDs in countries across Europe in the weeks following February 2, 2020 until May 11, 2020, for all sites combined, for children (a) aged 0–1 years; (b) 1–2 years; (c) 2–5 years; (d) 5–12 years; and (e) 12–18 years. The color and the size of the dots reflect the actual number of ED attendances for each site and for each time window. The line connects the mean of the observed vs. predicted point estimates for each of the individual sites for each time window. (PDF) [file pmed.1003974.s017.pdf]

S5 Fig. Observed versus predicted emergency department attendances (%) for different age categories

Legend:

The observed versus predicted number of children presenting to emergency departments in countries across Europe in the weeks following February 2nd 2020 until May 11th 2020, for all sites combined, for children a) aged 0-1 years, b) 1-2 years, c) 2-5 years, d) 5-12 years, e) 12 – 18 years. The color and the size of the dots reflect the actual number of ED attendances for each site and for each time window. The line connects the mean of the observed vs predicted point estimates for each of the individual sites for each time window.

Age group: 0 - <1 years

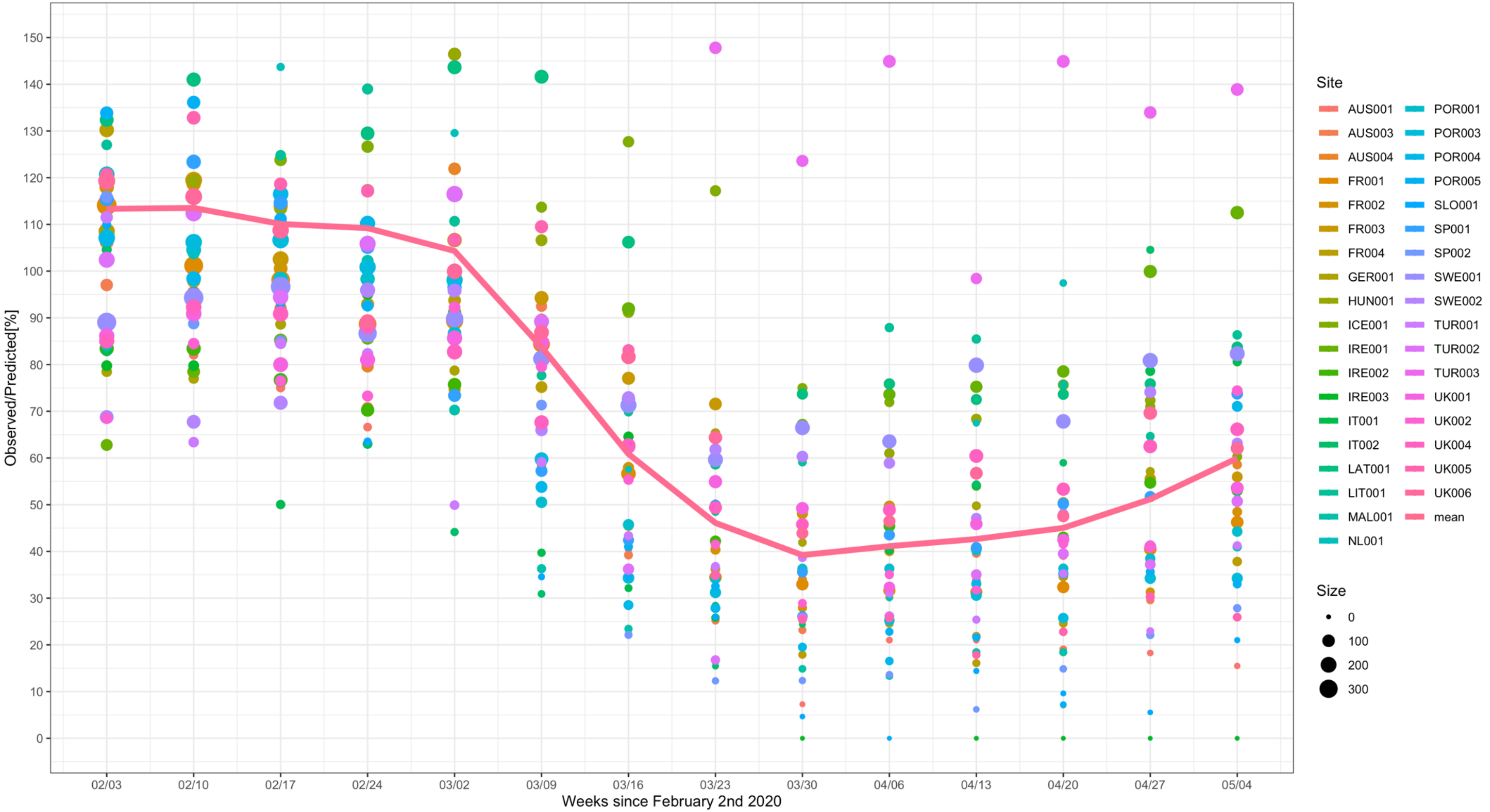

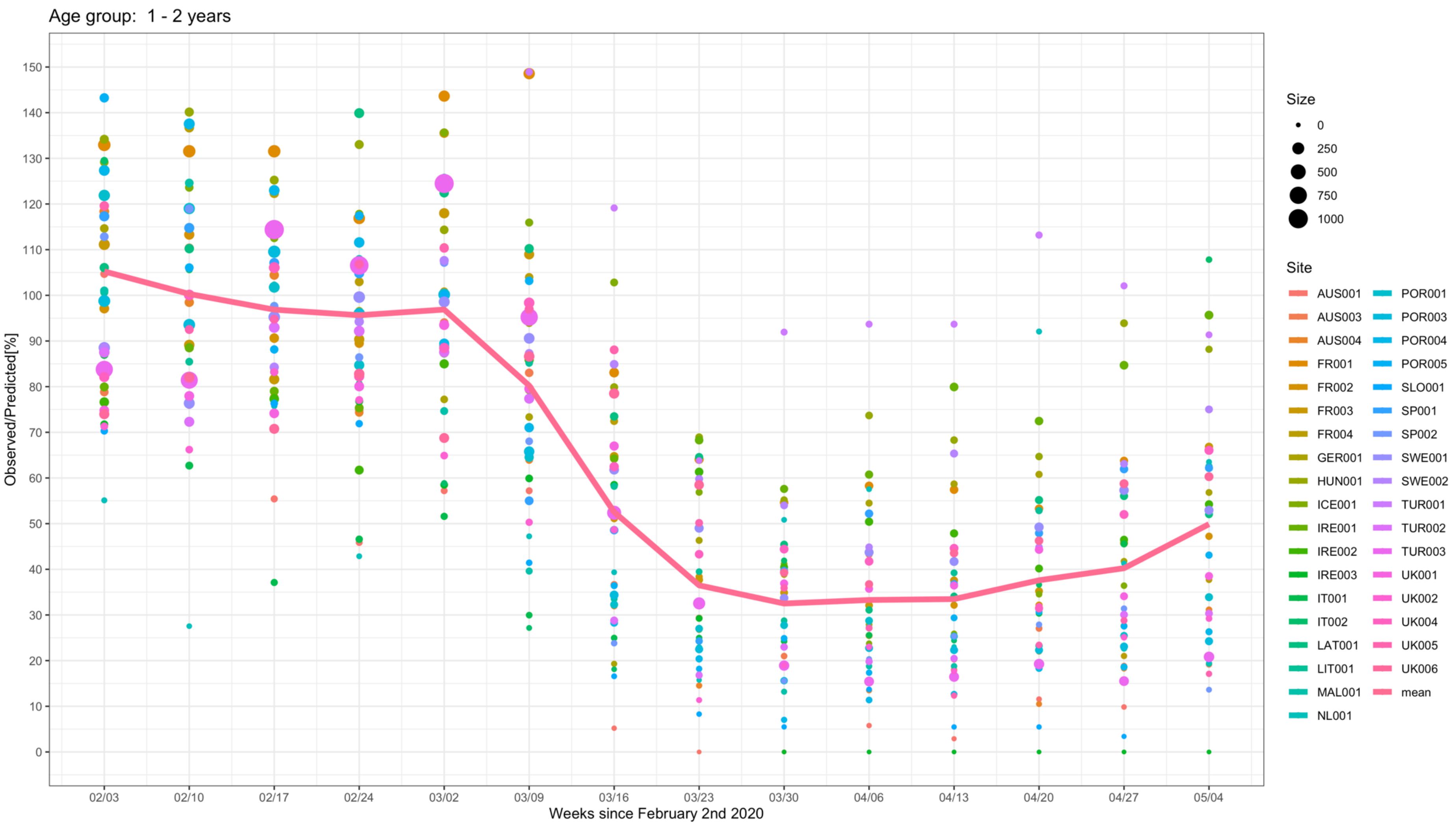
Age group: 1 - 2 years
Observed/Predicted[%]
Weeks since February 2nd 2020
0
10
20
30
40
50
60
70
80
90
100
110
120
130
140
150
02/03
02/10
02/17
02/24
03/02
03/09
03/16
03/23
03/30
04/06
04/13
04/20
04/27
05/04
Size
0
250
500
750
1000
Site
AUS001
AUS003
AUS004
FR001
FR002
FR003
FR004
GER001
HUN001
ICE001
IRE001
IRE002
IRE003
IT001
IT002
LAT001
LIT001
MAL001
NL001
POR001
POR003
POR004
POR005
SLO001
SP001
SP002
SWE001
SWE002
TUR001
TUR002
TUR003
UK001
UK002
UK004
UK005
UK006
mean

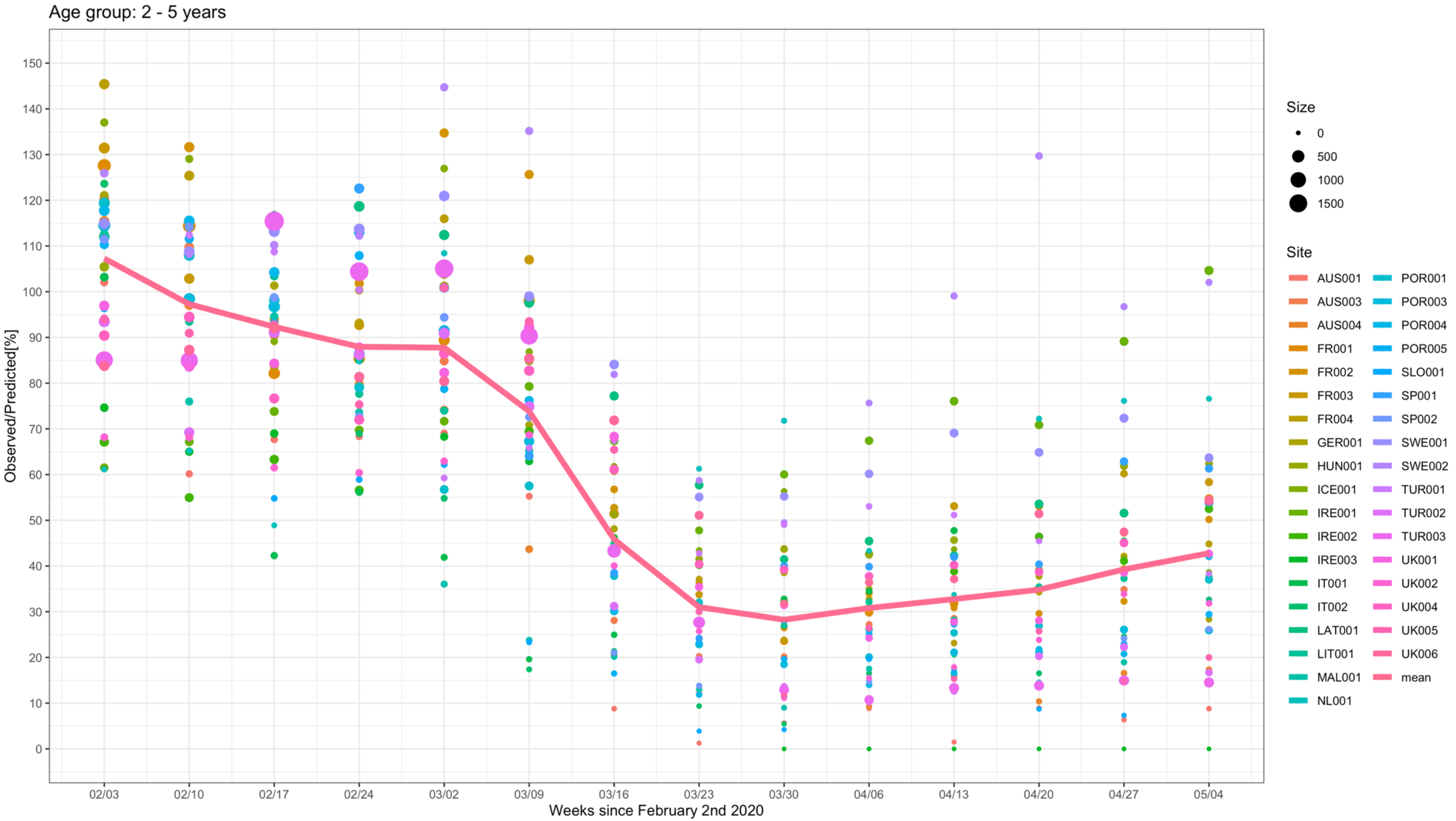
Age group: 2 - 5 years
Observed/Predicted[%]
0
10
20
30
40
50
60
70
80
90
100
110
120
130
140
150
02/03
02/10
02/17
02/24
03/02
03/09
03/16
03/23
03/30
04/06
04/13
04/20
04/27
05/04
Weeks since February 2nd 2020
Size
0
500
1000
1500
Site
AUS001
AUS003
AUS004
FR001
FR002
FR003
FR004
GER001
HUN001
ICE001
IRE001
IRE002
IRE003
IT001
IT002
LAT001
LIT001
MAL001
NL001
POR001
POR003
POR004
POR005
SLO001
SP001
SP002
SWE001
SWE002
TUR001
TUR002
TUR003
UK001
UK002
UK004
UK005
UK006
mean

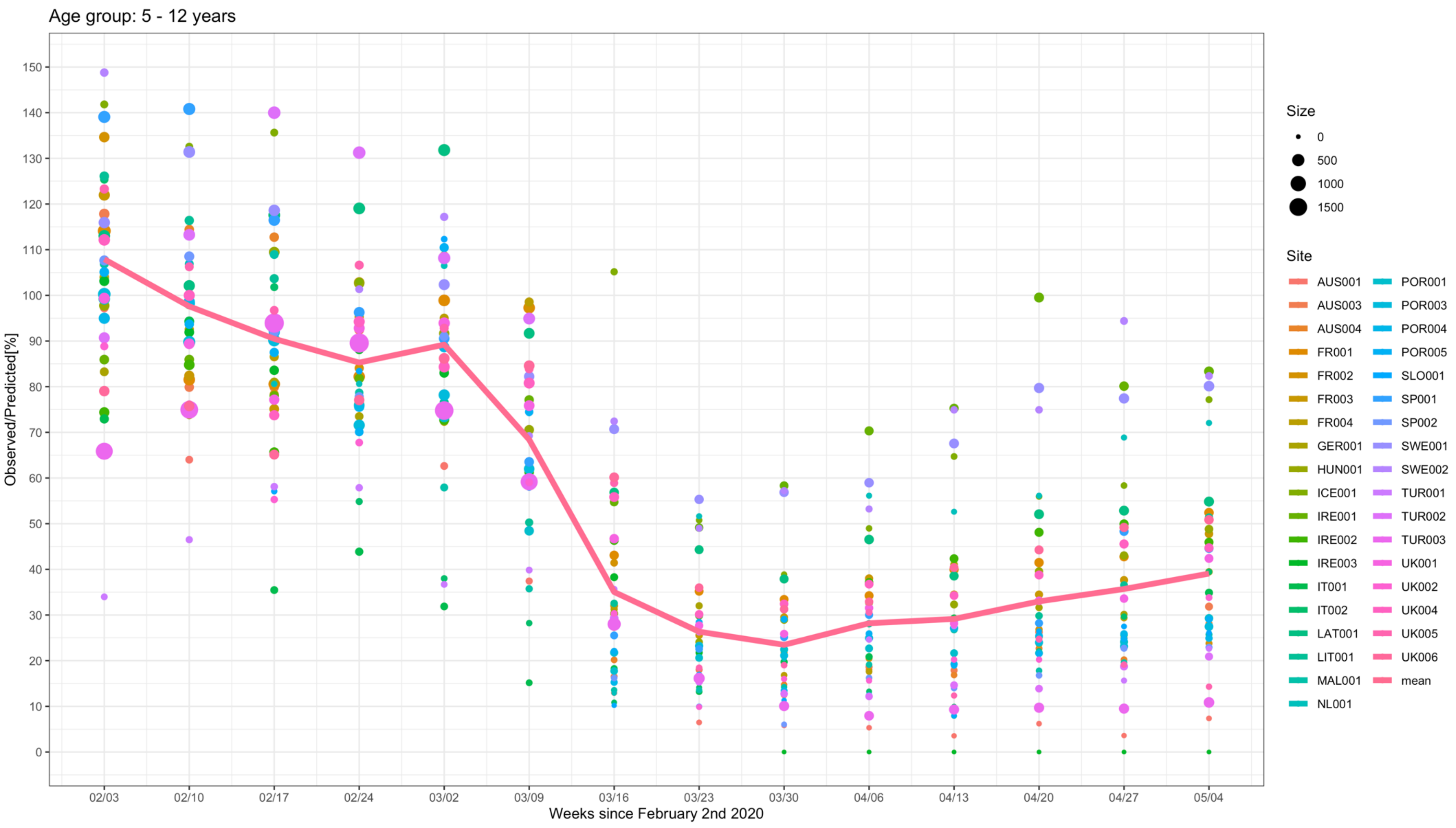
Age group: 5 - 12 years
Observed/Predicted[%]
150
140
130
120
110
100
90
80
70
60
50
40
30
20
10
0
02/03
02/10
02/17
02/24
03/02
03/09
03/16
03/23
03/30
04/06
04/13
04/20
04/27
05/04
Weeks since February 2nd 2020
Size
0
500
1000
1500
Site
AUS001
AUS003
AUS004
FR001
FR002
FR003
FR004
GER001
HUN001
ICE001
IRE001
IRE002
IRE003
IT001
IT002
LAT001
LIT001
MAL001
NL001
POR001
POR003
POR004
POR005
SLO001
SP001
SP002
SWE001
SWE002
TUR001
TUR002
TUR003
UK001
UK002
UK004
UK005
UK006
mean

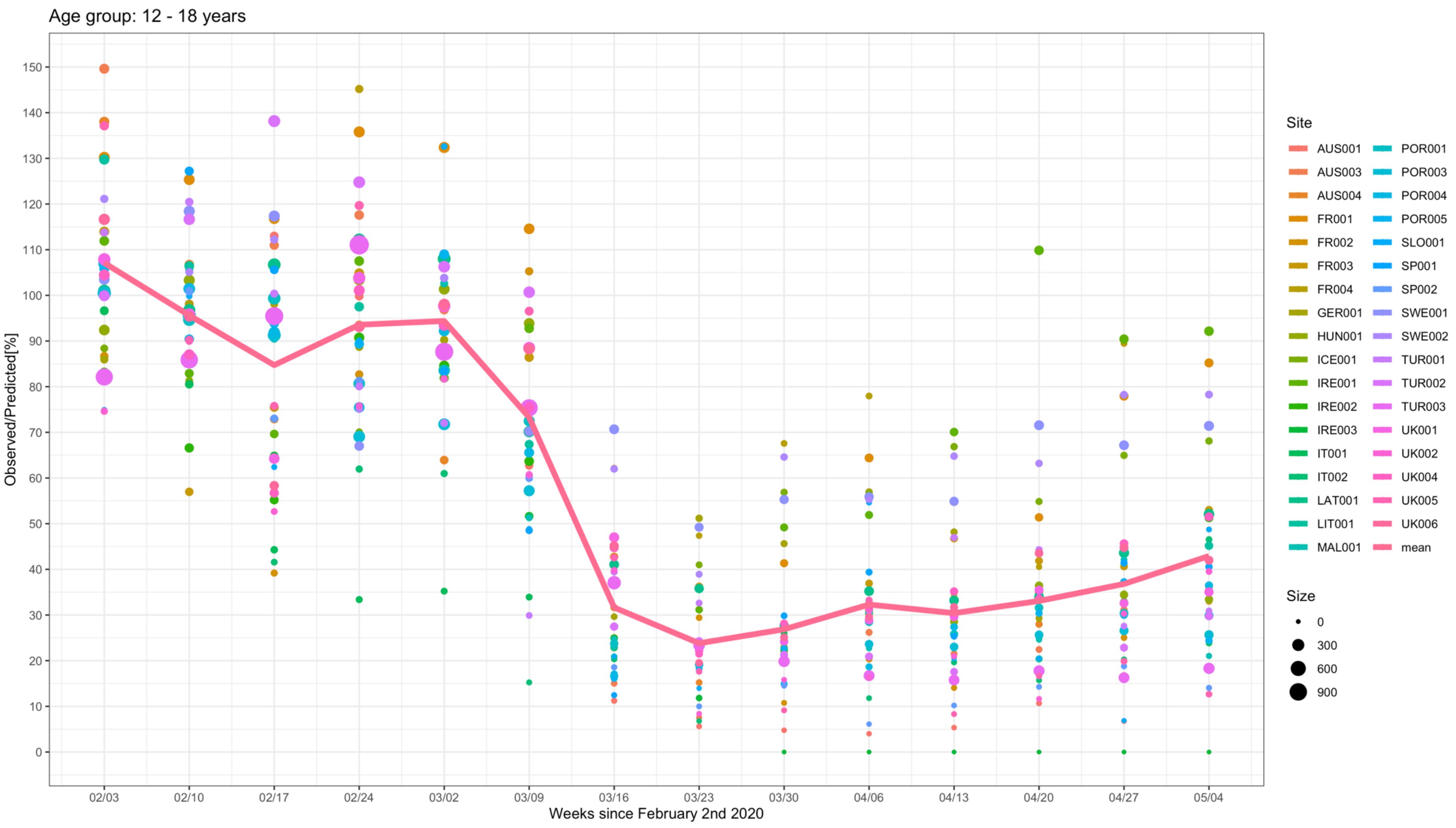
Age group: 12 - 18 years
Observed/Predicted[%]
150
140
130
120
110
100
90
80
70
60
50
40
30
20
10
0
02/03
02/10
02/17
02/24
03/02
03/09
03/16
03/23
03/30
04/06
04/13
04/20
04/27
05/04
Weeks since February 2nd 2020
Site
AUS001
AUS003
AUS004
FR001
FR002
FR003
FR004
GER001
HUN001
ICE001
IRE001
IRE002
IRE003
IT001
IT002
LAT001
LIT001
MAL001
POR001
POR003
POR004
POR005
SLO001
SP001
SP002
SWE001
SWE002
TUR001
TUR002
TUR003
UK001
UK002
UK004
UK005
UK006
mean
Size
0
300
600
900
