# Supplementary material for: Presentations of children to emergency departments across Europe and the COVID-19 pandemic: A multinational observational study
Source: PLoS Med. 2022 Aug 26;19(8):e1003974. doi: 10.1371/journal.pmed.1003974 (PMC9467376; doi:10.1371/journal.pmed.1003974)

S6 Fig. Observed versus predicted emergency department attendances (%) for different age categories, for individual sites

Austria: AUS001

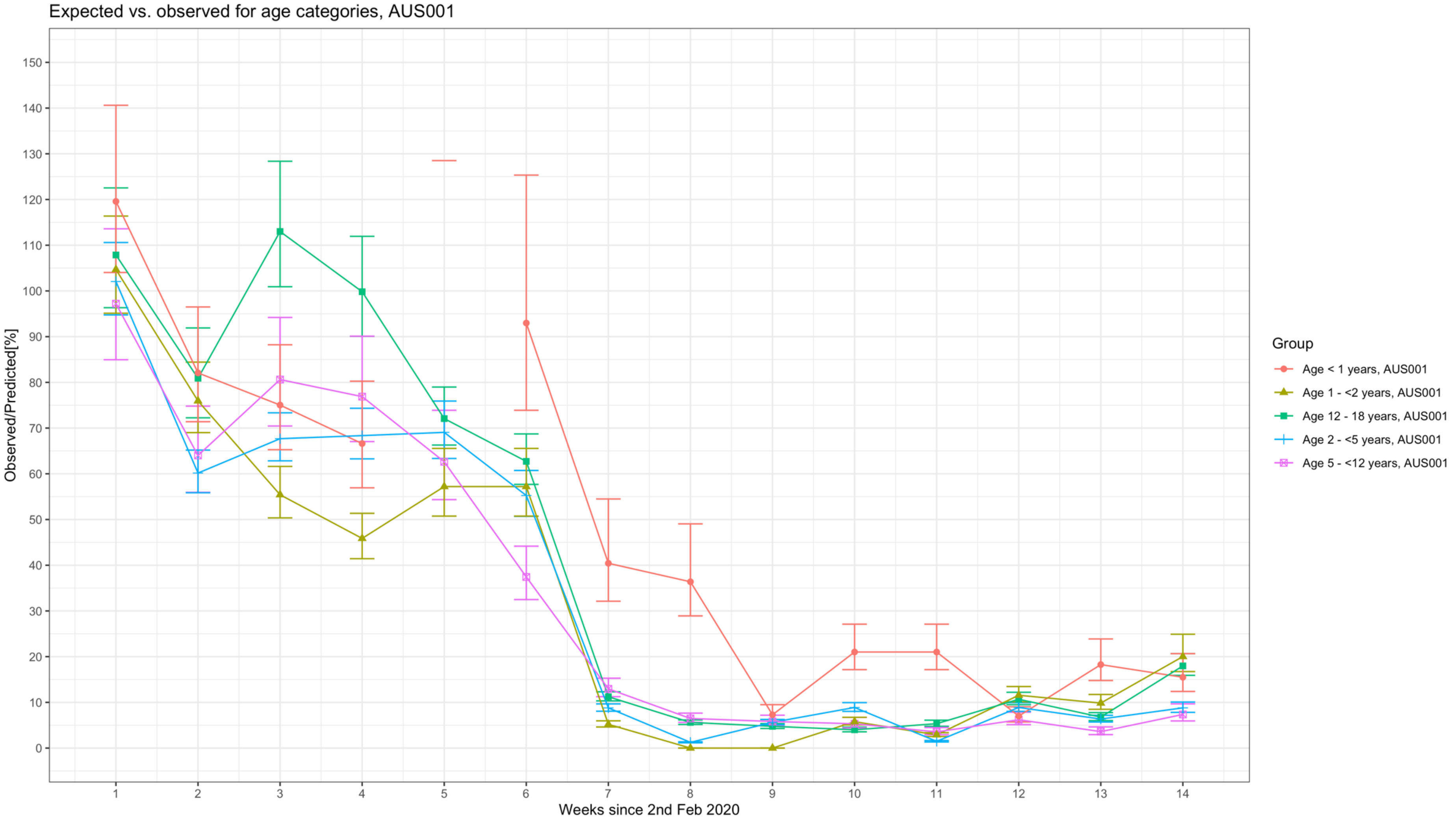

Austria: AUS003

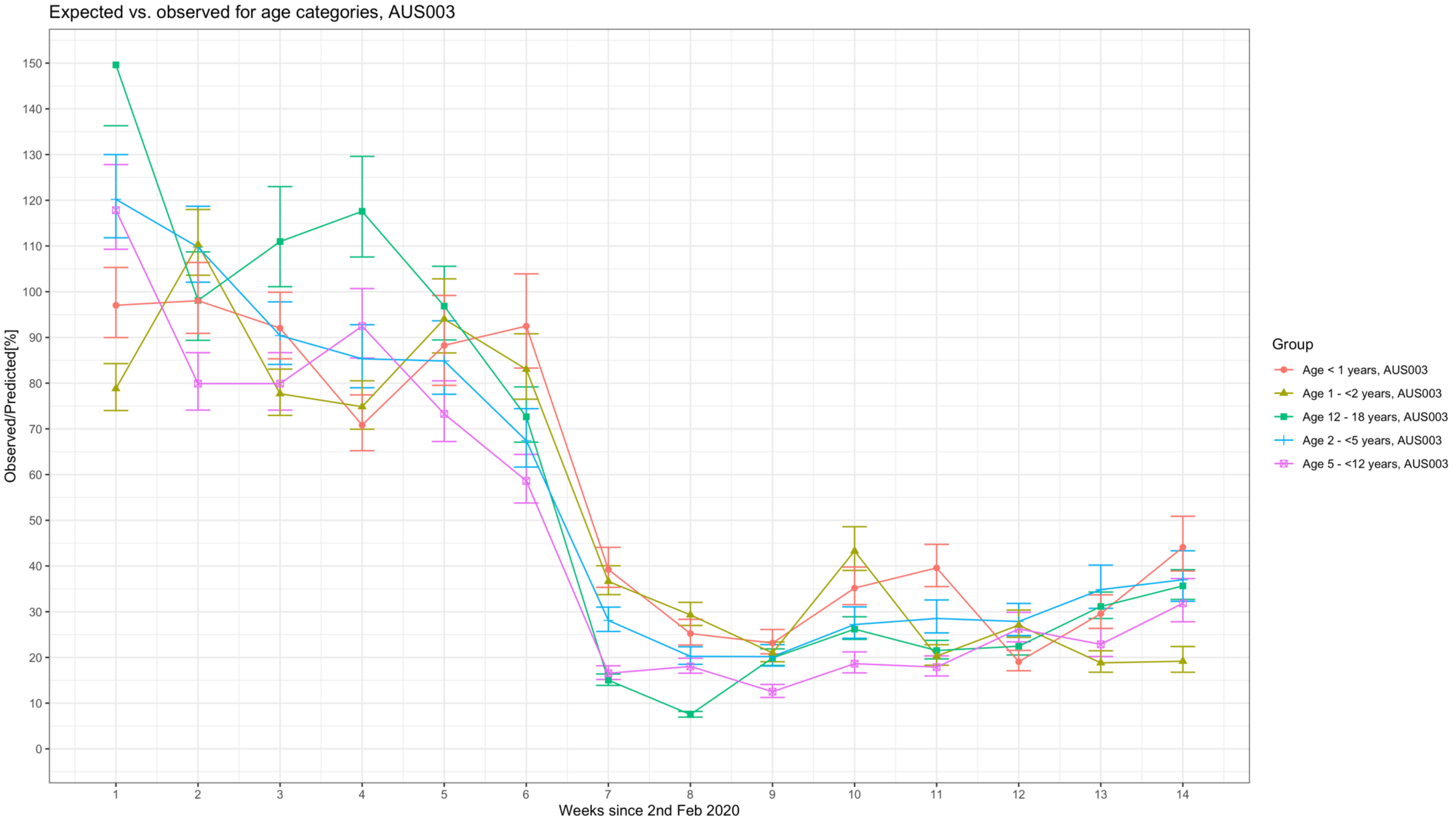

Austria: AUS004

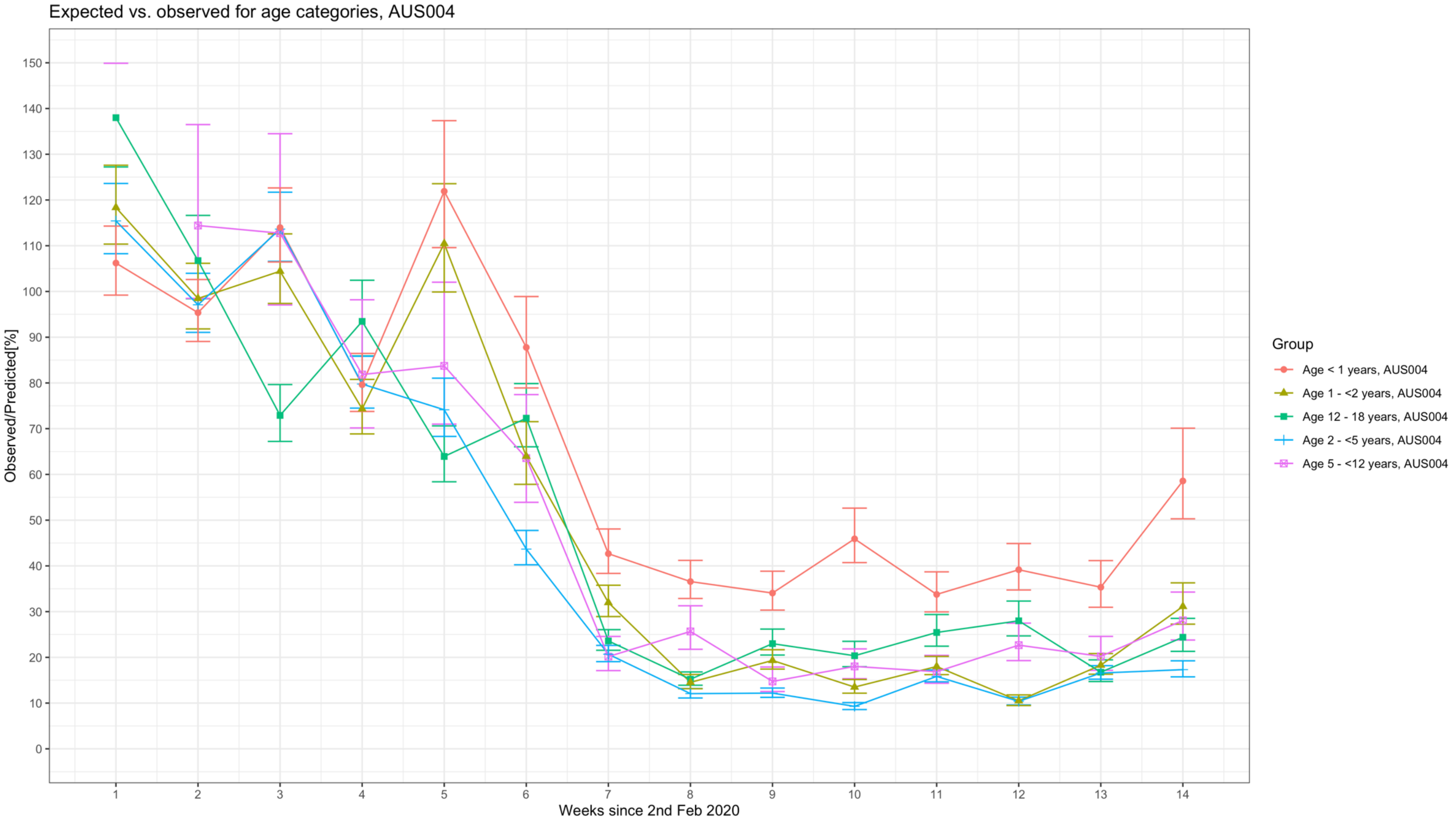

France: FR001

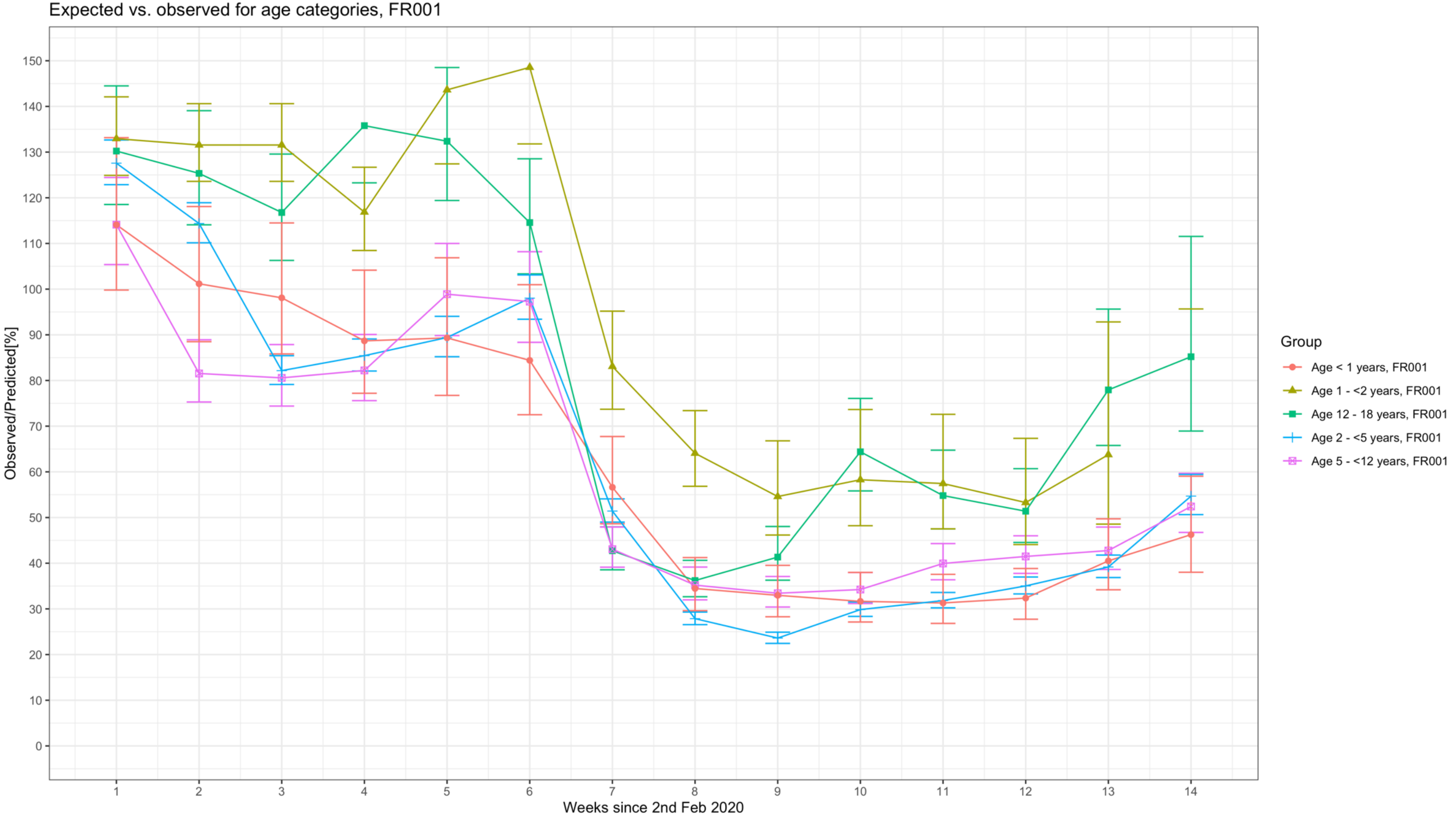

France: FR002

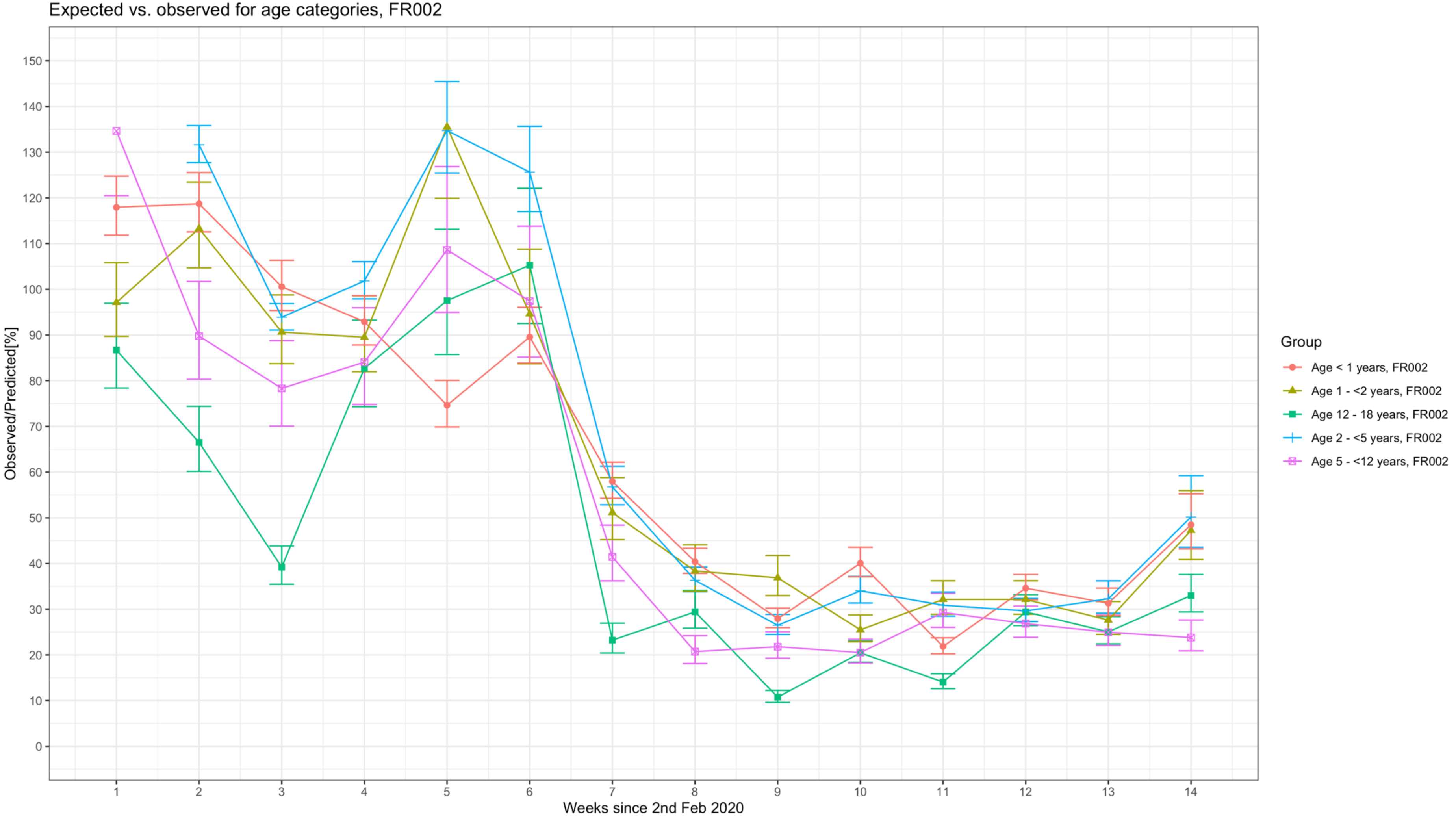

France: FR003

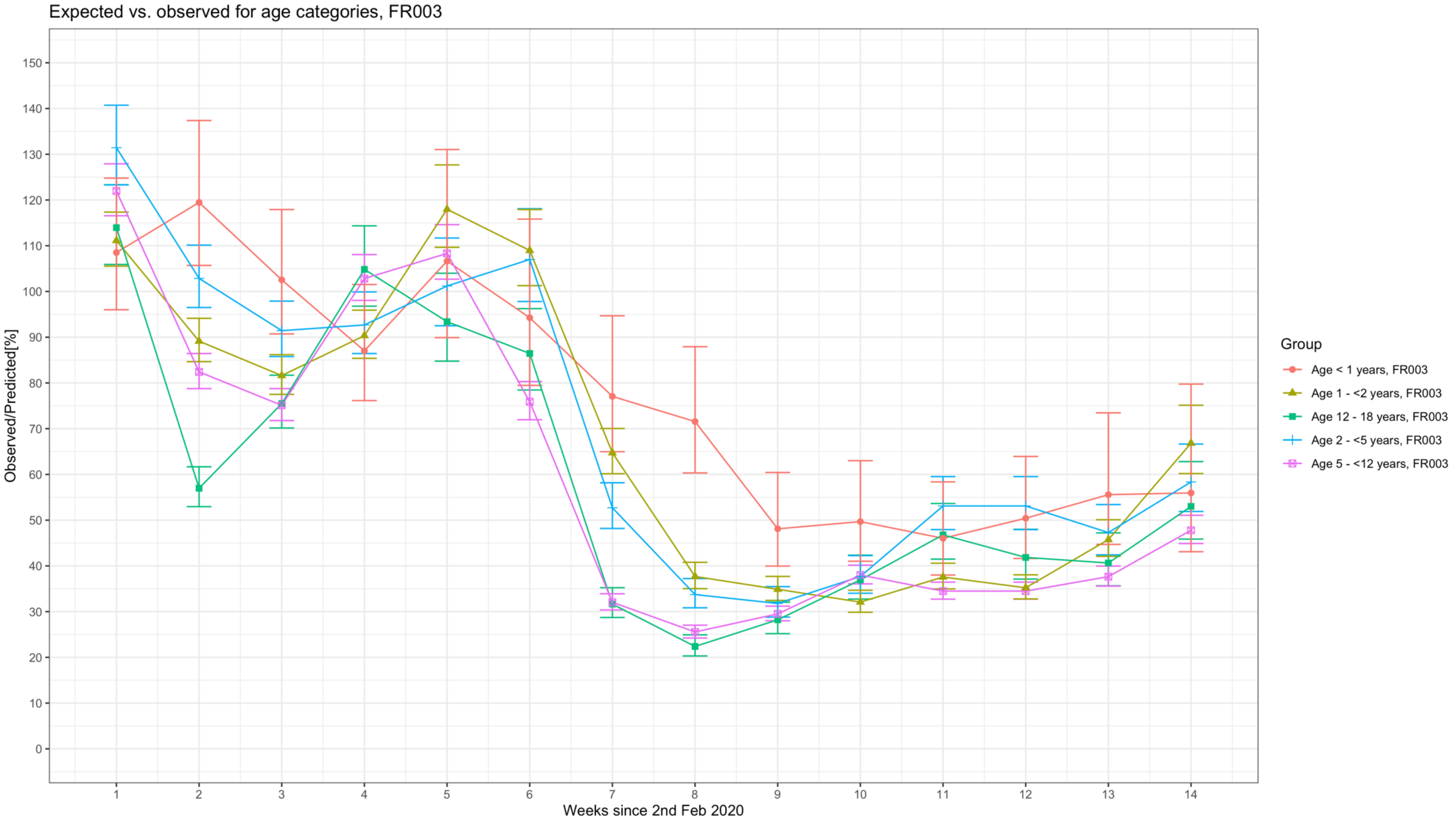

France: FR004

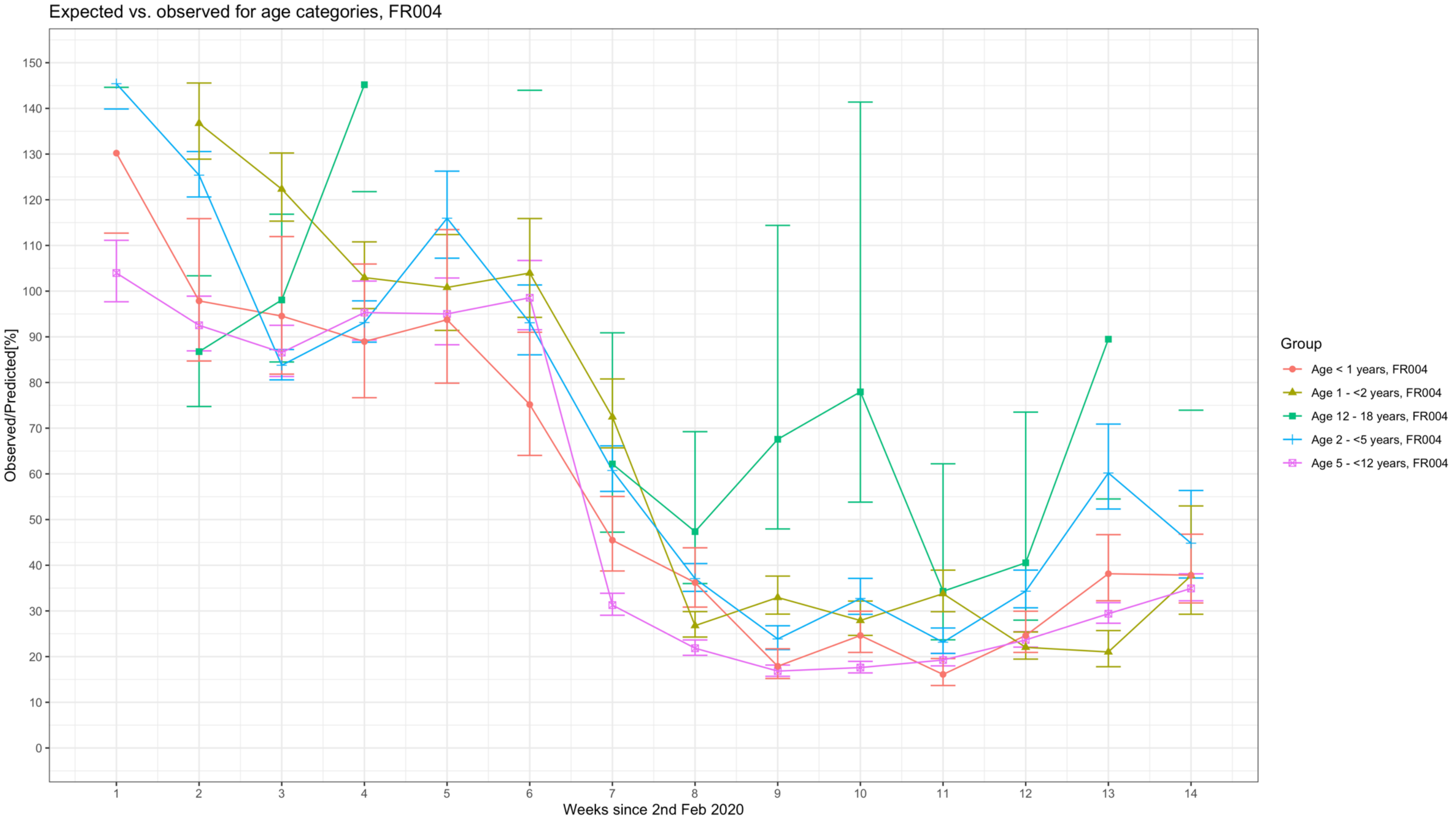

Germany: GER001

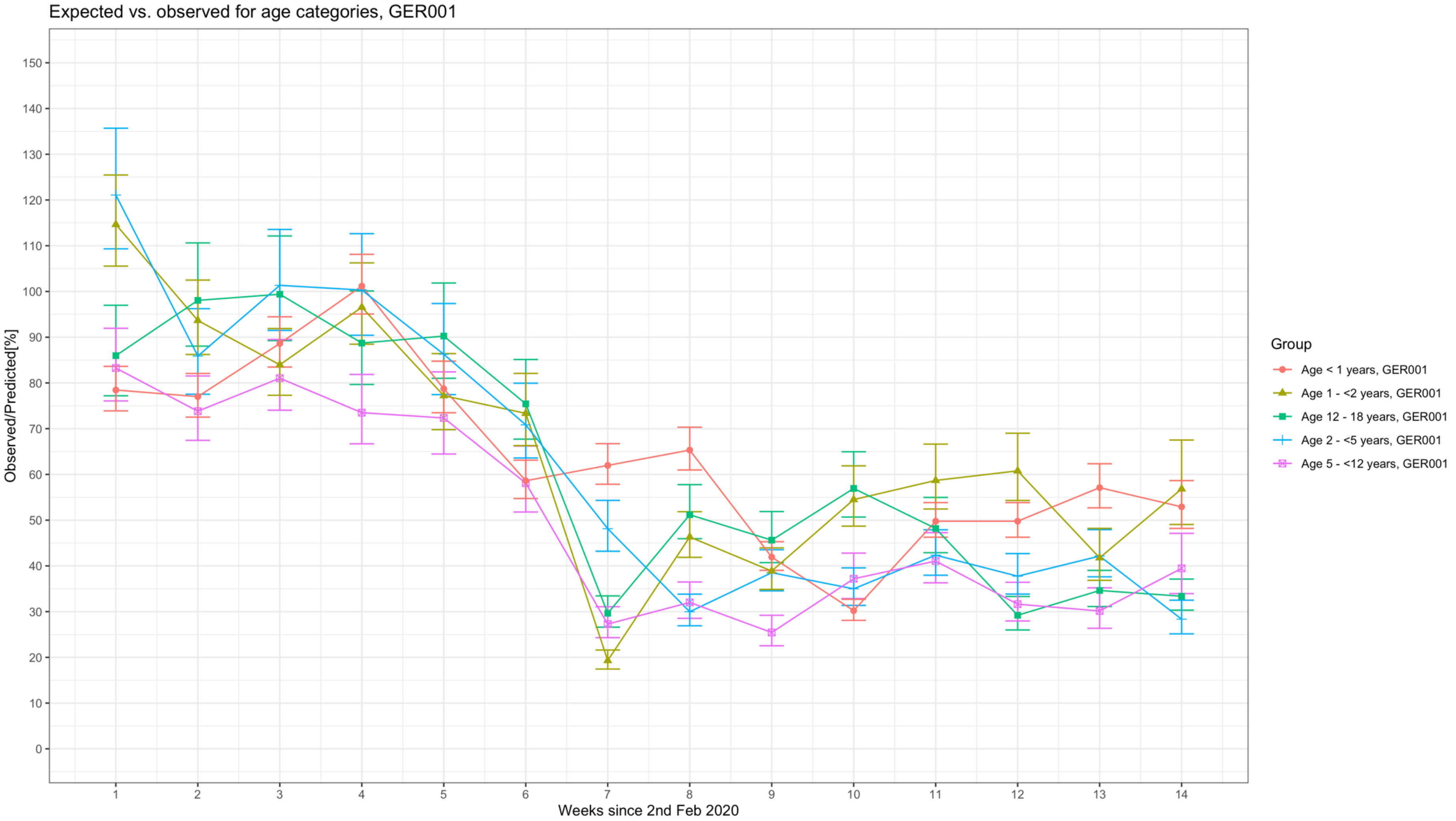

Hungary: HUN001

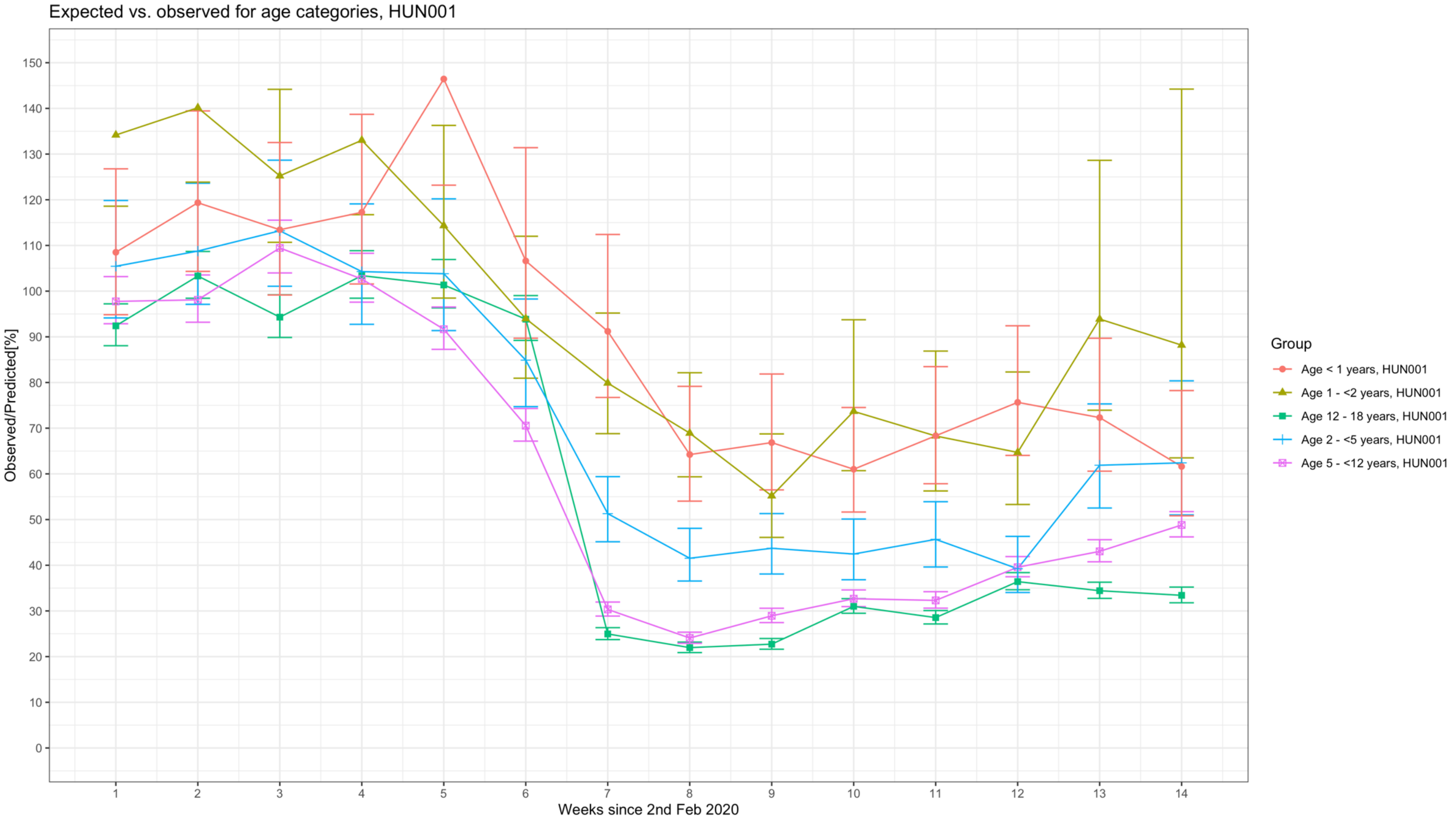

Iceland: ICE001

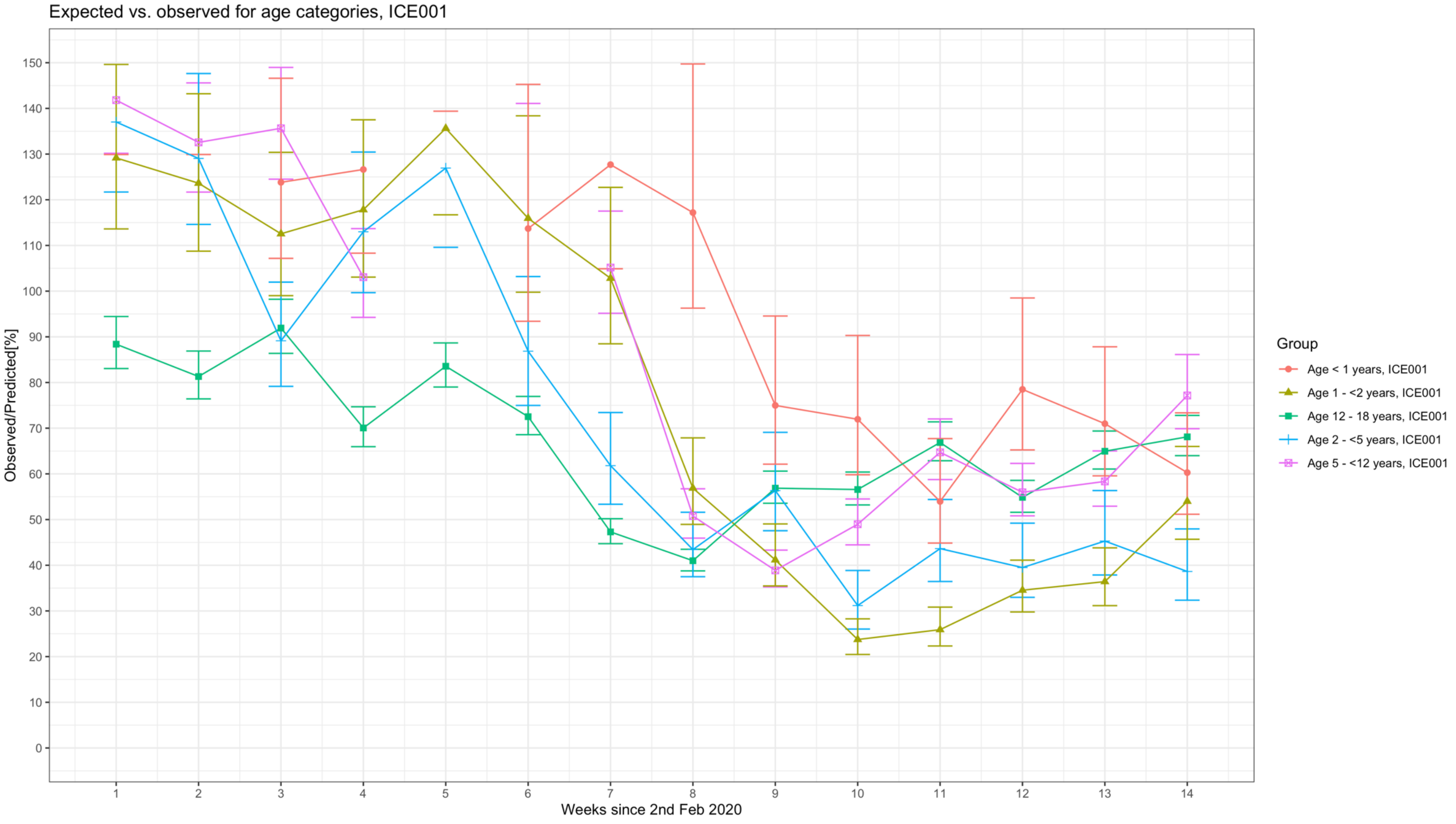

Ireland: IRE001

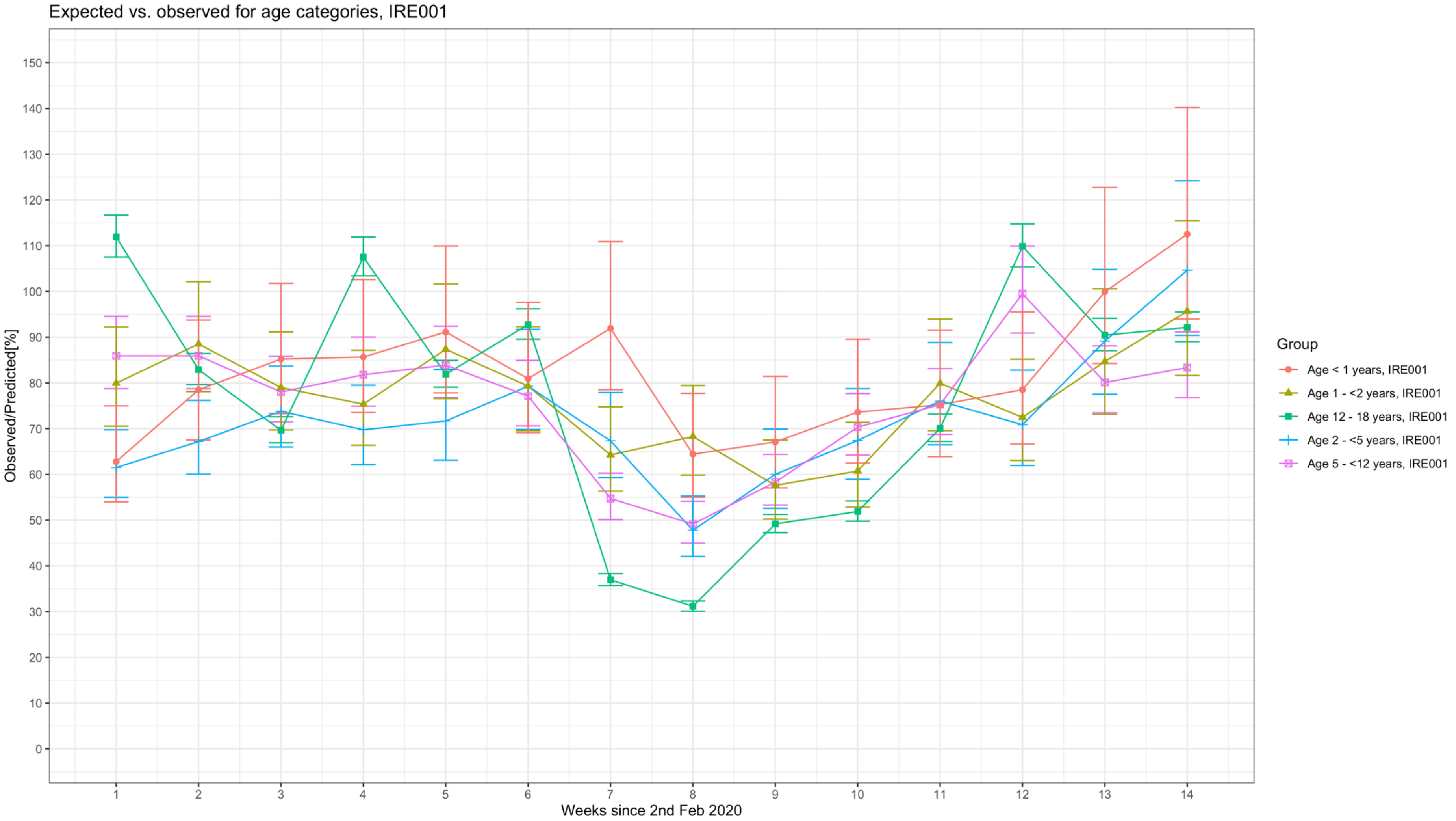

Ireland: IRE002

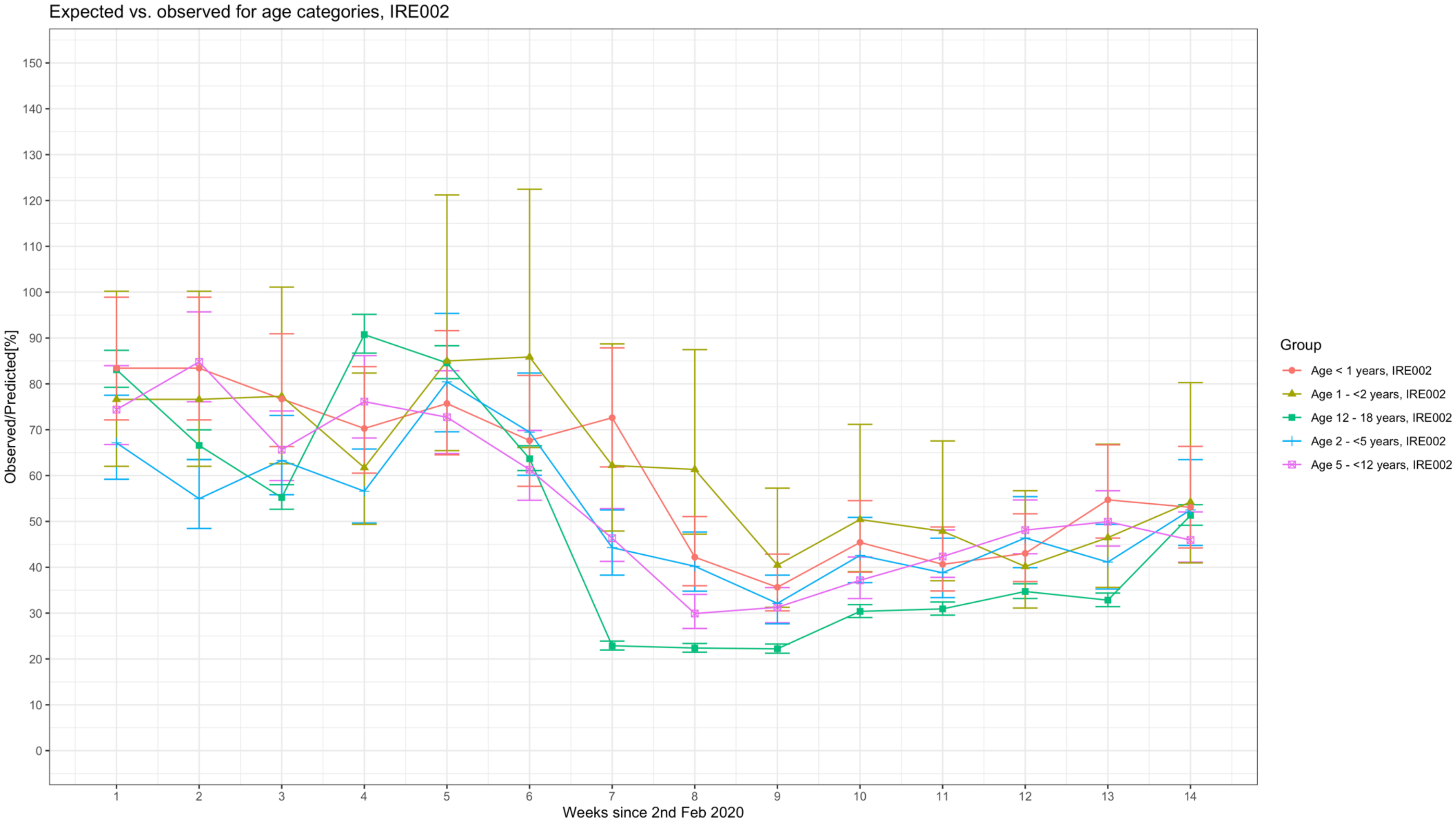

Ireland: IRE003

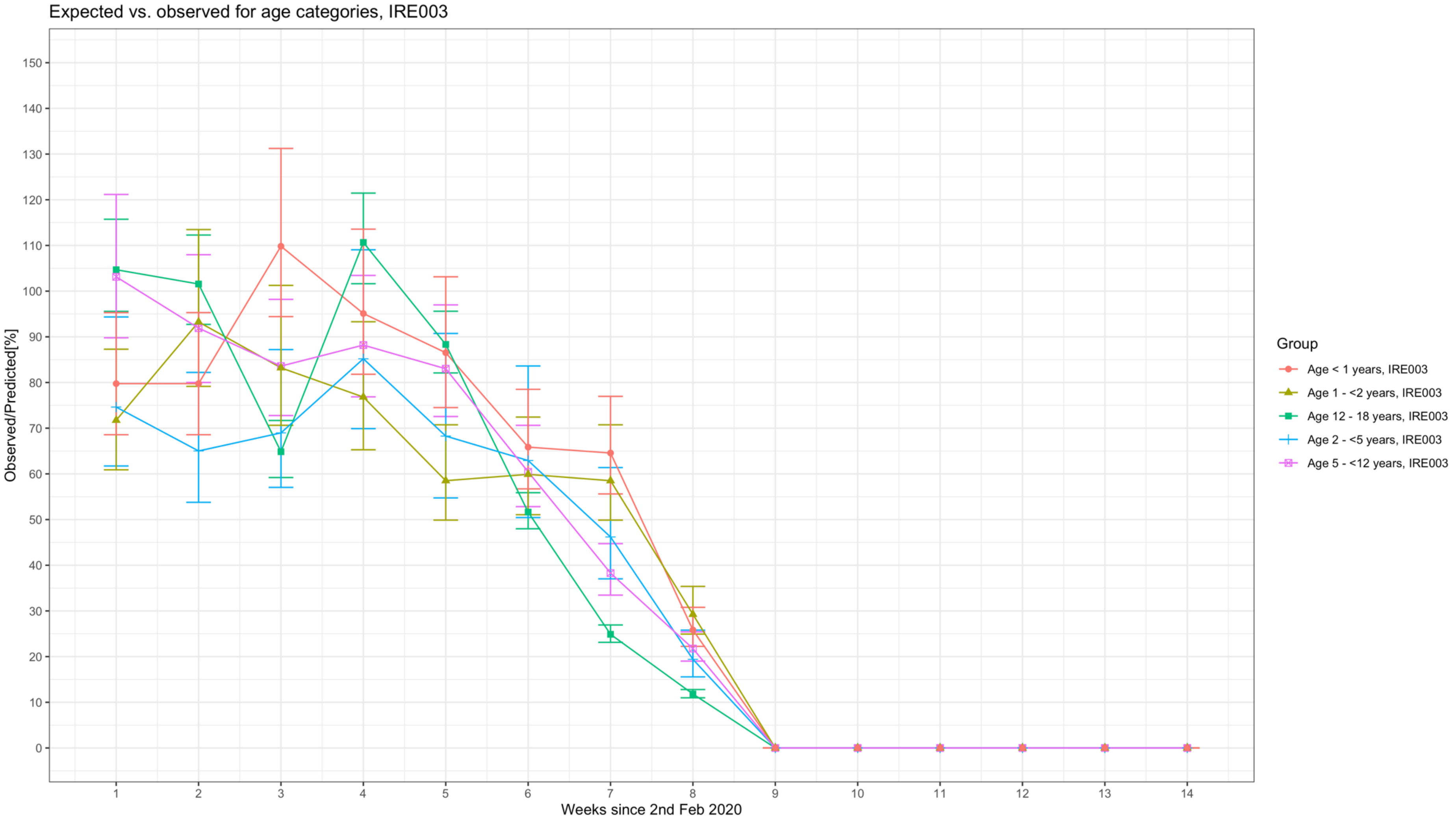

Italy: IT001

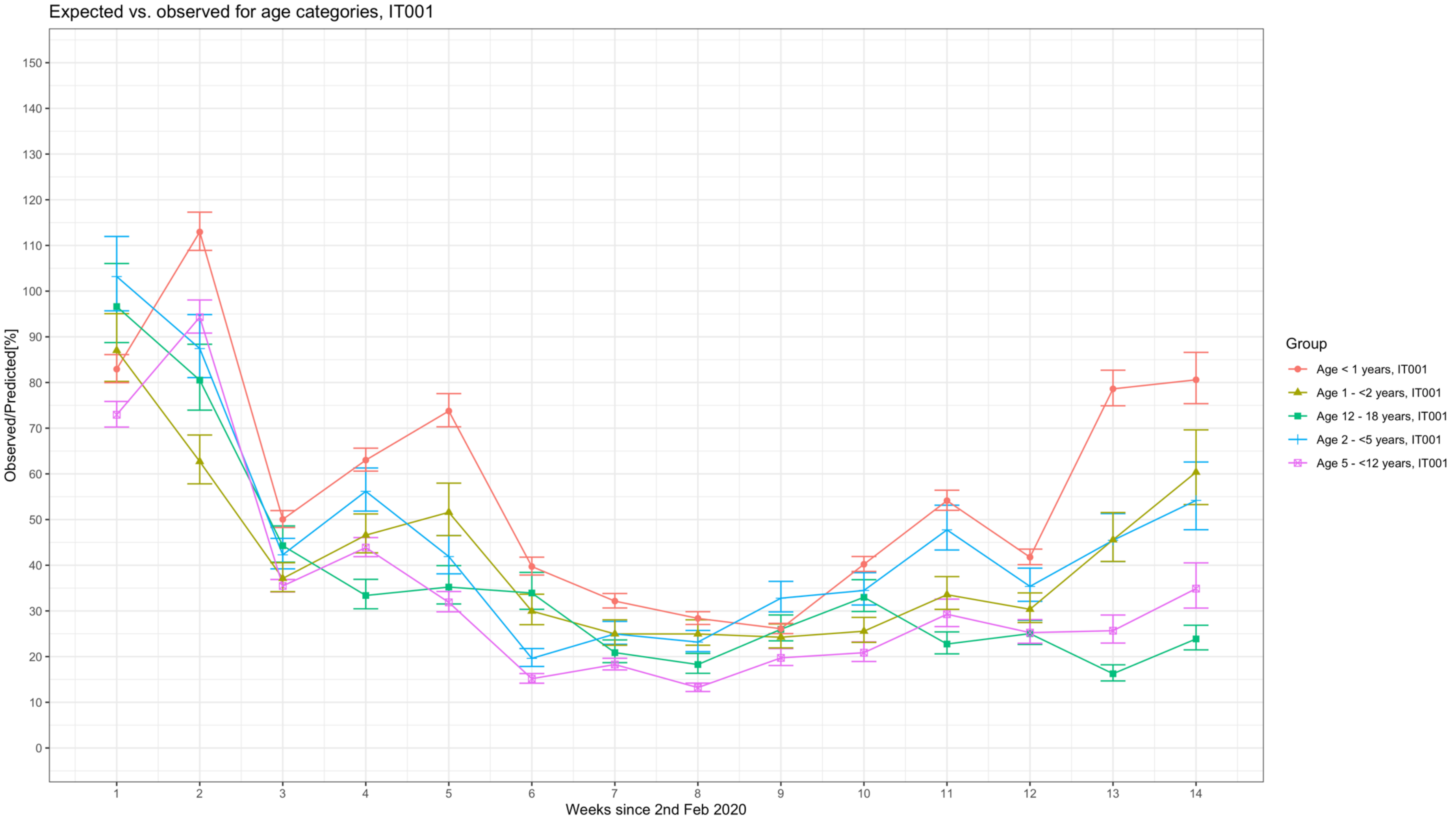

Expected vs. observed for age categories, IT001
Observed/Predicted[%]
Weeks since 2nd Feb 2020
Group
Age < 1 years, IT001
Age 1 - <2 years, IT001
Age 12 - 18 years, IT001
Age 2 - <5 years, IT001
Age 5 - <12 years, IT001

Italy: IT002

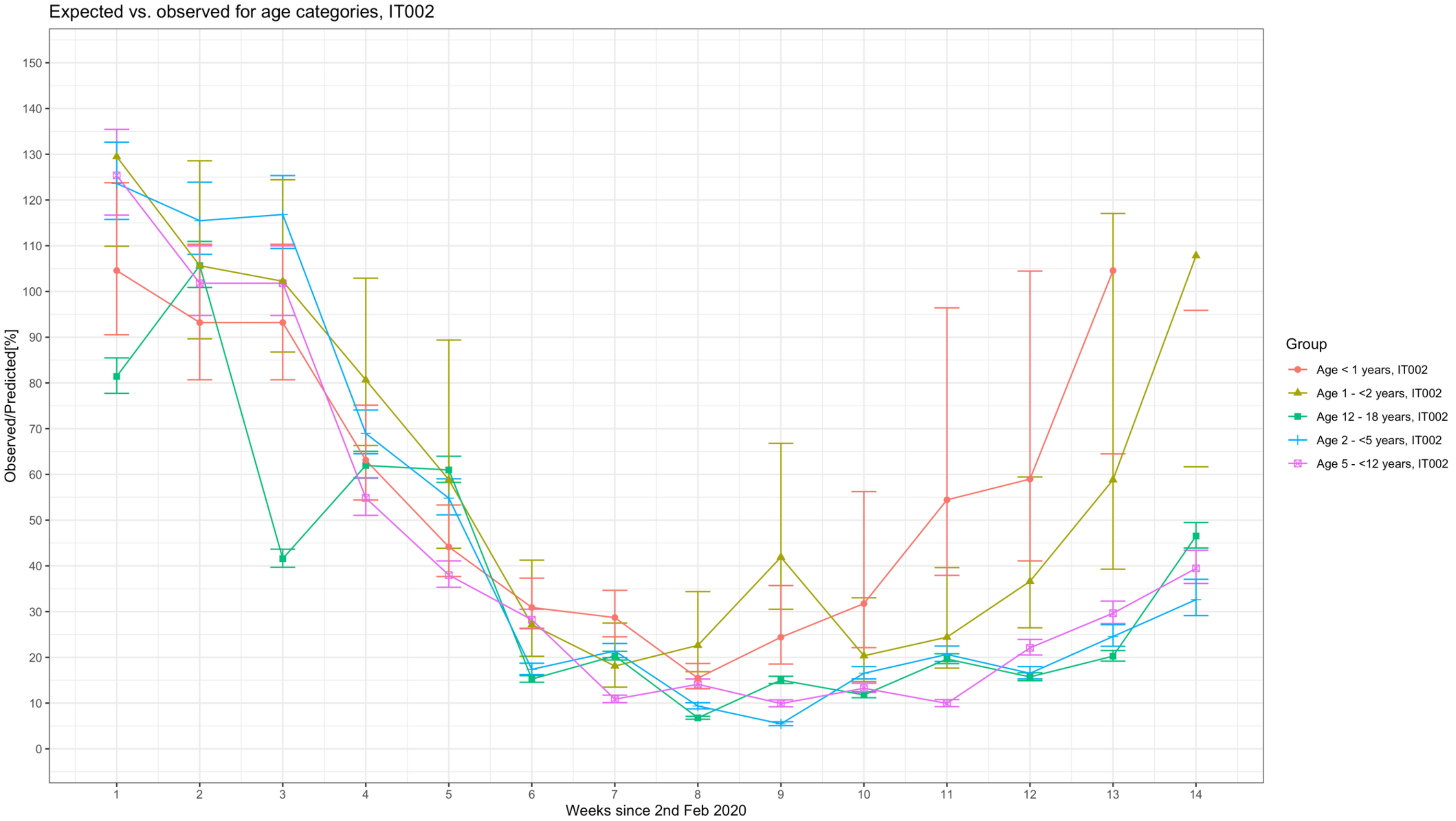
Expected vs. observed for age categories, IT002
Observed/Predicted[%]
150
140
130
120
110
100
90
80
70
60
50
40
30
20
10
0
1
2
3
4
5
6
7
8
9
10
11
12
13
14
Weeks since 2nd Feb 2020
Group
Age < 1 years, IT002
Age 1 - <2 years, IT002
Age 12 - 18 years, IT002
Age 2 - <5 years, IT002
Age 5 - <12 years, IT002

Latvia: LAT001

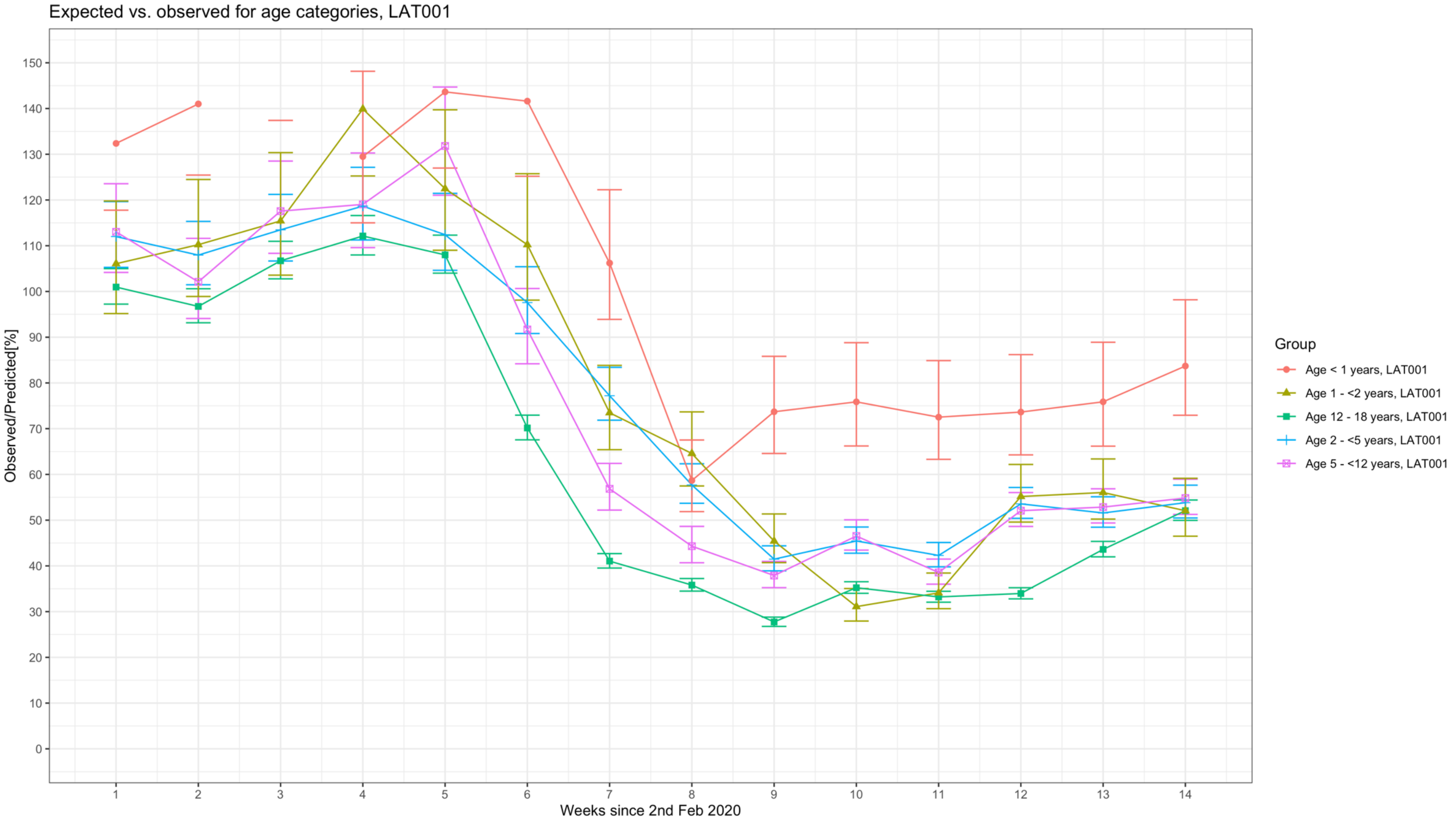

Lithuania: LIT001

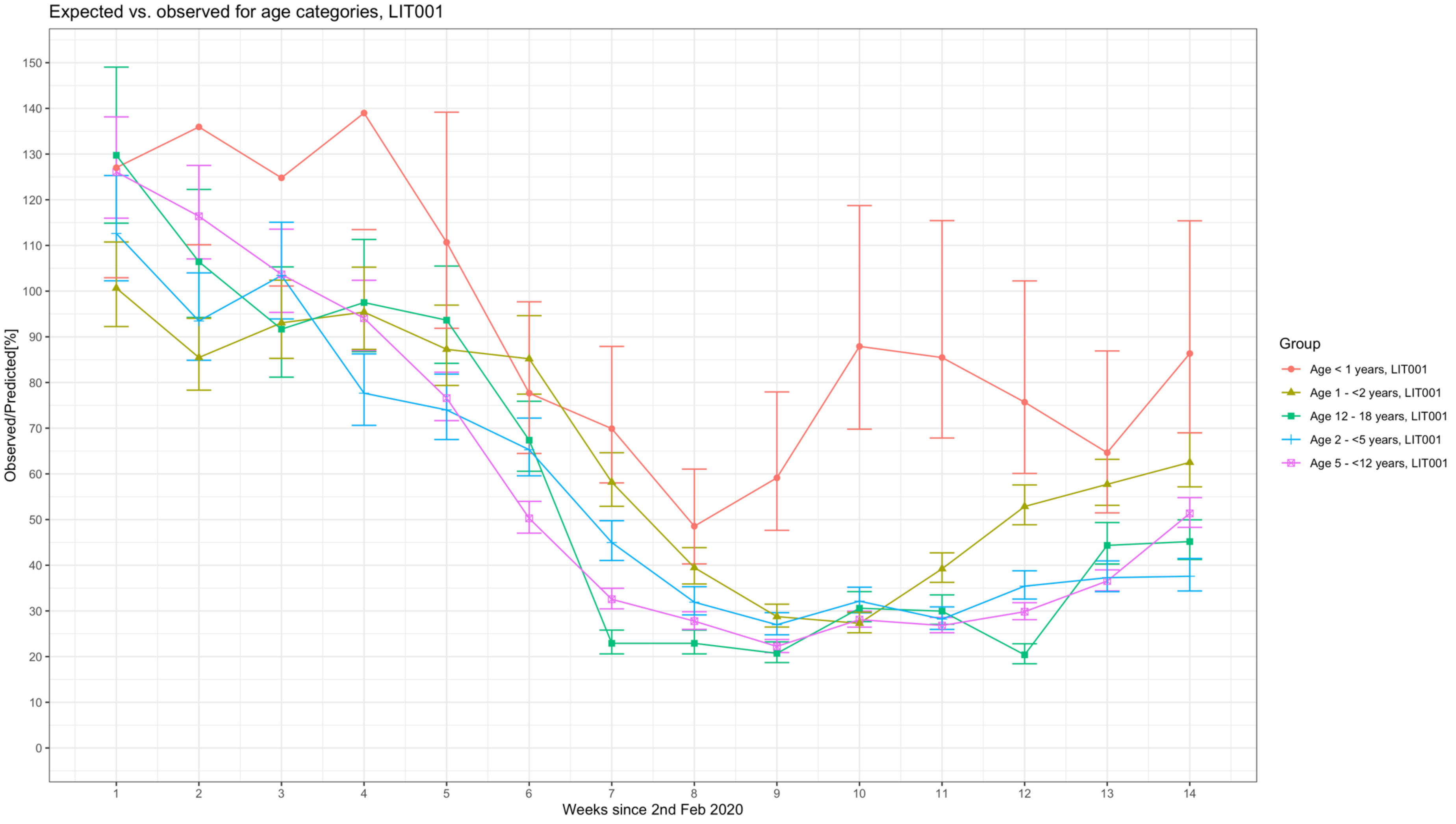

Malta: MAL001

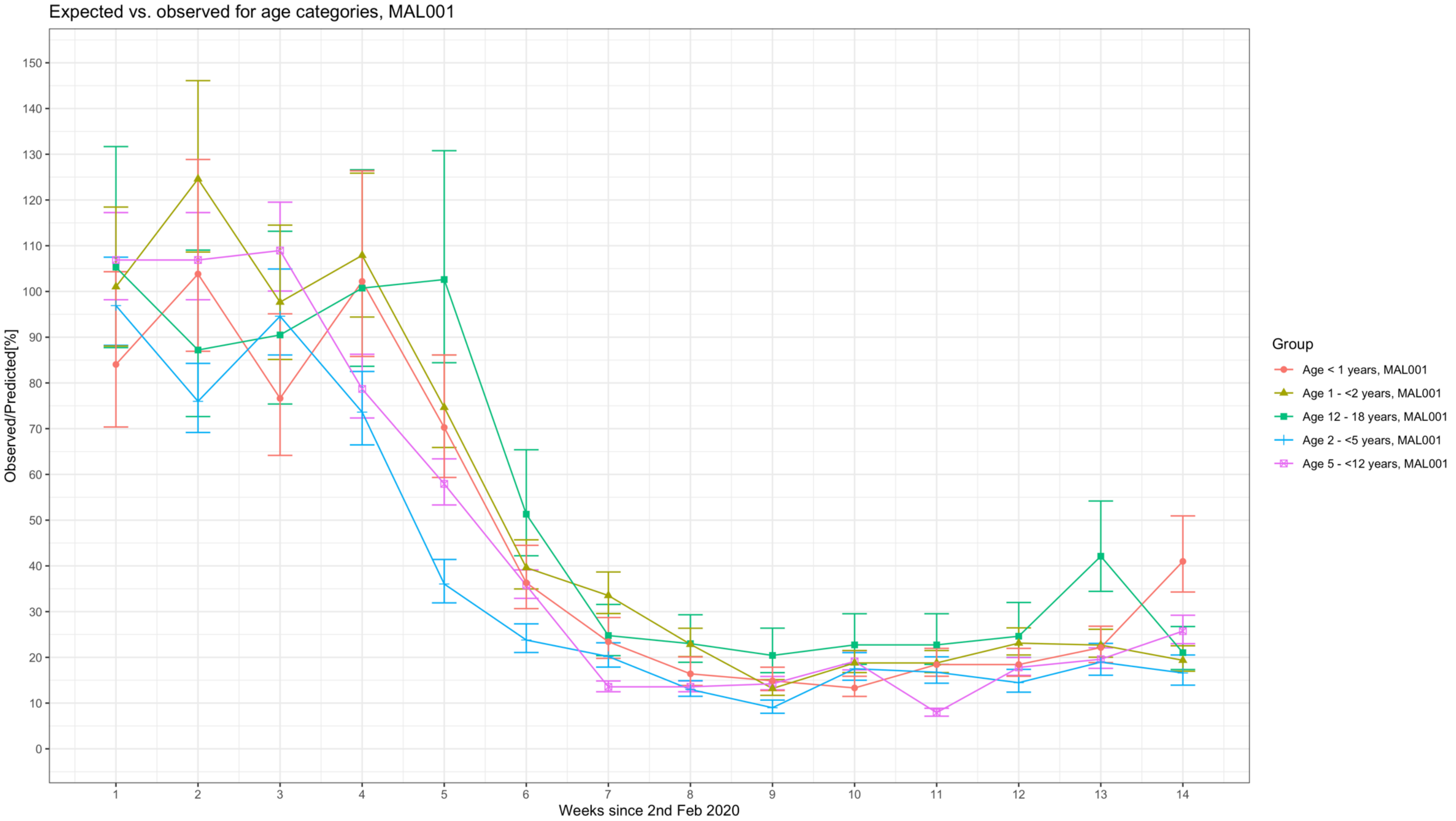

The Netherlands: NL001

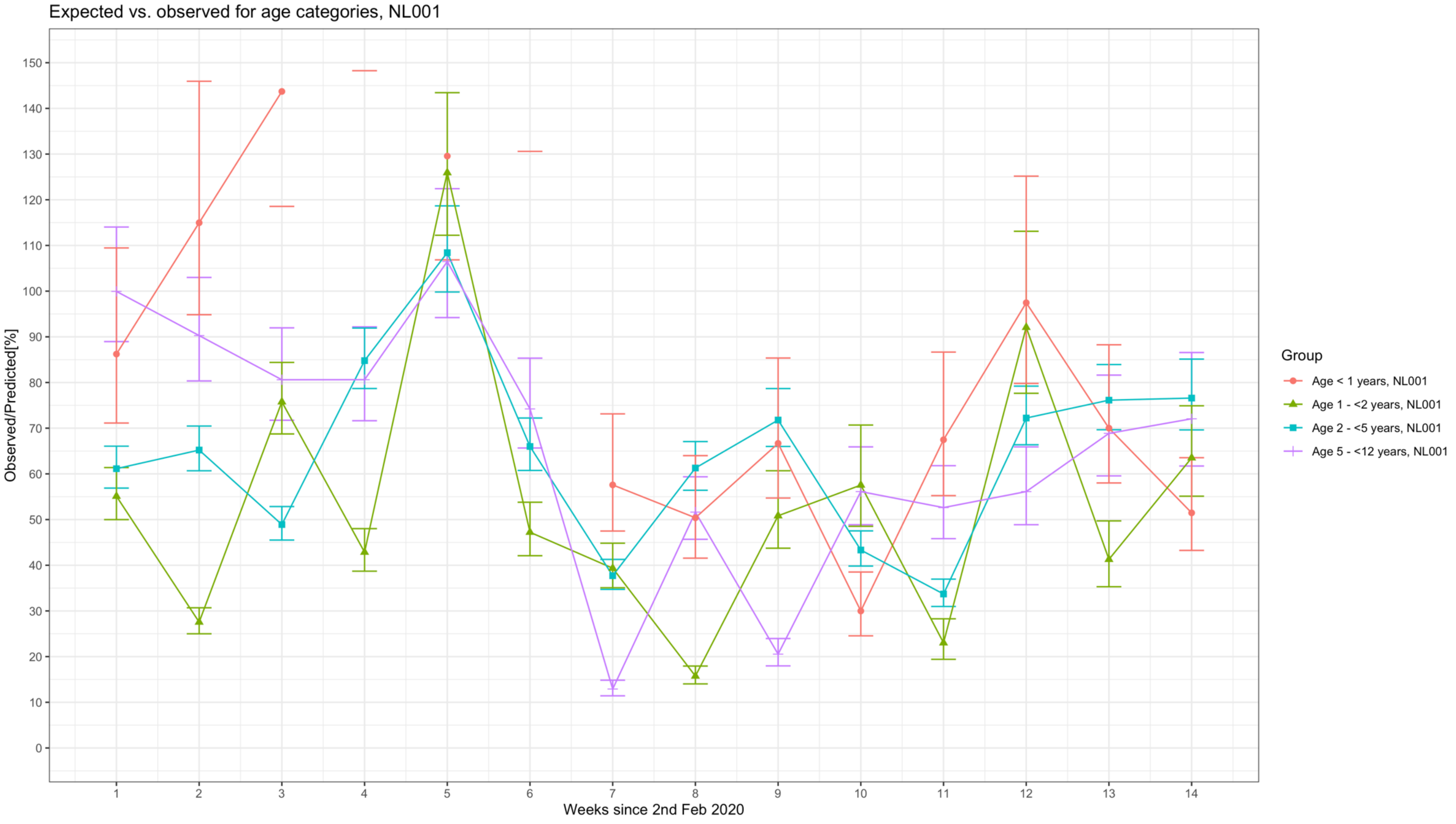

Portugal: POR001

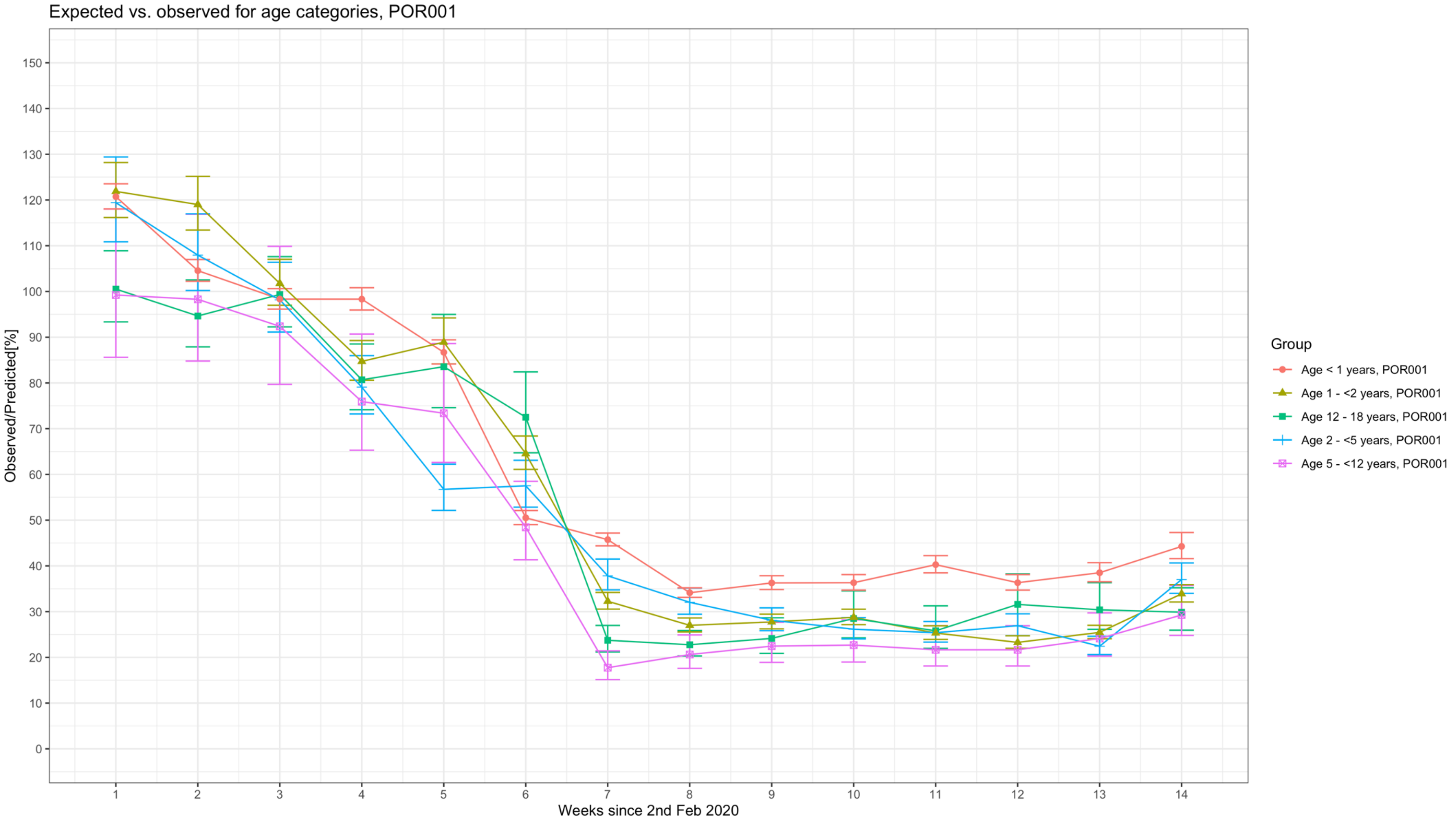

Portugal: POR003

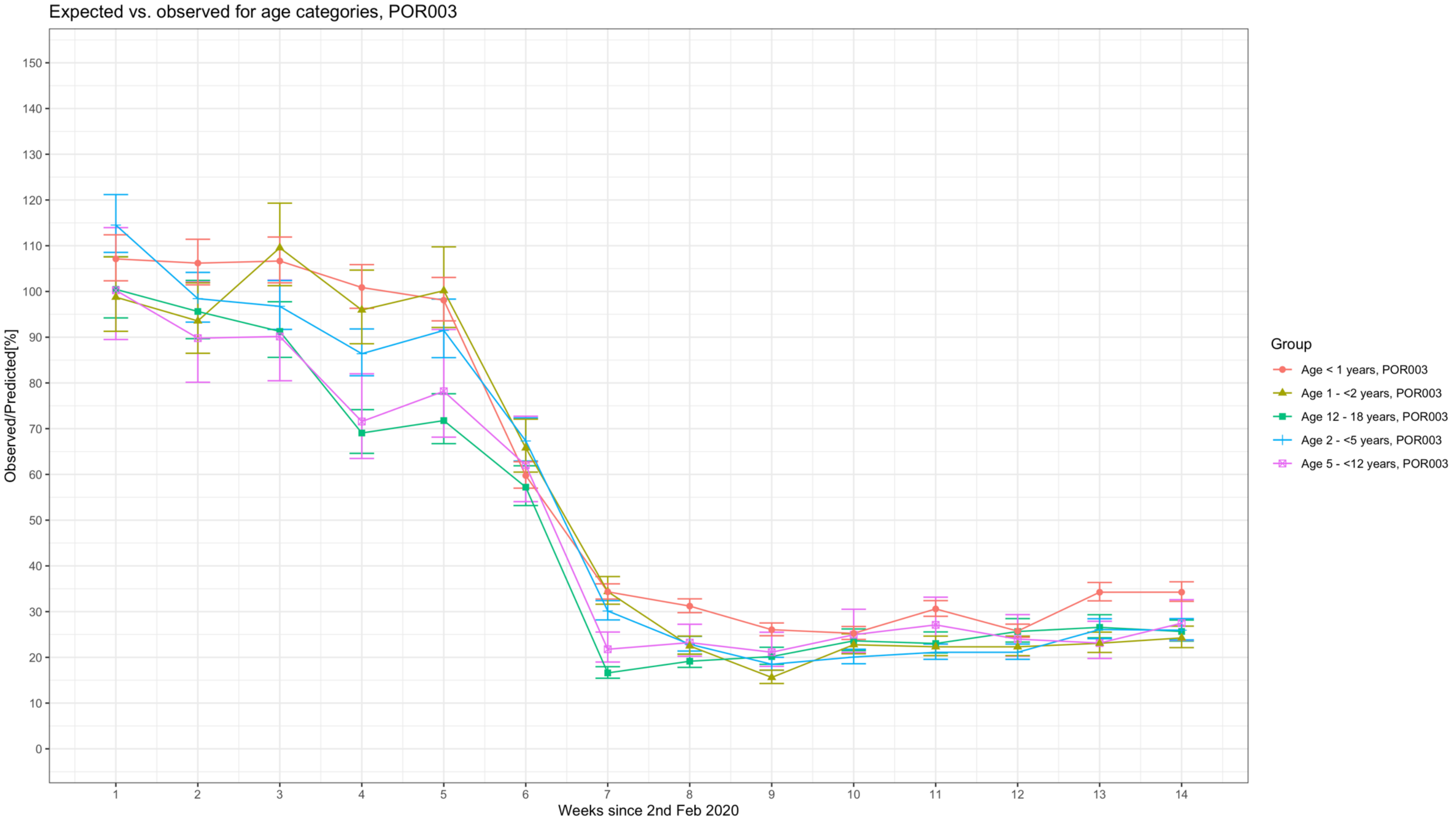

Portugal: POR004

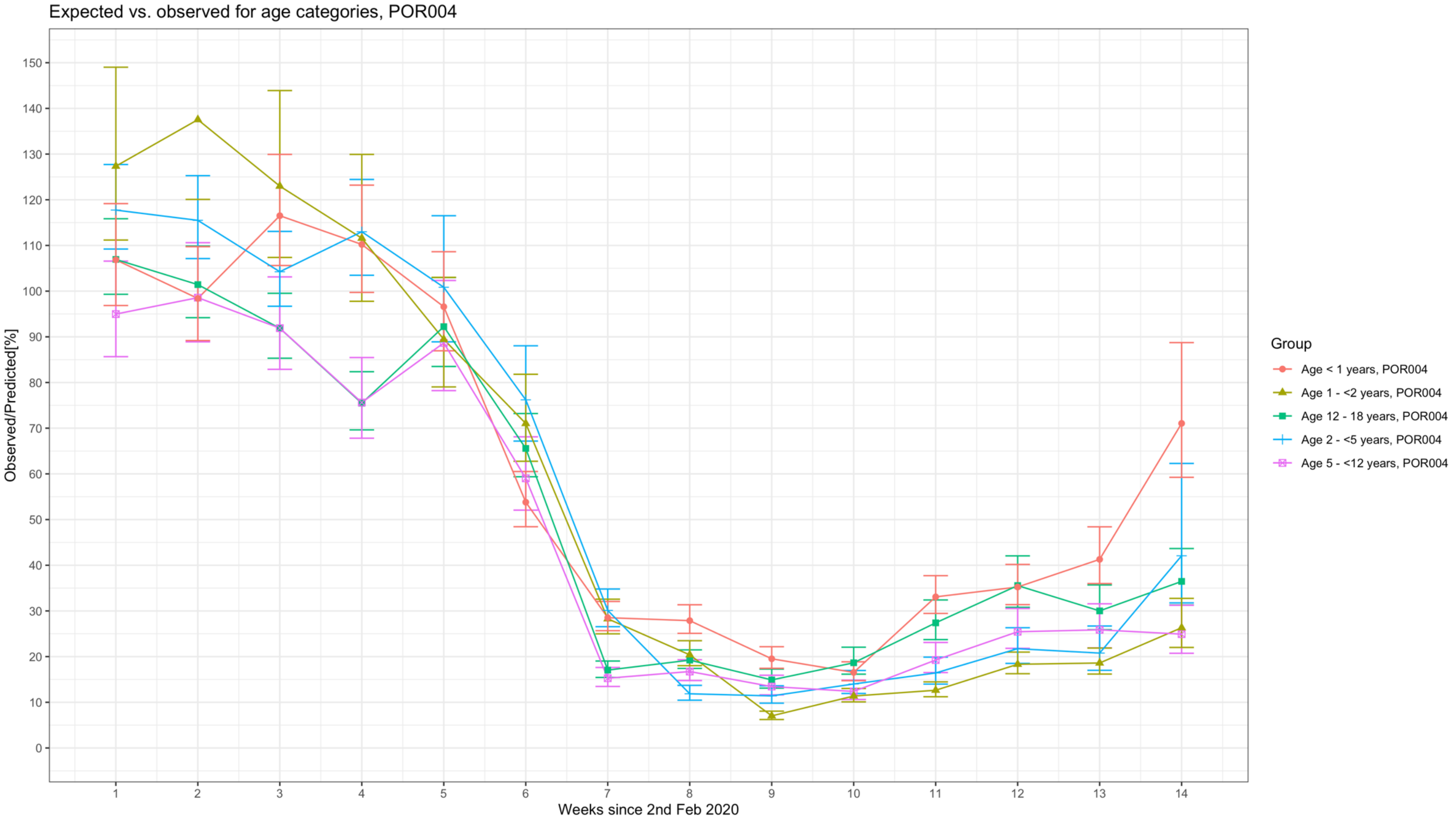

Portugal: POR005

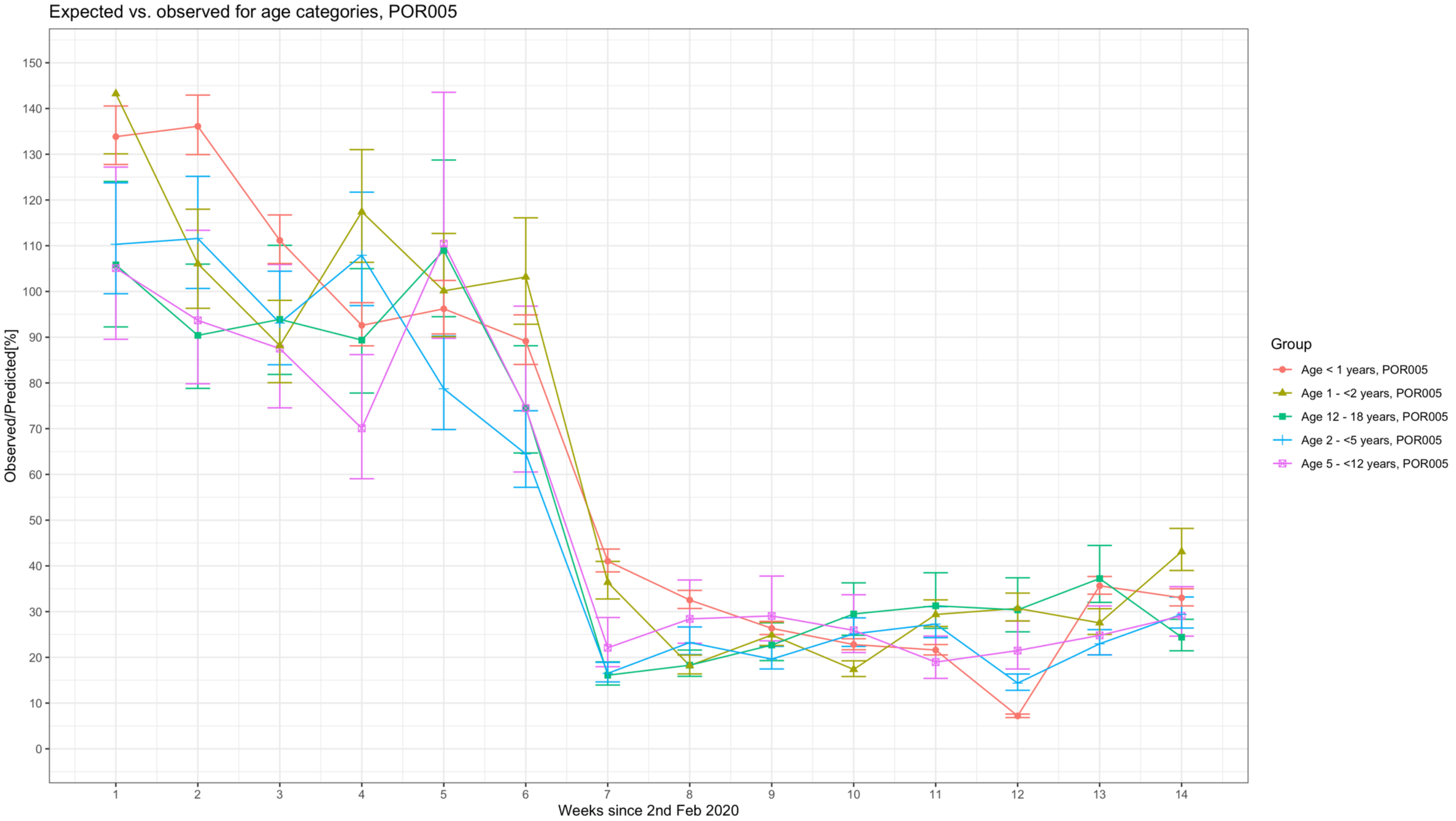

Slovenia: SLO001

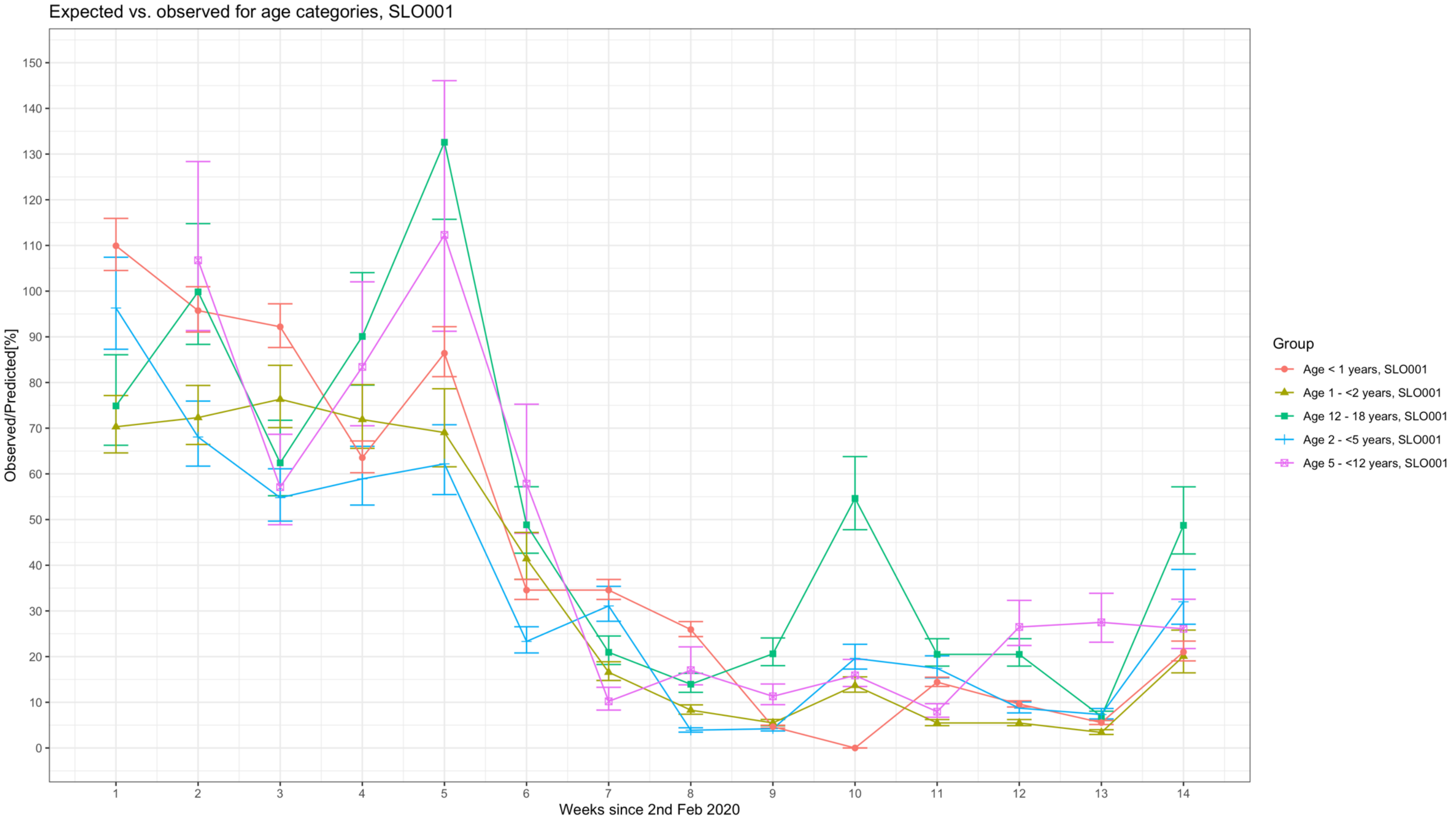

Spain: SP001

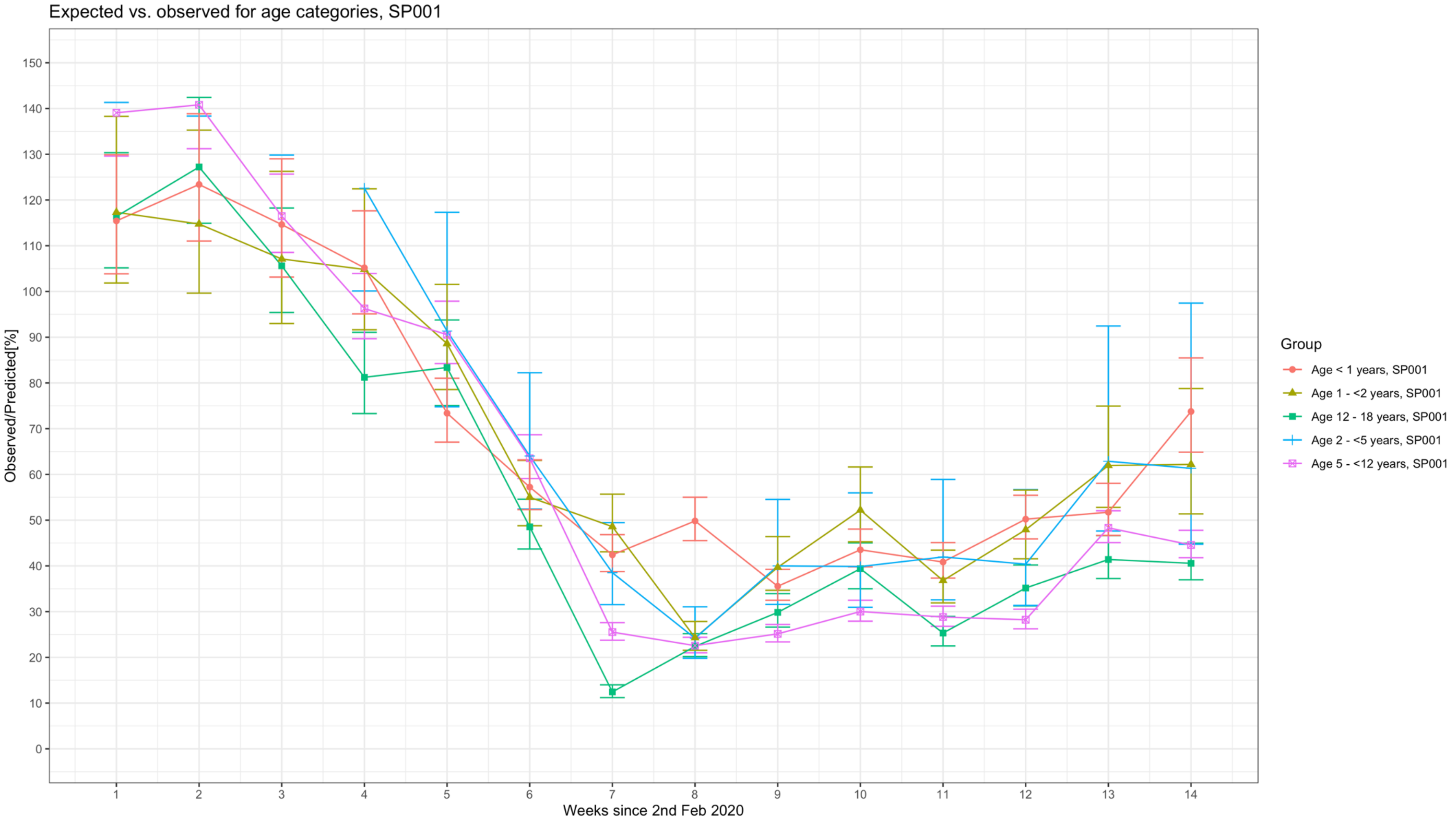

Expected vs. observed for age categories, SP001
Observed/Predicted[%]
Weeks since 2nd Feb 2020
Group
Age < 1 years, SP001
Age 1 - <2 years, SP001
Age 12 - 18 years, SP001
Age 2 - <5 years, SP001
Age 5 - <12 years, SP001

Spain: SP002

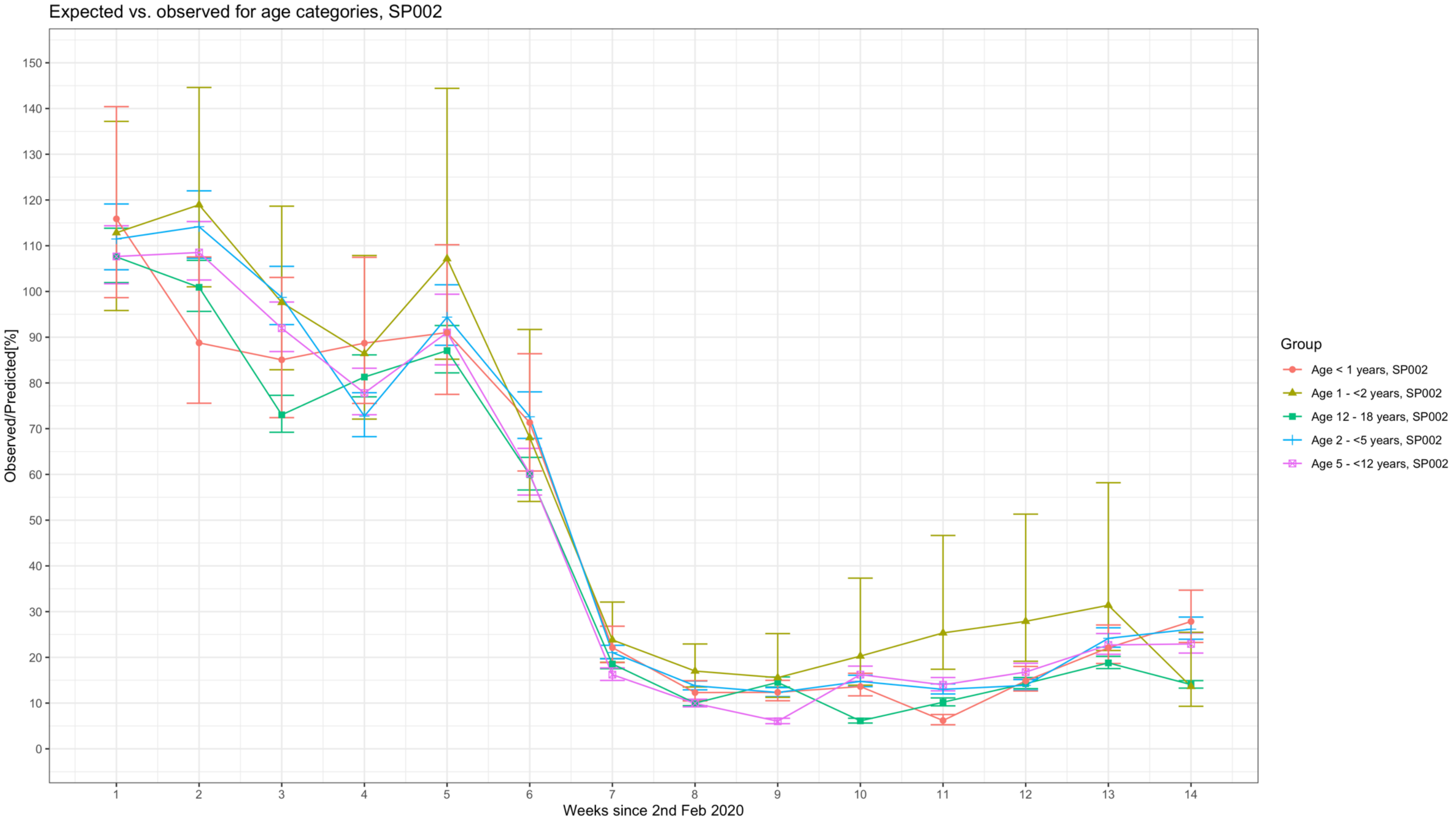

Expected vs. observed for age categories, SP002
Observed/Predicted[%]
150
140
130
120
110
100
90
80
70
60
50
40
30
20
10
0
1
2
3
4
5
6
7
8
9
10
11
12
13
14
Weeks since 2nd Feb 2020
Group
Age < 1 years, SP002
Age 1 - <2 years, SP002
Age 12 - 18 years, SP002
Age 2 - <5 years, SP002
Age 5 - <12 years, SP002

Sweden: SWE001

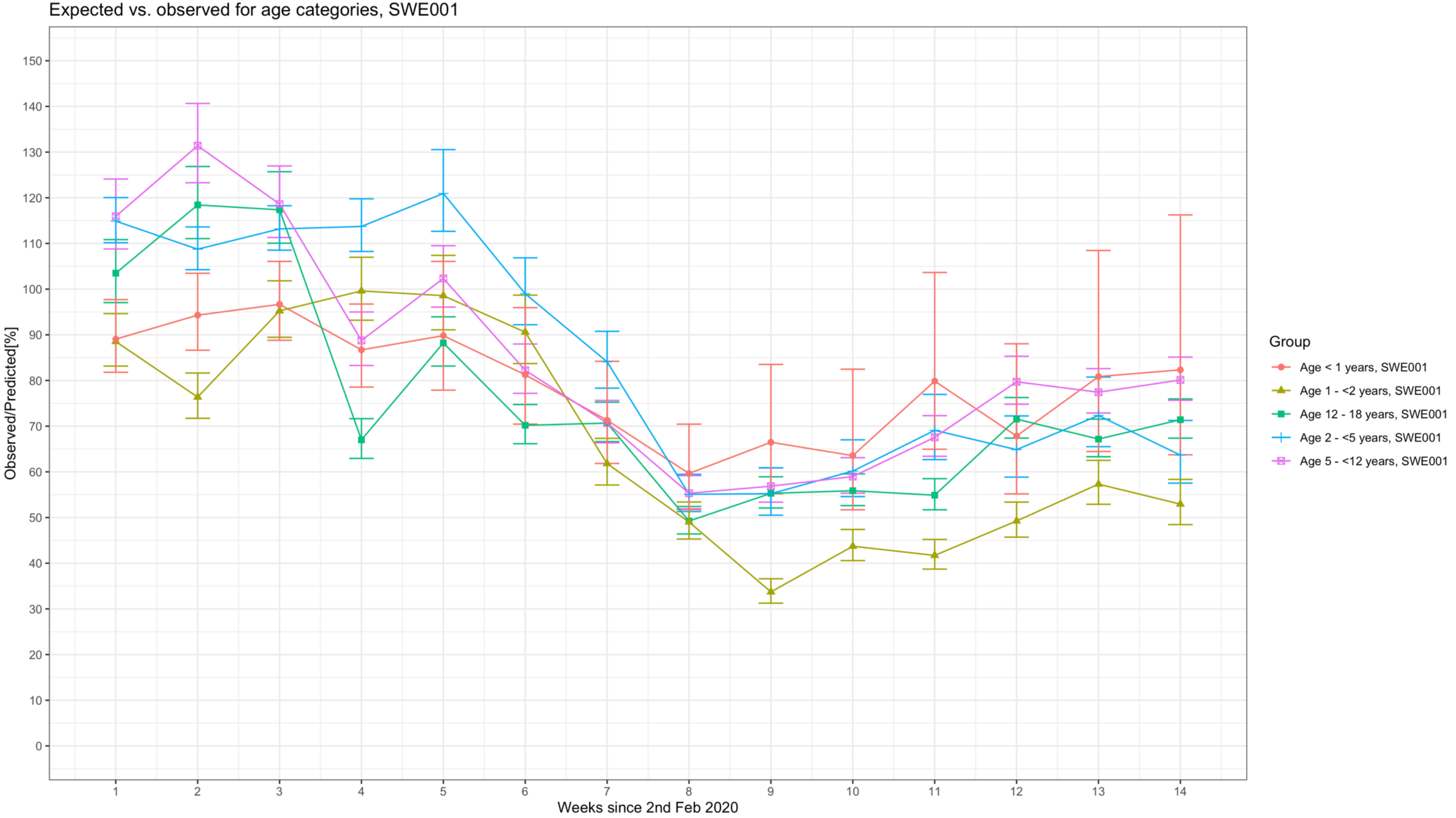

Sweden: SWE002

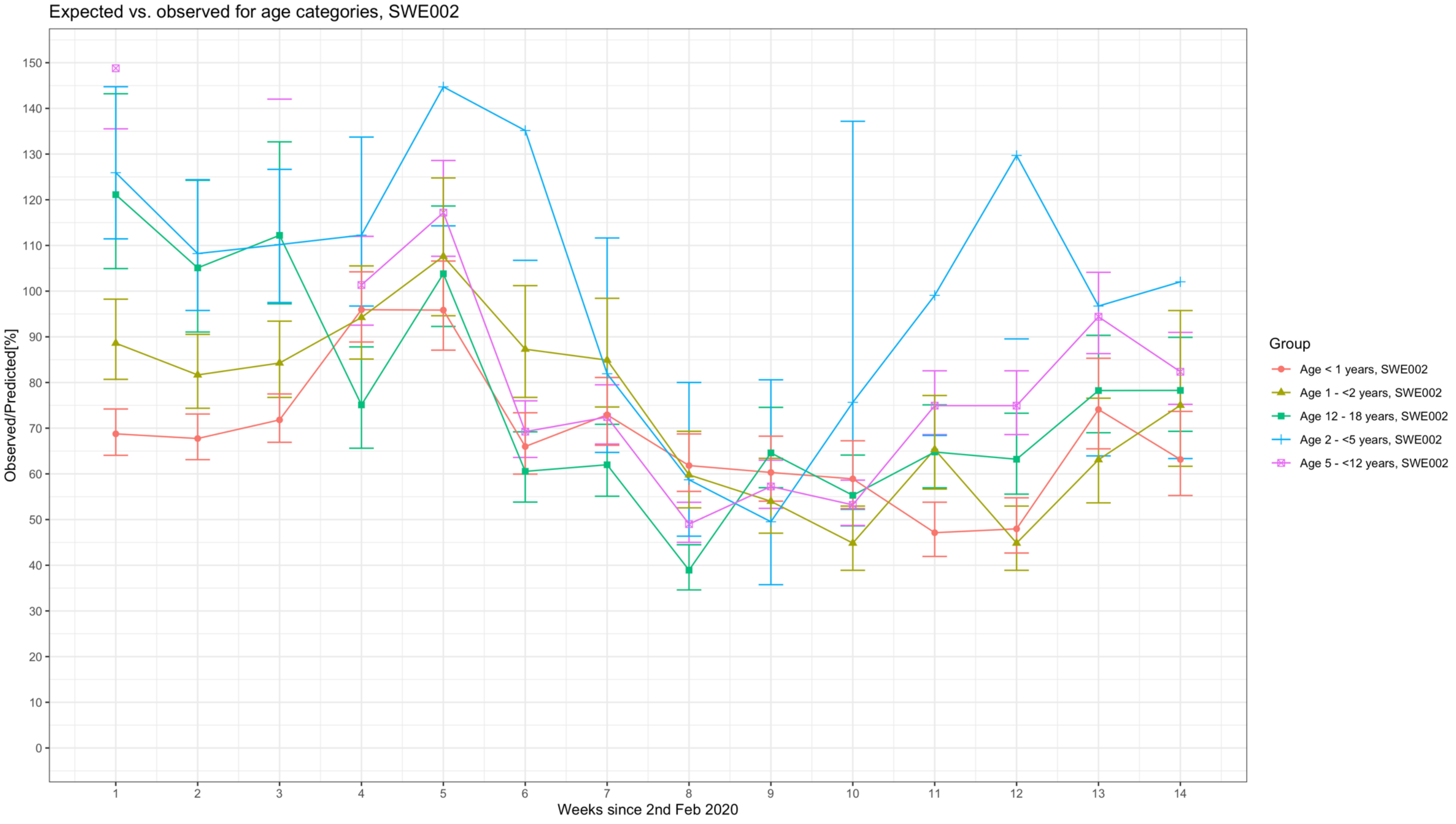

Turkey: TUR001

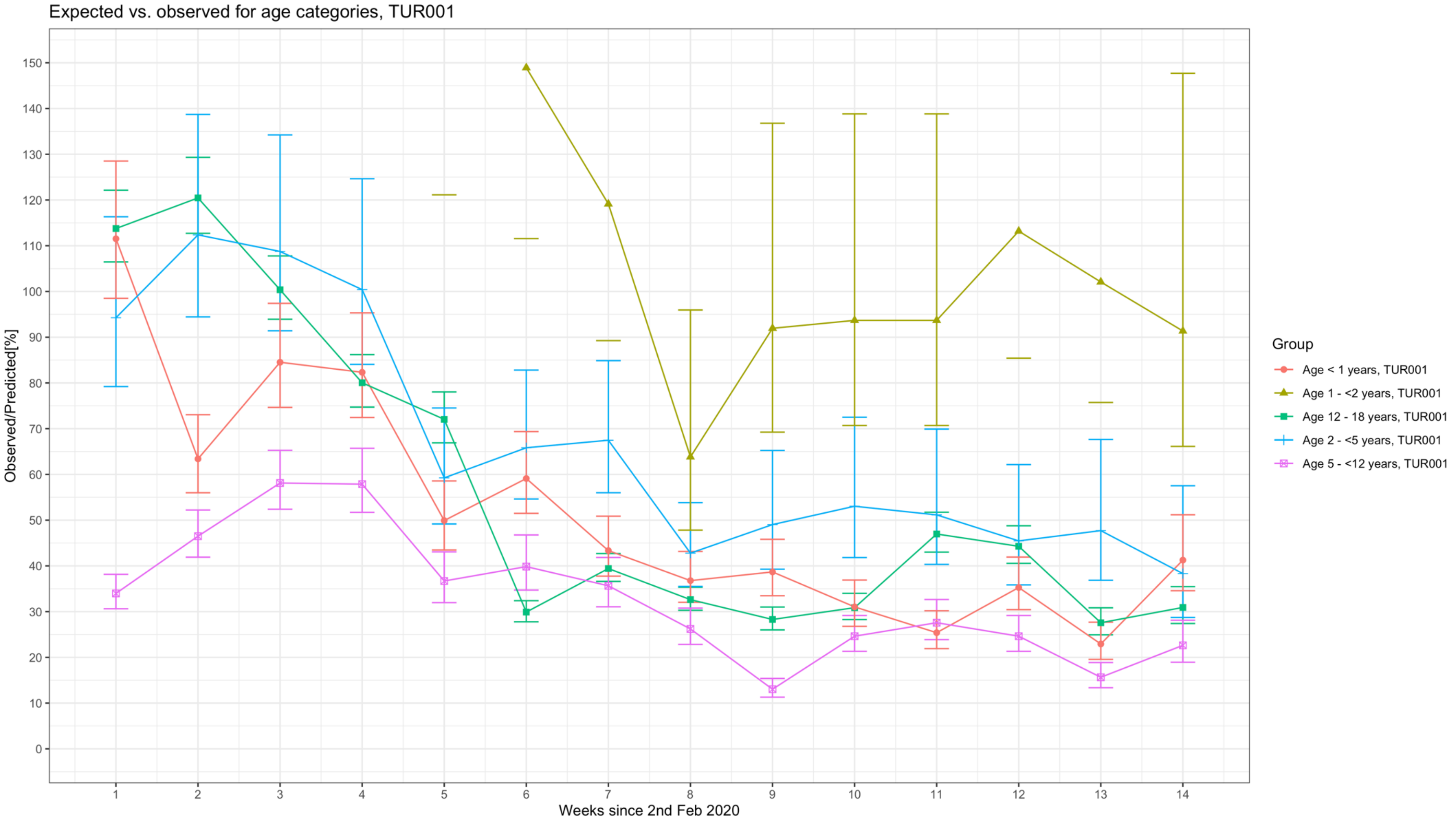

Turkey: TUR002

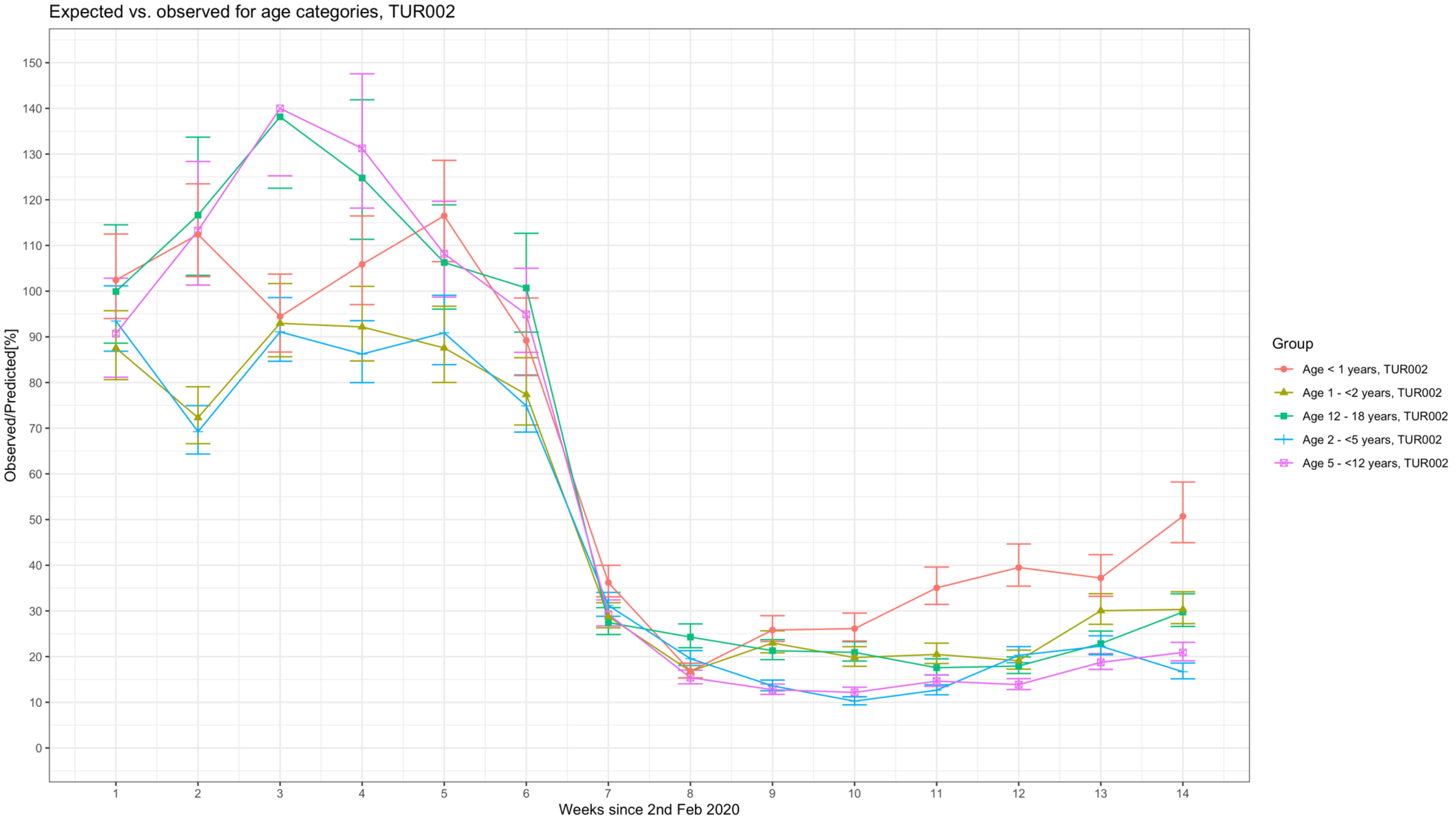

Turkey: TUR003

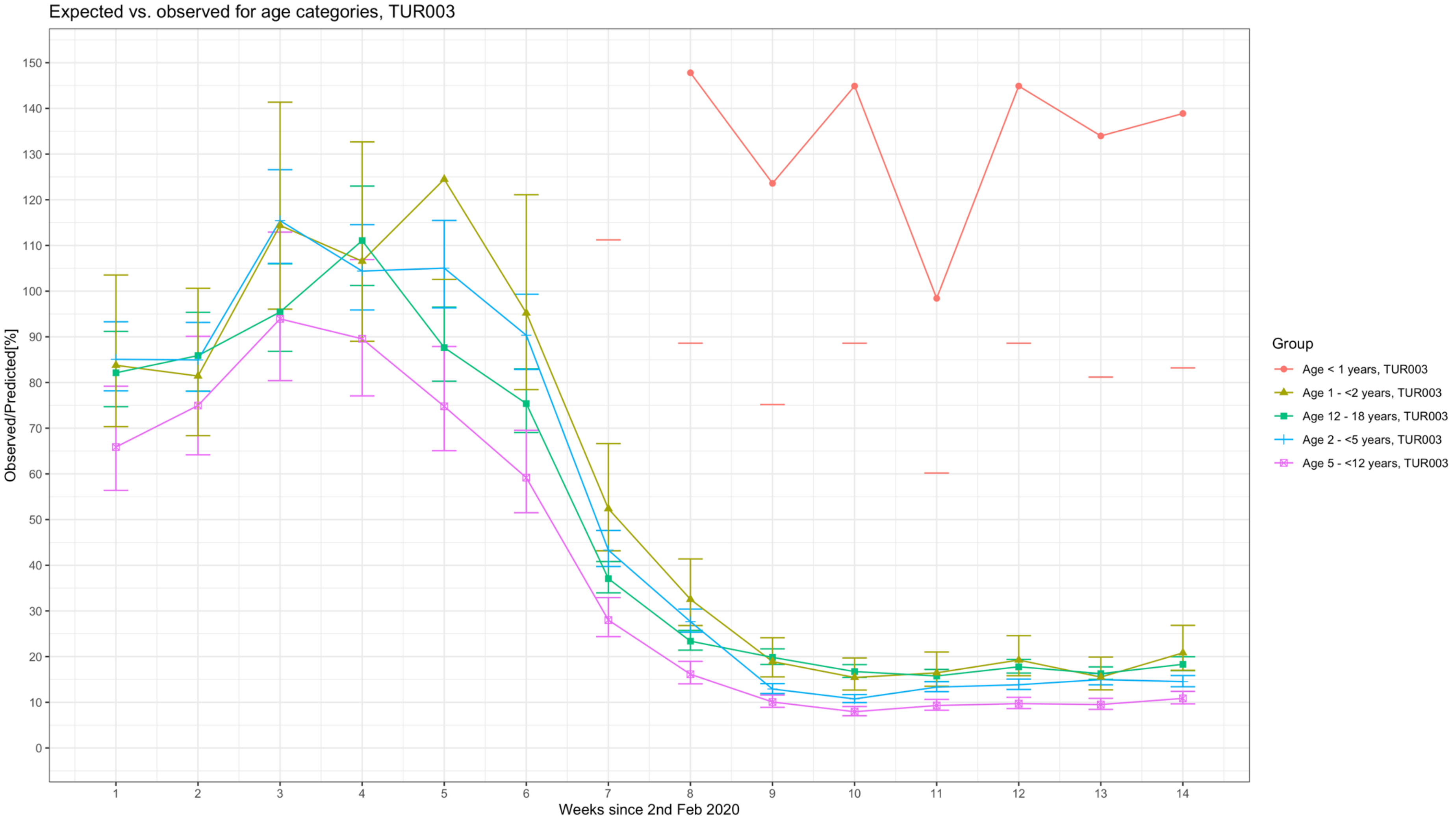

United Kingdom: UK001

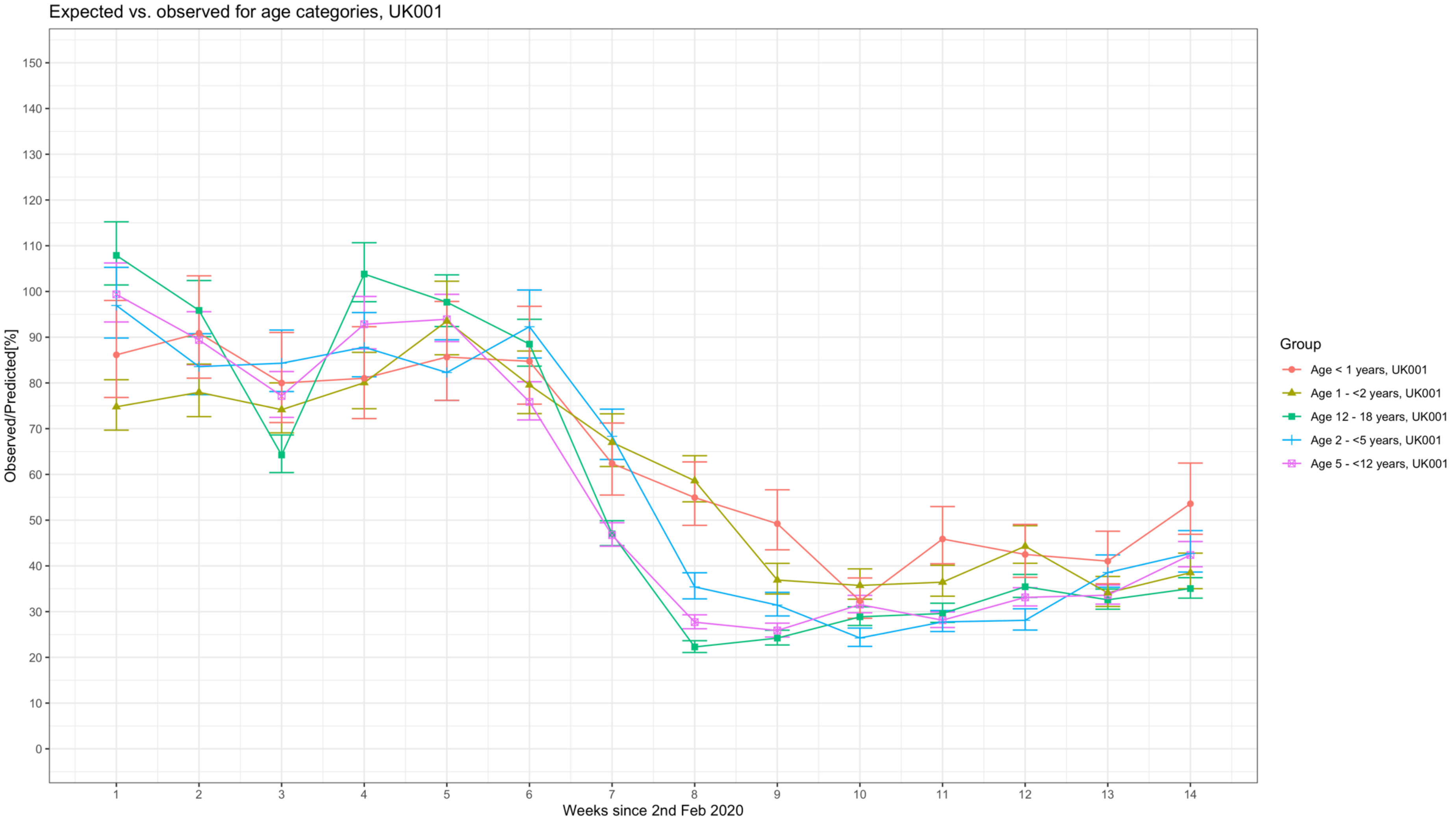

United Kingdom: UK002

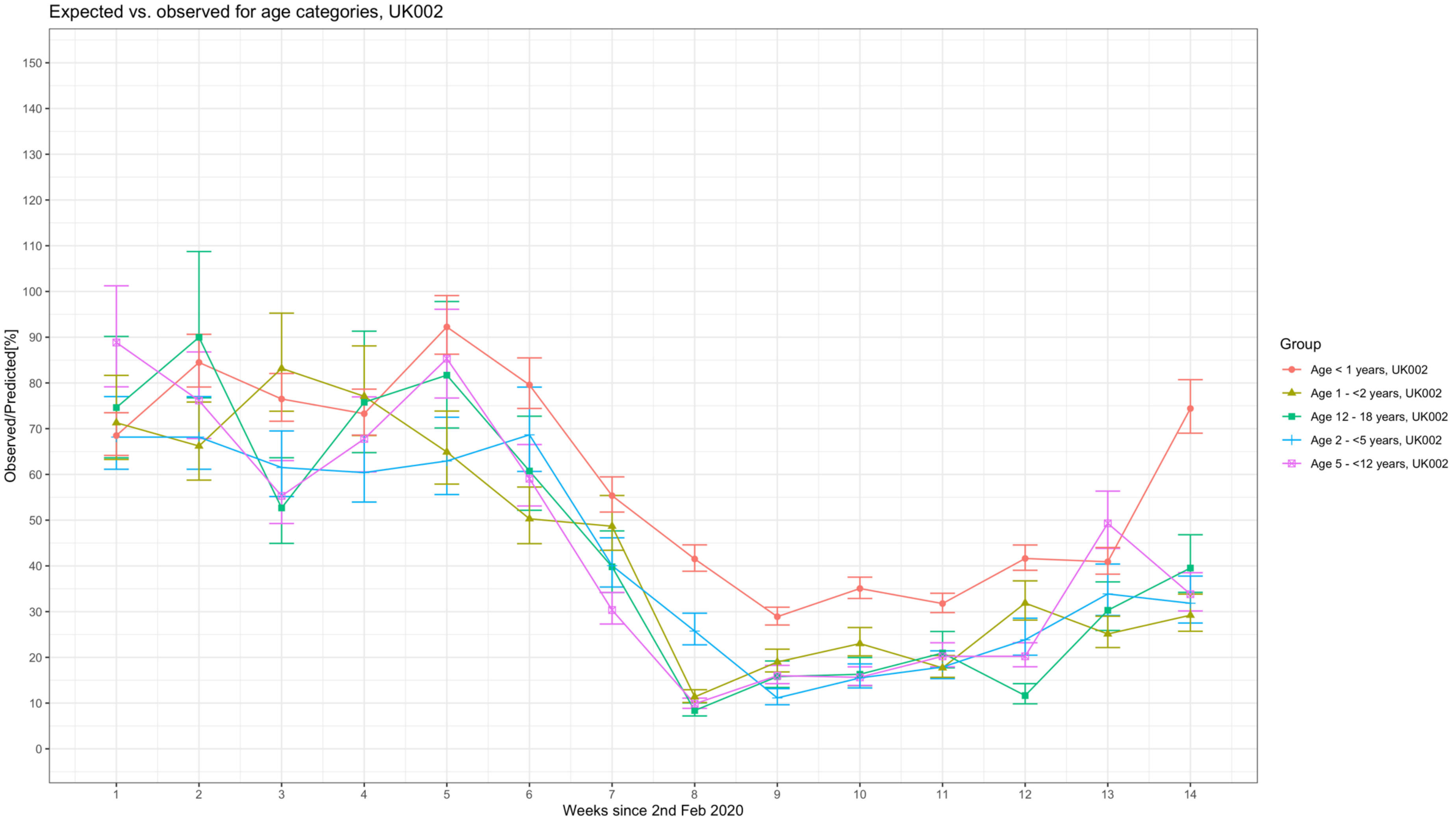

United Kingdom: UK004

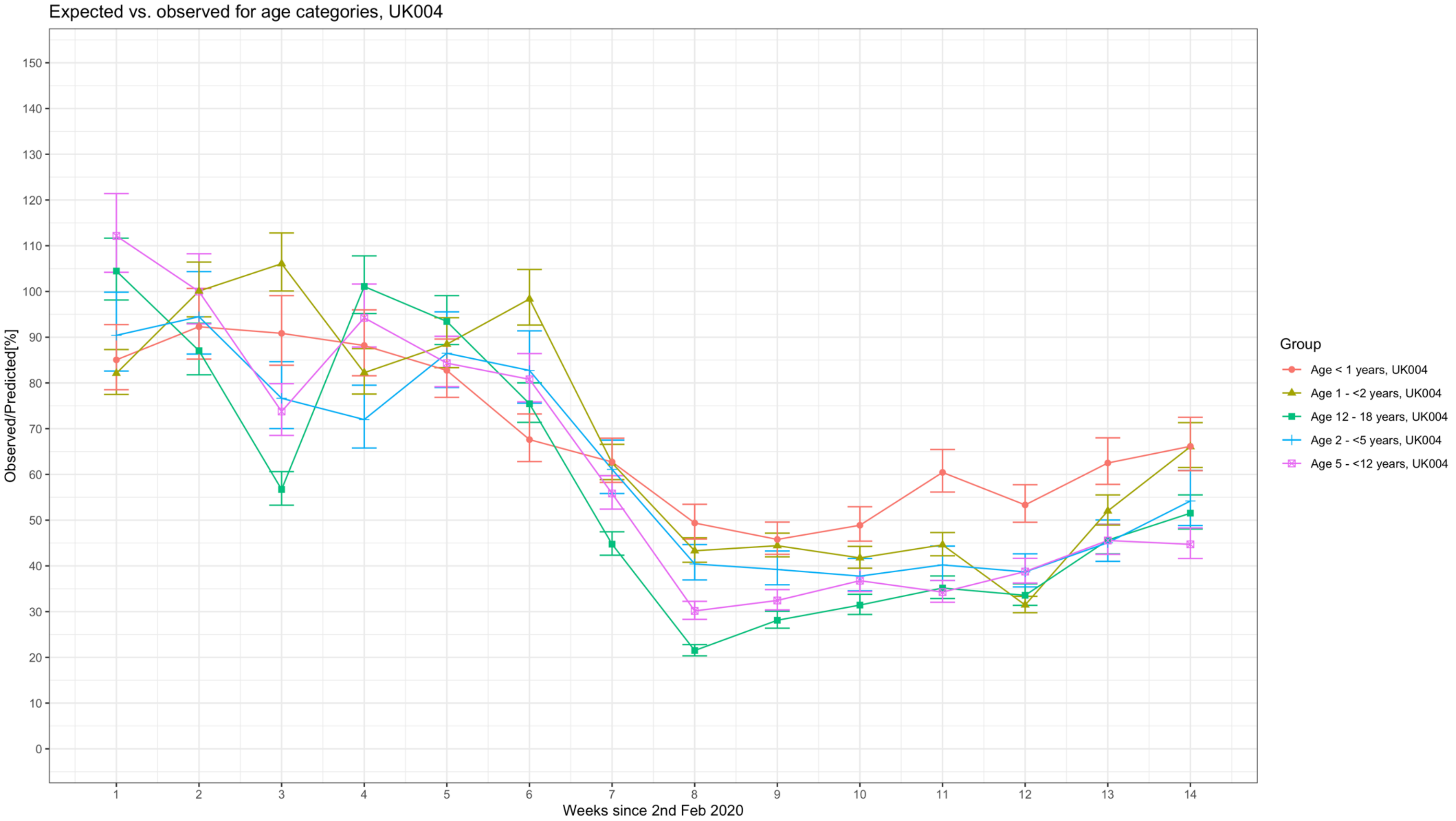

United Kingdom: UK005

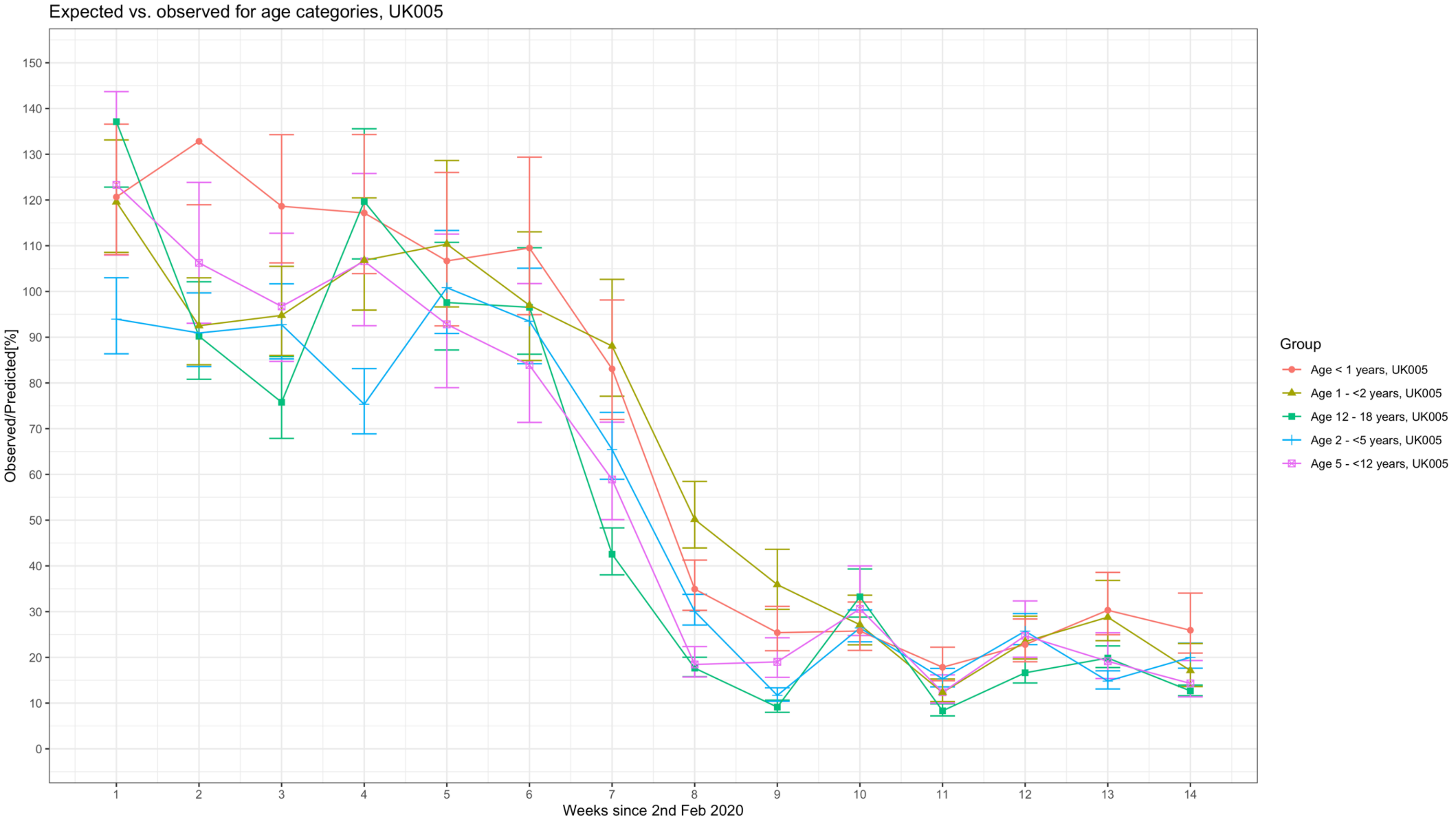

United Kingdom: UK006

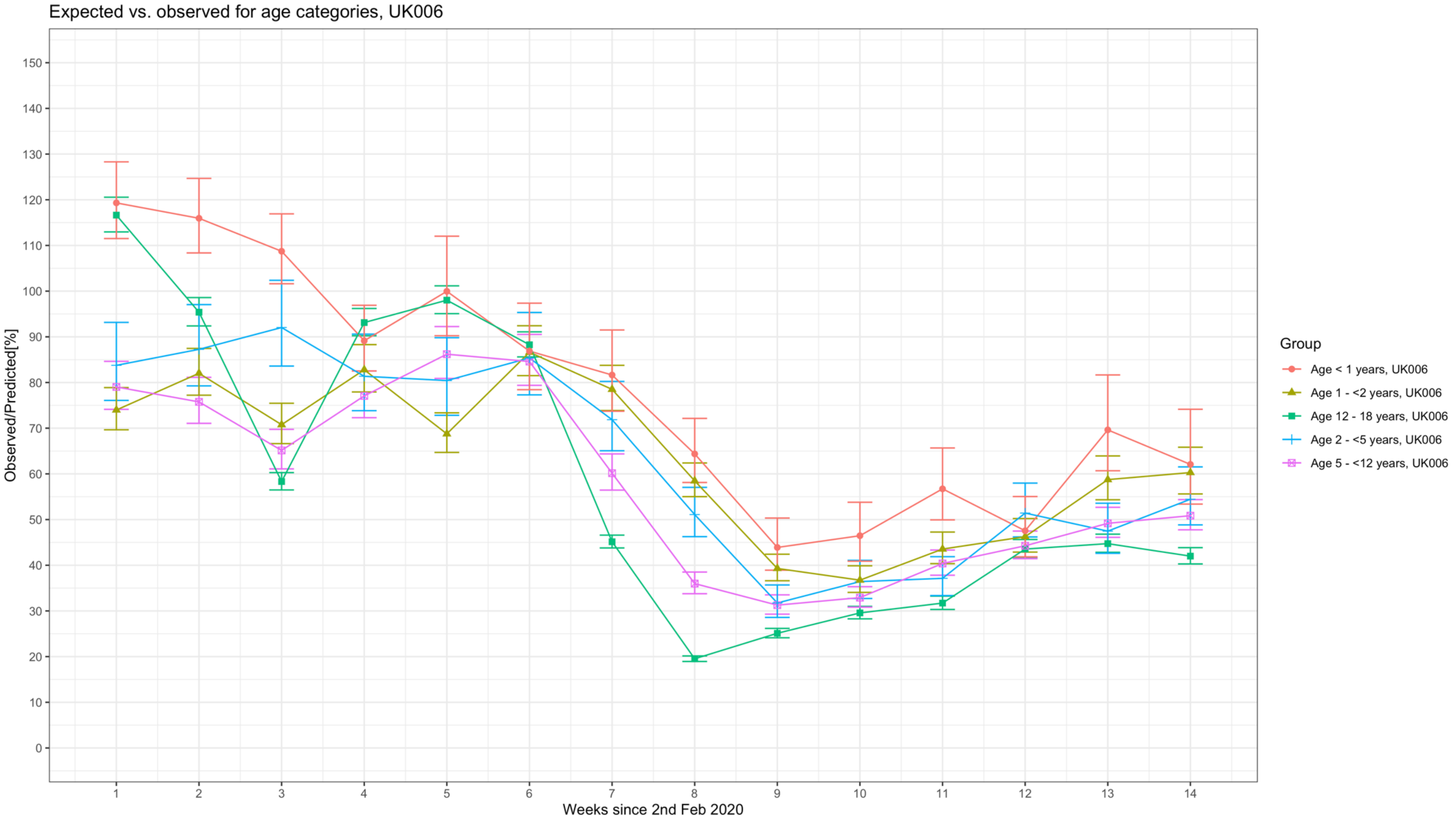

Supplement: S6 Fig — (PDF) [file pmed.1003974.s018.pdf]
