# Supplementary material for: Presentations of children to emergency departments across Europe and the COVID-19 pandemic: A multinational observational study
Source: PLoS Med. 2022 Aug 26;19(8):e1003974. doi: 10.1371/journal.pmed.1003974 (PMC9467376; doi:10.1371/journal.pmed.1003974)
Supplement: S7 Fig — The observed versus predicted number of children presenting to EDs in countries across Europe for which data from only 1 study site were available in the weeks following February 2, 2020 until May 11, 2020. A timeline is plotted (dashed line) to show the dates of the introduction of national social distancing measures.[20] One site from the Netherlands and 1 site from Hungary were excluded from these analyses as these sites could not provide data for the entire study duration. (PDF) [file pmed.1003974.s019.pdf]

S7.1 Fig. Observed versus predicted ED attendances (%) for each country.

Legend:

The observed versus predicted number of children presenting to emergency departments in countries across Europe for which data from only one study site were available in the weeks following February 2nd 2020 until May 11th 2020. A timeline is plotted (dashed line) to show the dates of the introduction of national social distancing measures.[1] One site from Netherlands and one site from Hungary were excluded from these analyses as these sites could not provide data for the entire study duration.

Austria

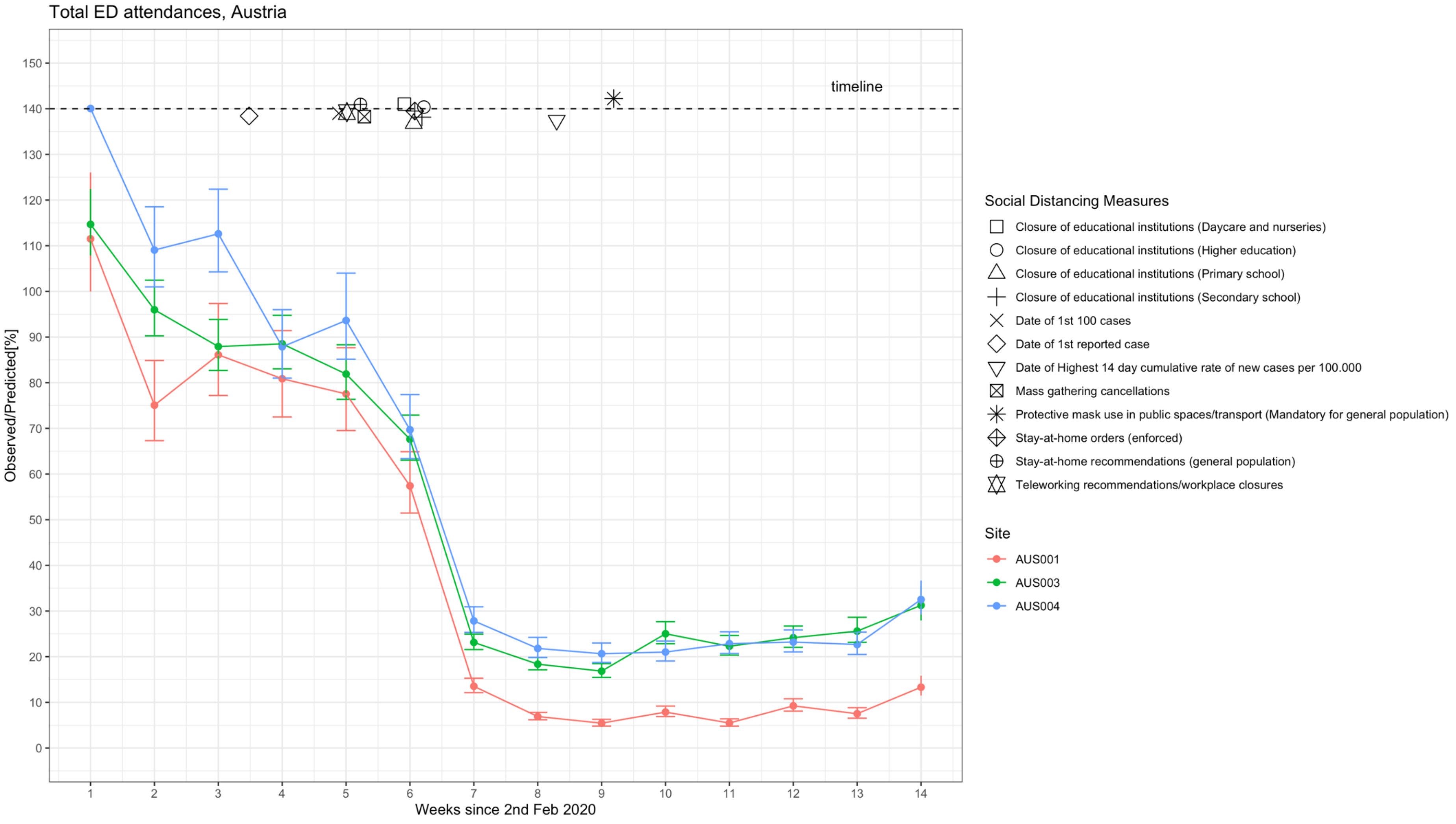

Total ED attendances, Austria
timeline
Observed/Predicted[%]
Weeks since 2nd Feb 2020
150
140
130
120
110
100
90
80
70
60
50
40
30
20
10
0
1
2
3
4
5
6
7
8
9
10
11
12
13
14
Social Distancing Measures
Closure of educational institutions (Daycare and nurseries)
Closure of educational institutions (Higher education)
Closure of educational institutions (Primary school)
Closure of educational institutions (Secondary school)
Date of 1st 100 cases
Date of 1st reported case
Date of Highest 14 day cumulative rate of new cases per 100.000
Mass gathering cancellations
Protective mask use in public spaces/transport (Mandatory for general population)
Stay-at-home orders (enforced)
Stay-at-home recommendations (general population)
Teleworking recommendations/workplace closures
Site
AUS001
AUS003
AUS004

France

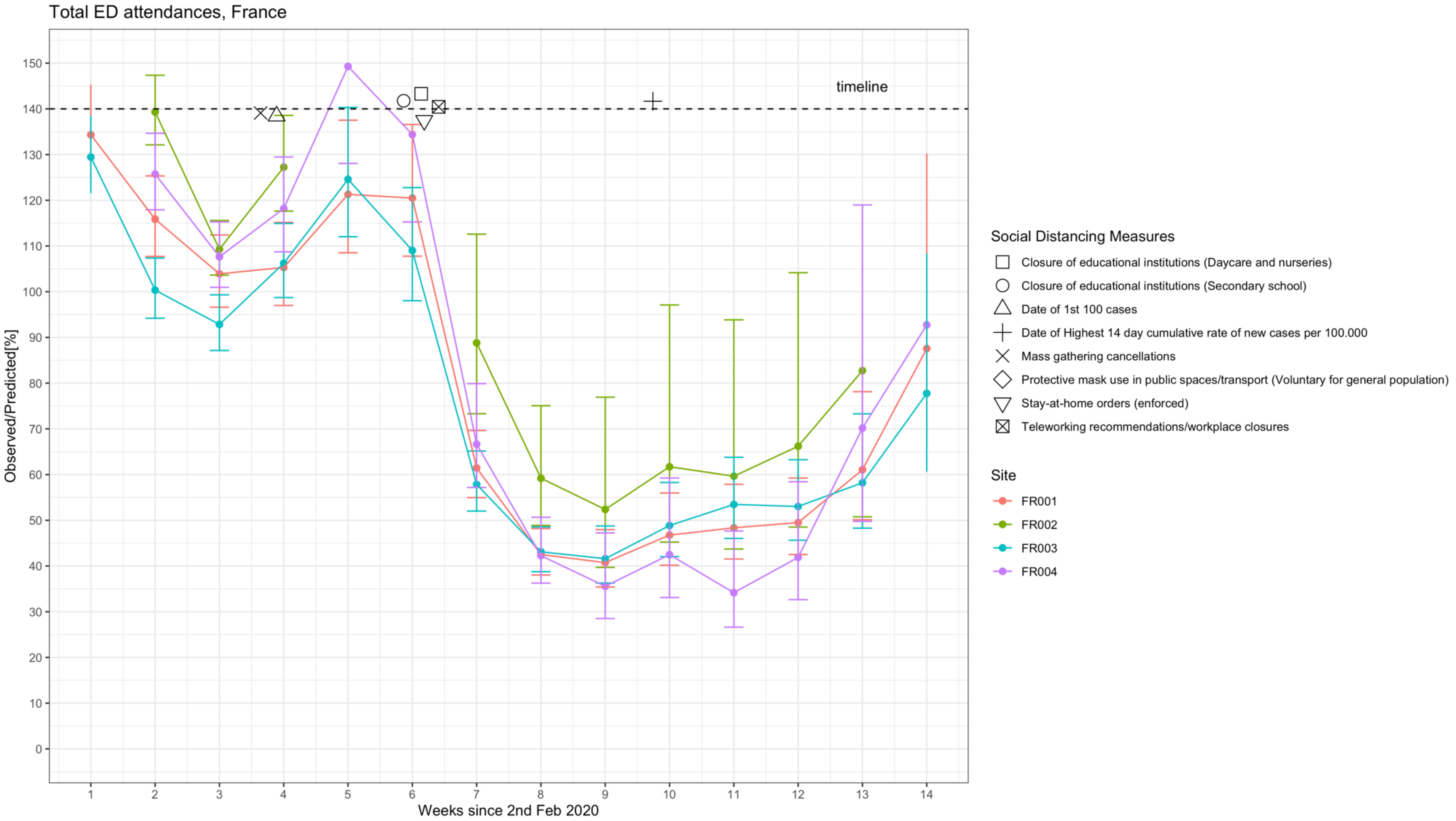

Total ED attendances, France
Observed/Predicted[%]
0
10
20
30
40
50
60
70
80
90
100
110
120
130
140
150
timeline
1
2
3
4
5
6
7
8
9
10
11
12
13
14
Weeks since 2nd Feb 2020
Social Distancing Measures
Closure of educational institutions (Daycare and nurseries)
Closure of educational institutions (Secondary school)
Date of 1st 100 cases
Date of Highest 14 day cumulative rate of new cases per 100.000
Mass gathering cancellations
Protective mask use in public spaces/transport (Voluntary for general population)
Stay-at-home orders (enforced)
Teleworking recommendations/workplace closures
Site
FR001
FR002
FR003
FR004

Italy

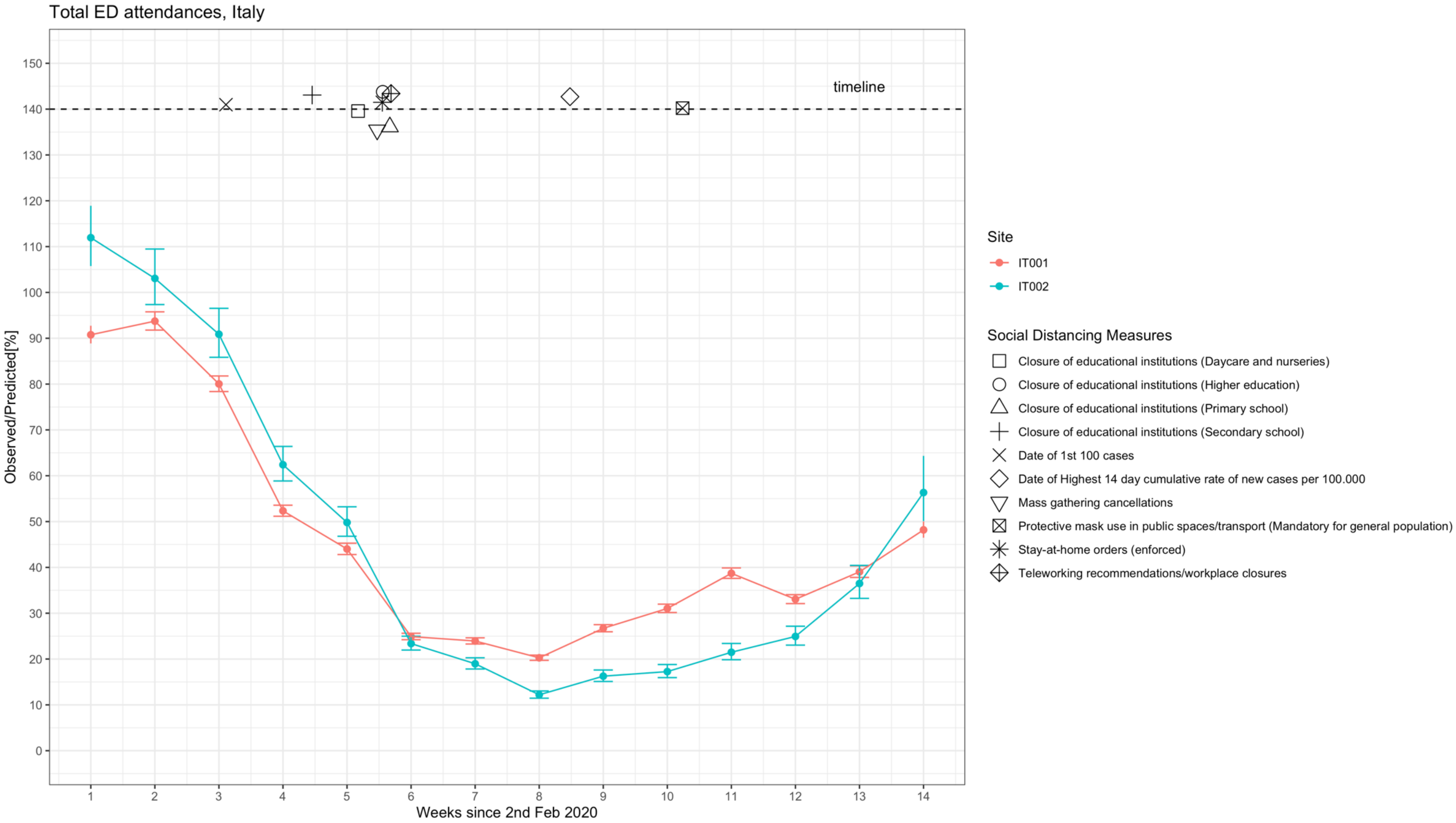

Total ED attendances, Italy
timeline
Observed/Predicted[%]
150
140
130
120
110
100
90
80
70
60
50
40
30
20
10
0
1
2
3
4
5
6
7
8
9
10
11
12
13
14
Weeks since 2nd Feb 2020
Site
IT001
IT002
Social Distancing Measures
Closure of educational institutions (Daycare and nurseries)
Closure of educational institutions (Higher education)
Closure of educational institutions (Primary school)
Closure of educational institutions (Secondary school)
Date of 1st 100 cases
Date of Highest 14 day cumulative rate of new cases per 100.000
Mass gathering cancellations
Protective mask use in public spaces/transport (Mandatory for general population)
Stay-at-home orders (enforced)
Teleworking recommendations/workplace closures

Ireland

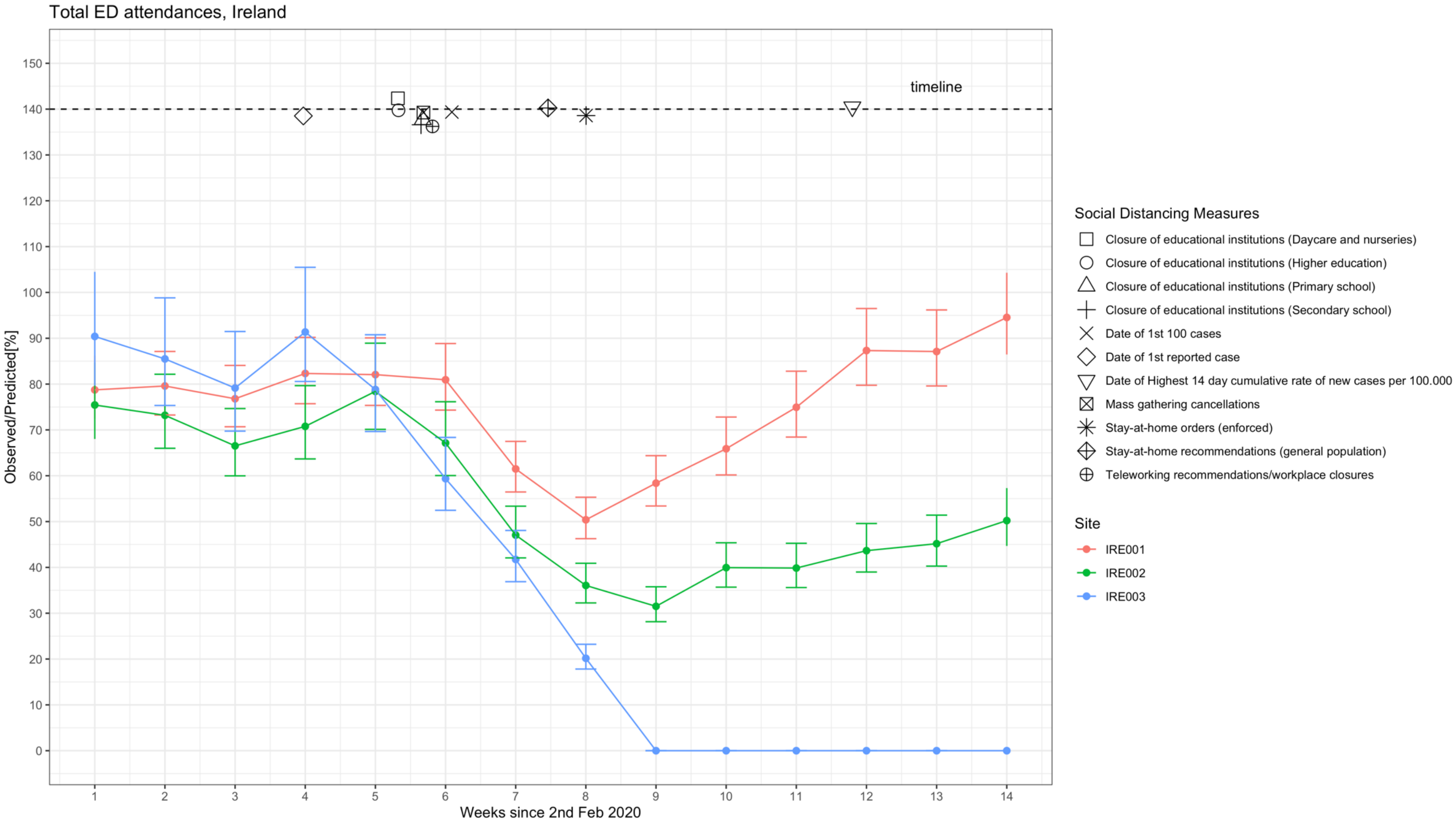

Total ED attendances, Ireland
timeline
Observed/Predicted[%]
150
140
130
120
110
100
90
80
70
60
50
40
30
20
10
0
1
2
3
4
5
6
7
8
9
10
11
12
13
14
Weeks since 2nd Feb 2020
Social Distancing Measures
Closure of educational institutions (Daycare and nurseries)
Closure of educational institutions (Higher education)
Closure of educational institutions (Primary school)
Closure of educational institutions (Secondary school)
Date of 1st 100 cases
Date of 1st reported case
Date of Highest 14 day cumulative rate of new cases per 100.000
Mass gathering cancellations
Stay-at-home orders (enforced)
Stay-at-home recommendations (general population)
Teleworking recommendations/workplace closures
Site
IRE001
IRE002
IRE003

Portugal

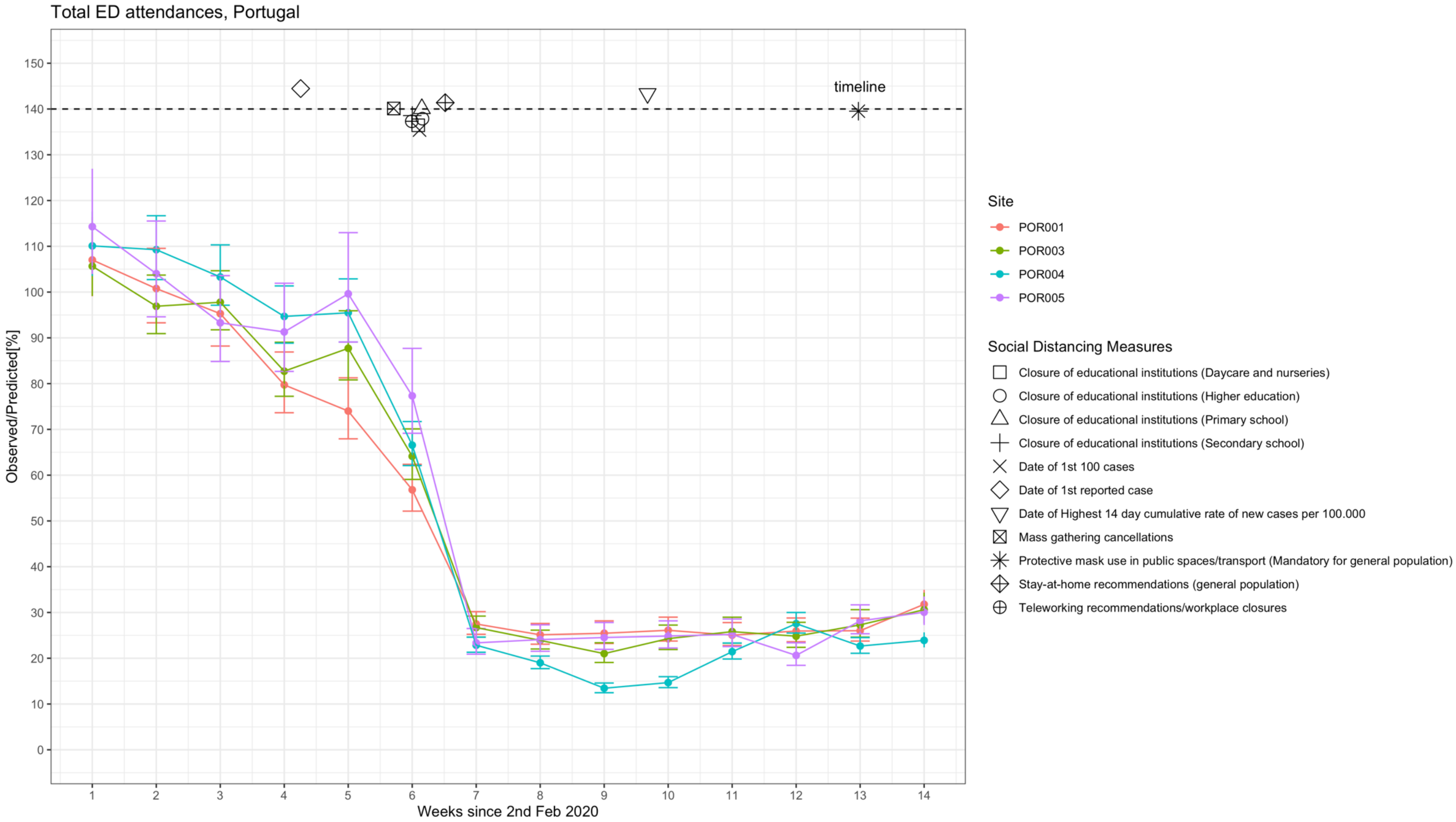

Total ED attendances, Portugal
150
140
130
120
110
100
90
80
70
60
50
40
30
20
10
0
Observed/Predicted[%]
timeline
1
2
3
4
5
6
7
8
9
10
11
12
13
14
Weeks since 2nd Feb 2020
Site
POR001
POR003
POR004
POR005
Social Distancing Measures
Closure of educational institutions (Daycare and nurseries)
Closure of educational institutions (Higher education)
Closure of educational institutions (Primary school)
Closure of educational institutions (Secondary school)
Date of 1st 100 cases
Date of 1st reported case
Date of Highest 14 day cumulative rate of new cases per 100.000
Mass gathering cancellations
Protective mask use in public spaces/transport (Mandatory for general population)
Stay-at-home recommendations (general population)
Teleworking recommendations/workplace closures

Spain

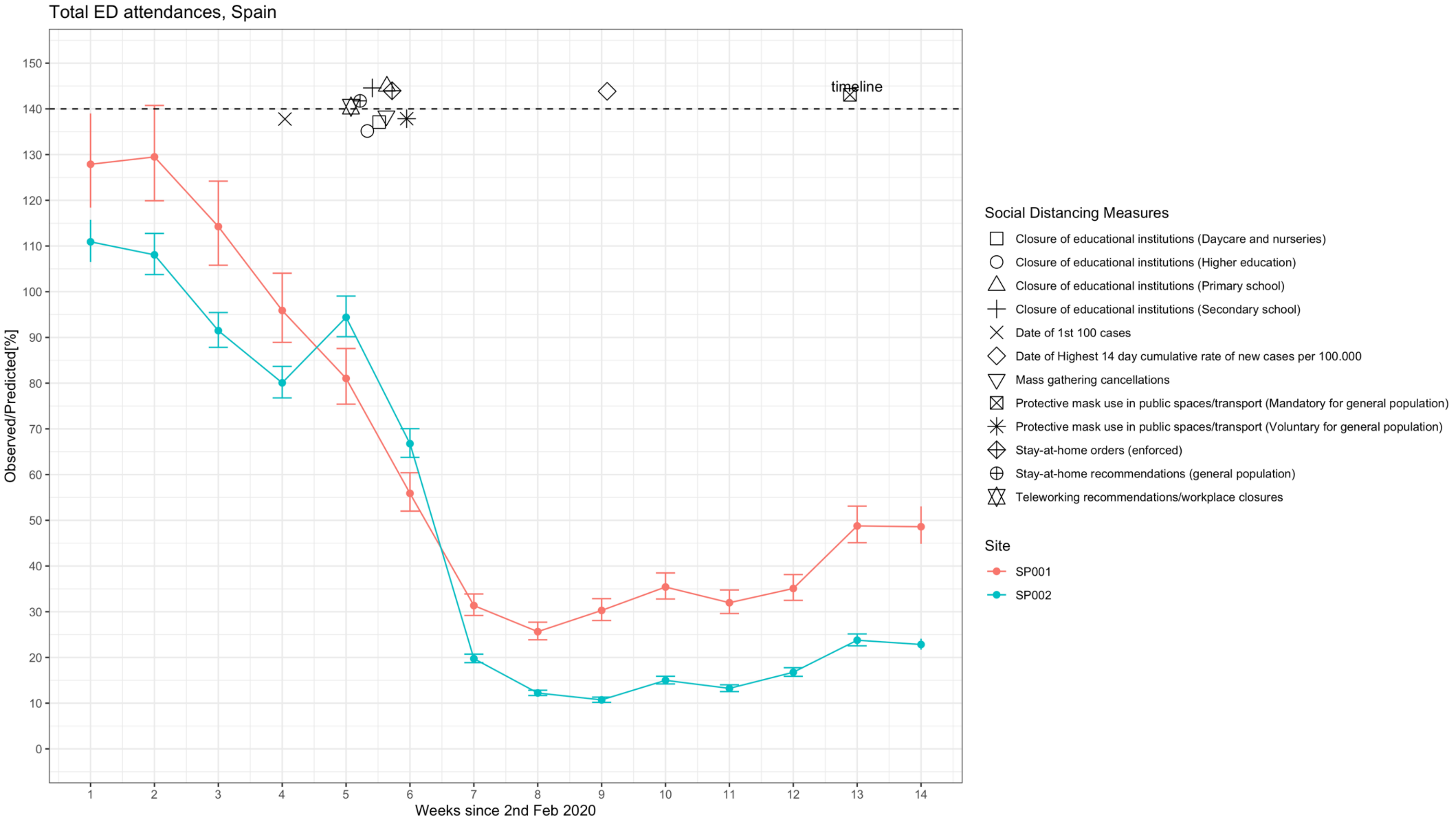

Total ED attendances, Spain
Observed/Predicted[%]
Weeks since 2nd Feb 2020
timeline
Social Distancing Measures
Closure of educational institutions (Daycare and nurseries)
Closure of educational institutions (Higher education)
Closure of educational institutions (Primary school)
Closure of educational institutions (Secondary school)
Date of 1st 100 cases
Date of Highest 14 day cumulative rate of new cases per 100.000
Mass gathering cancellations
Protective mask use in public spaces/transport (Mandatory for general population)
Protective mask use in public spaces/transport (Voluntary for general population)
Stay-at-home orders (enforced)
Stay-at-home recommendations (general population)
Teleworking recommendations/workplace closures
Site
SP001
SP002

Sweden

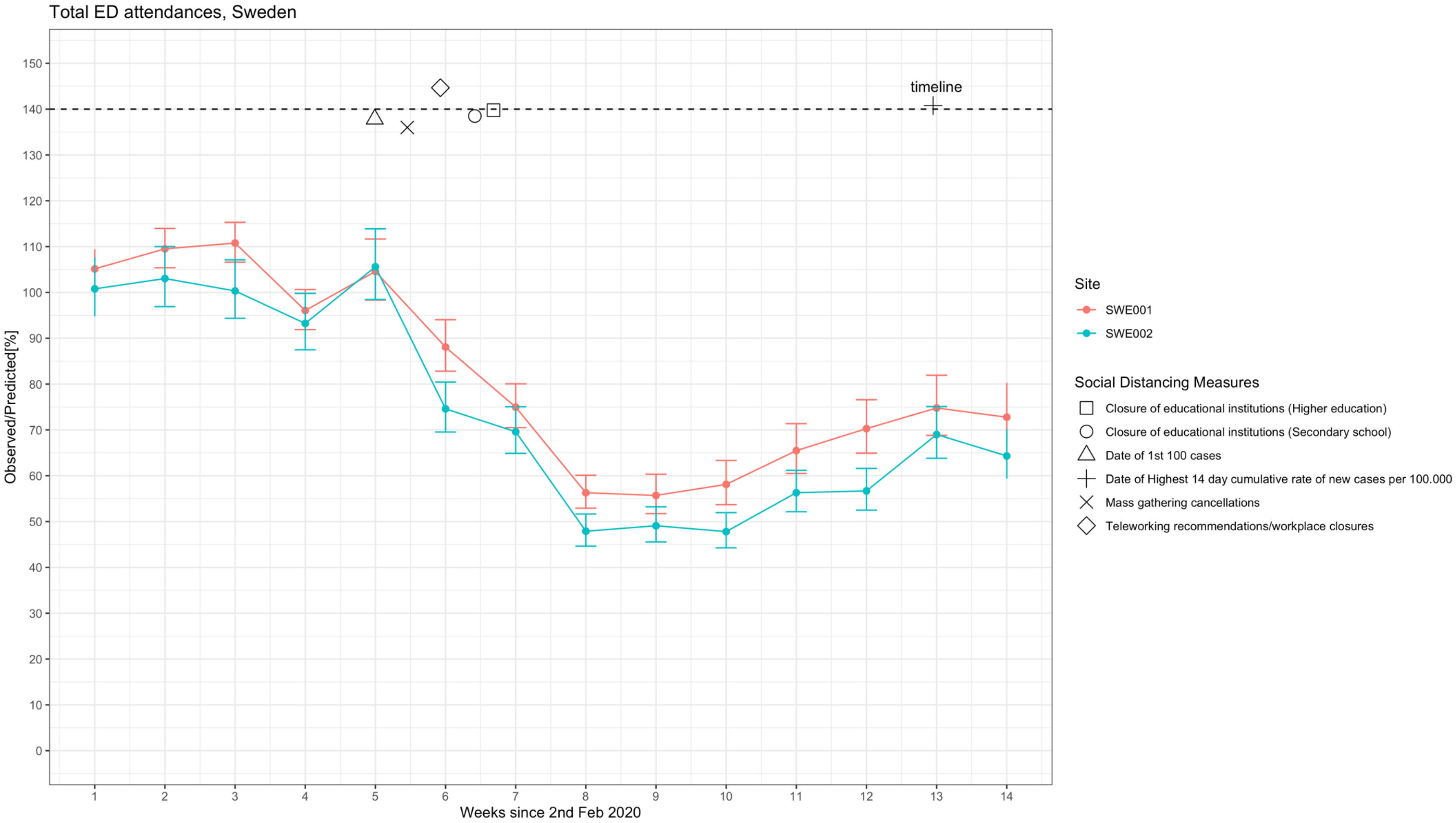

Total ED attendances, Sweden
timeline
Observed/Predicted[%]
Weeks since 2nd Feb 2020
Site
SWE001
SWE002
Social Distancing Measures
Closure of educational institutions (Higher education)
Closure of educational institutions (Secondary school)
Date of 1st 100 cases
Date of Highest 14 day cumulative rate of new cases per 100.000
Mass gathering cancellations
Teleworking recommendations/workplace closures

Turkey

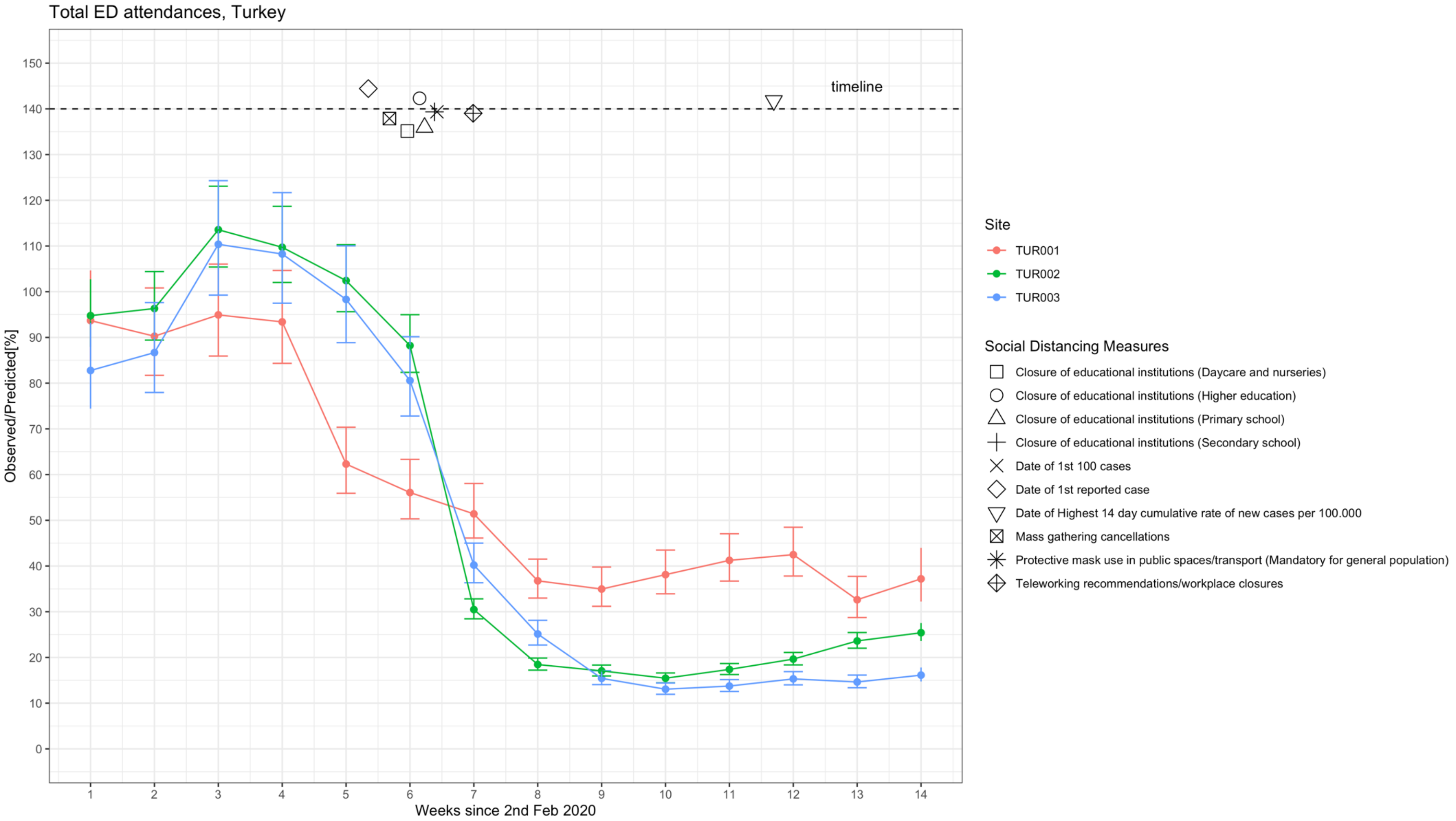

Total ED attendances, Turkey
timeline
Observed/Predicted[%]
Weeks since 2nd Feb 2020
Site
TUR001
TUR002
TUR003
Social Distancing Measures
Closure of educational institutions (Daycare and nurseries)
Closure of educational institutions (Higher education)
Closure of educational institutions (Primary school)
Closure of educational institutions (Secondary school)
Date of 1st 100 cases
Date of 1st reported case
Date of Highest 14 day cumulative rate of new cases per 100.000
Mass gathering cancellations
Protective mask use in public spaces/transport (Mandatory for general population)
Teleworking recommendations/workplace closures

United Kingdom

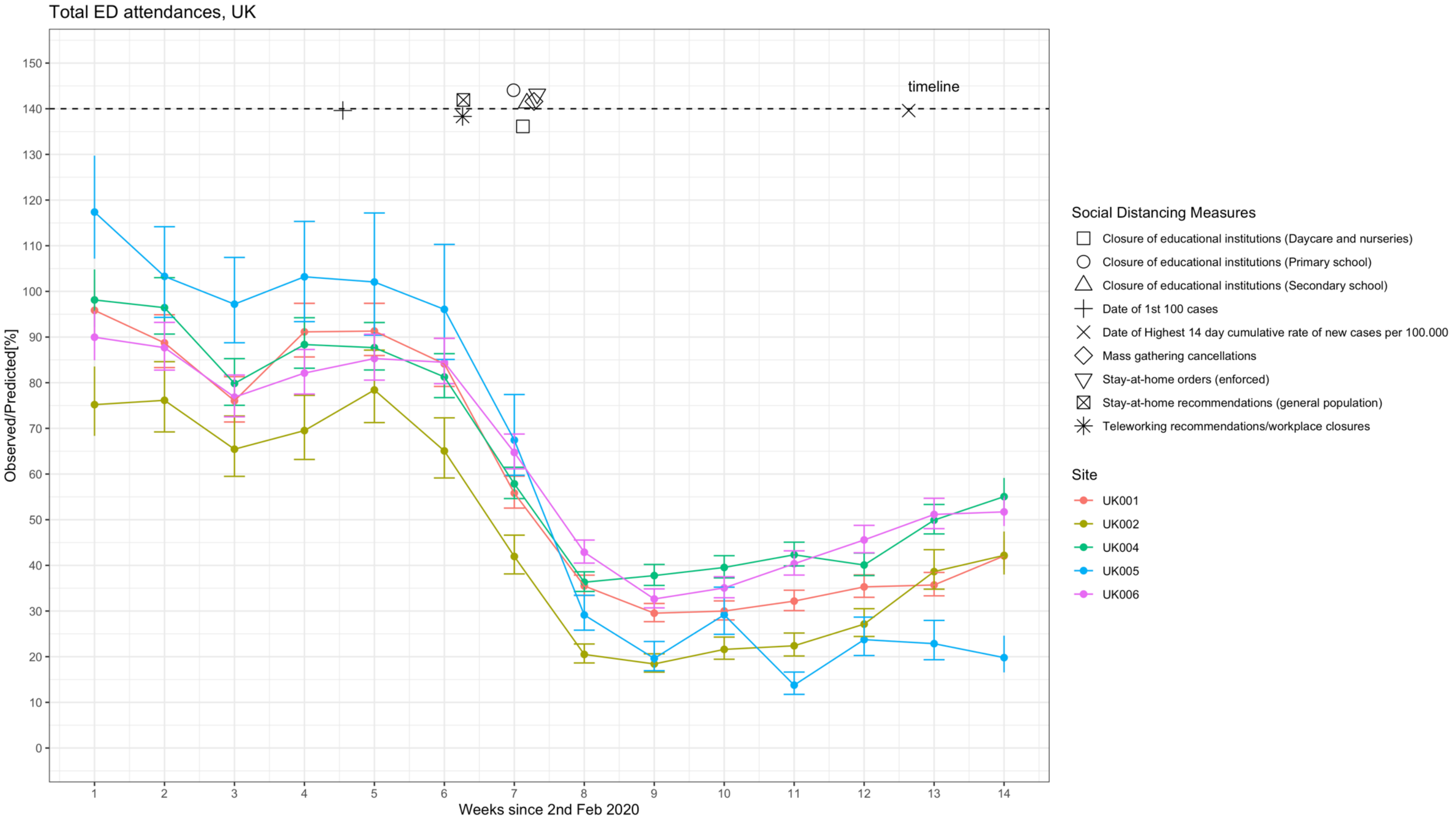

S7.2 Fig. Graphs of reduced versus observed total emergency department attendances (%), for countries with one participating site

Germany

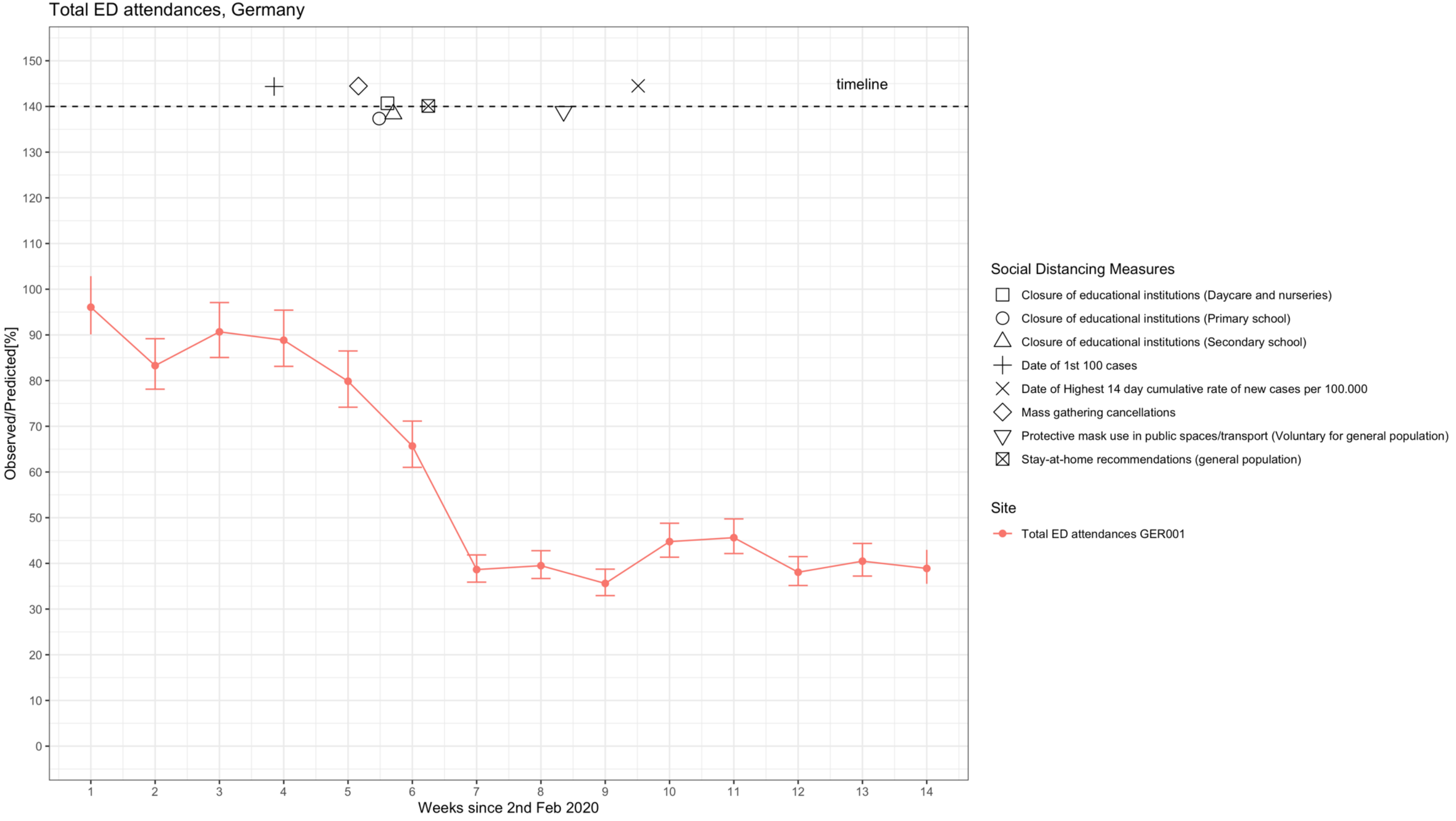

Hungary

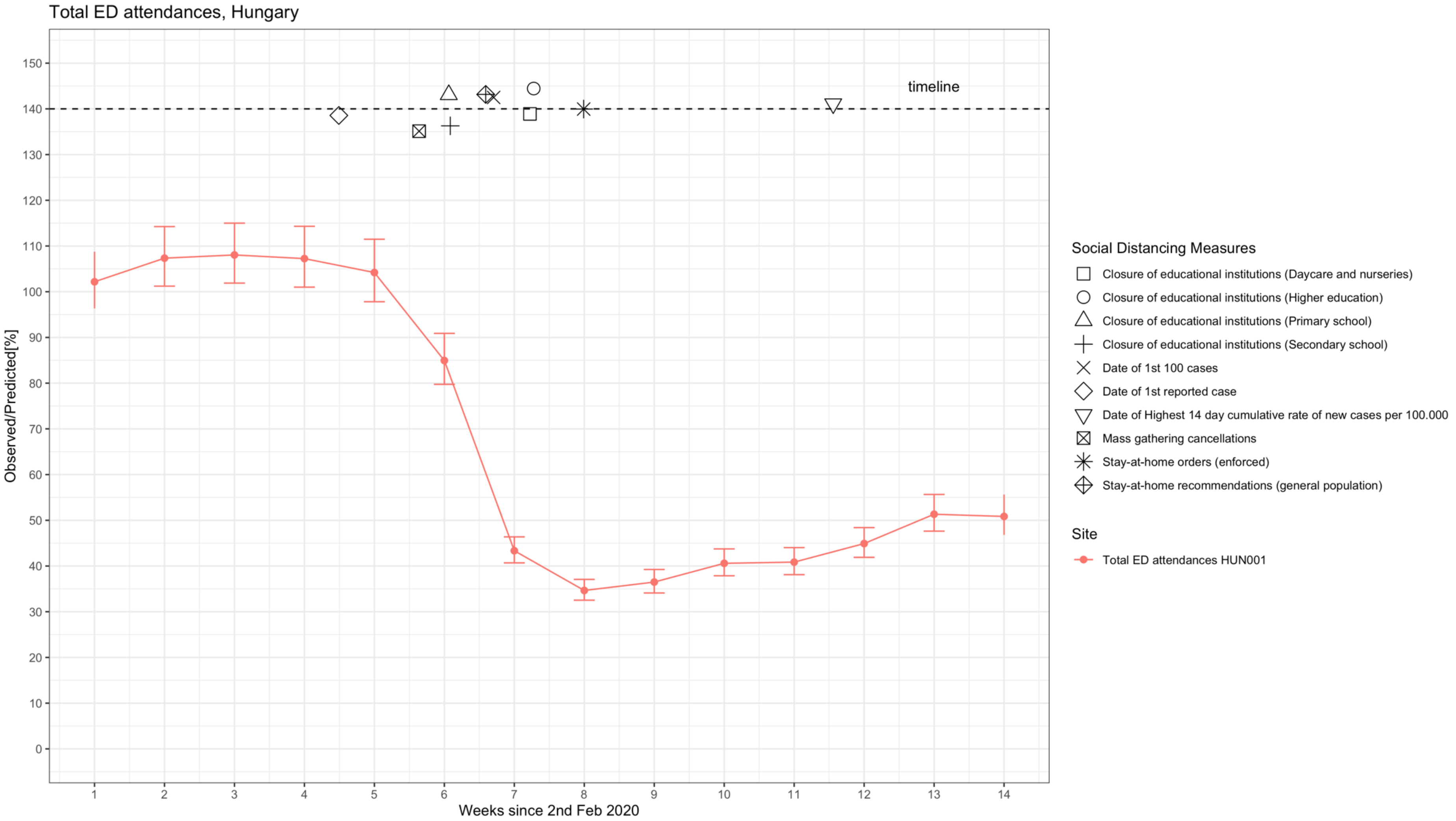
Total ED attendances, Hungary
timeline
150
140
130
120
110
100
90
80
70
60
50
40
30
20
10
0
Observed/Predicted[%]
1
2
3
4
5
6
7
8
9
10
11
12
13
14
Weeks since 2nd Feb 2020
Social Distancing Measures
Closure of educational institutions (Daycare and nurseries)
Closure of educational institutions (Higher education)
Closure of educational institutions (Primary school)
Closure of educational institutions (Secondary school)
Date of 1st 100 cases
Date of 1st reported case
Date of Highest 14 day cumulative rate of new cases per 100.000
Mass gathering cancellations
Stay-at-home orders (enforced)
Stay-at-home recommendations (general population)
Site
Total ED attendances HUN001

Iceland

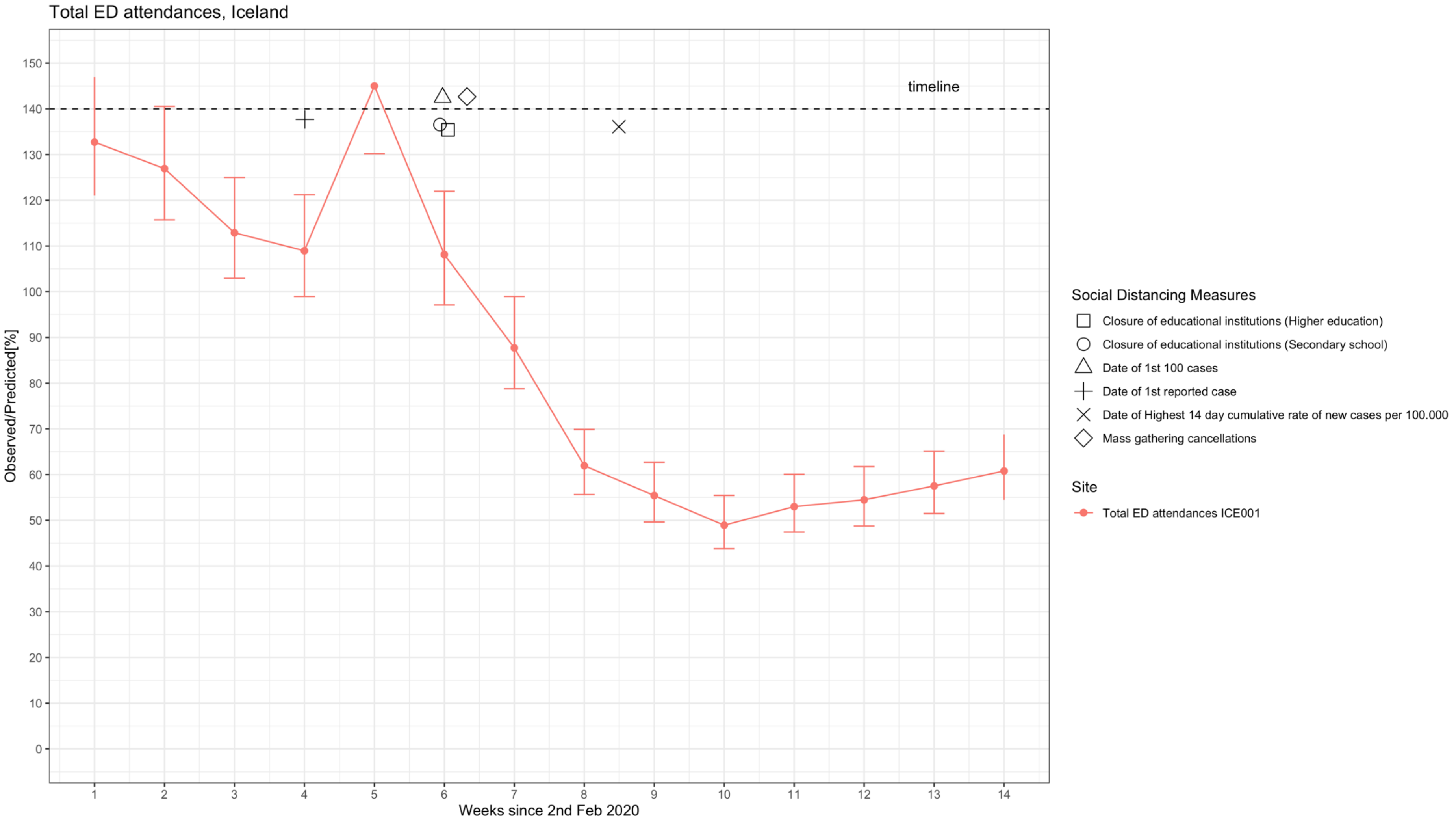

Total ED attendances, Iceland
timeline
Observed/Predicted[%]
Weeks since 2nd Feb 2020
0
10
20
30
40
50
60
70
80
90
100
110
120
130
140
150
1
2
3
4
5
6
7
8
9
10
11
12
13
14
Social Distancing Measures
Closure of educational institutions (Higher education)
Closure of educational institutions (Secondary school)
Date of 1st 100 cases
Date of 1st reported case
Date of Highest 14 day cumulative rate of new cases per 100.000
Mass gathering cancellations
Site
Total ED attendances ICE001

Latvia

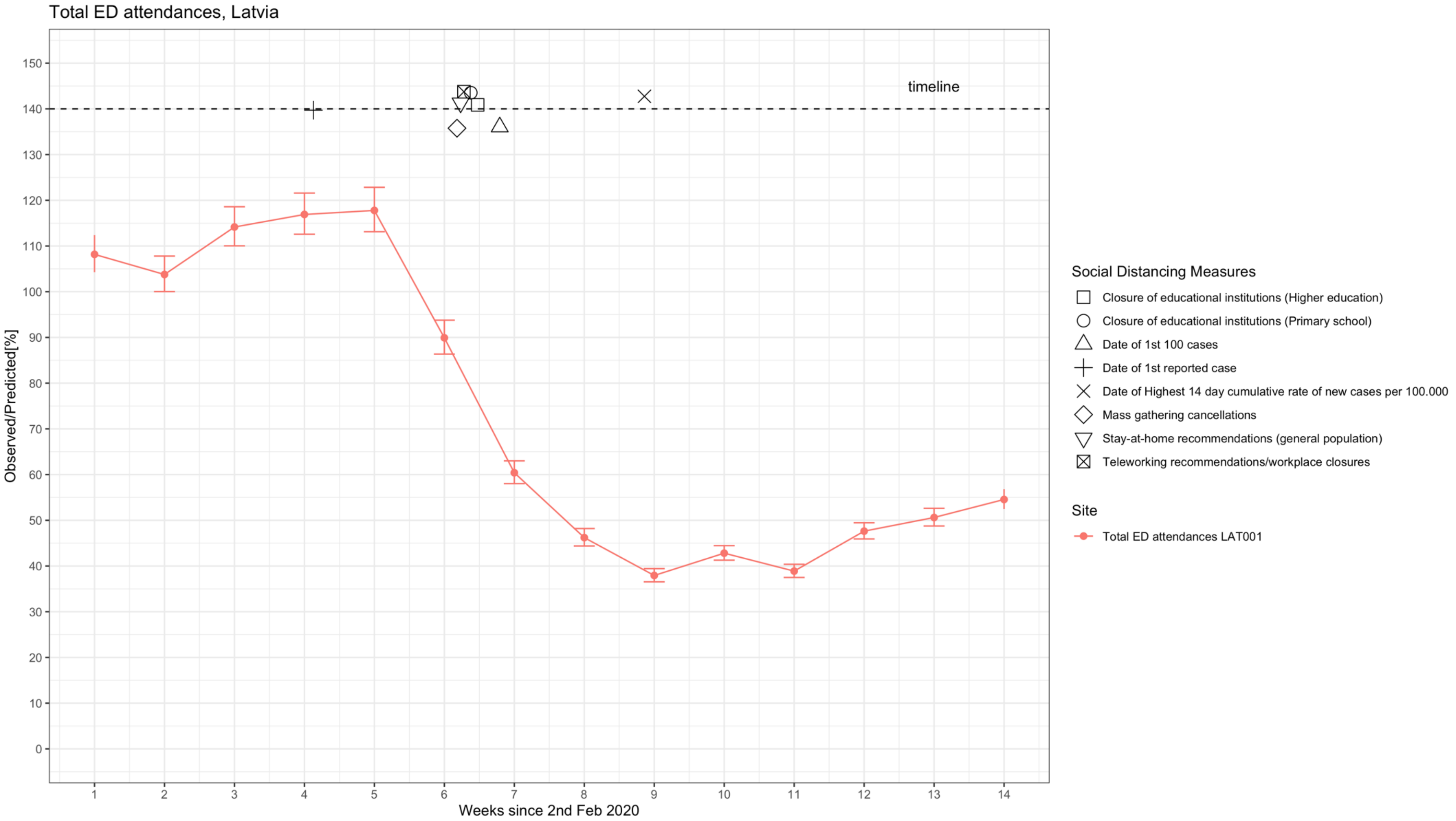

Total ED attendances, Latvia
timeline
Observed/Predicted[%]
150
140
130
120
110
100
90
80
70
60
50
40
30
20
10
0
1
2
3
4
5
6
7
8
9
10
11
12
13
14
Weeks since 2nd Feb 2020
Social Distancing Measures
Closure of educational institutions (Higher education)
Closure of educational institutions (Primary school)
Date of 1st 100 cases
Date of 1st reported case
Date of Highest 14 day cumulative rate of new cases per 100.000
Mass gathering cancellations
Stay-at-home recommendations (general population)
Teleworking recommendations/workplace closures
Site
Total ED attendances LAT001

Lithuania

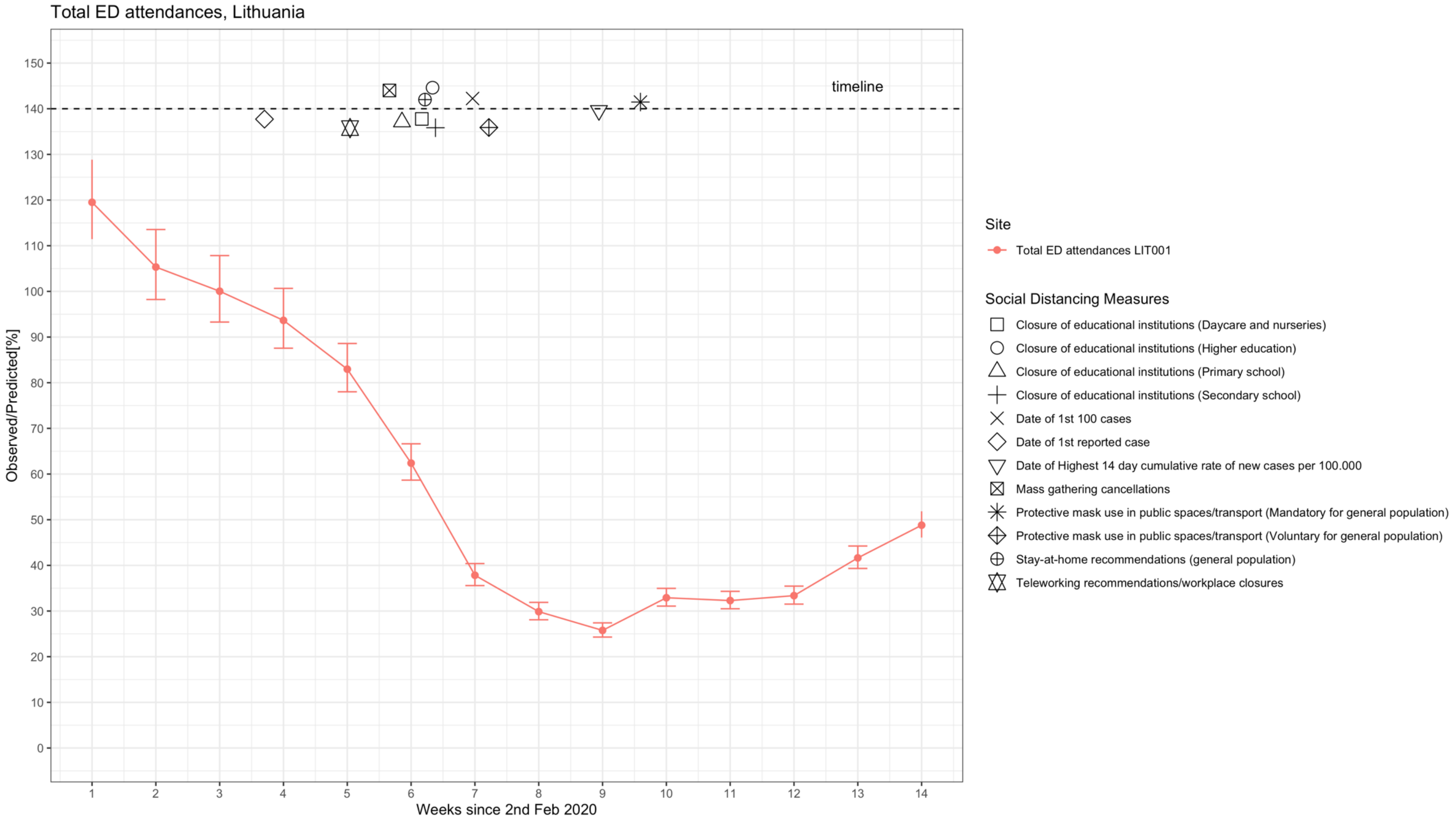

Total ED attendances, Lithuania
Observed/Predicted[%]
Weeks since 2nd Feb 2020
timeline
Site
Total ED attendances LIT001
Social Distancing Measures
Closure of educational institutions (Daycare and nurseries)
Closure of educational institutions (Higher education)
Closure of educational institutions (Primary school)
Closure of educational institutions (Secondary school)
Date of 1st 100 cases
Date of 1st reported case
Date of Highest 14 day cumulative rate of new cases per 100.000
Mass gathering cancellations
Protective mask use in public spaces/transport (Mandatory for general population)
Protective mask use in public spaces/transport (Voluntary for general population)
Stay-at-home recommendations (general population)
Teleworking recommendations/workplace closures

Malta

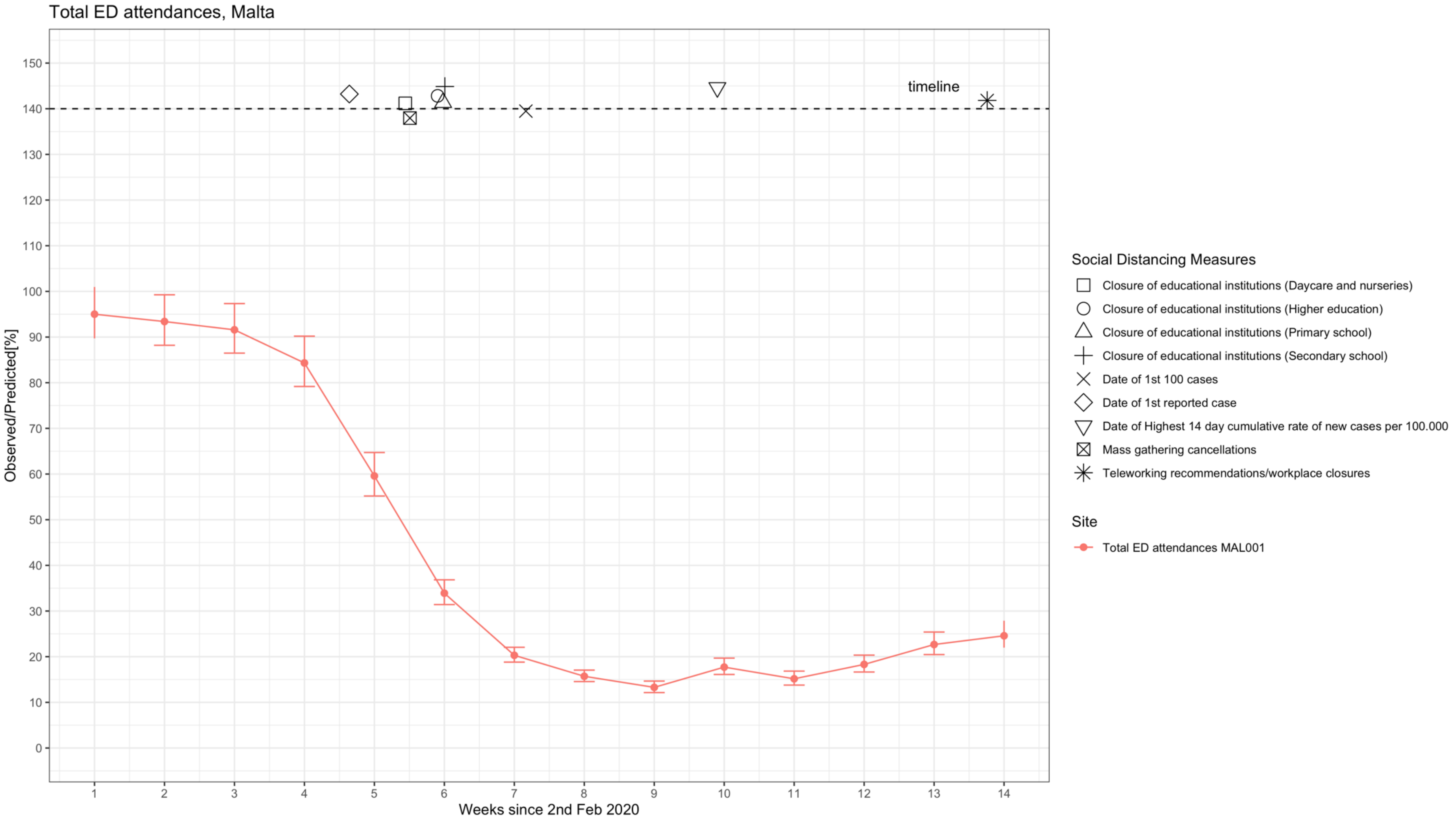

Total ED attendances, Malta
timeline
Observed/Predicted[%]
150
140
130
120
110
100
90
80
70
60
50
40
30
20
10
0
1
2
3
4
5
6
7
8
9
10
11
12
13
14
Weeks since 2nd Feb 2020
Social Distancing Measures
Closure of educational institutions (Daycare and nurseries)
Closure of educational institutions (Higher education)
Closure of educational institutions (Primary school)
Closure of educational institutions (Secondary school)
Date of 1st 100 cases
Date of 1st reported case
Date of Highest 14 day cumulative rate of new cases per 100.000
Mass gathering cancellations
Teleworking recommendations/workplace closures
Site
Total ED attendances MAL001

Netherlands

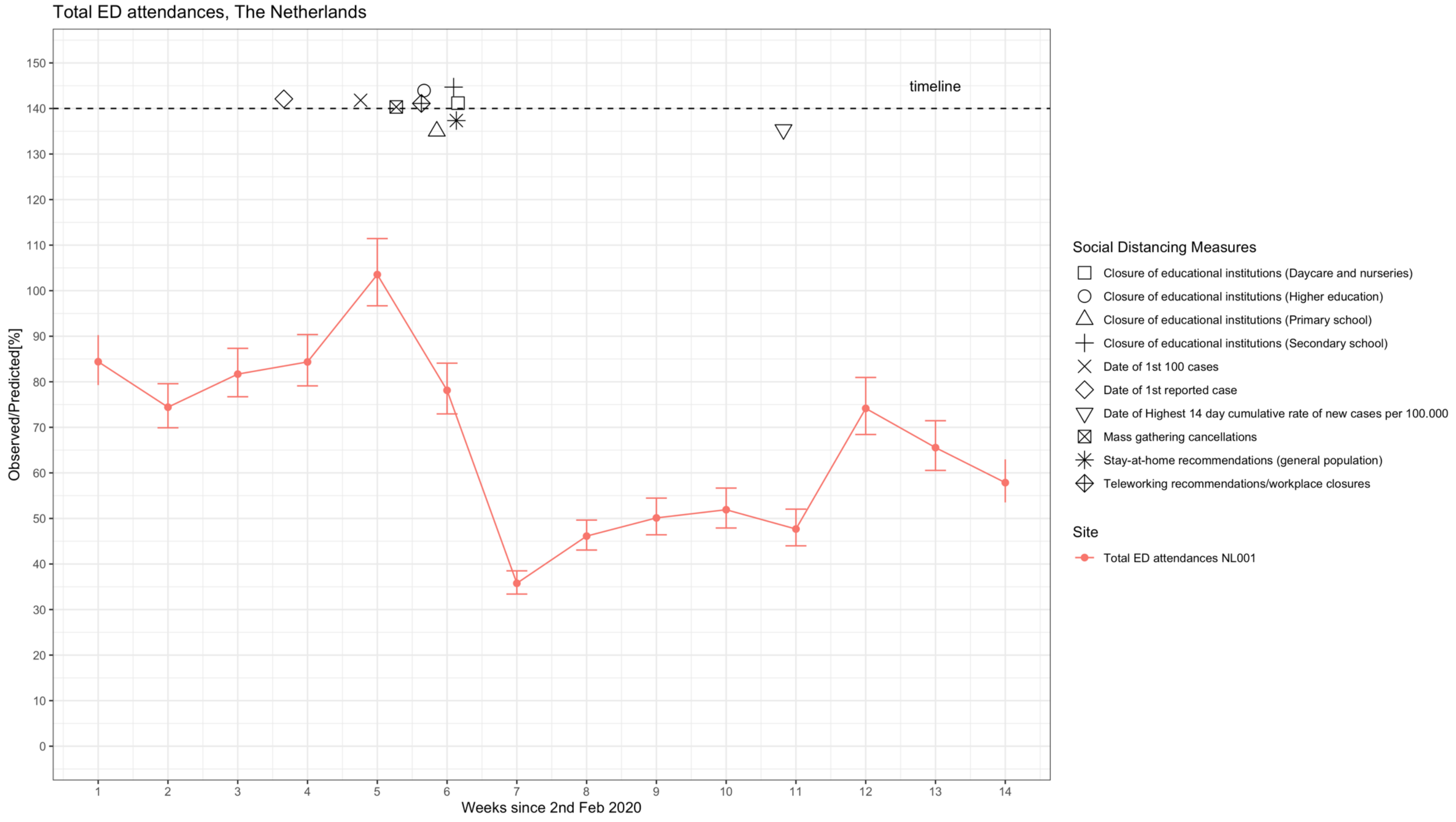

Slovenia

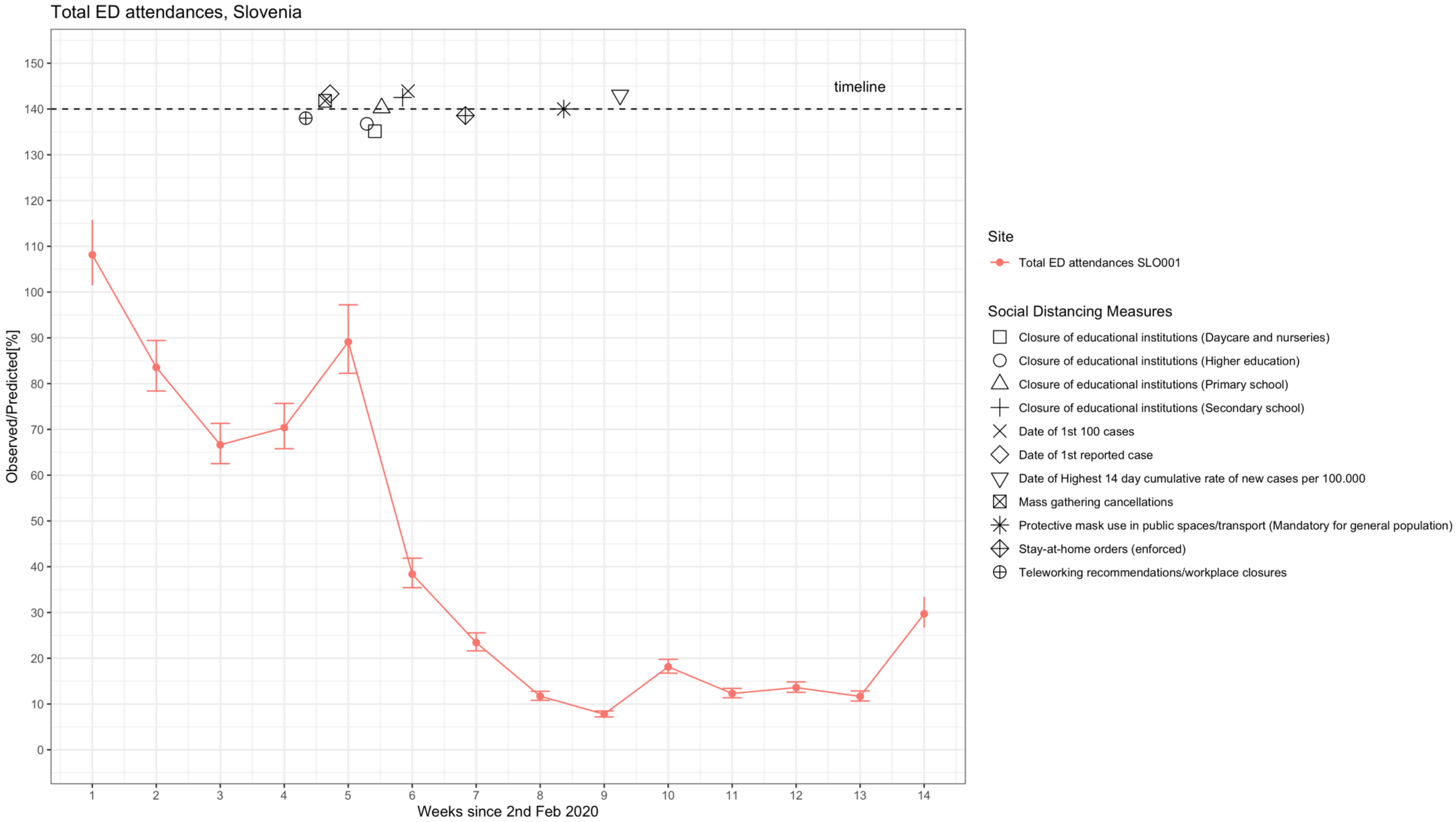

Total ED attendances, Slovenia
timeline
Observed/Predicted[%]
150
140
130
120
110
100
90
80
70
60
50
40
30
20
10
0
1
2
3
4
5
6
7
8
9
10
11
12
13
14
Weeks since 2nd Feb 2020
Site
Total ED attendances SLO001
Social Distancing Measures
Closure of educational institutions (Daycare and nurseries)
Closure of educational institutions (Higher education)
Closure of educational institutions (Primary school)
Closure of educational institutions (Secondary school)
Date of 1st 100 cases
Date of 1st reported case
Date of Highest 14 day cumulative rate of new cases per 100.000
Mass gathering cancellations
Protective mask use in public spaces/transport (Mandatory for general population)
Stay-at-home orders (enforced)
Teleworking recommendations/workplace closures
