# Supplementary material for: Presentations of children to emergency departments across Europe and the COVID-19 pandemic: A multinational observational study
Source: PLoS Med. 2022 Aug 26;19(8):e1003974. doi: 10.1371/journal.pmed.1003974 (PMC9467376; doi:10.1371/journal.pmed.1003974)
Supplement: S9 Fig — Percentages of total ED attendances (left) and absolute numbers (right) of children admitted to hospital (top) and PICUs (bottom); comparing the 28-day standardized numbers for the months of January–April for 2018 vs. 2019 vs. 2020. ED, emergency department; PICU, pediatric intensive care unit. (PDF) [file pmed.1003974.s021.pdf]

S9 Fig. Percentage of children admitted to hospital for individual sites

Legend:

Percentages of total ED attendances (left) and absolute numbers (right) of children admitted to hospital (top) and pediatric intensive care units (bottom); comparing the 28-day standardized numbers for the months of January – April for 2018 vs. 2019 vs. 2020.

AUS001

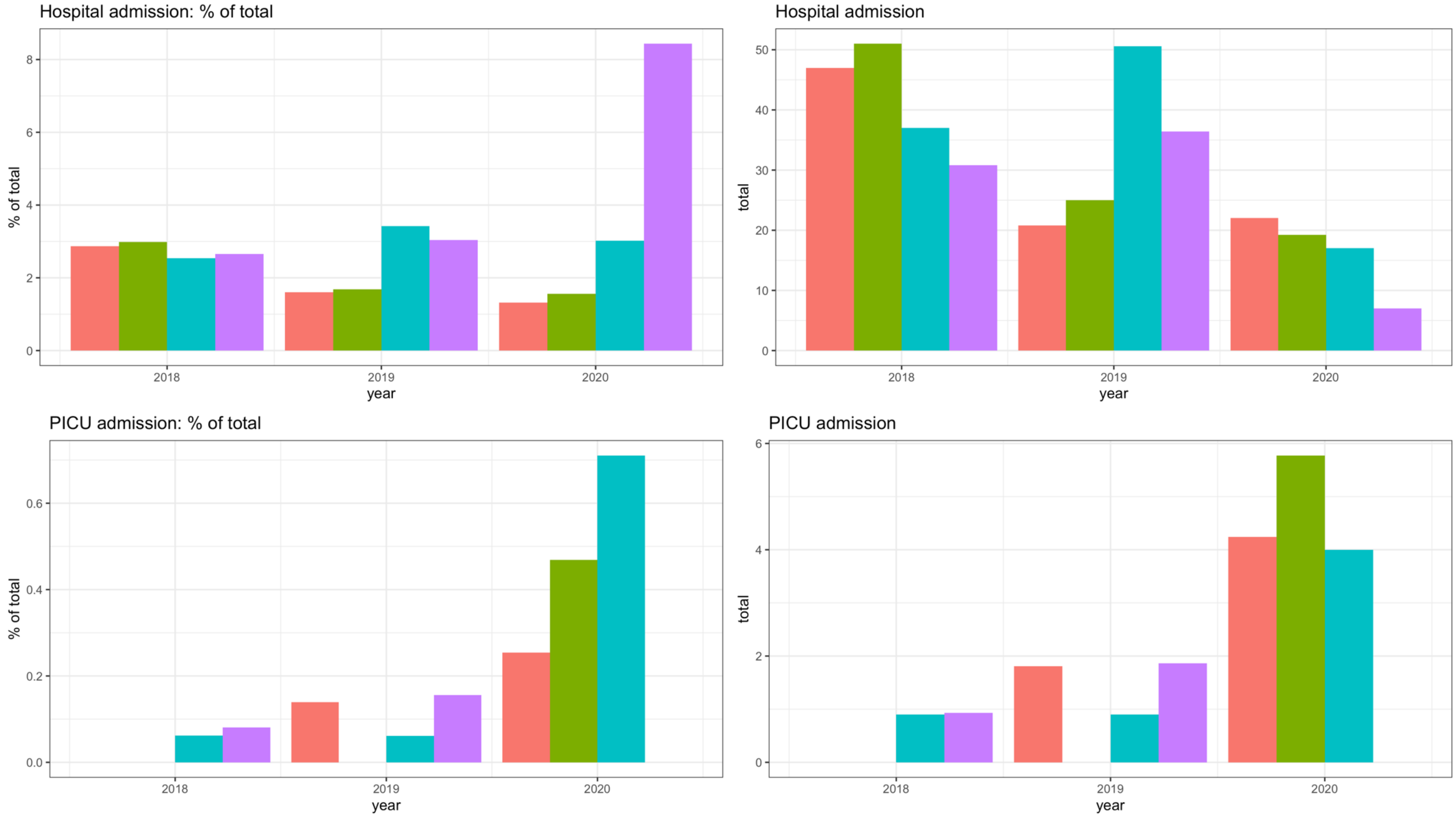

Hospital admission: % of total
% of total
0
2
4
6
8
2018
2019
2020
year
Hospital admission
total
0
10
20
30
40
50
2018
2019
2020
year
PICU admission: % of total
% of total
0.0
0.2
0.4
0.6
2018
2019
2020
year
PICU admission
total
0
2
4
6
2018
2019
2020
year

AUS003

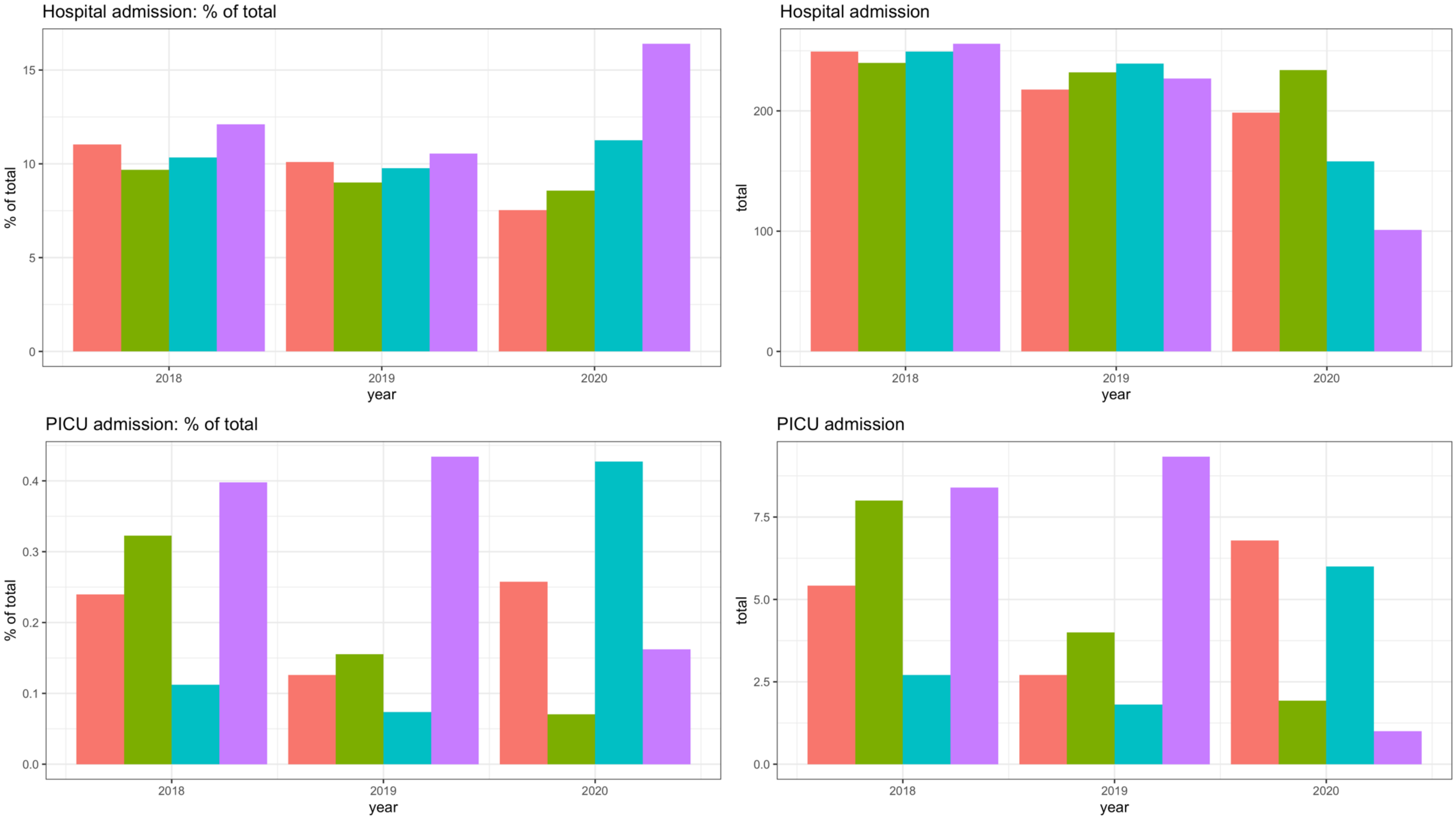

Hospital admission: % of total
% of total
0
5
10
15
2018
2019
2020
year
Hospital admission
total
0
100
200
2018
2019
2020
year
PICU admission: % of total
% of total
0.0
0.1
0.2
0.3
0.4
2018
2019
2020
year
PICU admission
total
0.0
2.5
5.0
7.5
2018
2019
2020
year

AUS004

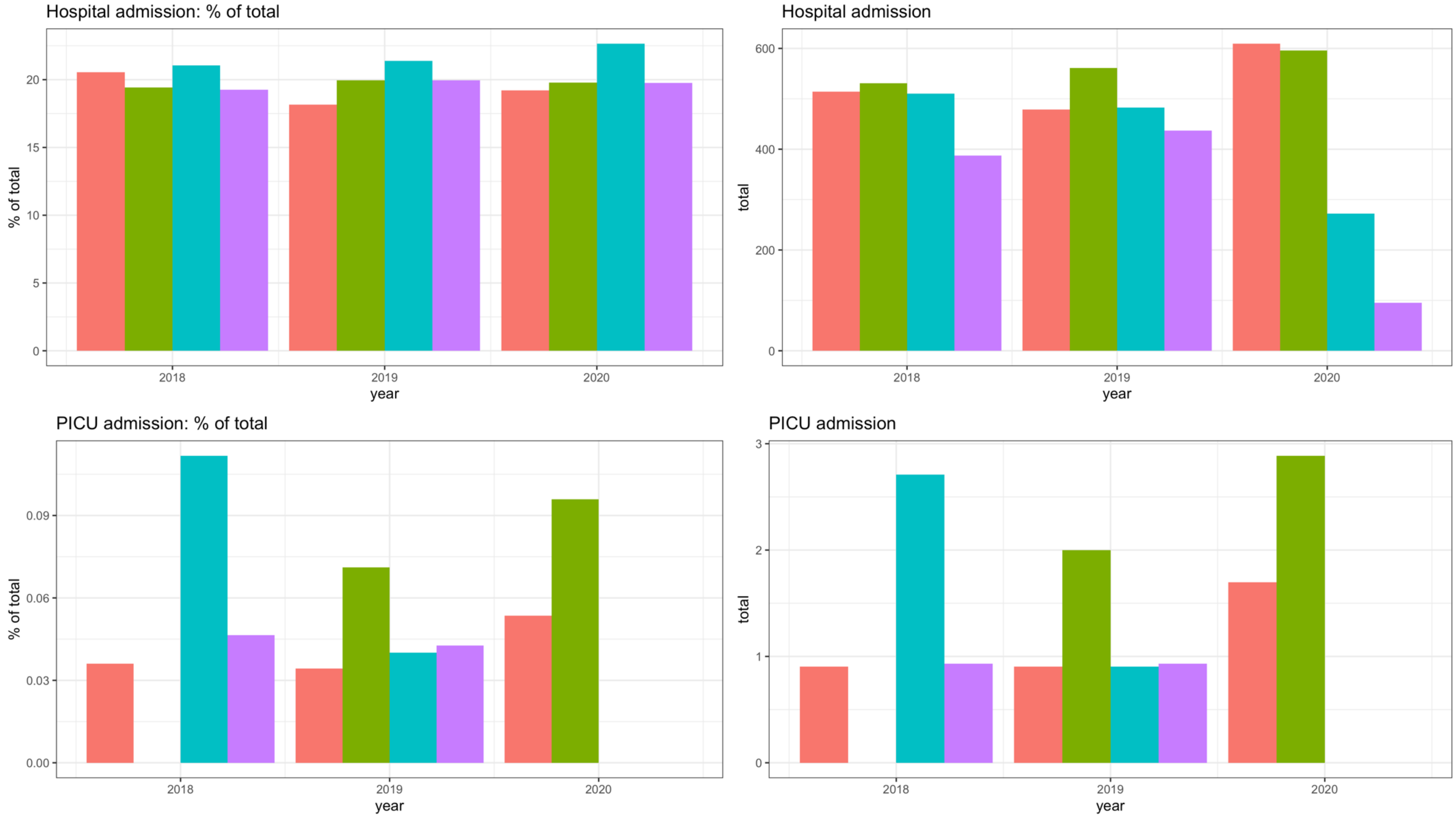

Hospital admission: % of total
% of total
0
5
10
15
20
2018
2019
2020
year
Hospital admission
total
0
200
400
600
2018
2019
2020
year
PICU admission: % of total
% of total
0.00
0.03
0.06
0.09
2018
2019
2020
year
PICU admission
total
0
1
2
3
2018
2019
2020
year

FR001

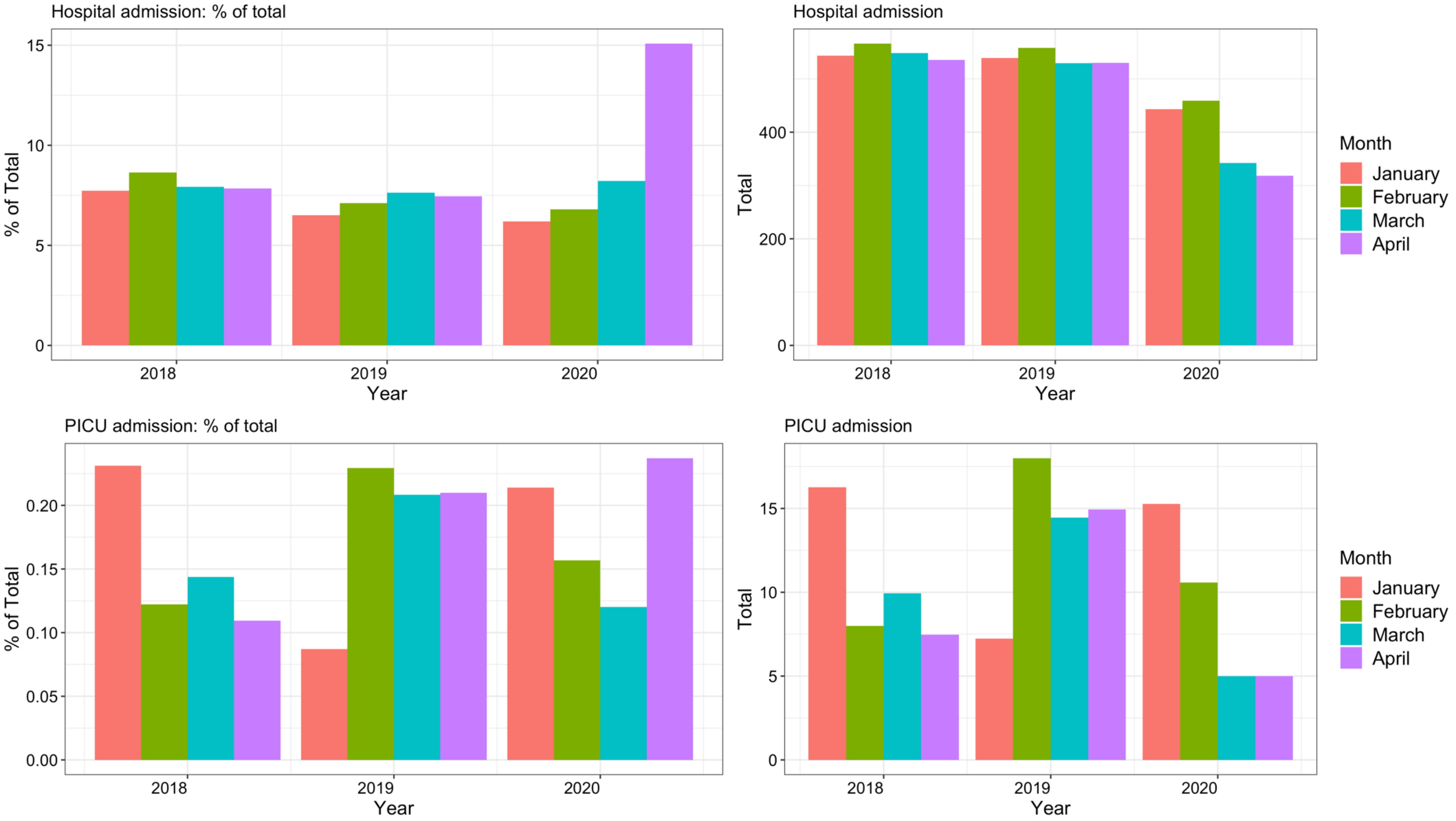

Hospital admission: % of total
% of Total
0
5
10
15
2018
2019
2020
Year
Hospital admission
Total
0
200
400
2018
2019
2020
Year
Month
January
February
March
April
PICU admission: % of total
% of Total
0.00
0.05
0.10
0.15
0.20
2018
2019
2020
Year
PICU admission
Total
0
5
10
15
2018
2019
2020
Year
Month
January
February
March
April

FR002

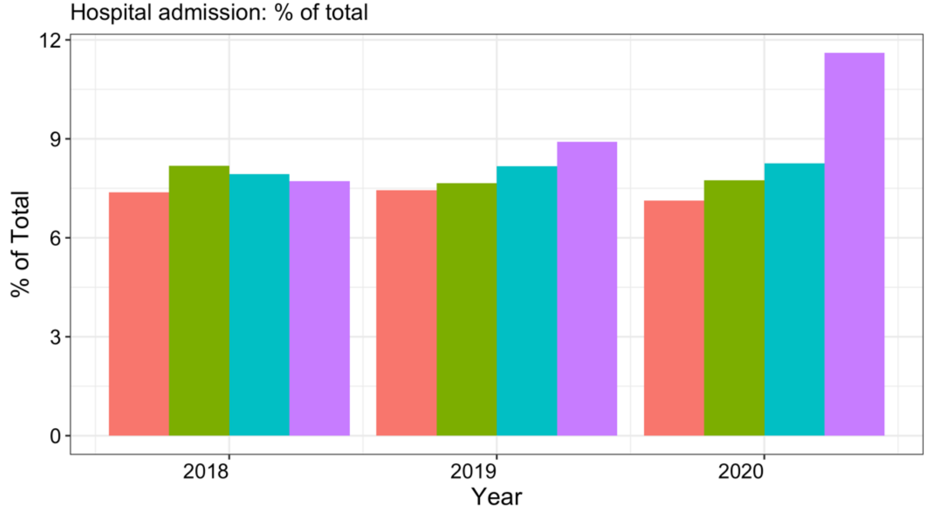

Hospital admission: % of total
% of Total
0
3
6
9
12
2018
2019
2020
Year

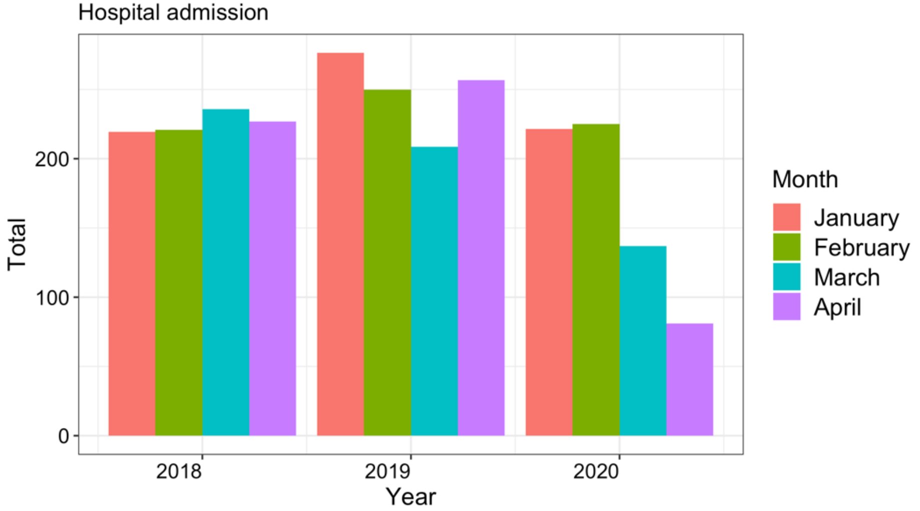

Hospital admission
Total
0
100
200
2018
2019
2020
Year
Month
January
February
March
April

FR003

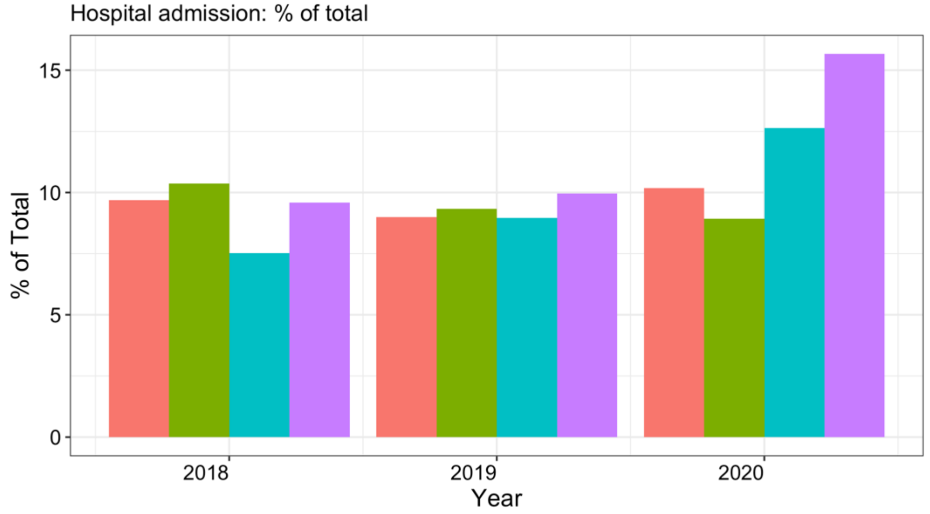

Hospital admission: % of total
% of Total
0
5
10
15
2018
2019
2020
Year

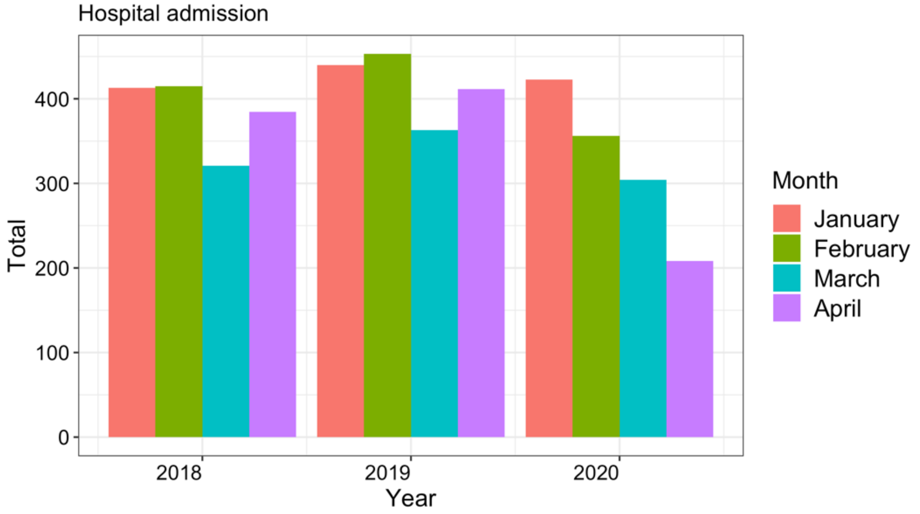

Hospital admission
Total
0
100
200
300
400
2018
2019
2020
Year
Month
January
February
March
April

FR004

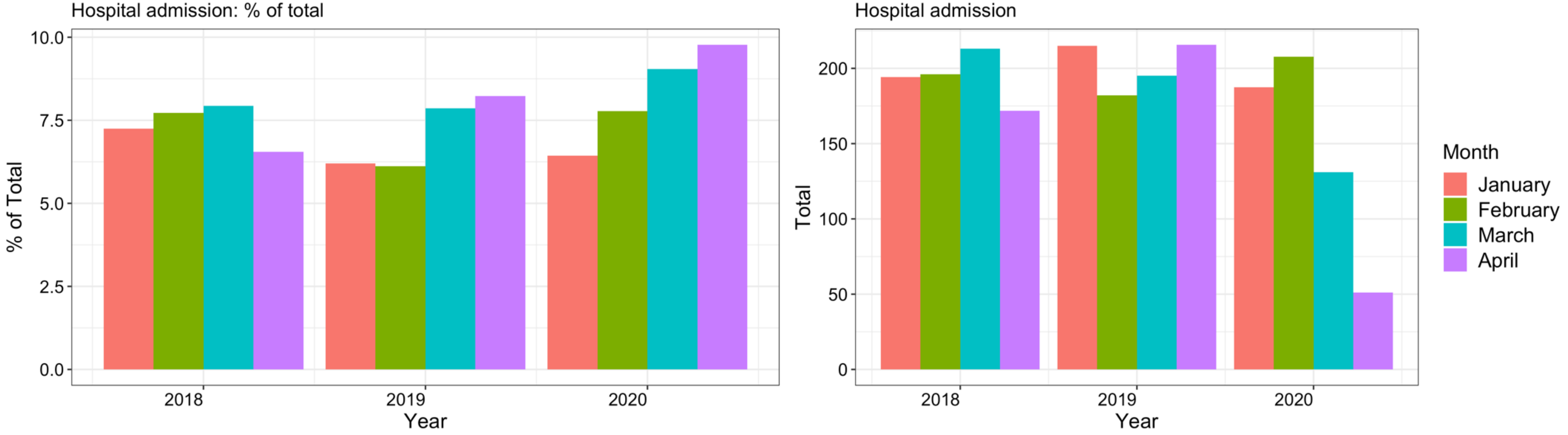

Hospital admission: % of total
10.0
7.5
5.0
2.5
0.0
% of Total
2018
2019
2020
Year
Hospital admission
200
150
100
50
0
Total
2018
2019
2020
Year
Month
January
February
March
April

GER001

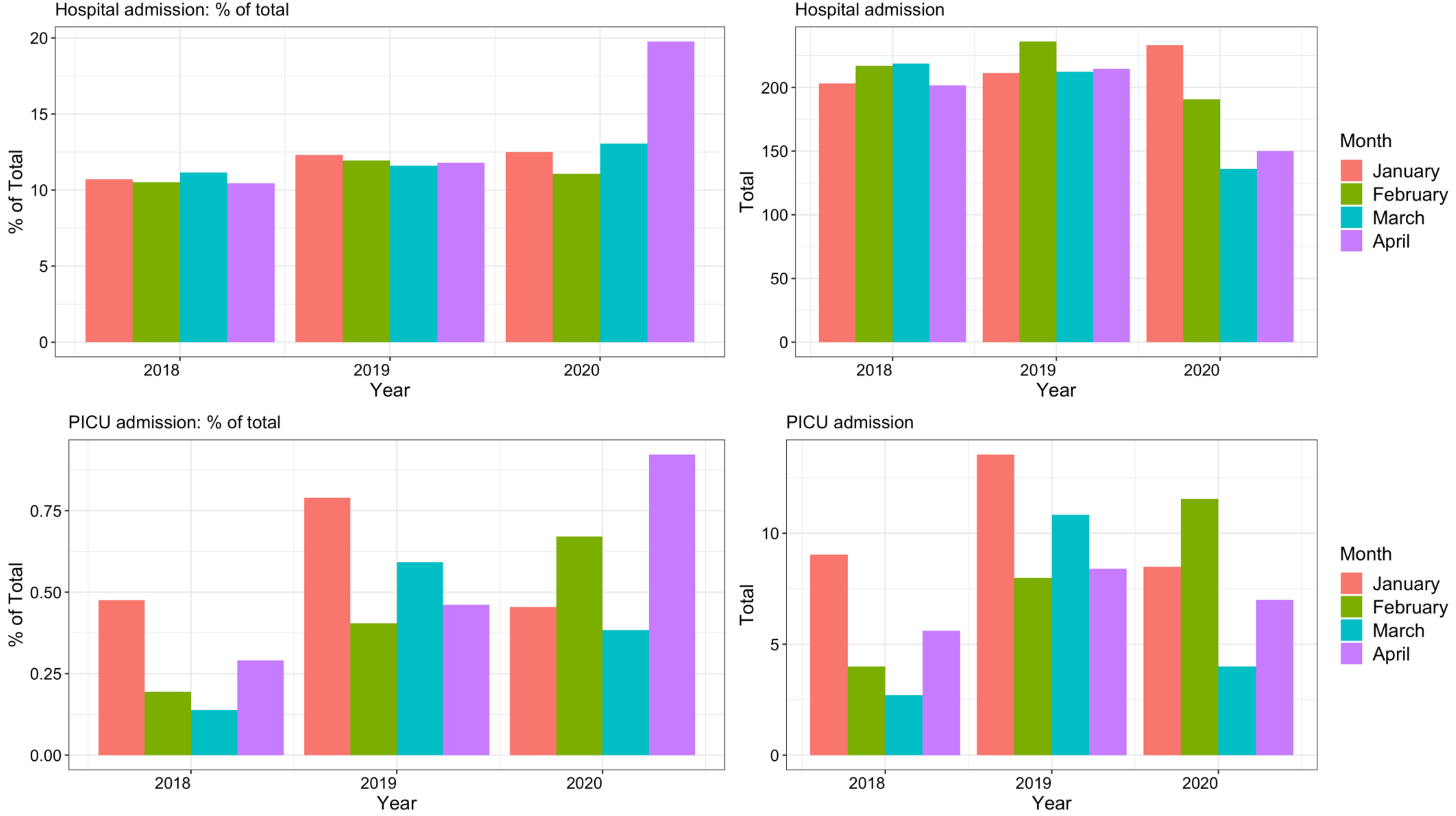

Hospital admission: % of total
% of Total
0
5
10
15
20
2018
2019
2020
Year
Hospital admission
Total
0
50
100
150
200
2018
2019
2020
Year
Month
January
February
March
April
PICU admission: % of total
% of Total
0.00
0.25
0.50
0.75
2018
2019
2020
Year
PICU admission
Total
0
5
10
2018
2019
2020
Year
Month
January
February
March
April

HUN001

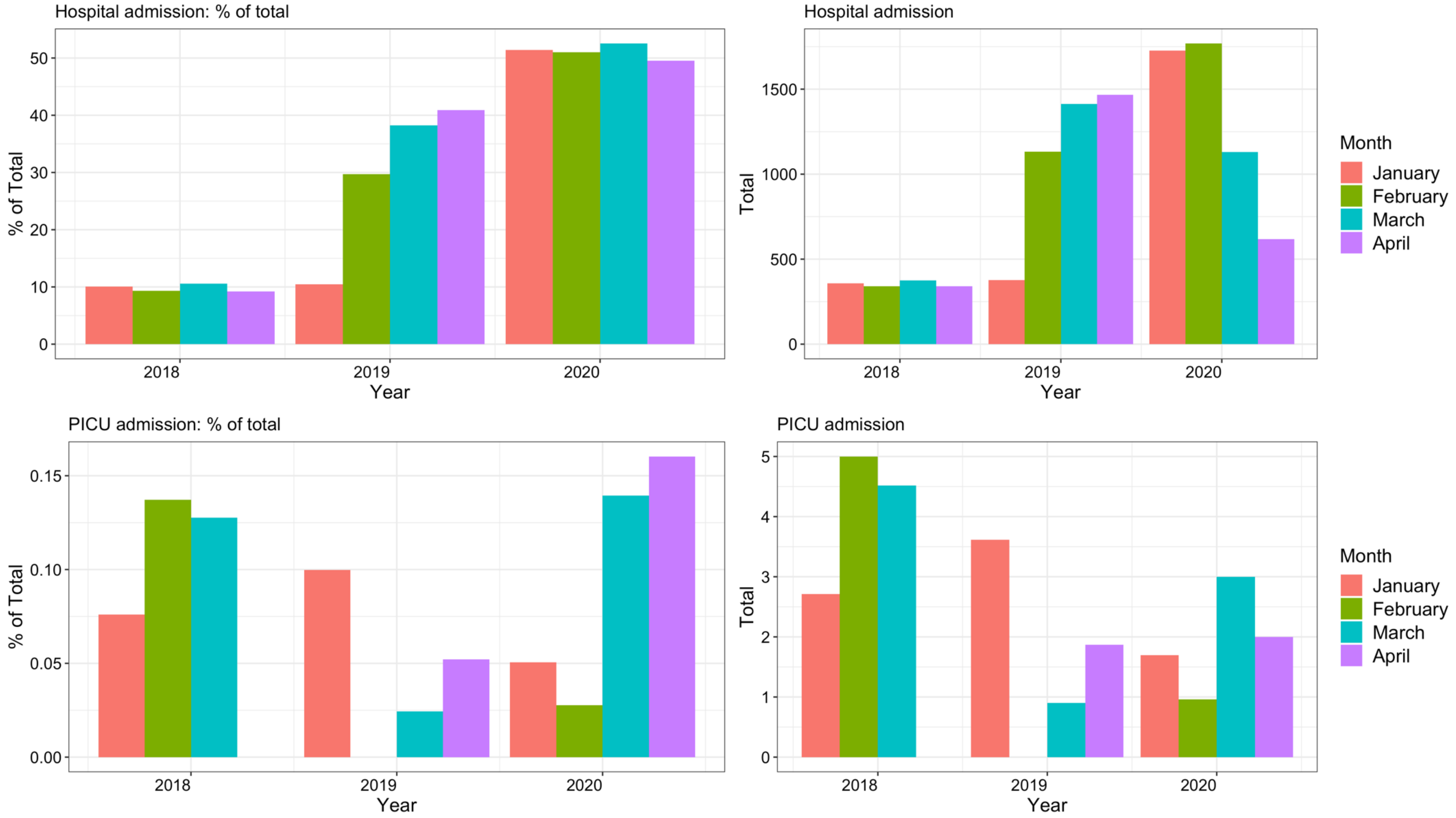

Hospital admission: % of total
% of Total
Year
2018
2019
2020
0
10
20
30
40
50
Hospital admission
Total
0
500
1000
1500
PICU admission: % of total
0.00
0.05
0.10
0.15
PICU admission
0
1
2
3
4
5
Month
January
February
March
April

HUN002

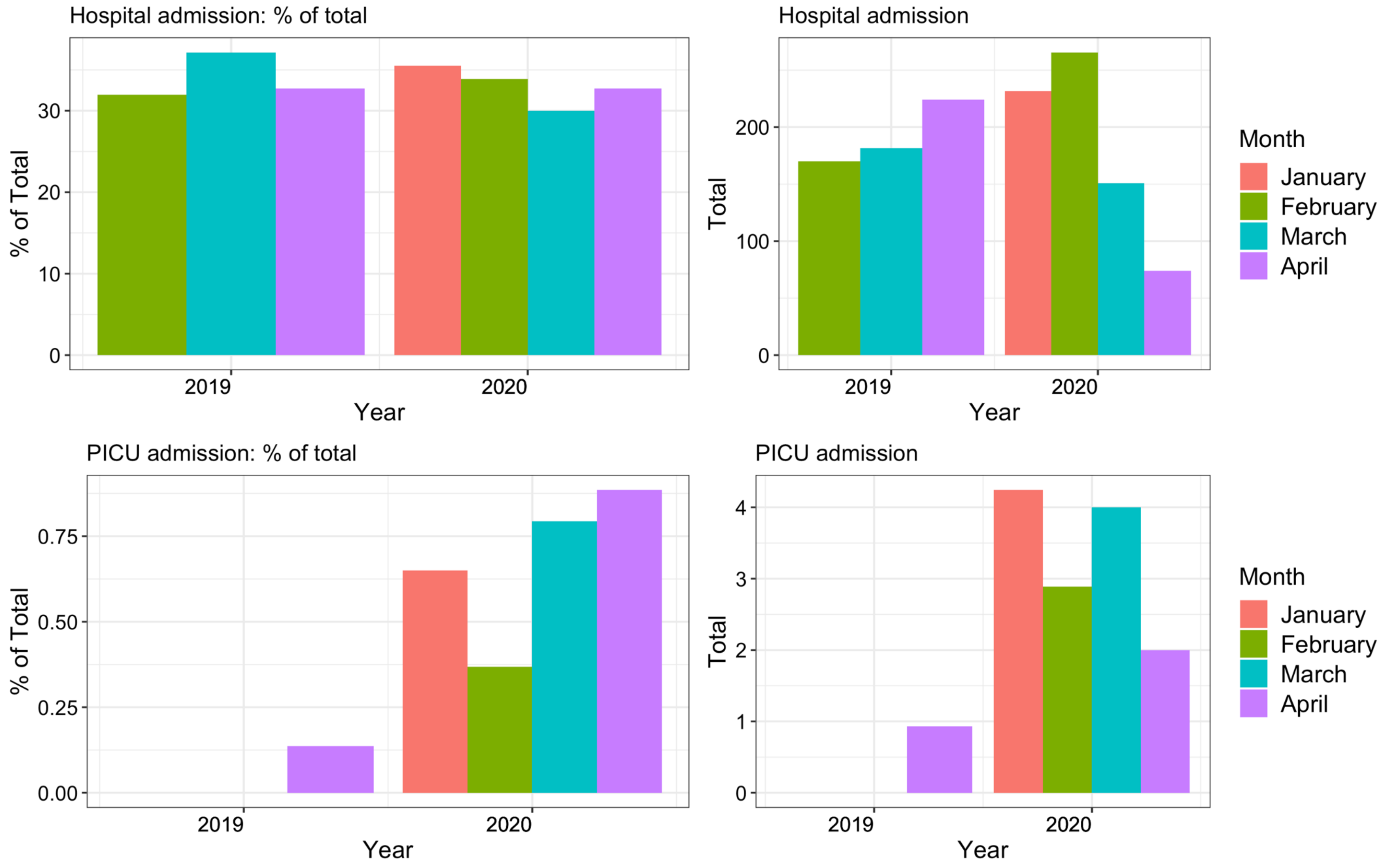

Hospital admission: % of total
% of Total
0
10
20
30
2019
2020
Year
Hospital admission
Total
0
100
200
2019
2020
Year
Month
January
February
March
April
PICU admission: % of total
% of Total
0.00
0.25
0.50
0.75
2019
2020
Year
PICU admission
Total
0
1
2
3
4
2019
2020
Year
Month
January
February
March
April

ICE001

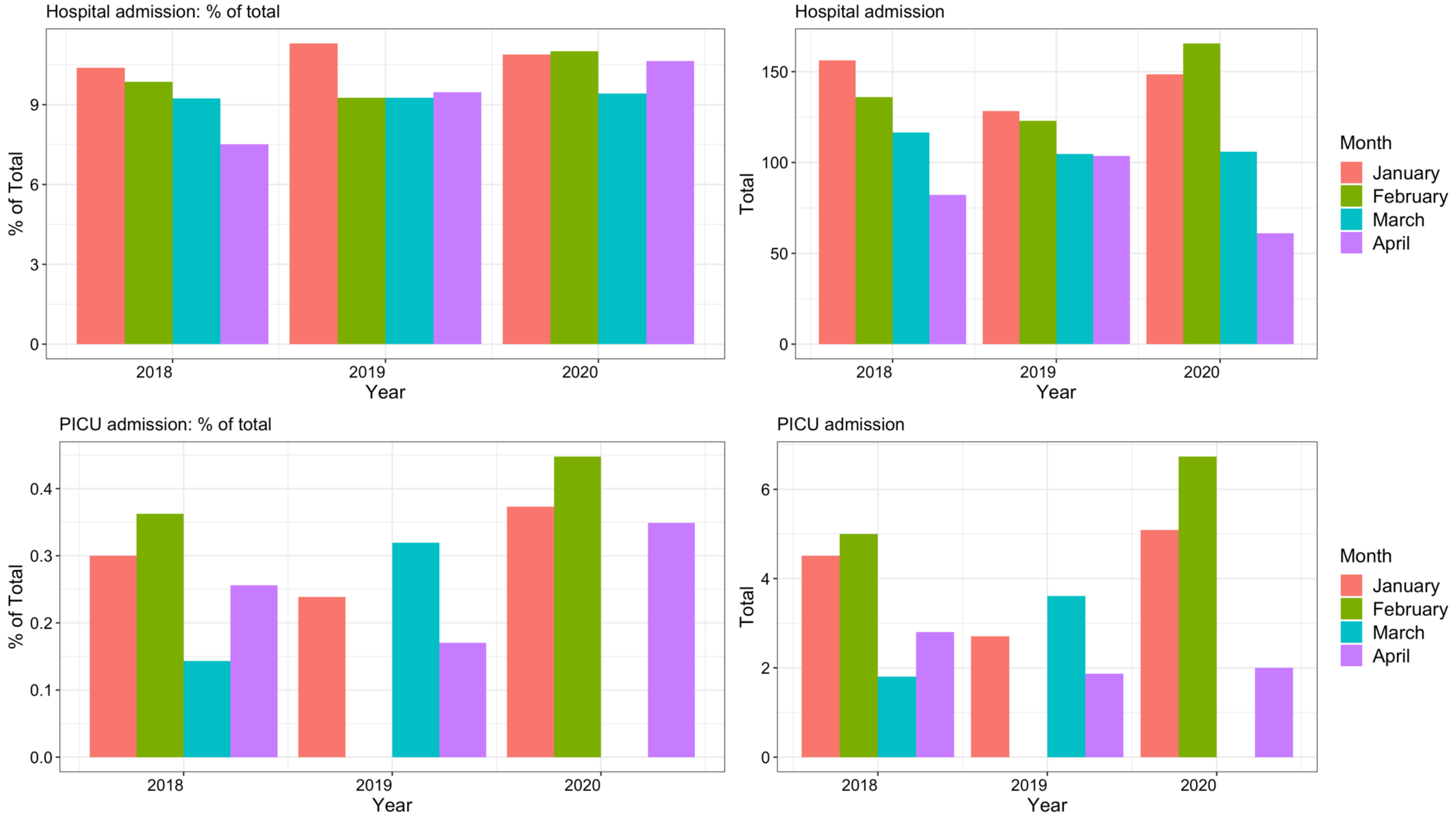

Hospital admission: % of total
% of Total
Year
2018
2019
2020
Hospital admission
Total
Month
January
February
March
April
PICU admission: % of total
PICU admission

IRE001

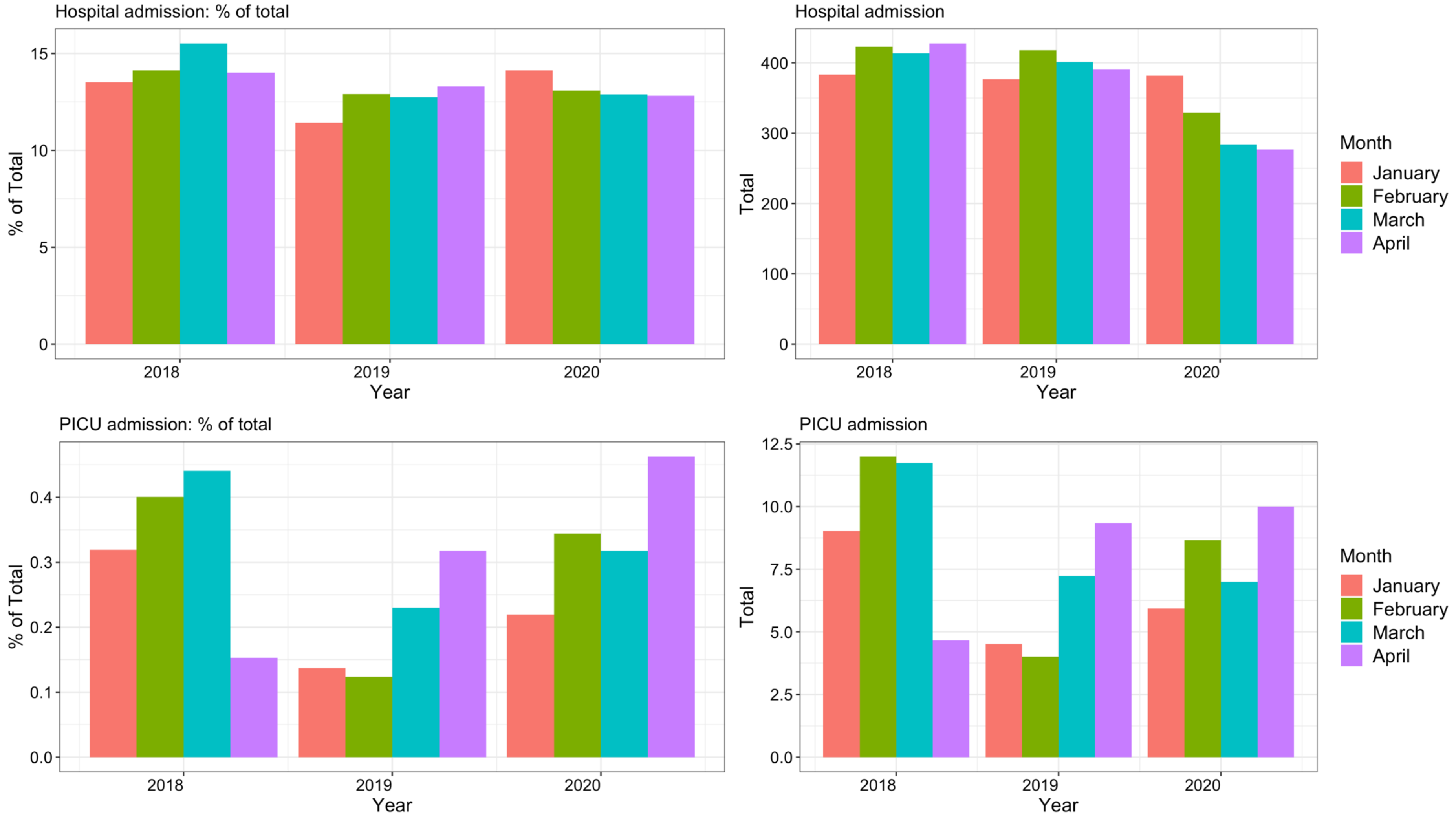
Hospital admission: % of total
% of Total
0
5
10
15
2018
2019
2020
Year
Hospital admission
Total
0
100
200
300
400
2018
2019
2020
Year
Month
January
February
March
April
PICU admission: % of total
% of Total
0.0
0.1
0.2
0.3
0.4
2018
2019
2020
Year
PICU admission
Total
0.0
2.5
5.0
7.5
10.0
12.5
2018
2019
2020
Year
Month
January
February
March
April

IRE002

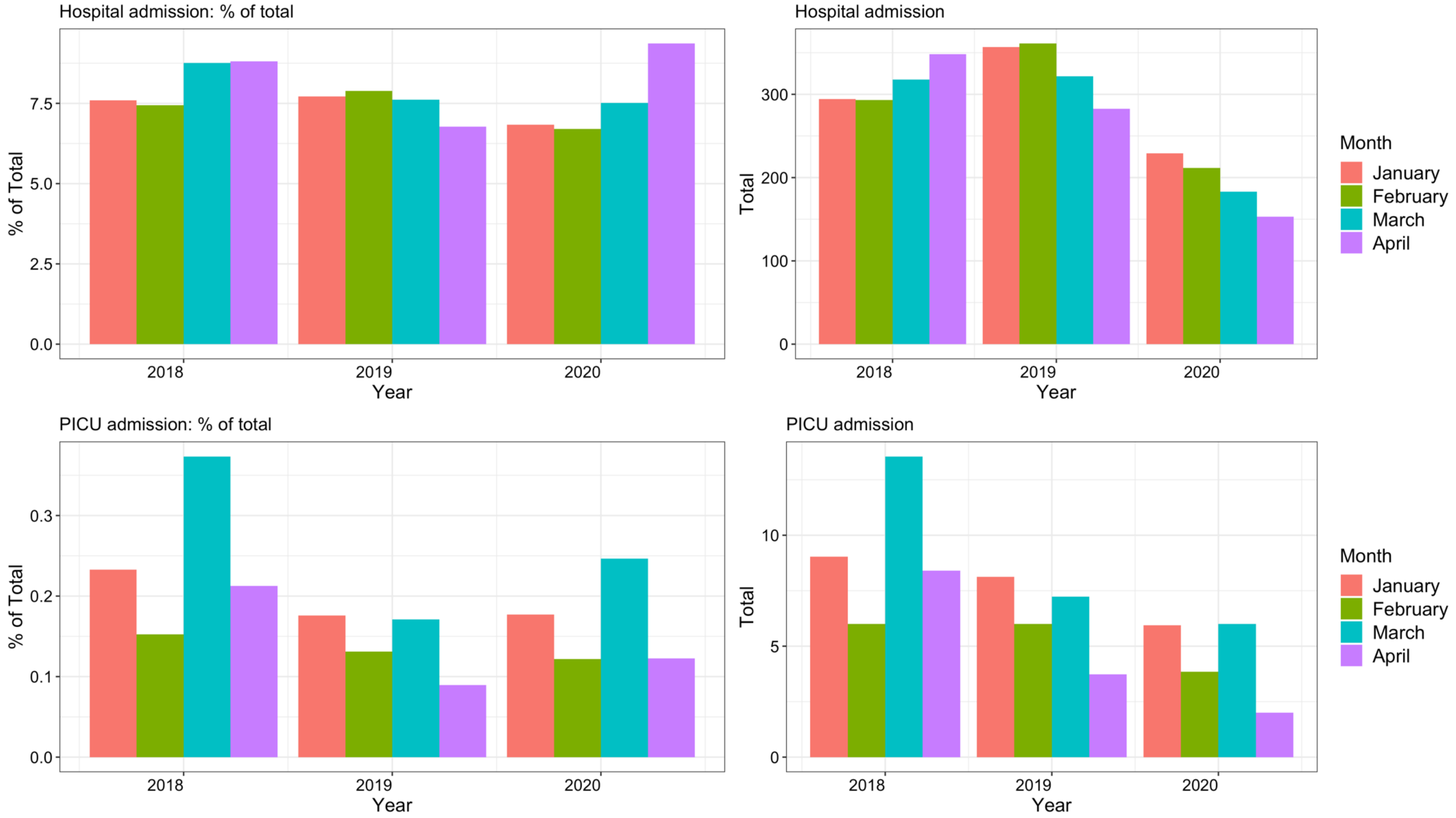
Hospital admission: % of total
% of Total
0.0
2.5
5.0
7.5
2018
2019
2020
Year
Hospital admission
Total
0
100
200
300
2018
2019
2020
Year
Month
January
February
March
April
PICU admission: % of total
% of Total
0.0
0.1
0.2
0.3
2018
2019
2020
Year
PICU admission
Total
0
5
10
2018
2019
2020
Year
Month
January
February
March
April

IRE003

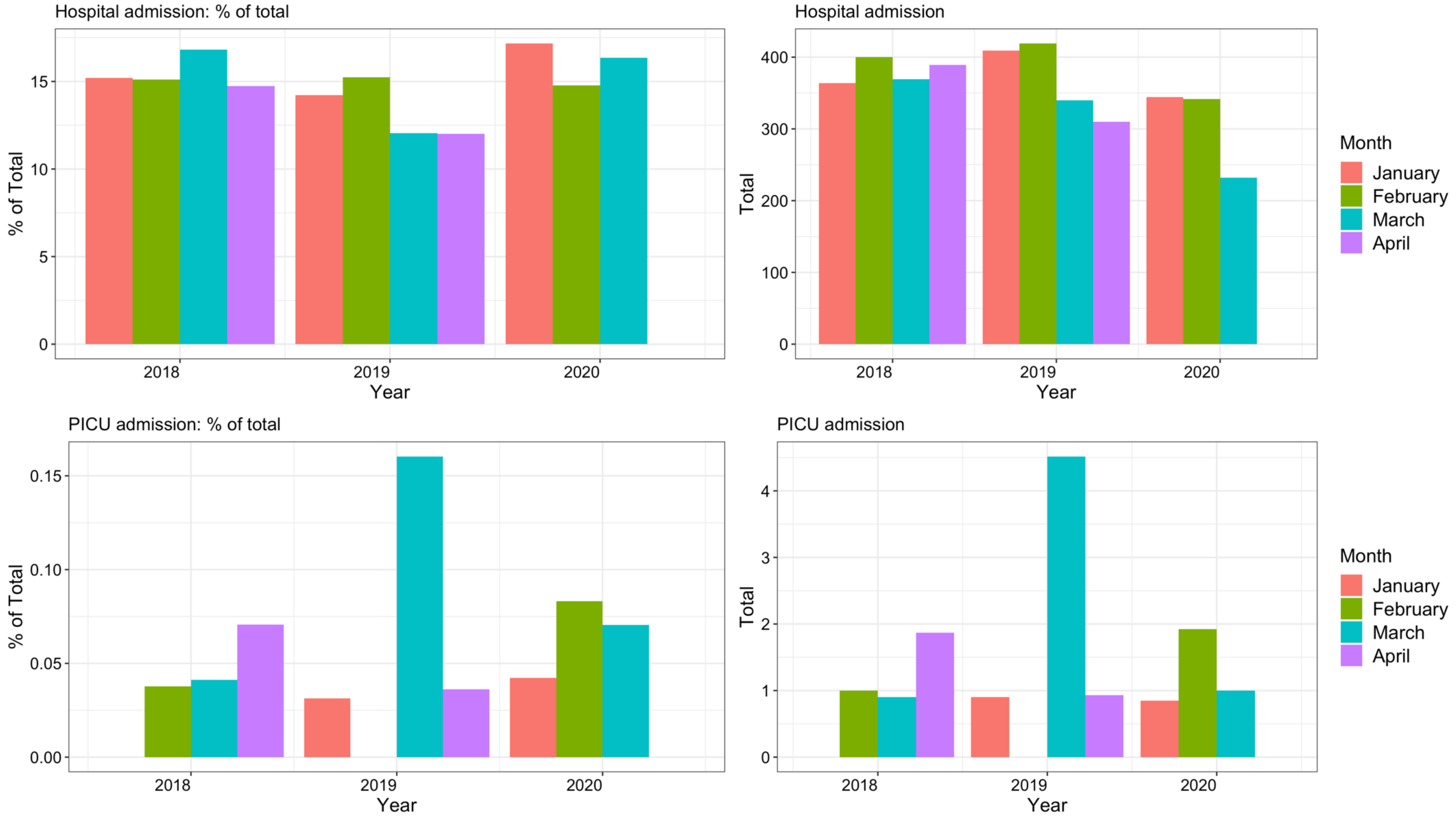
Hospital admission: % of total
% of Total
0
5
10
15
2018
2019
2020
Year
Hospital admission
Total
0
100
200
300
400
2018
2019
2020
Year
Month
January
February
March
April
PICU admission: % of total
% of Total
0.00
0.05
0.10
0.15
2018
2019
2020
Year
PICU admission
Total
0
1
2
3
4
2018
2019
2020
Year
Month
January
February
March
April

IT001

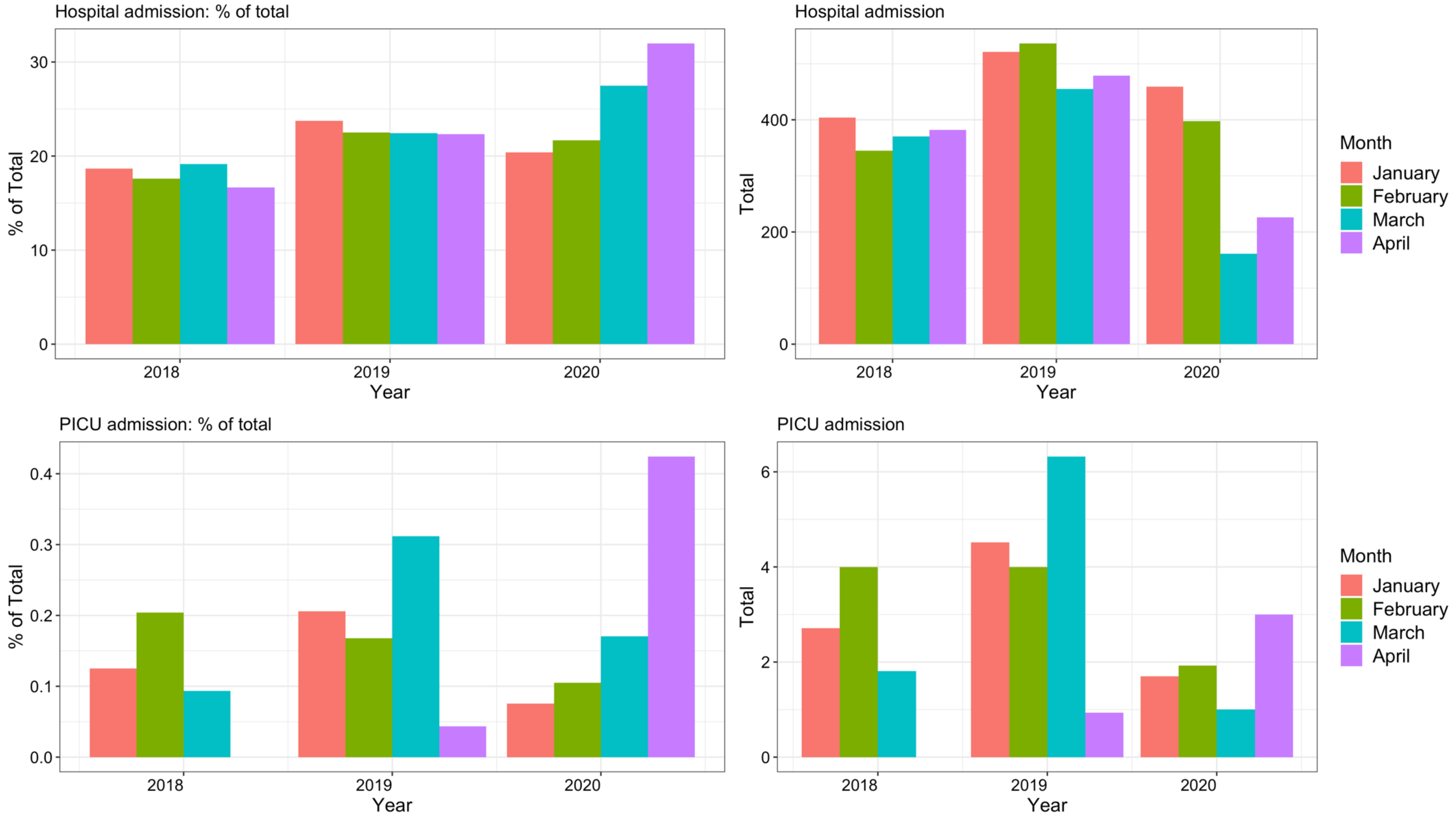

Hospital admission: % of total
% of Total
Year
2018
2019
2020
Hospital admission
Total
Month
January
February
March
April
PICU admission: % of total
PICU admission

IT002

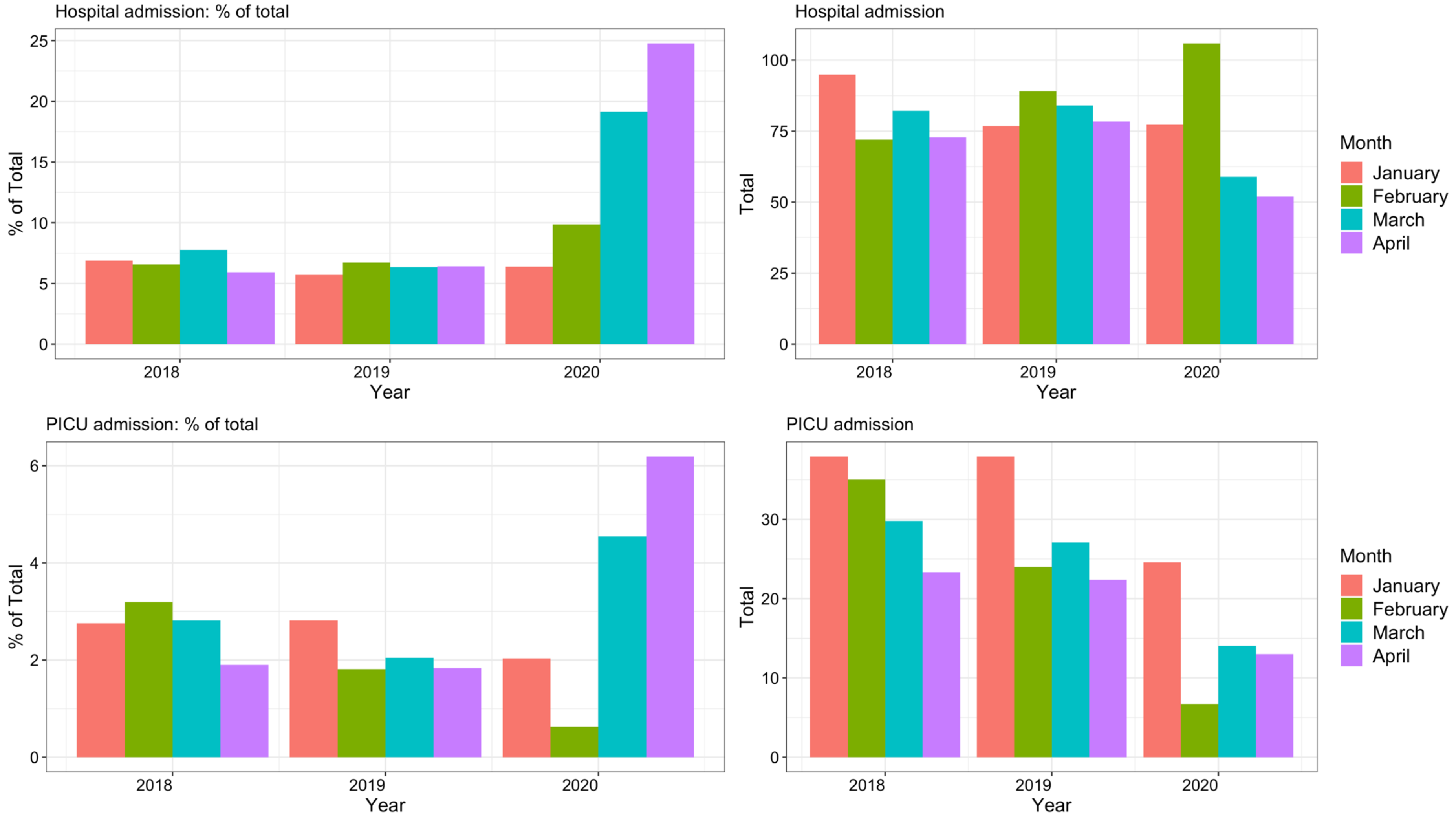

Hospital admission: % of total
% of Total
0
5
10
15
20
25
2018
2019
2020
Year
Hospital admission
Total
0
25
50
75
100
2018
2019
2020
Year
Month
January
February
March
April
PICU admission: % of total
% of Total
0
2
4
6
2018
2019
2020
Year
PICU admission
Total
0
10
20
30
2018
2019
2020
Year
Month
January
February
March
April

LAT001

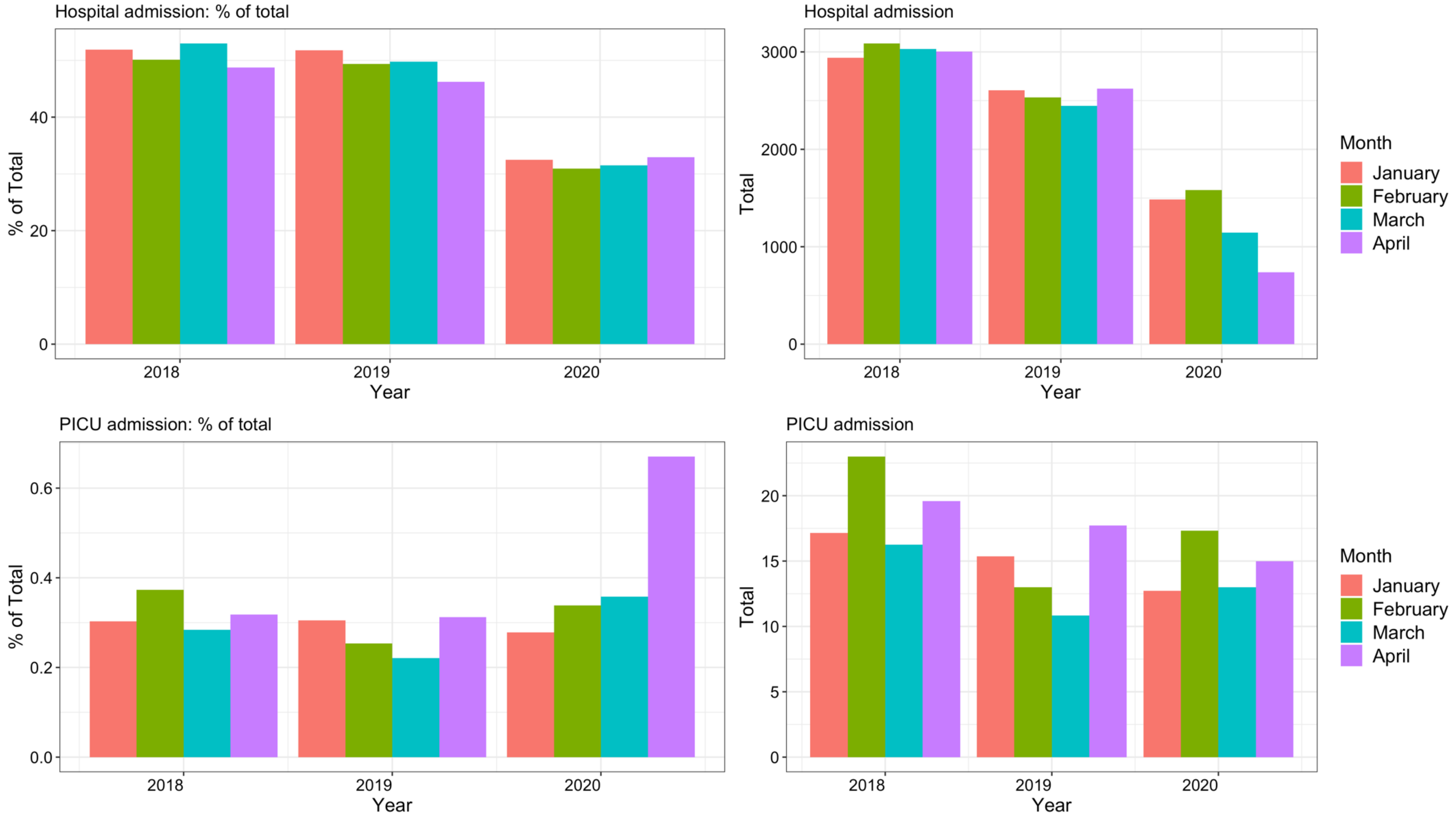
Hospital admission: % of total
% of Total
0
20
40
2018
2019
2020
Year
Hospital admission
Total
0
1000
2000
3000
2018
2019
2020
Year
Month
January
February
March
April
PICU admission: % of total
% of Total
0.0
0.2
0.4
0.6
2018
2019
2020
Year
PICU admission
Total
0
5
10
15
20
2018
2019
2020
Year
Month
January
February
March
April

LIT001

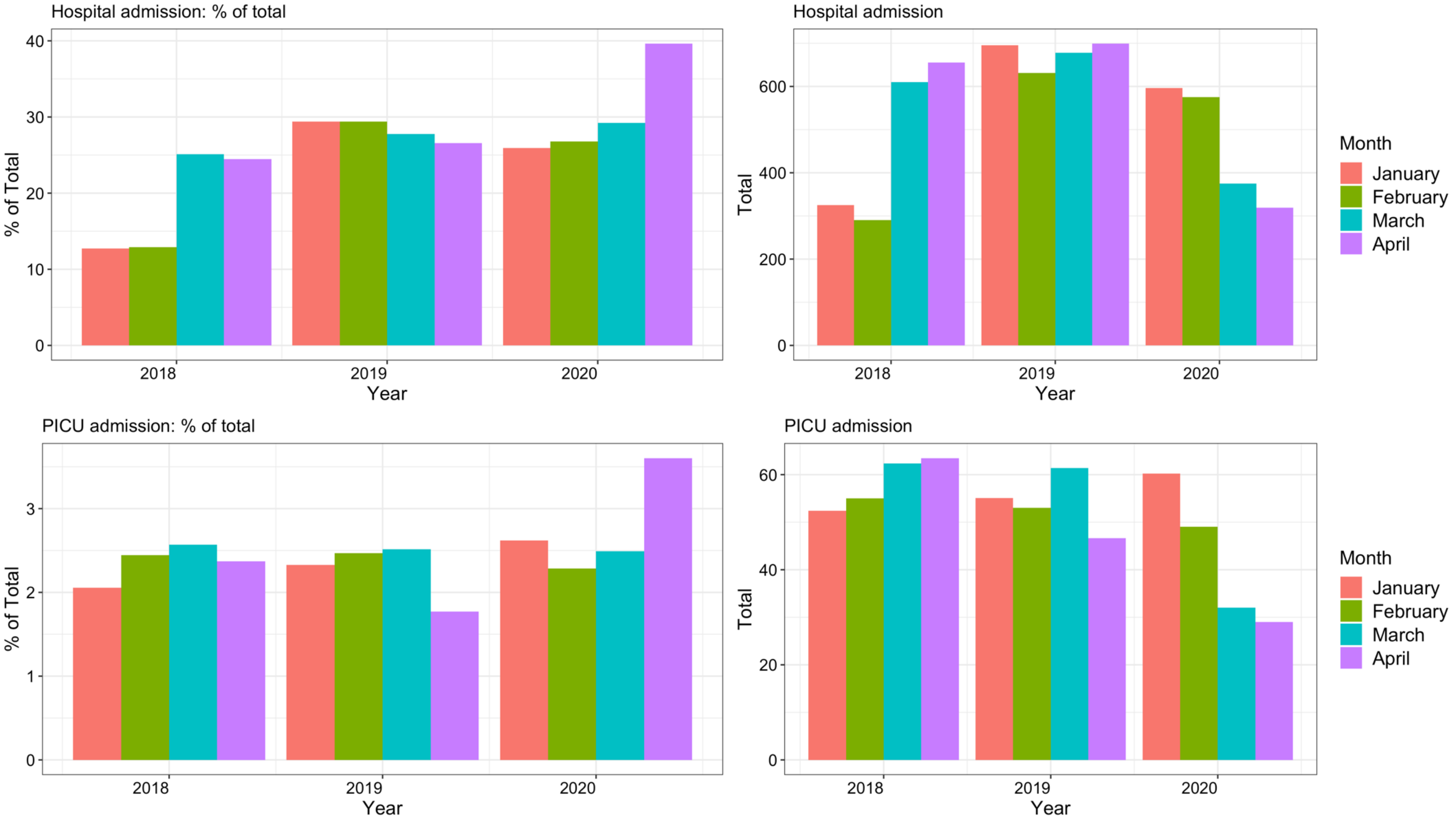

Hospital admission: % of total
% of Total
0
10
20
30
40
2018
2019
2020
Year
Hospital admission
Total
0
200
400
600
2018
2019
2020
Year
Month
January
February
March
April
PICU admission: % of total
% of Total
0
1
2
3
2018
2019
2020
Year
PICU admission
Total
0
20
40
60
2018
2019
2020
Year
Month
January
February
March
April

MAL001

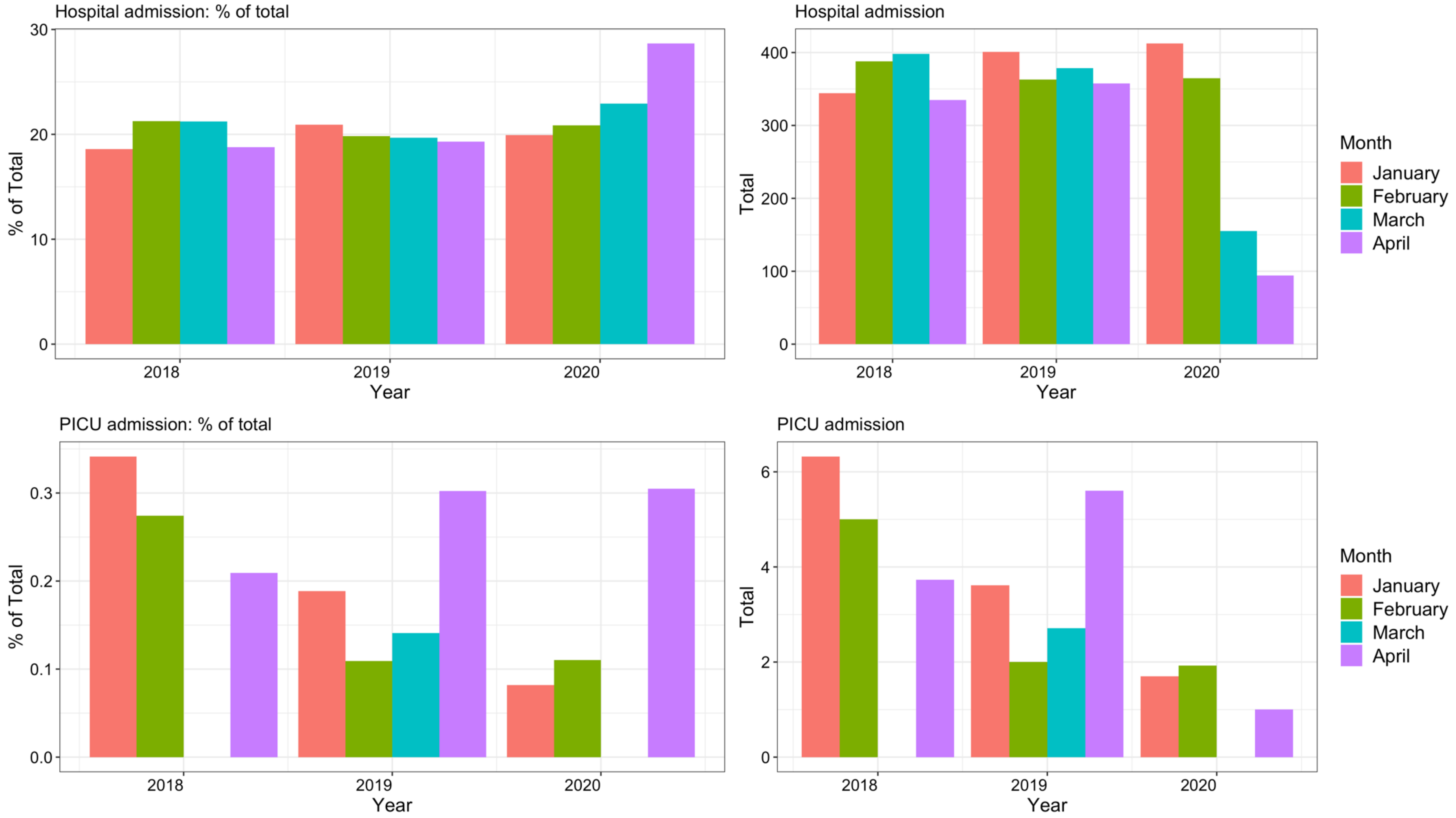

Hospital admission: % of total
% of Total
30
20
10
0
2018
2019
2020
Year
Hospital admission
Total
400
300
200
100
0
2018
2019
2020
Year
Month
January
February
March
April
PICU admission: % of total
% of Total
0.3
0.2
0.1
0.0
2018
2019
2020
Year
PICU admission
Total
6
4
2
0
2018
2019
2020
Year
Month
January
February
March
April

NL001

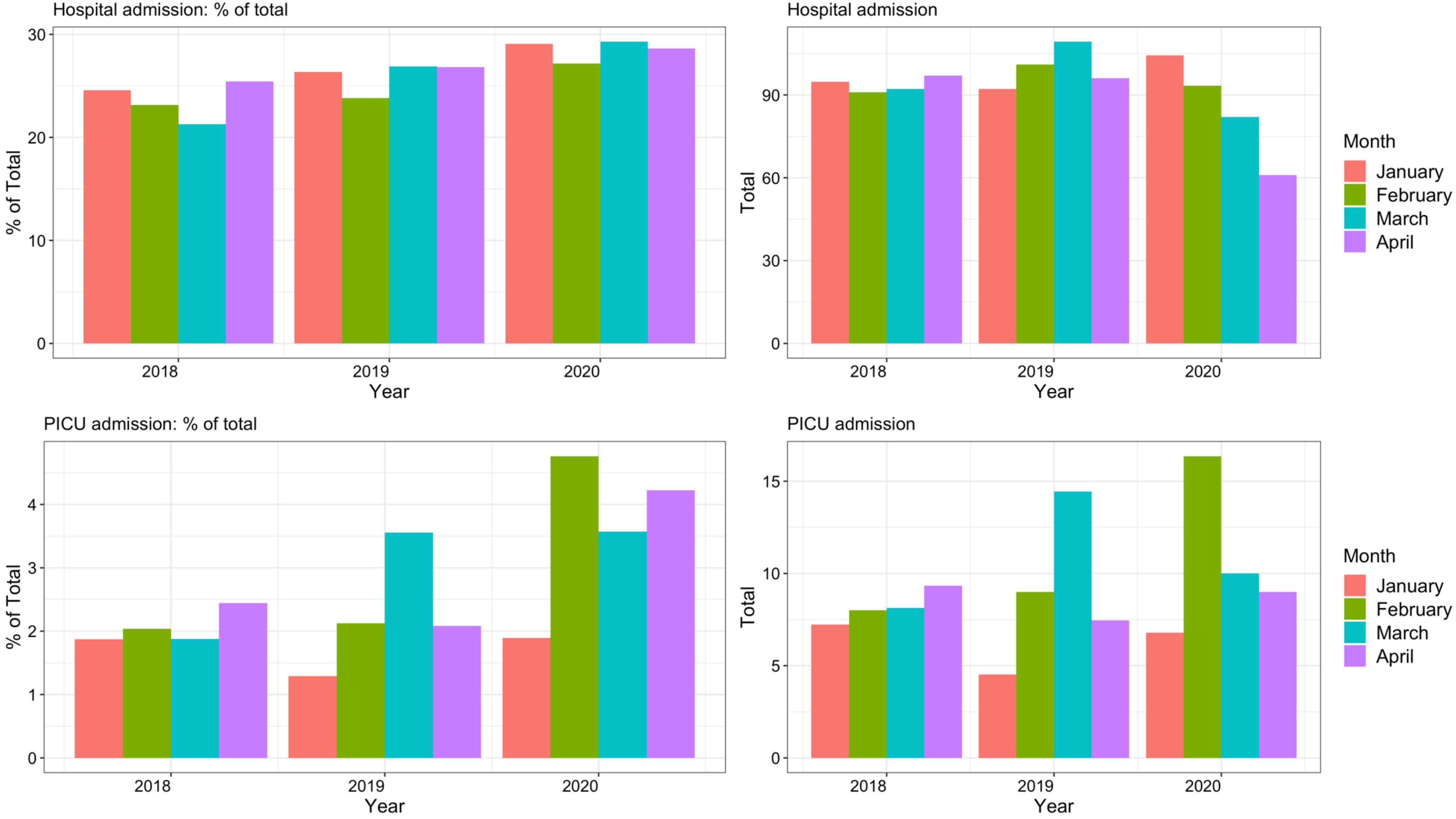

Hospital admission: % of total
% of Total
Year
2018
2019
2020
Hospital admission
Total
PICU admission: % of total
PICU admission
Month
January
February
March
April

NL002

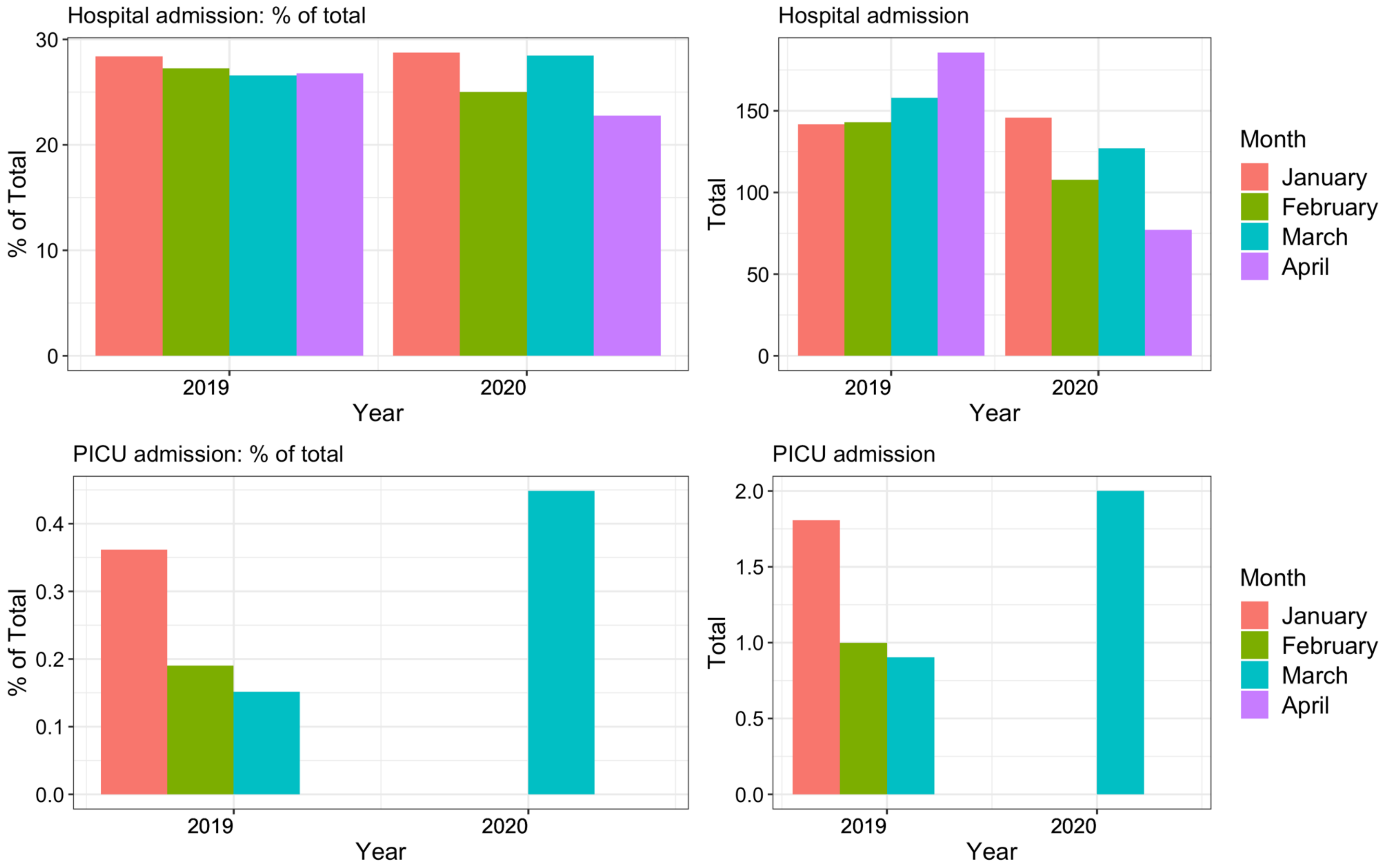

Hospital admission: % of total
% of Total
30
20
10
0
2019
2020
Year
Hospital admission
Total
150
100
50
0
2019
2020
Year
Month
January
February
March
April
PICU admission: % of total
% of Total
0.4
0.3
0.2
0.1
0.0
2019
2020
Year
PICU admission
Total
2.0
1.5
1.0
0.5
0.0
2019
2020
Year
Month
January
February
March
April

POR001

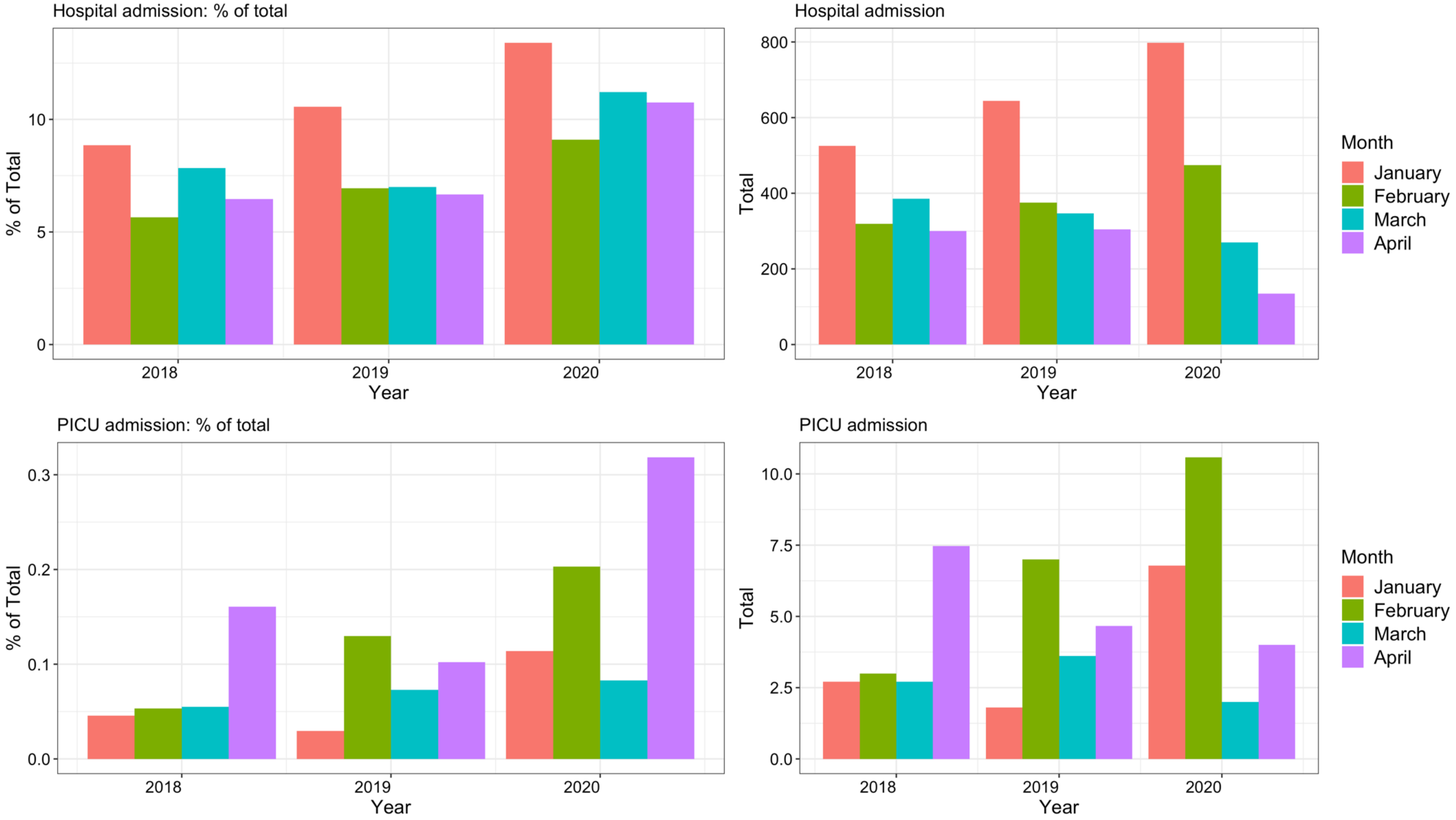

Hospital admission: % of total
% of Total
0
5
10
2018
2019
2020
Year
Hospital admission
Total
0
200
400
600
800
2018
2019
2020
Year
Month
January
February
March
April
PICU admission: % of total
% of Total
0.0
0.1
0.2
0.3
2018
2019
2020
Year
PICU admission
Total
0.0
2.5
5.0
7.5
10.0
2018
2019
2020
Year
Month
January
February
March
April

POR003

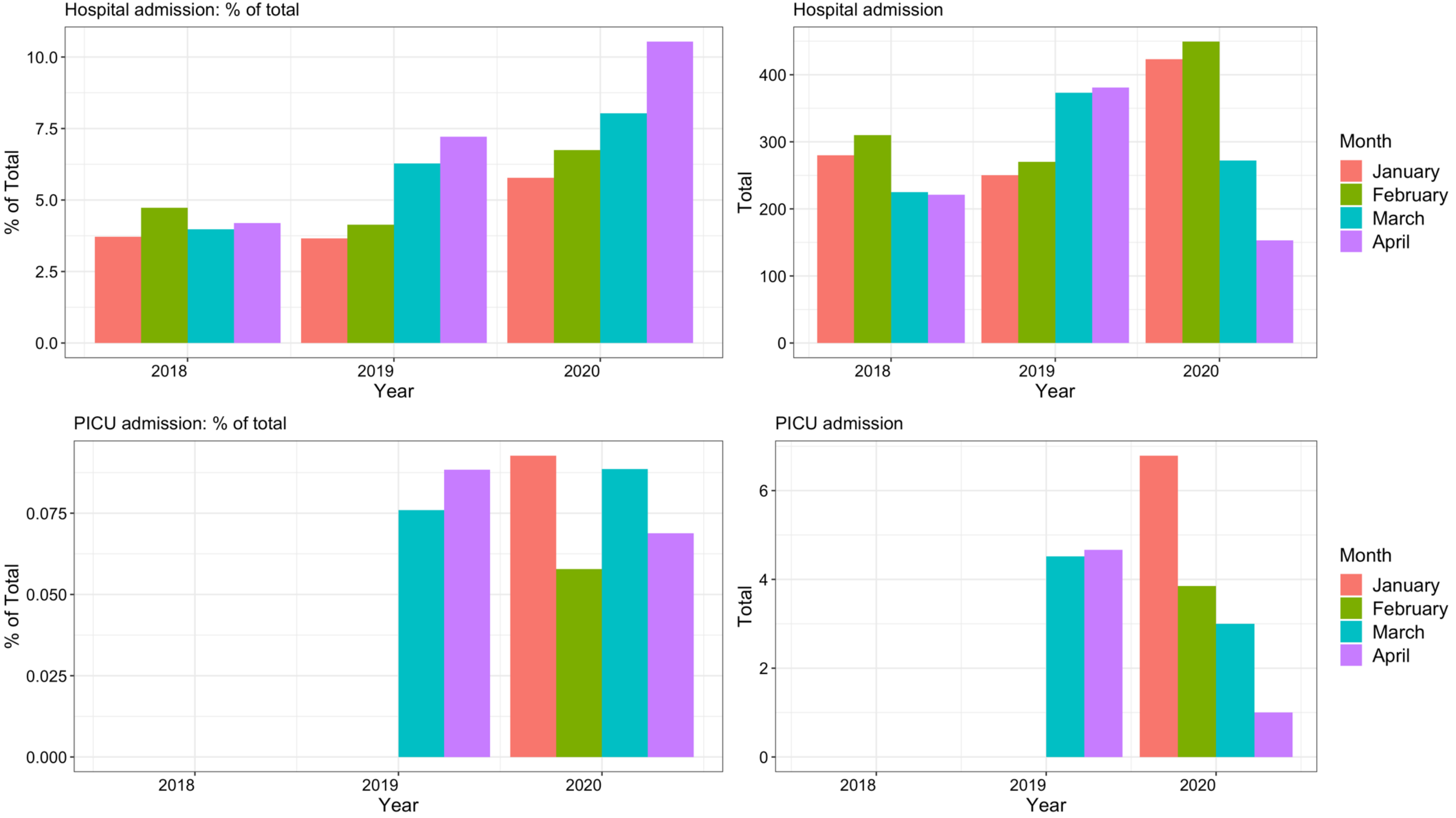
Hospital admission: % of total
% of Total
10.0
7.5
5.0
2.5
0.0
2018
2019
2020
Year
Hospital admission
Total
400
300
200
100
0
Month
January
February
March
April
PICU admission: % of total
0.075
0.050
0.025
0.000
PICU admission
6
4
2

POR004

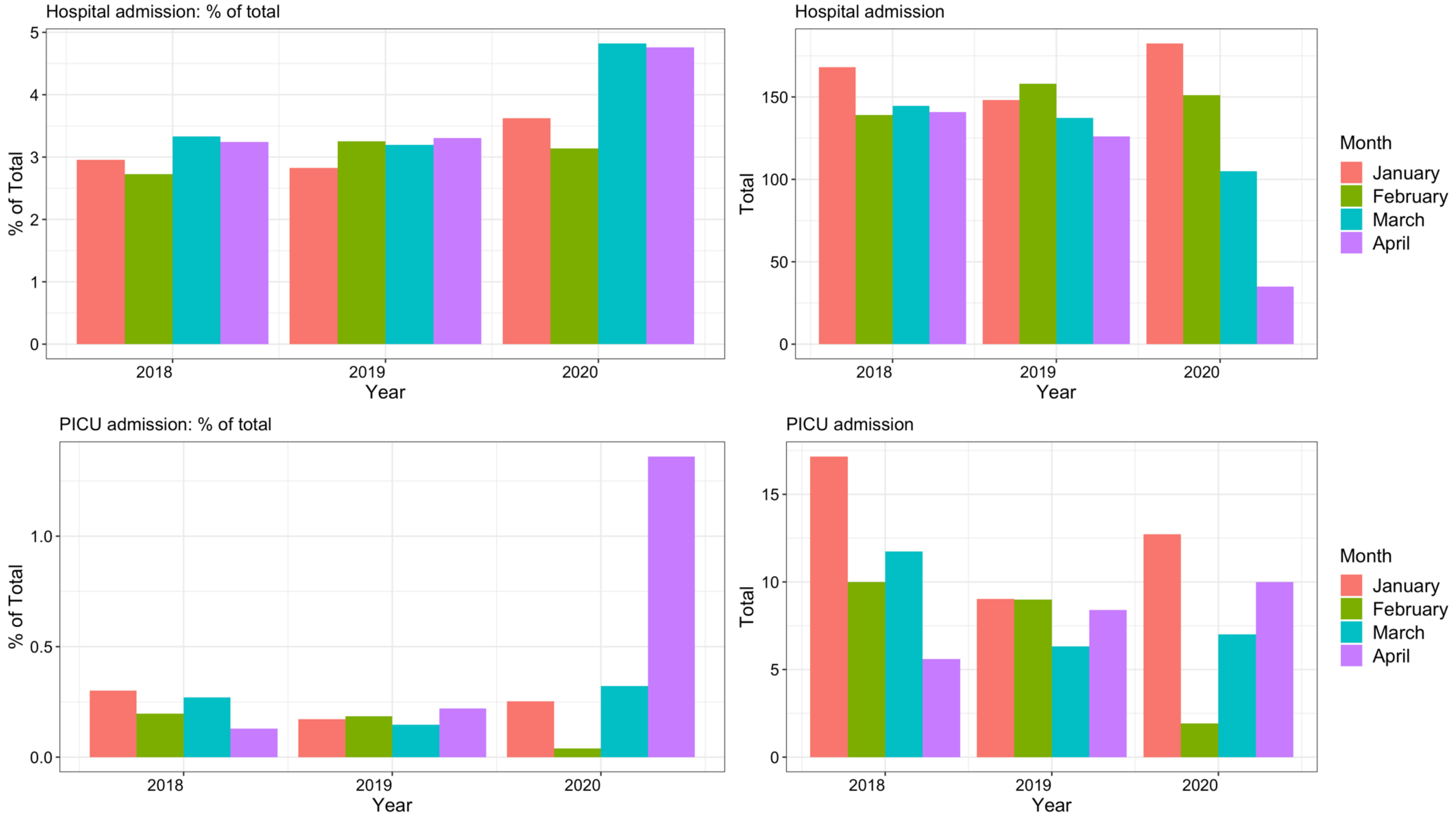
Hospital admission: % of total
% of Total
Year
2018
2019
2020
Hospital admission
Total
Month
January
February
March
April
PICU admission: % of total
PICU admission

POR005

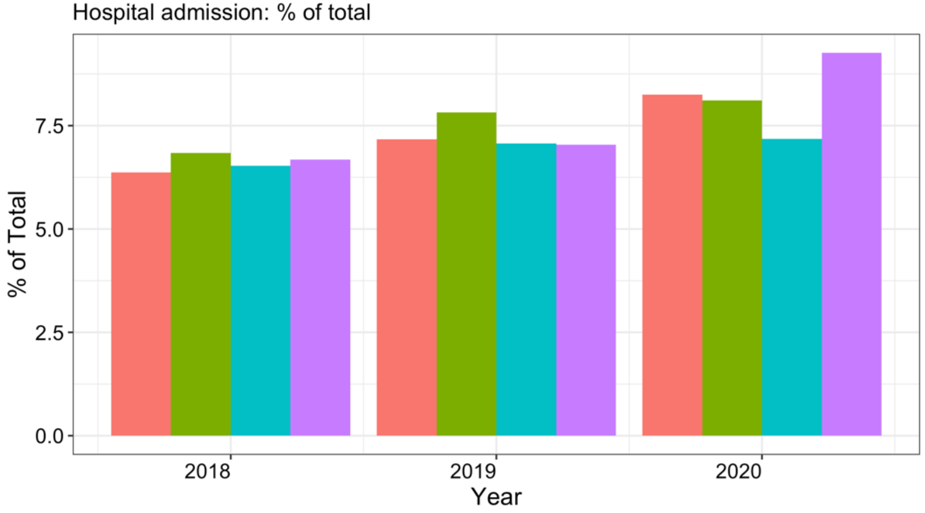
Hospital admission: % of total
% of Total
0.0
2.5
5.0
7.5
2018
2019
2020
Year

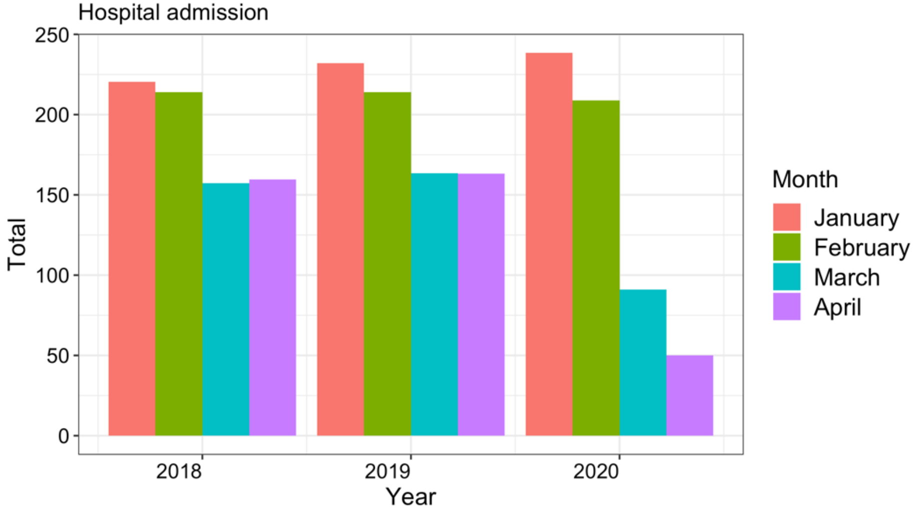
Hospital admission
Total
0
50
100
150
200
250
2018
2019
2020
Year
Month
January
February
March
April

SLO001

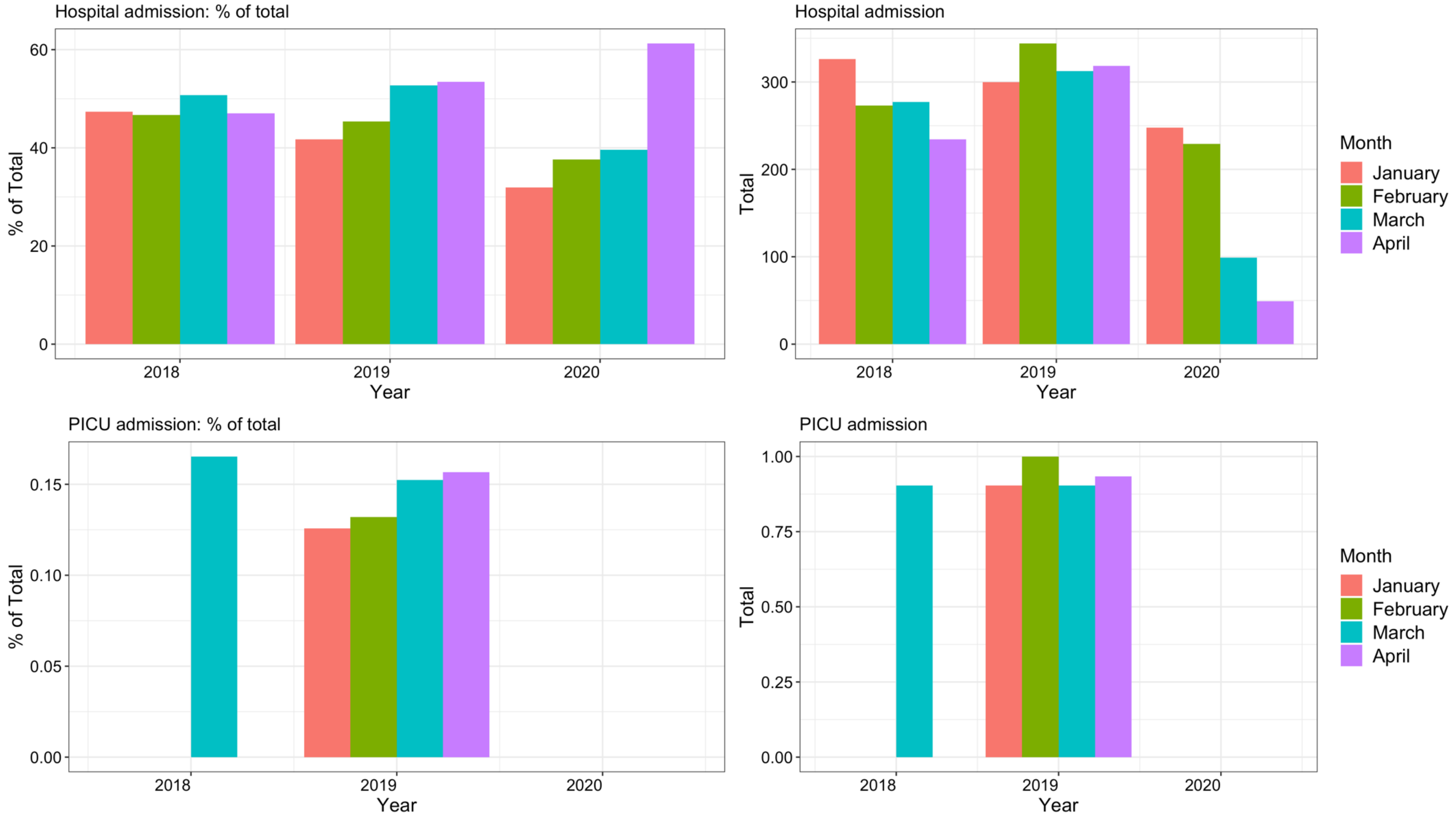
Hospital admission: % of total
% of Total
Year
2018
2019
2020
Hospital admission
Total
PICU admission: % of total
PICU admission
Month
January
February
March
April

SP001

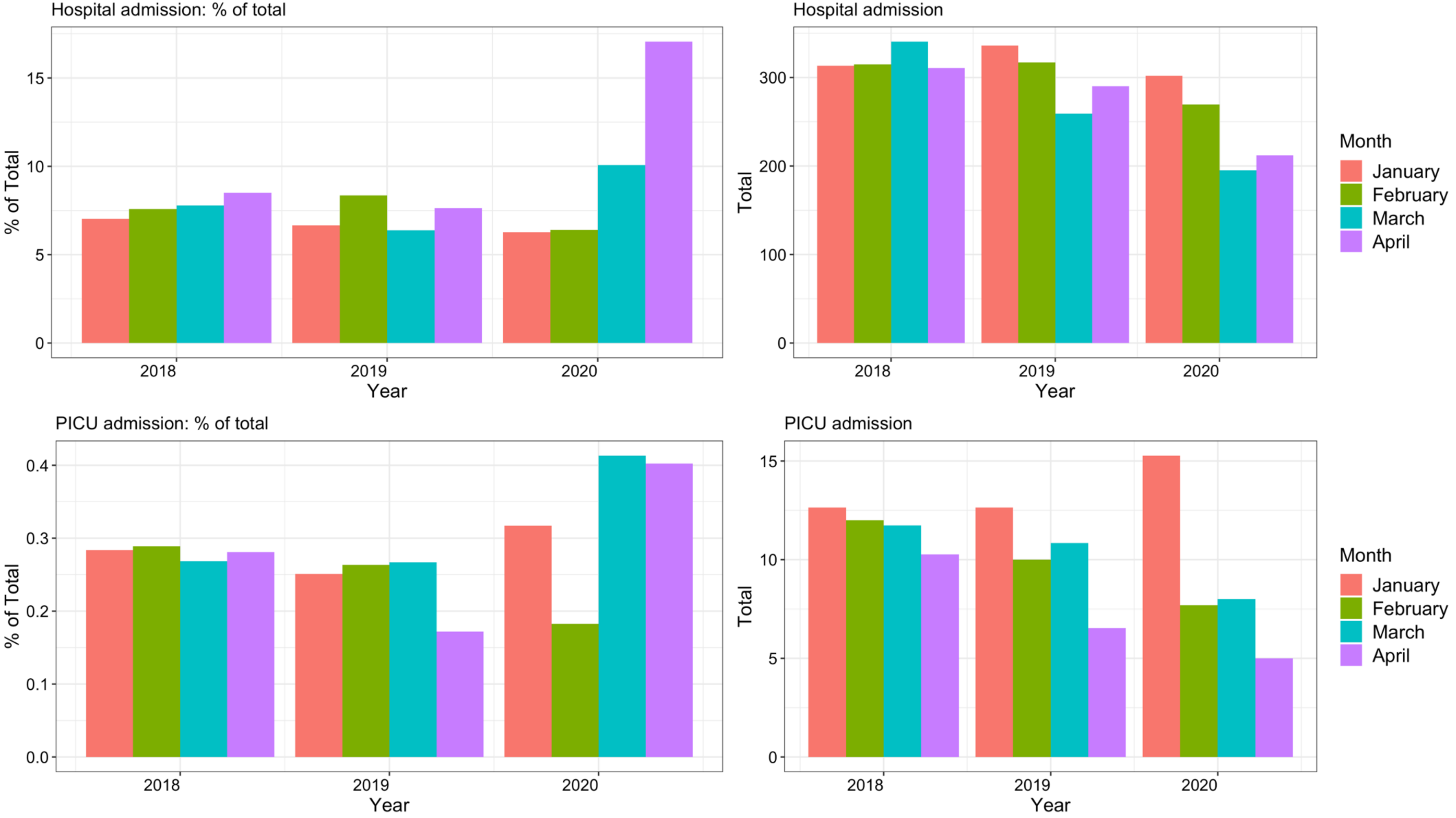

Hospital admission: % of total
% of Total
0
5
10
15
2018
2019
2020
Year
Hospital admission
Total
0
100
200
300
2018
2019
2020
Year
Month
January
February
March
April
PICU admission: % of total
% of Total
0.0
0.1
0.2
0.3
0.4
2018
2019
2020
Year
PICU admission
Total
0
5
10
15
2018
2019
2020
Year
Month
January
February
March
April

SP002

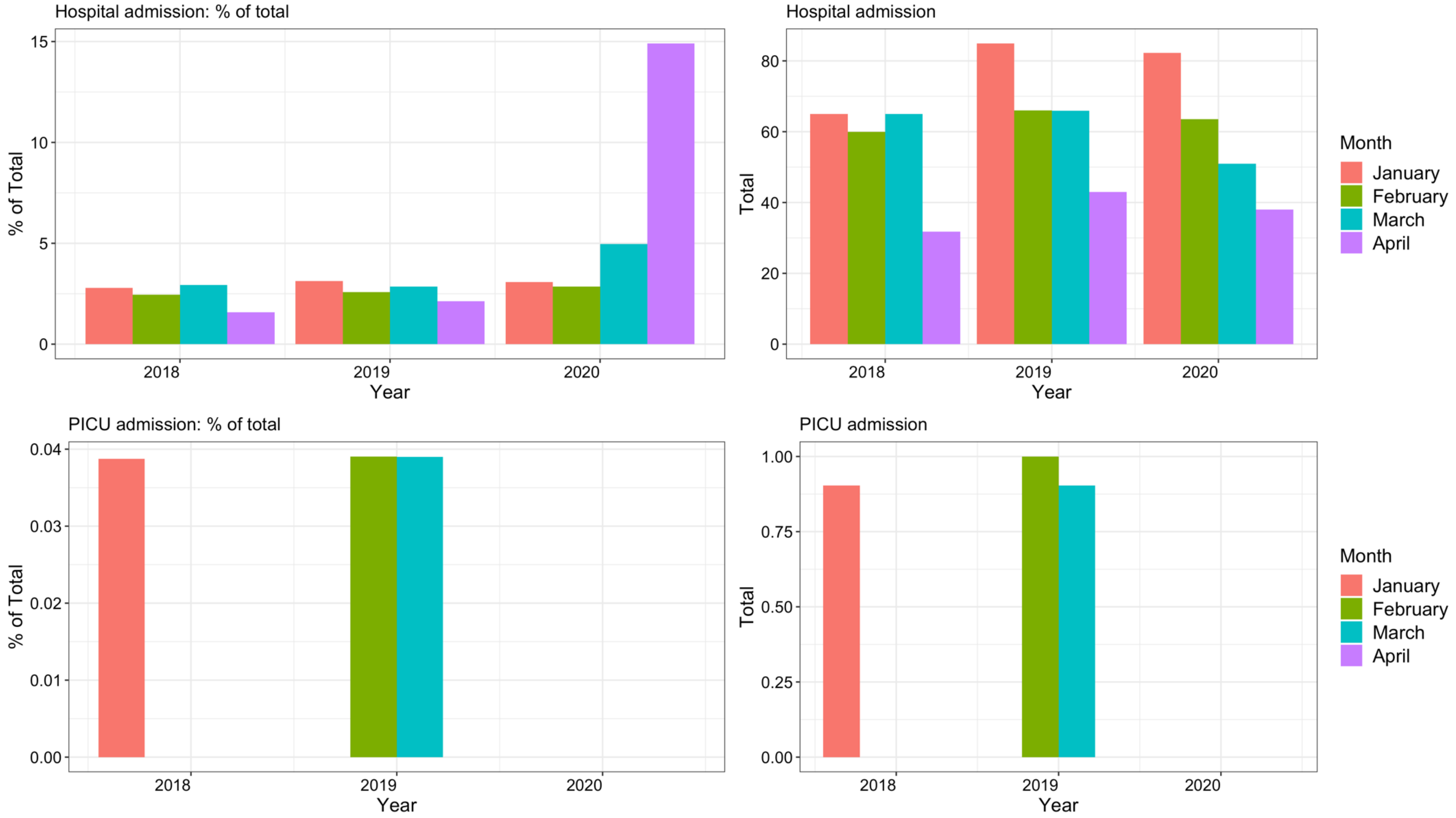
Hospital admission: % of total
% of Total
15
10
5
0
2018
2019
2020
Year
Hospital admission
Total
80
60
40
20
0
2018
2019
2020
Year
Month
January
February
March
April
PICU admission: % of total
% of Total
0.04
0.03
0.02
0.01
0.00
2018
2019
2020
Year
PICU admission
Total
1.00
0.75
0.50
0.25
0.00
2018
2019
2020
Year
Month
January
February
March
April

SWE001

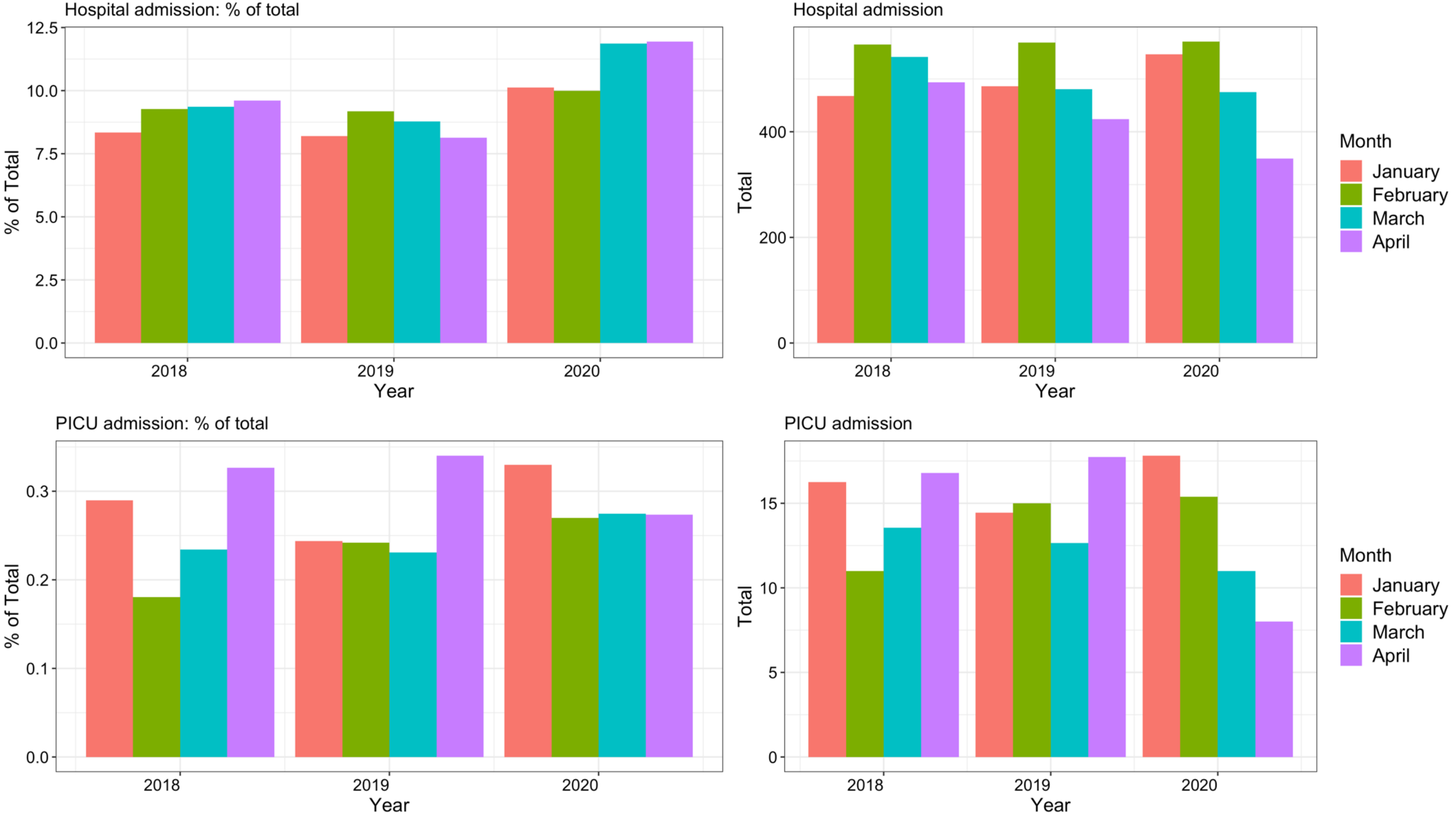

Hospital admission: % of total
12.5
10.0
7.5
5.0
2.5
0.0
% of Total
2018
2019
2020
Year
Hospital admission
400
200
0
Total
2018
2019
2020
Year
Month
January
February
March
April
PICU admission: % of total
0.3
0.2
0.1
0.0
% of Total
2018
2019
2020
Year
PICU admission
15
10
5
0
Total
2018
2019
2020
Year
Month
January
February
March
April

SWE002

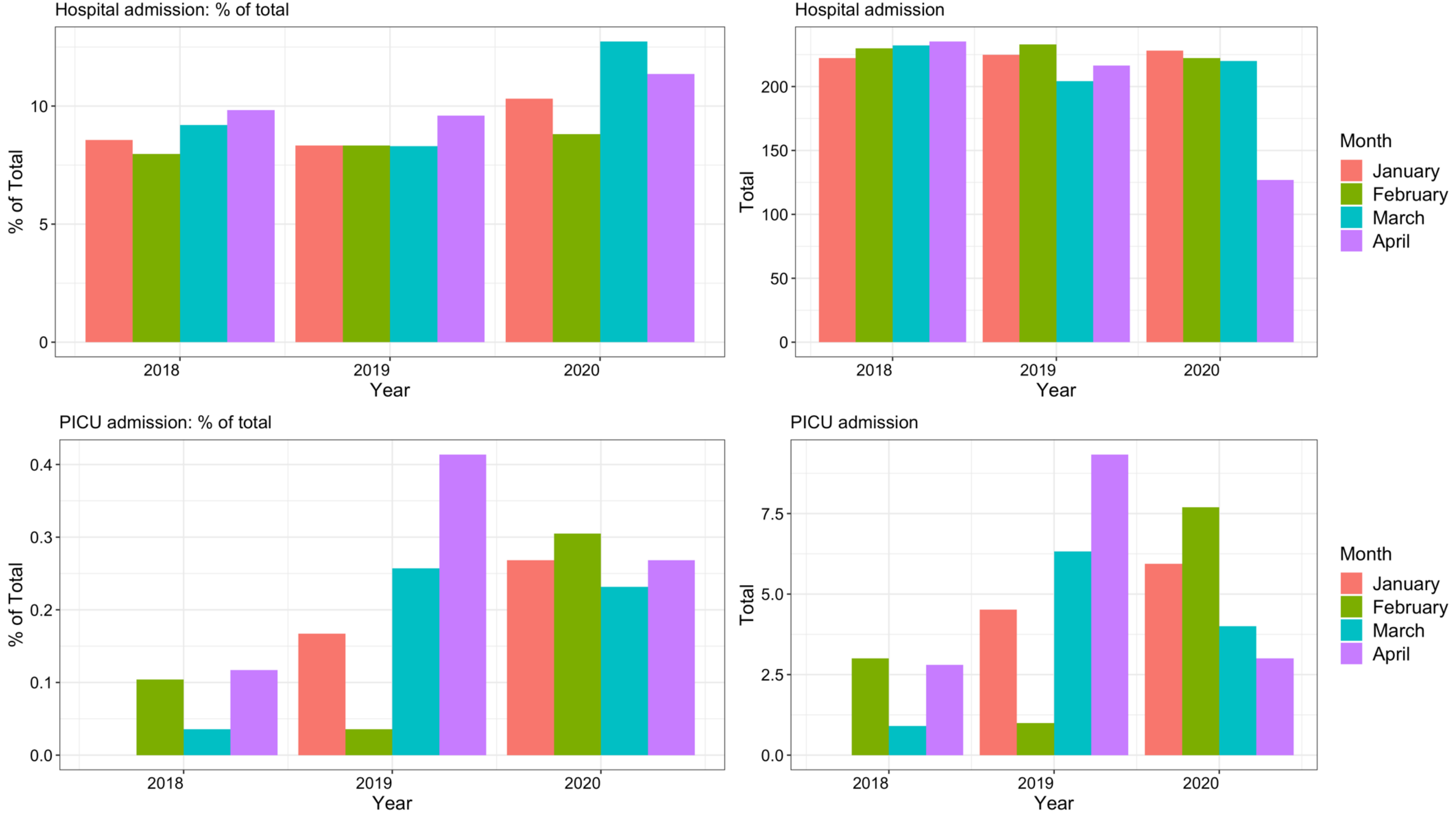

Hospital admission: % of total
% of Total
0
5
10
2018
2019
2020
Year
Hospital admission
Total
0
50
100
150
200
2018
2019
2020
Year
Month
January
February
March
April
PICU admission: % of total
% of Total
0.0
0.1
0.2
0.3
0.4
2018
2019
2020
Year
PICU admission
Total
0.0
2.5
5.0
7.5
2018
2019
2020
Year
Month
January
February
March
April

TUR001

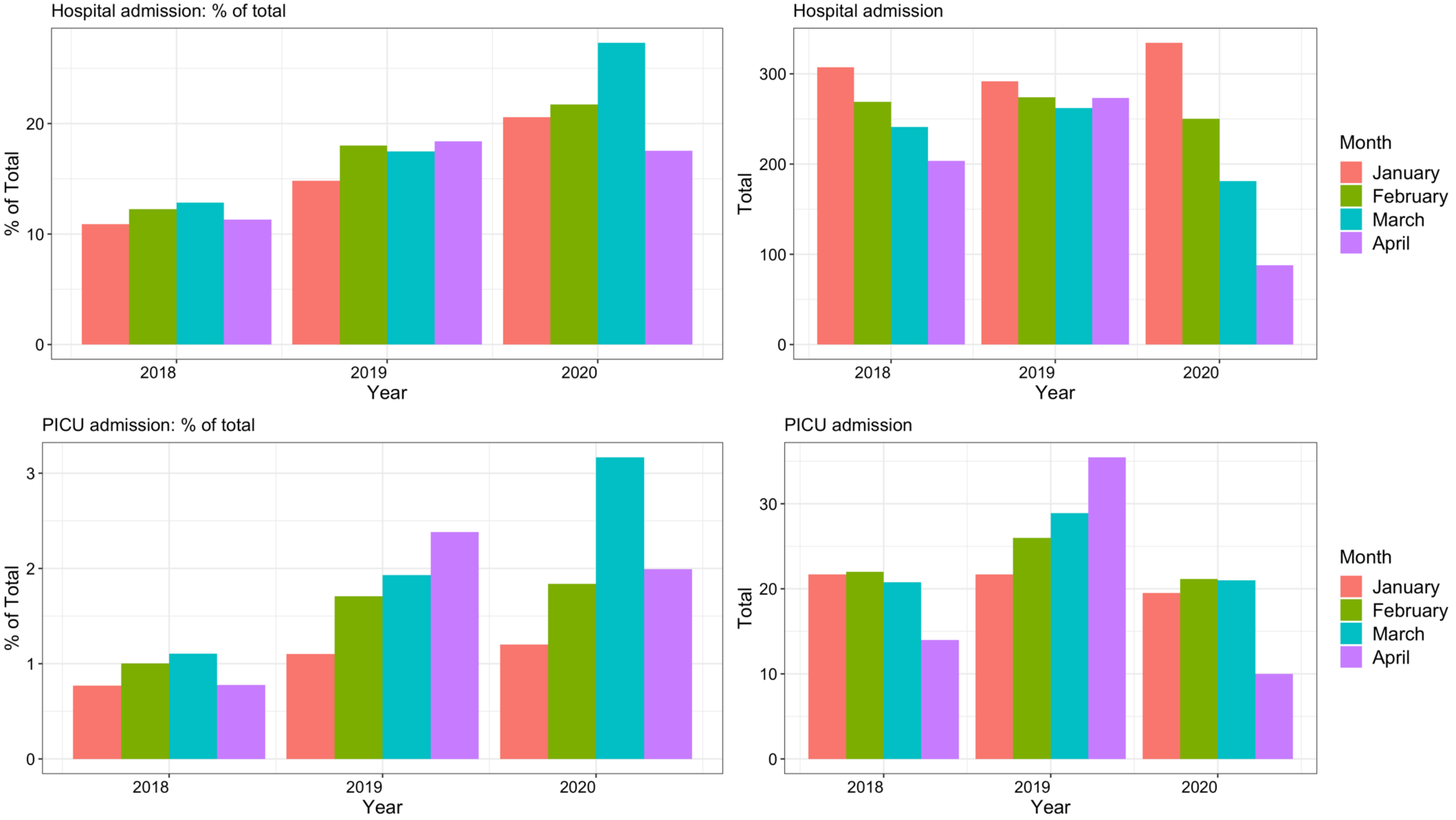

Hospital admission: % of total
% of Total
0
10
20
2018
2019
2020
Year
Hospital admission
Total
0
100
200
300
2018
2019
2020
Year
Month
January
February
March
April
PICU admission: % of total
% of Total
0
1
2
3
2018
2019
2020
Year
PICU admission
Total
0
10
20
30
2018
2019
2020
Year
Month
January
February
March
April

TUR002

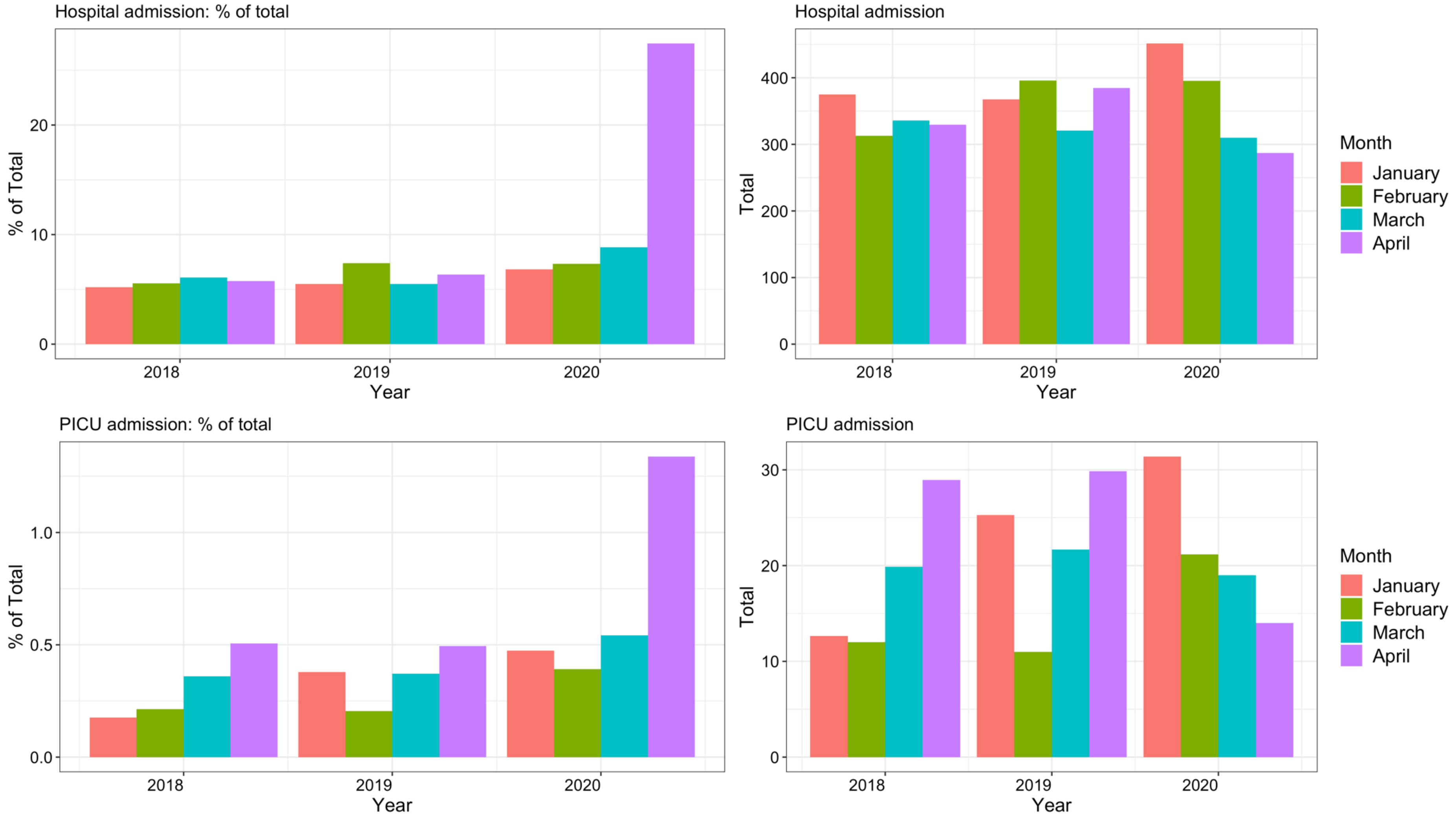

Hospital admission: % of total
% of Total
Year
Hospital admission
Total
Month
January
February
March
April
PICU admission: % of total
PICU admission
2018
2019
2020

TUR003

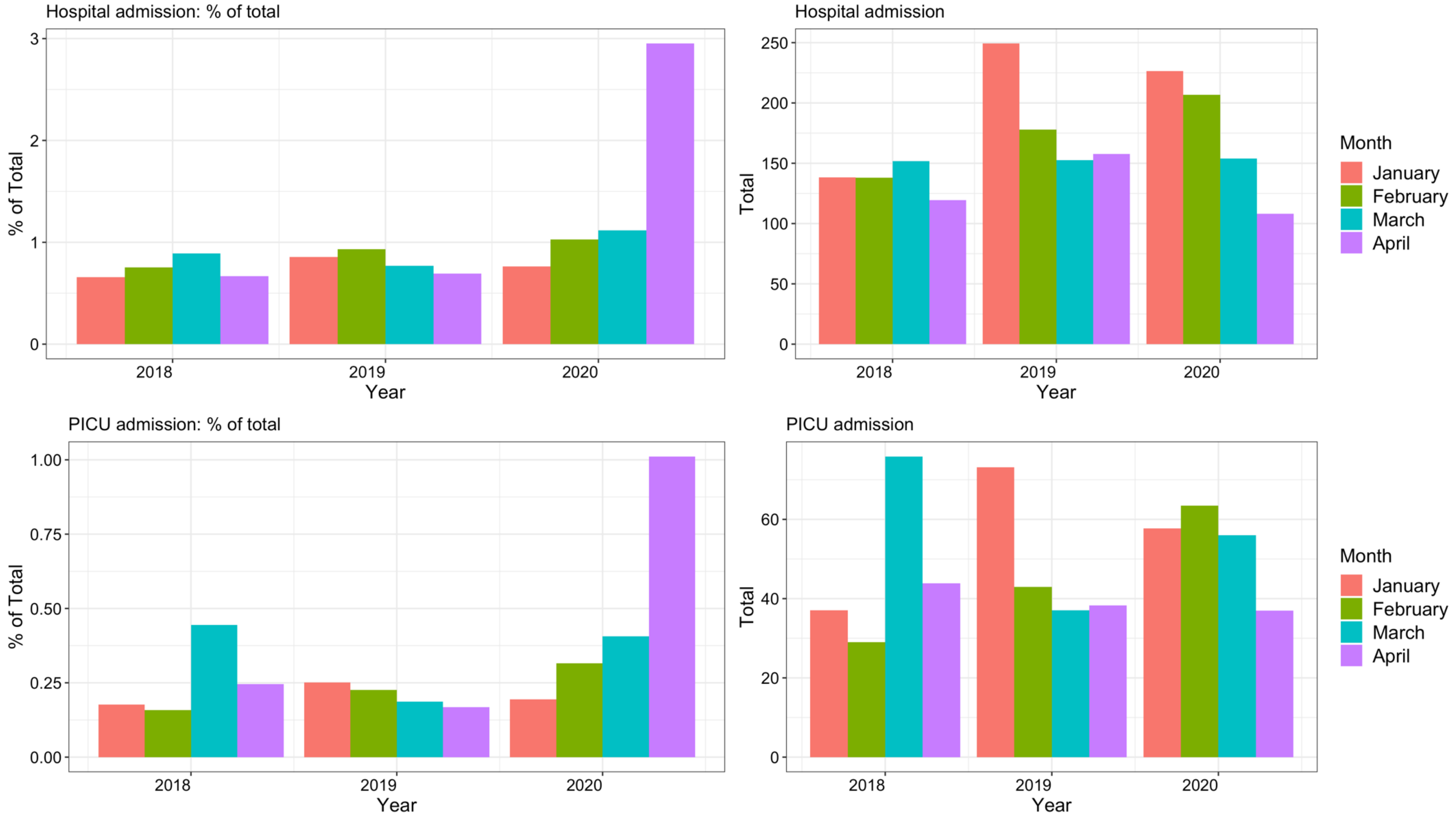
Hospital admission: % of total
% of Total
3
2
1
0
2018
2019
2020
Year
Hospital admission
Total
250
200
150
100
50
0
2018
2019
2020
Year
Month
January
February
March
April
PICU admission: % of total
% of Total
1.00
0.75
0.50
0.25
0.00
2018
2019
2020
Year
PICU admission
Total
60
40
20
0
2018
2019
2020
Year
Month
January
February
March
April

UK001

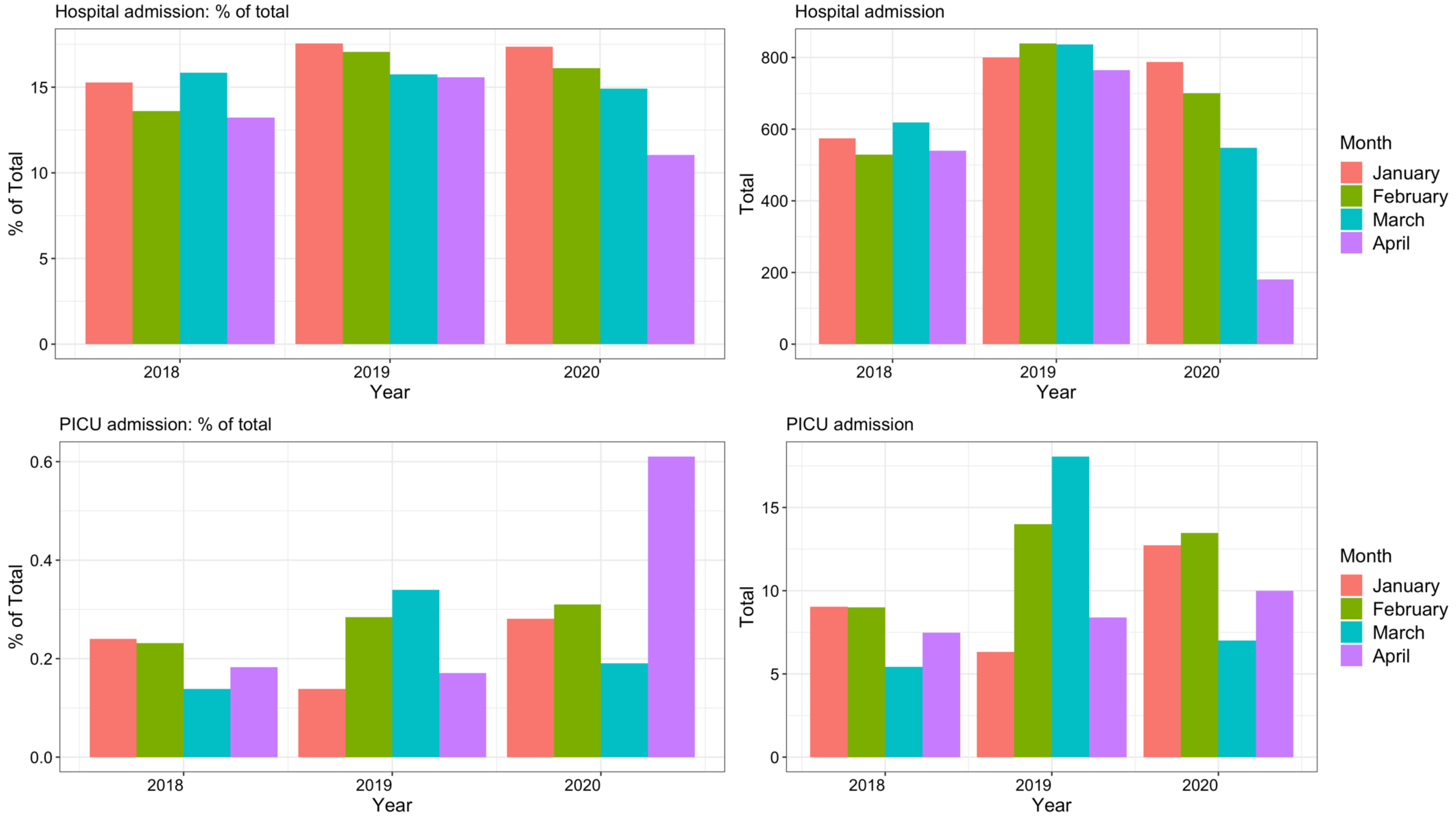
Hospital admission: % of total
% of Total
0
5
10
15
2018
2019
2020
Year
Hospital admission
Total
0
200
400
600
800
2018
2019
2020
Year
Month
January
February
March
April
PICU admission: % of total
% of Total
0.0
0.2
0.4
0.6
2018
2019
2020
Year
PICU admission
Total
0
5
10
15
2018
2019
2020
Year
Month
January
February
March
April

UK002

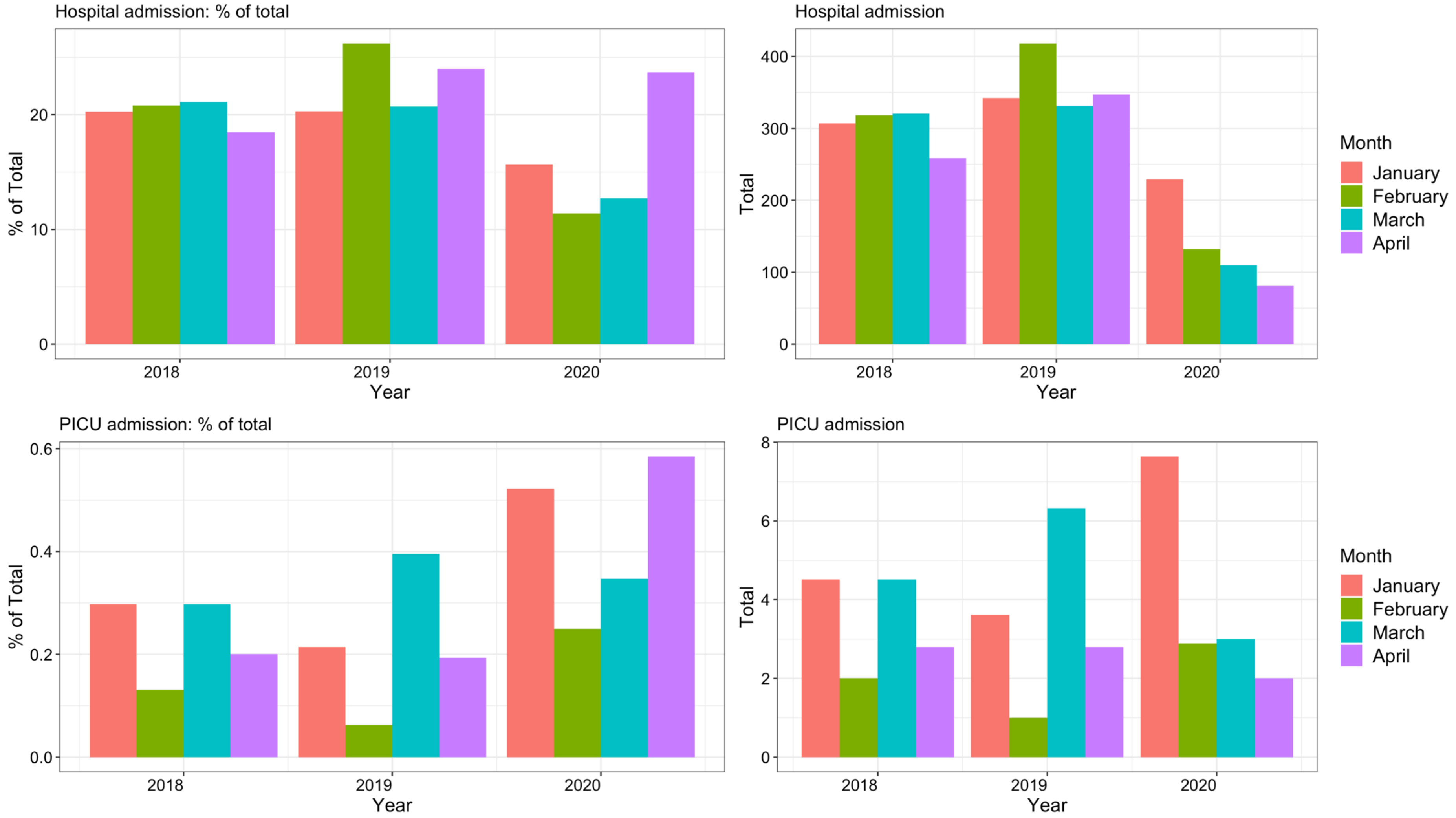
Hospital admission: % of total
% of Total
Year
2018
2019
2020
Hospital admission
Total
Month
January
February
March
April
PICU admission: % of total
PICU admission

UK004

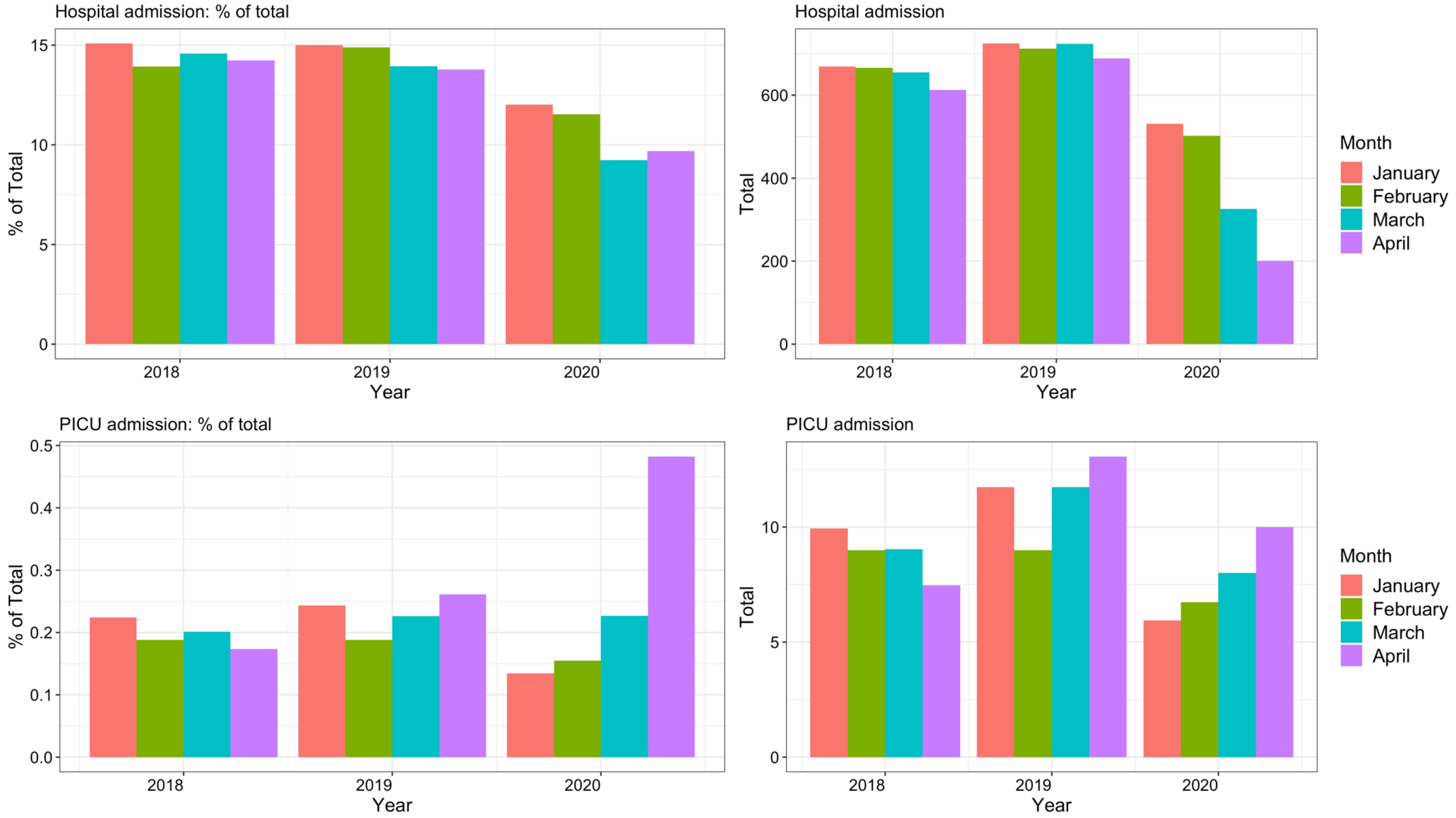
Hospital admission: % of total
% of Total
0
5
10
15
2018
2019
2020
Year
Hospital admission
Total
0
200
400
600
2018
2019
2020
Year
Month
January
February
March
April
PICU admission: % of total
% of Total
0.0
0.1
0.2
0.3
0.4
0.5
2018
2019
2020
Year
PICU admission
Total
0
5
10
2018
2019
2020
Year
Month
January
February
March
April

UK005

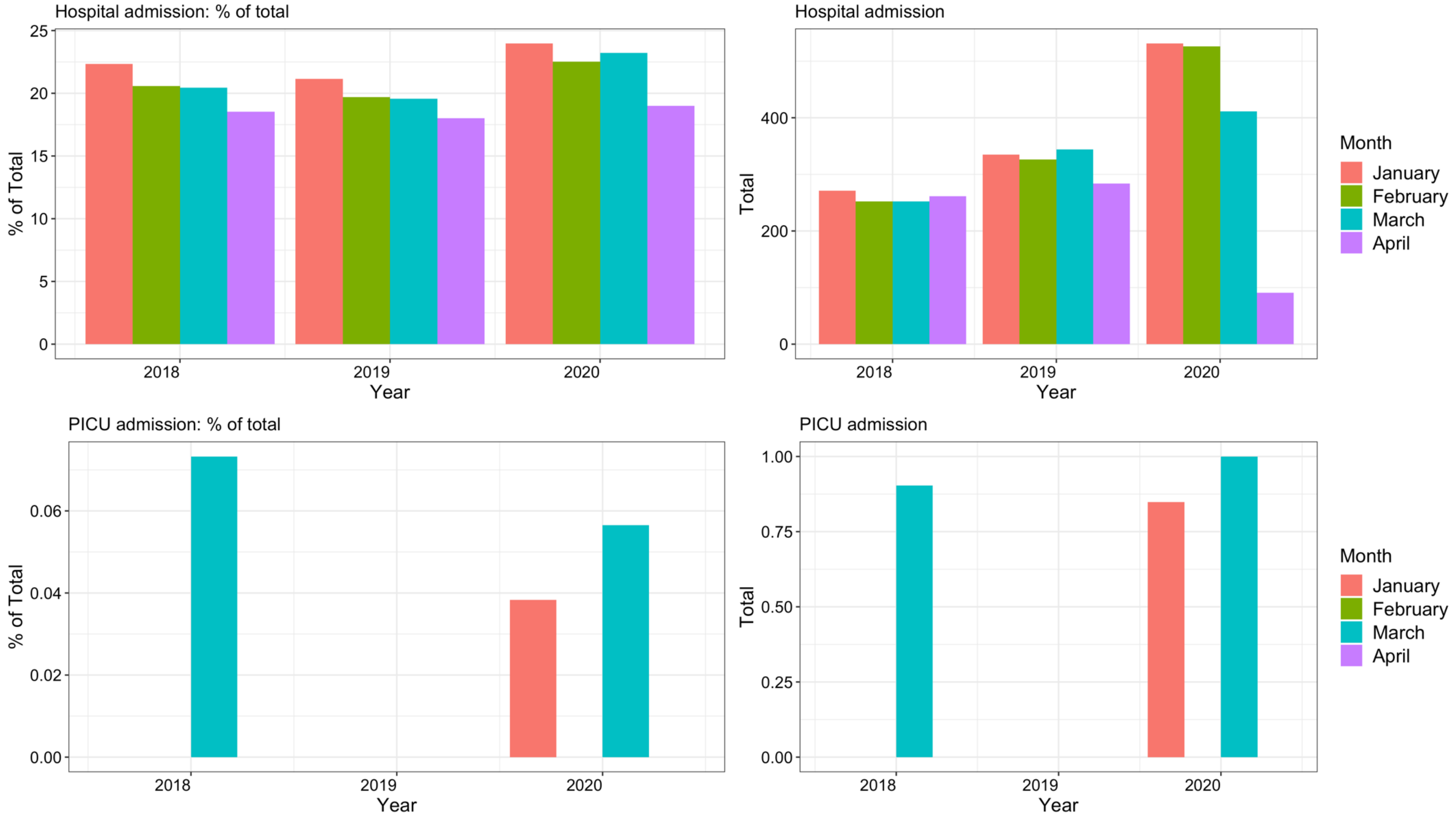
Hospital admission: % of total
% of Total
25
20
15
10
5
0
2018
2019
2020
Year
Hospital admission
Total
400
200
0
2018
2019
2020
Year
Month
January
February
March
April
PICU admission: % of total
% of Total
0.06
0.04
0.02
0.00
2018
2019
2020
Year
PICU admission
Total
1.00
0.75
0.50
0.25
0.00
2018
2019
2020
Year
Month
January
February
March
April

UK006

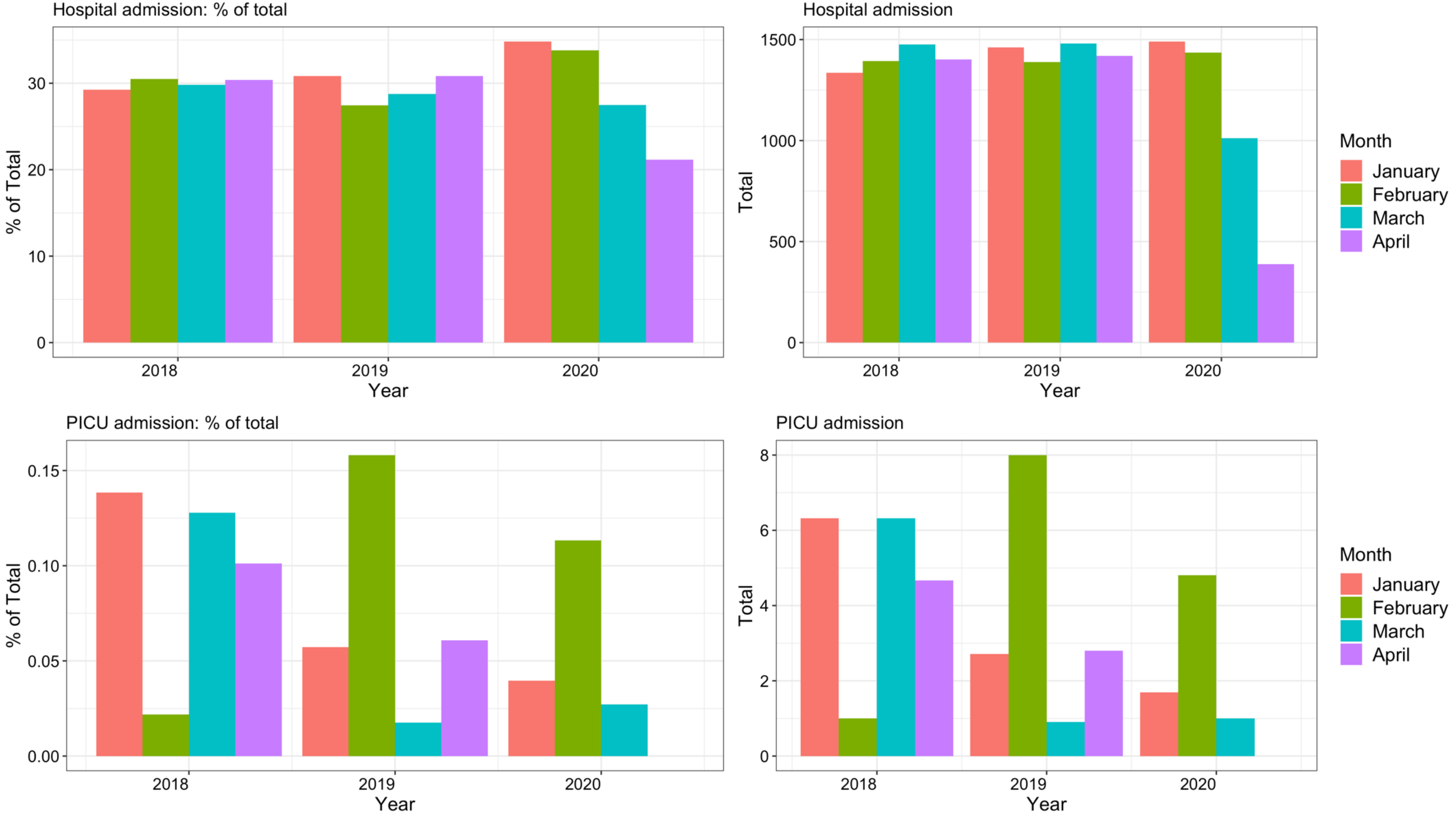

Hospital admission: % of total
% of Total
0
10
20
30
2018
2019
2020
Year
Hospital admission
Total
0
500
1000
1500
2018
2019
2020
Year
Month
January
February
March
April
PICU admission: % of total
% of Total
0.00
0.05
0.10
0.15
2018
2019
2020
Year
PICU admission
Total
0
2
4
6
8
2018
2019
2020
Year
Month
January
February
March
April
