# Supplementary material for: Presentations of children to emergency departments across Europe and the COVID-19 pandemic: A multinational observational study
Source: PLoS Med. 2022 Aug 26;19(8):e1003974. doi: 10.1371/journal.pmed.1003974 (PMC9467376; doi:10.1371/journal.pmed.1003974)

S10 Fig. Selected clinical diagnoses in the emergency department for the period January – April over a three-year period, for high prevalence countries.

Legend:

Percentages of total ED attendances (left) and absolute numbers (right) of children with diagnosis of a) tonsillitis, b) otitis media, c) lower respiratory tract infections (LRTI), d) gastro-intestinal (GI) infections, e) appendicitis, f) testicular torsion, g) intussusception, h) mental health issues, i) diabetic keto-acidosis, j) radius fracture, k) minor head injury; comparing the 28-day standardized numbers for the months of January – April for 2018 vs. 2019 vs. 2020, shown for countries with of a cumulative 14 day rate of new SARS-CoV-2 cases per 100.000 of 80 or more.

Tonsillitis

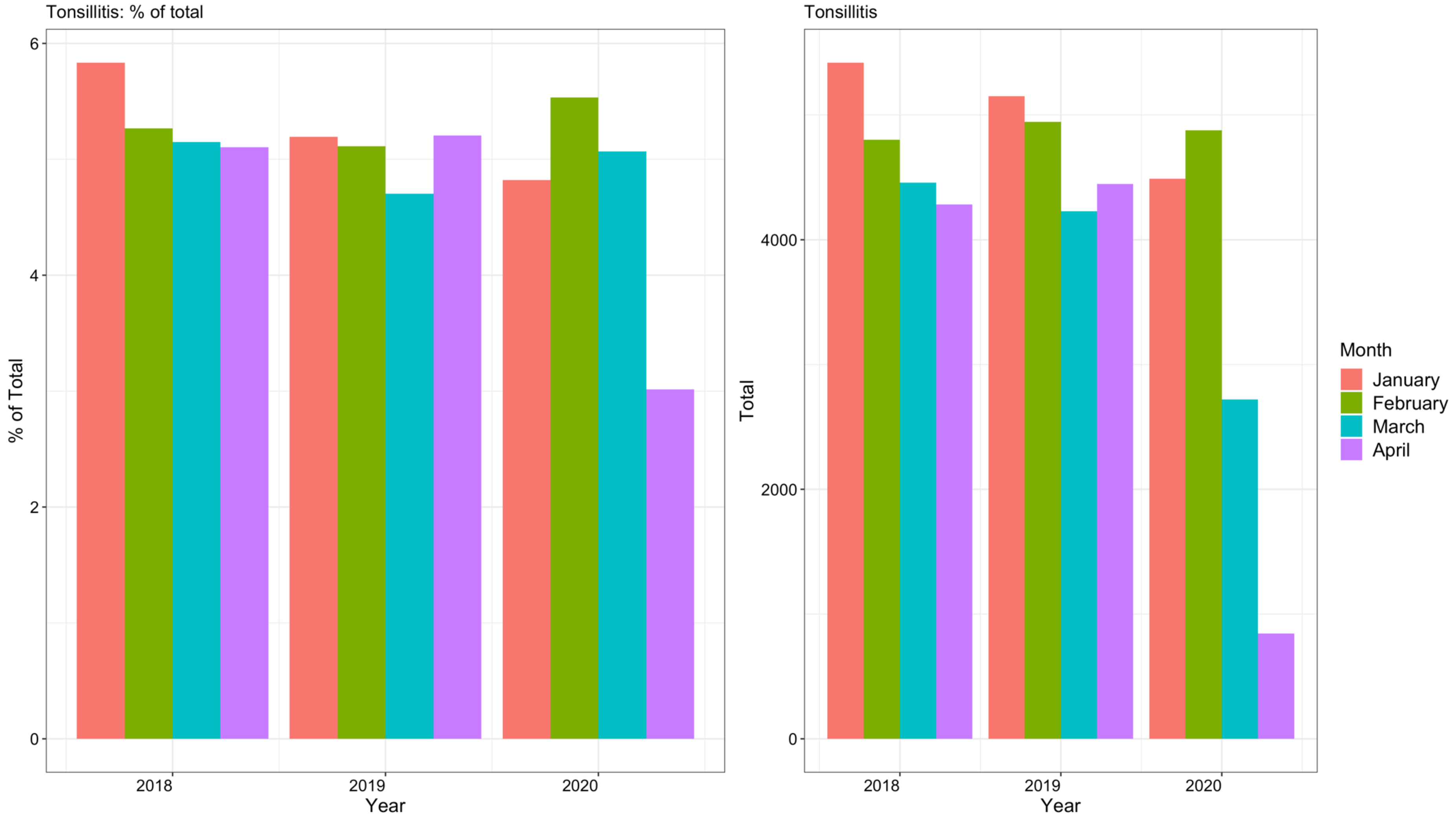

Otitis media

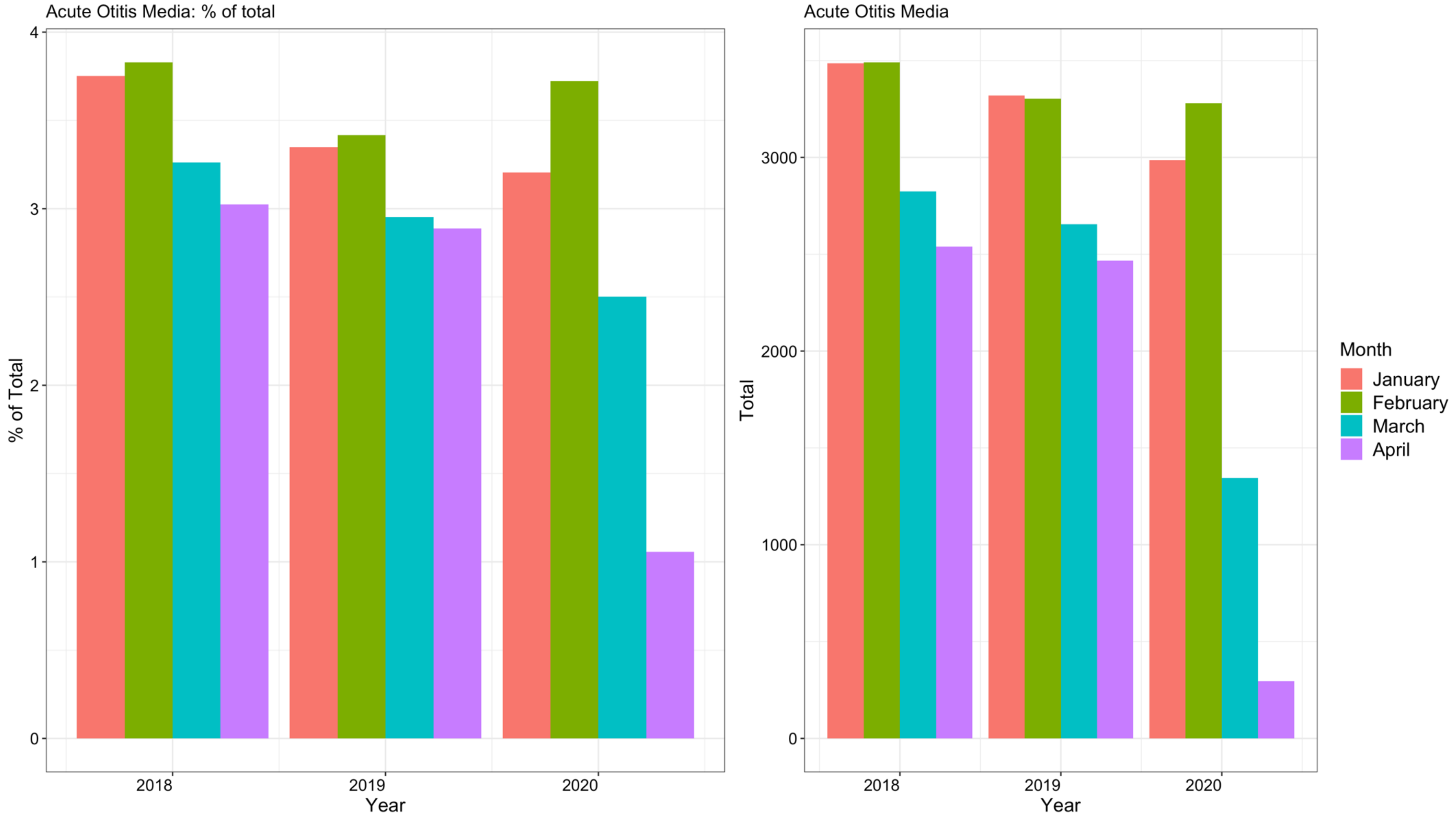

Lower respiratory tract infections

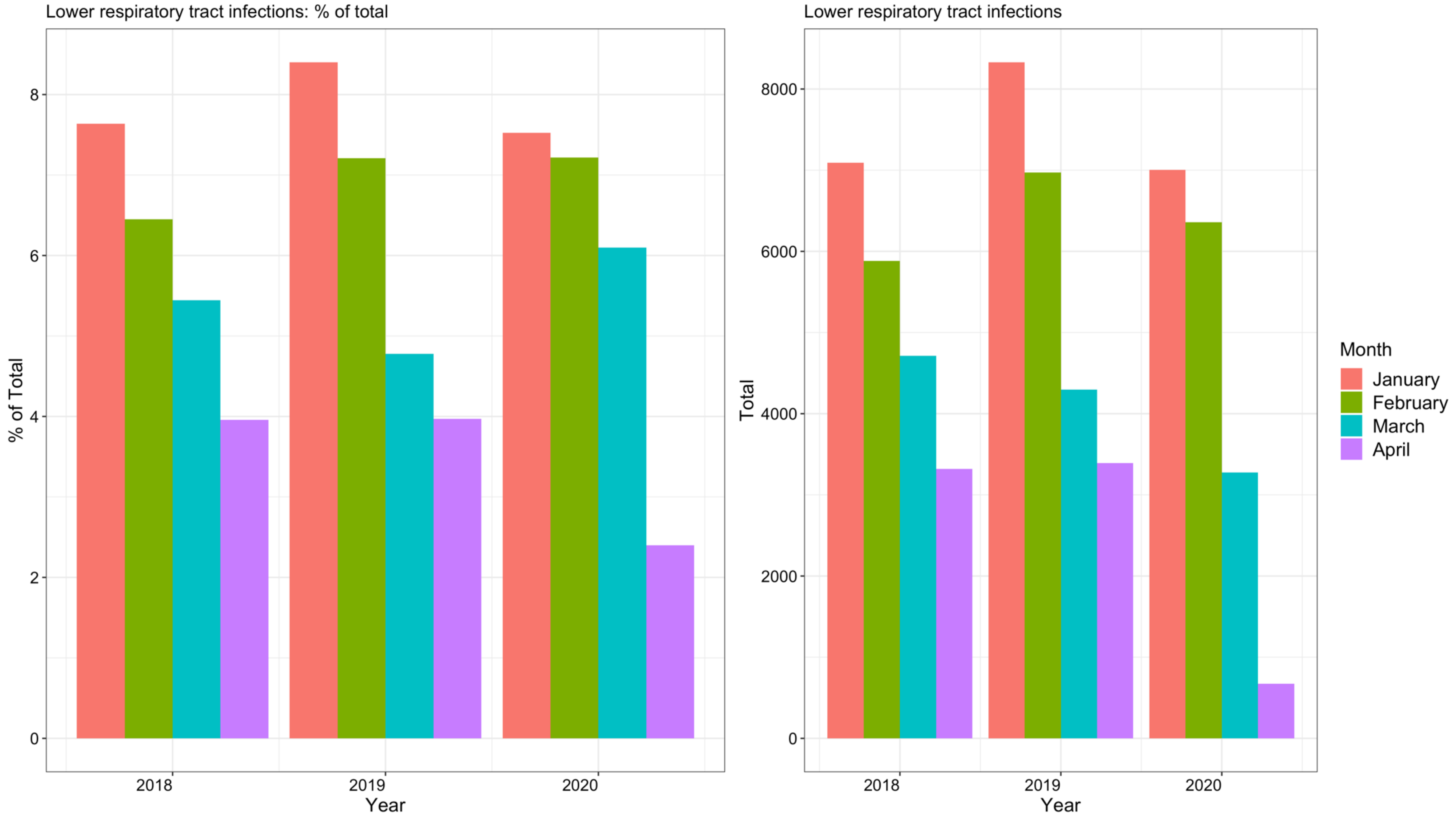

Gastro-intestinal infections

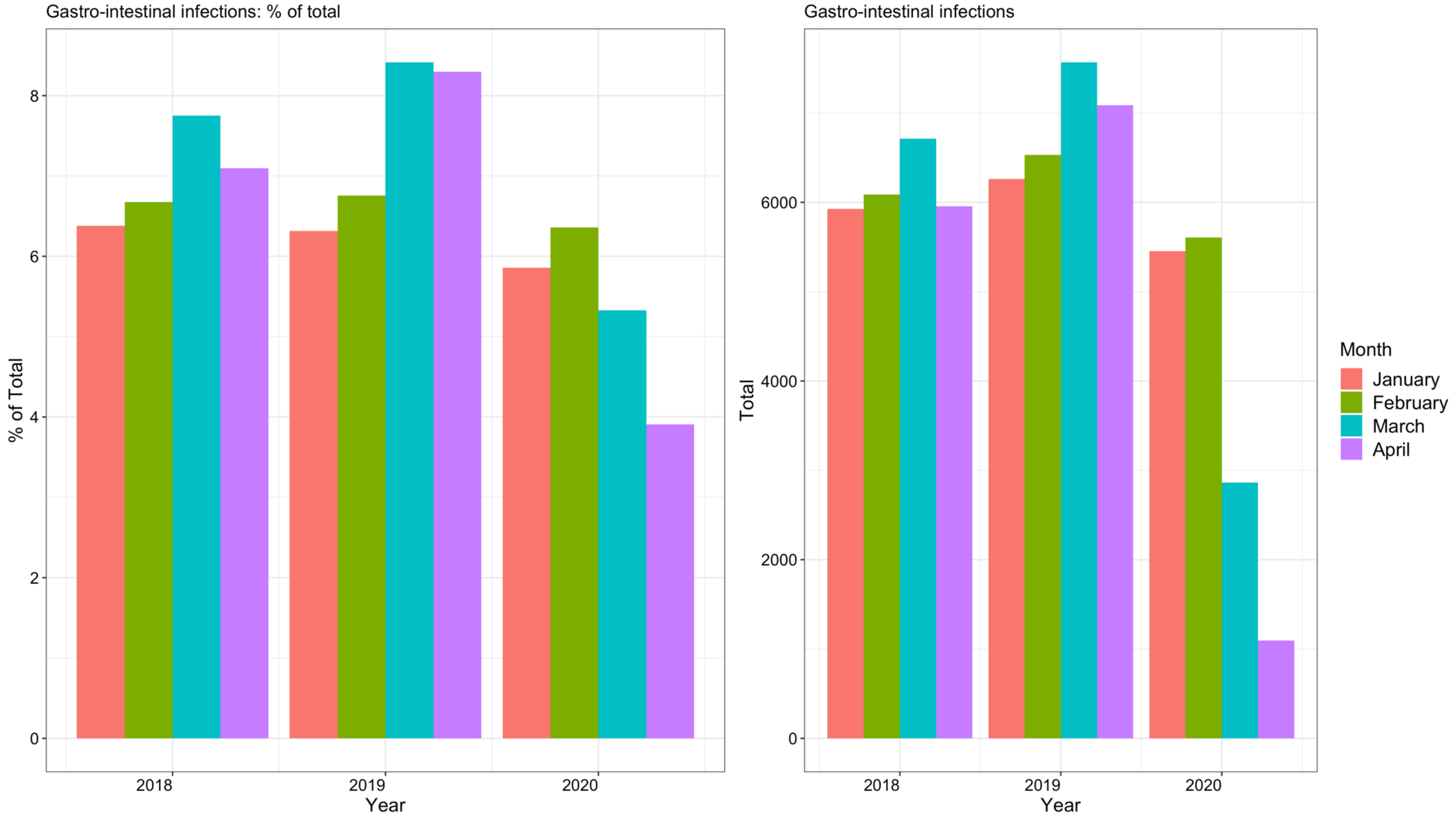

Appendicitis

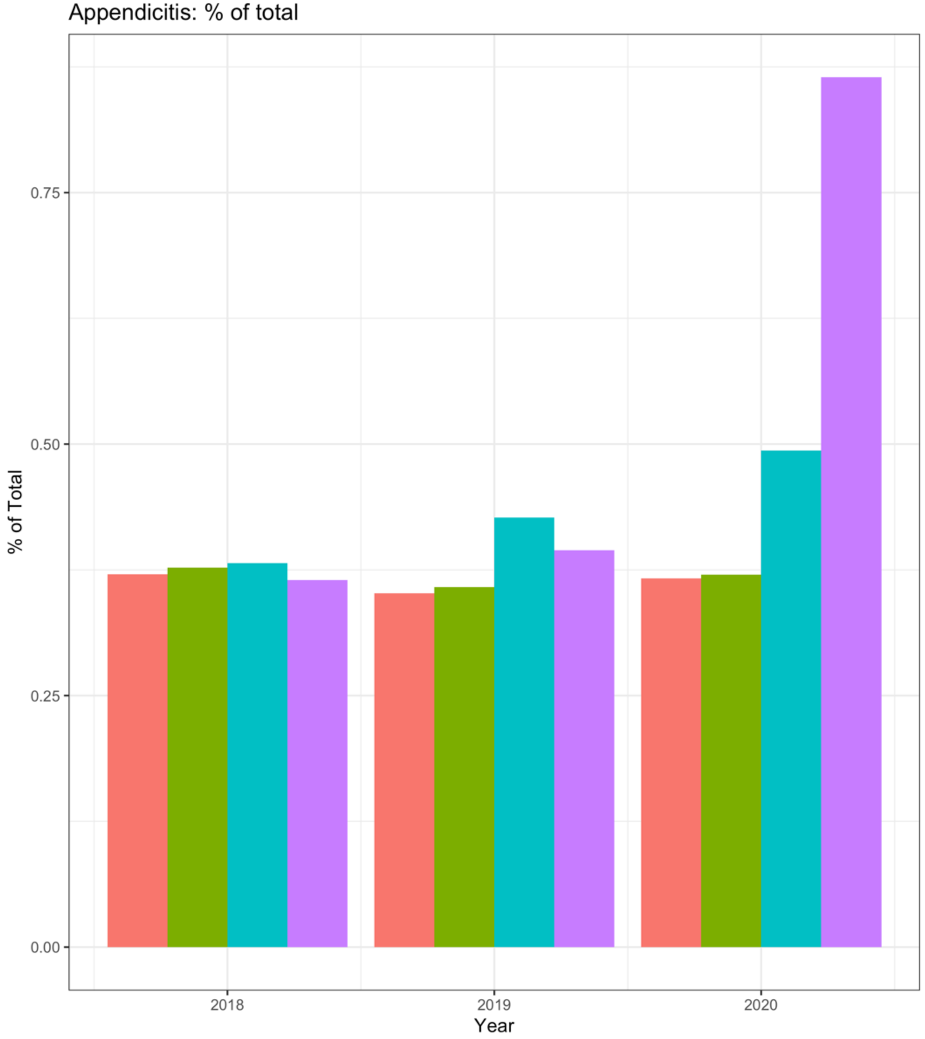

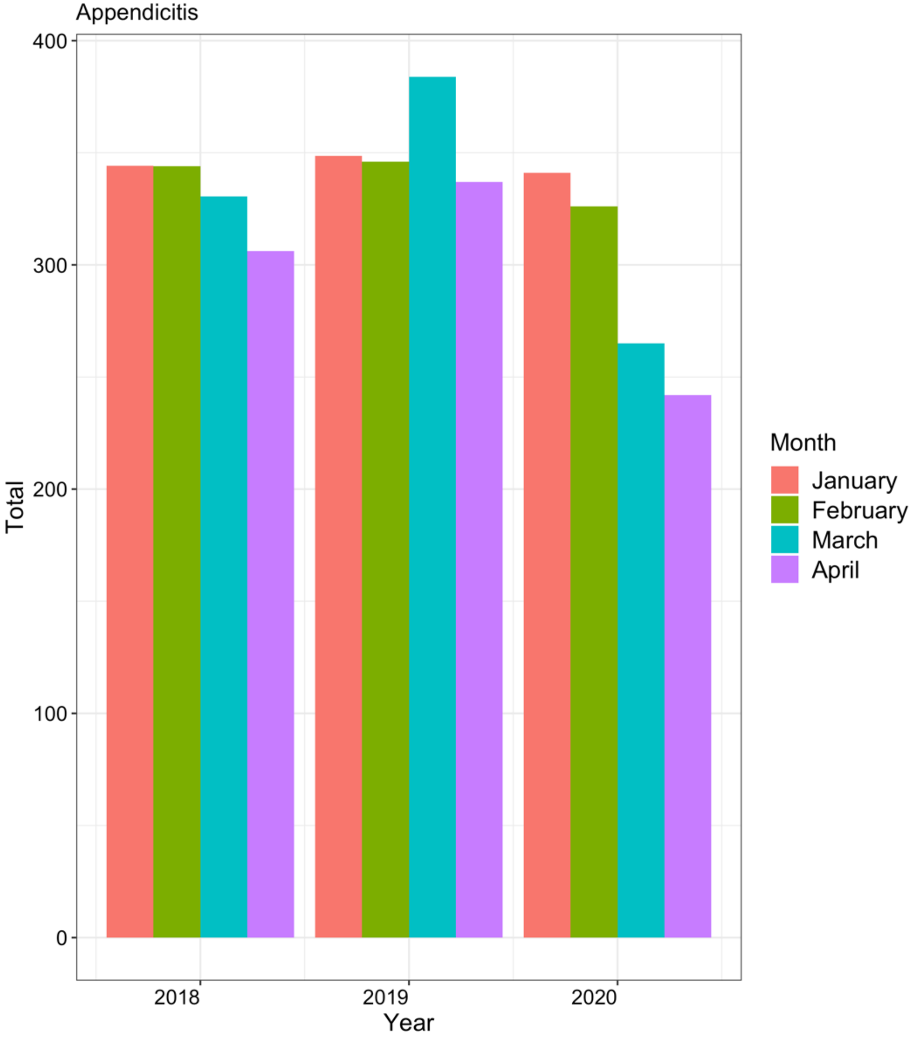

Testicular torsion

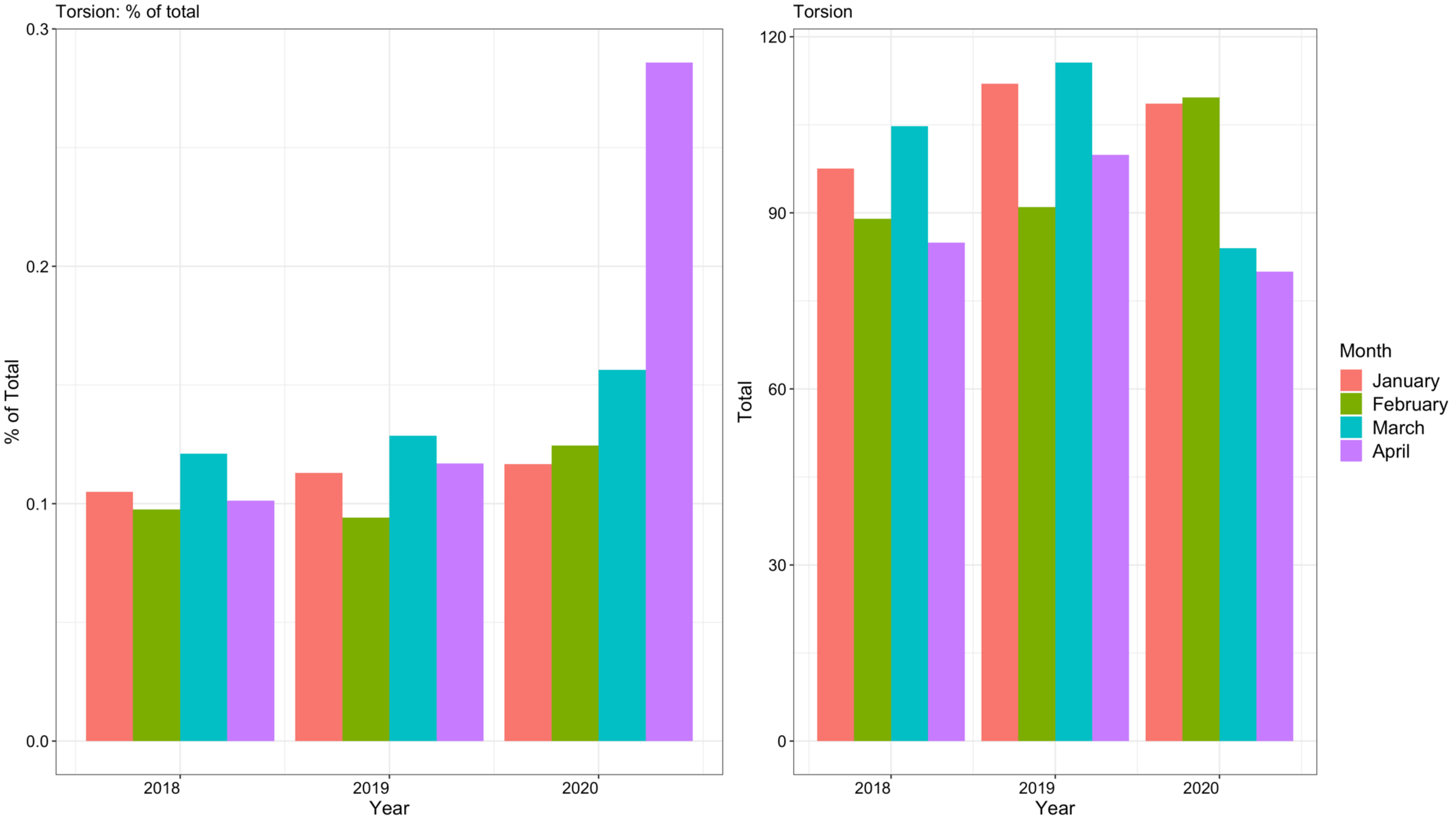

Intussusception, volvulus, and malrotation

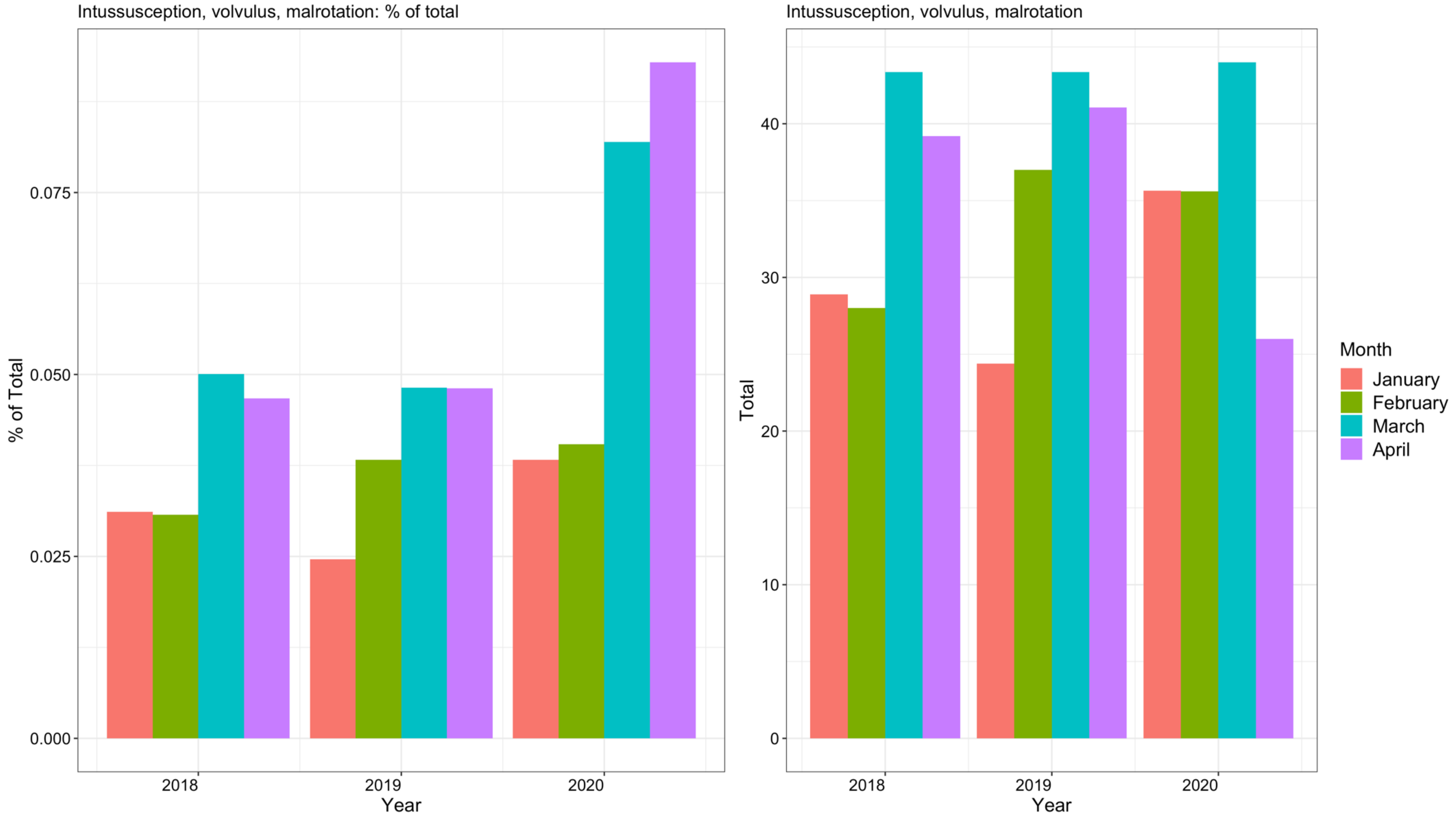

Mental health issues

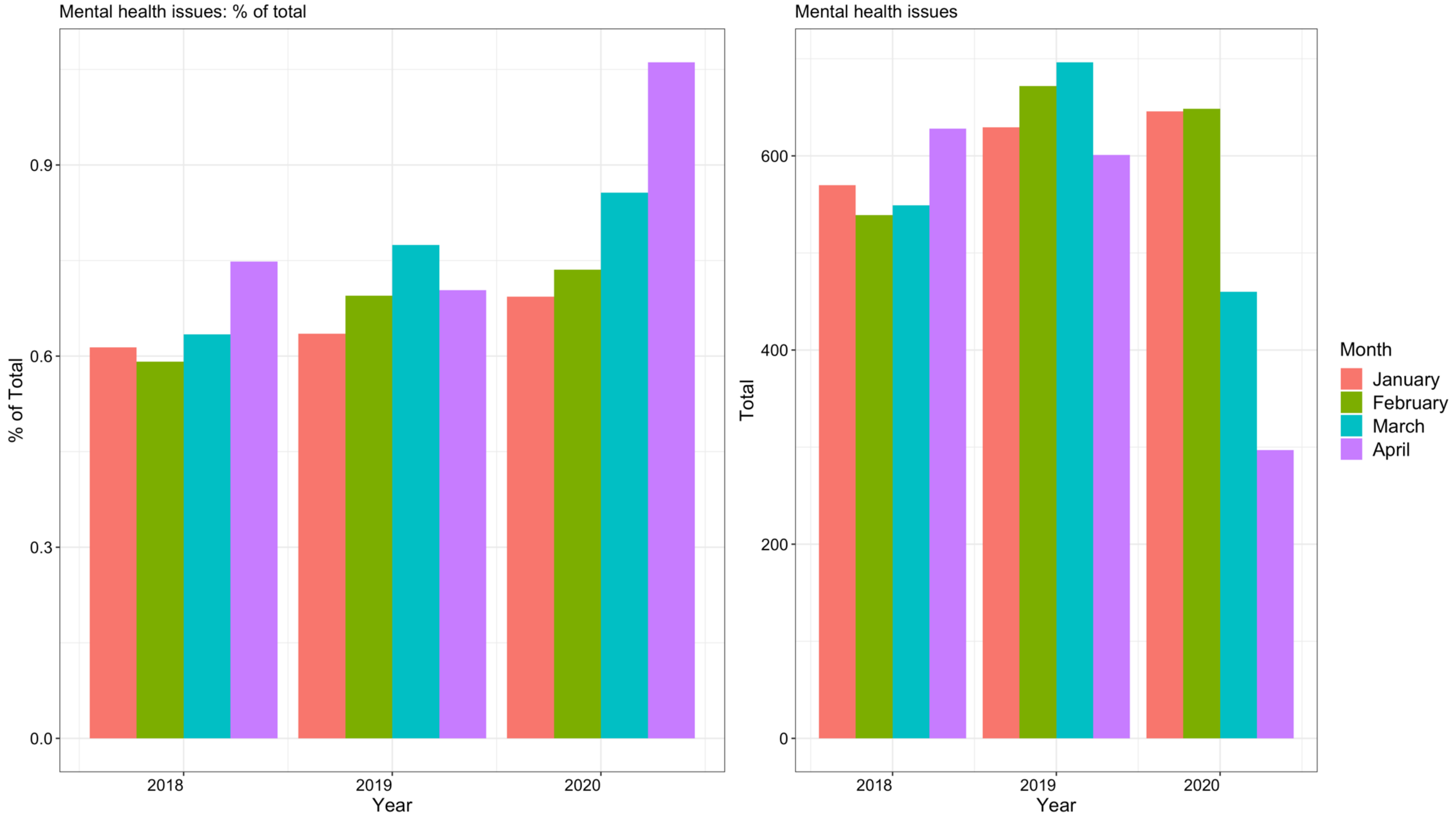

Diabetic keto-acidosis

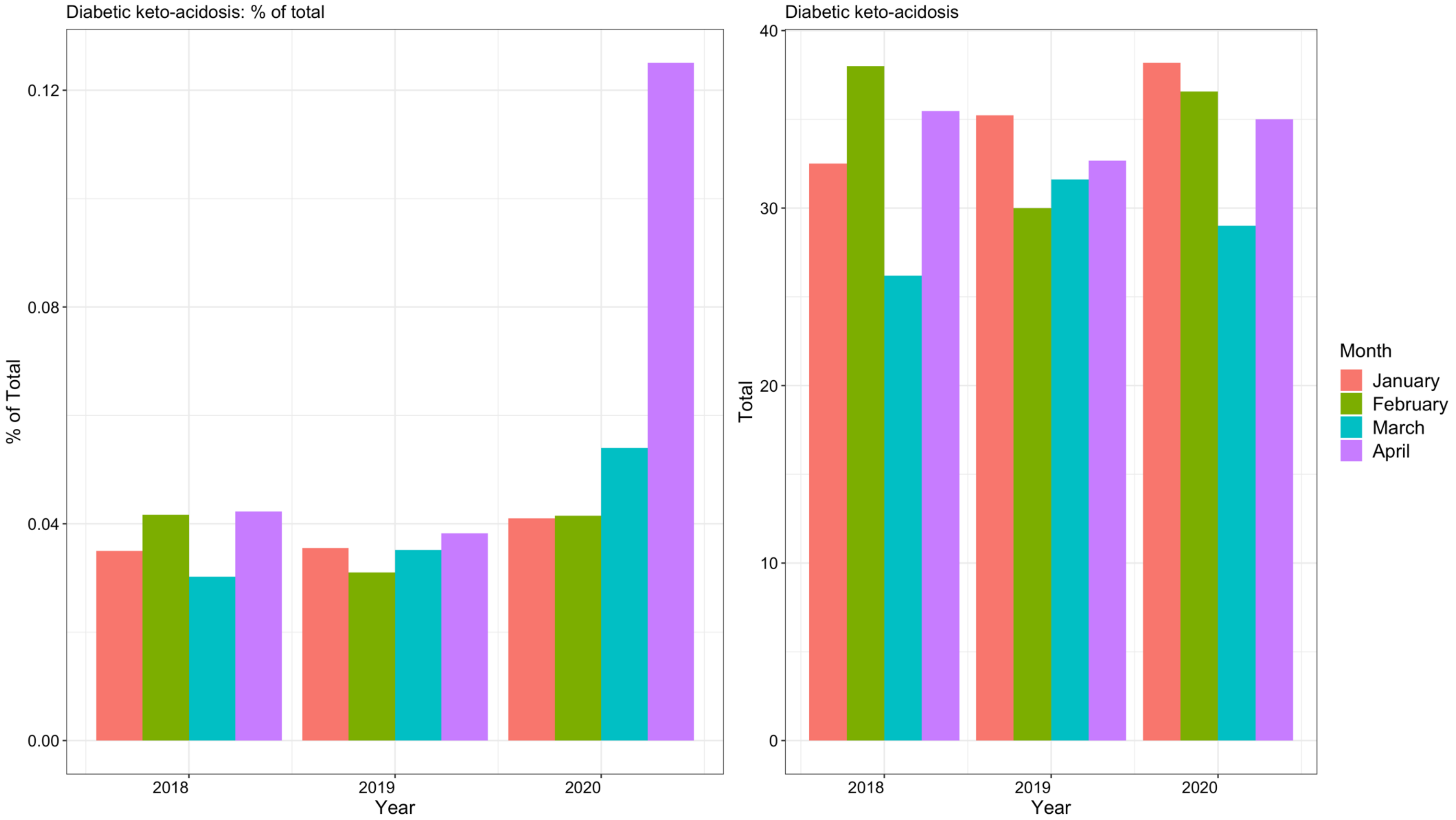

Minor head injury

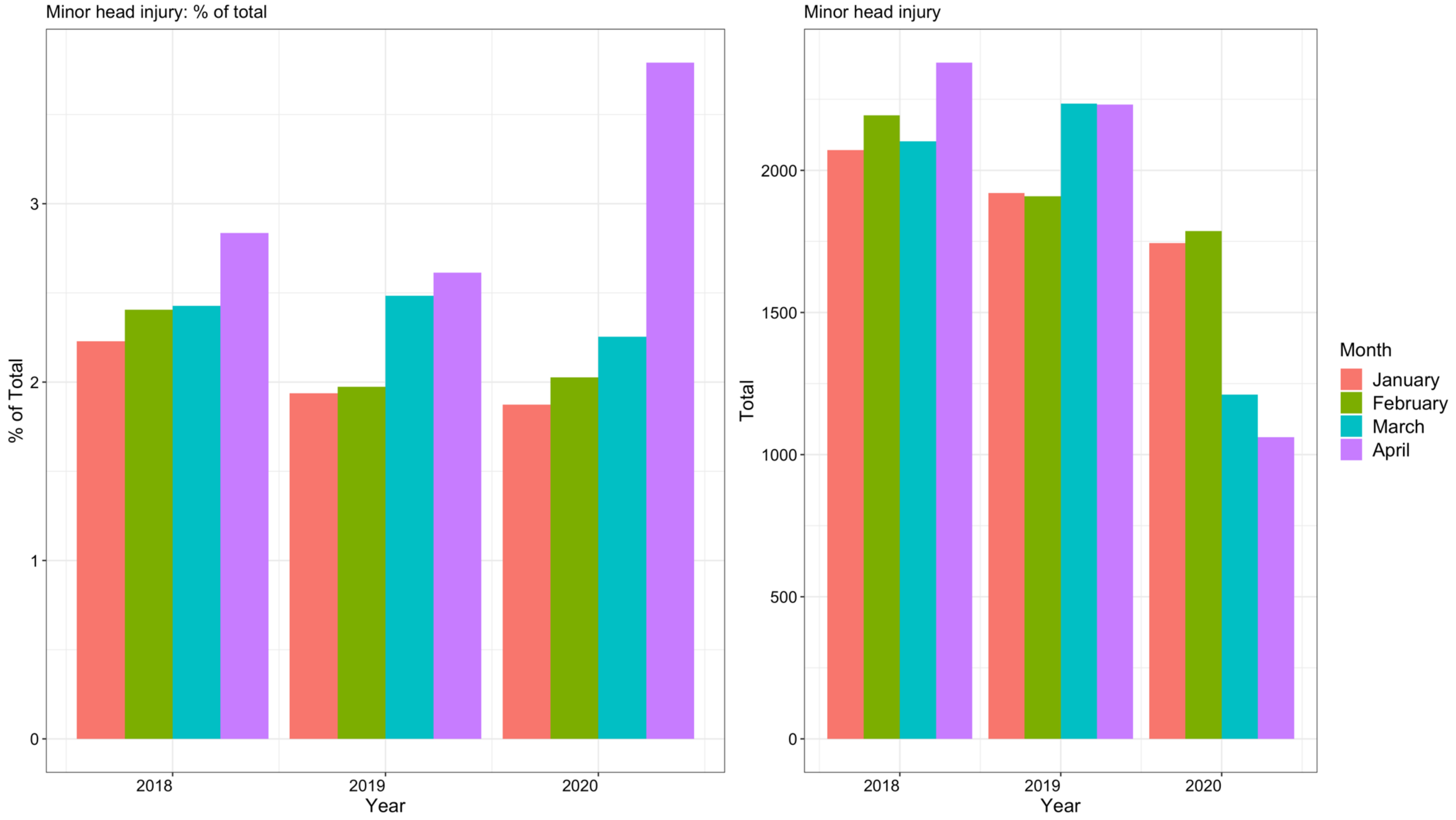

Supplement: S10 Fig — Percentages of total ED attendances (left) and absolute numbers (right) of children with diagnosis of (a) tonsillitis; (b) otitis media; (c) LRTIs; (d) GI infections; (e) appendicitis; (f) testicular torsion; (g) intussusception; (h) mental health issues; (i) diabetic ketoacidosis; (j) radius fracture; and (k) minor head injury; comparing the 28-day standardized numbers for the months of January–April for 2018 vs. 2019 vs. 2020, shown for countries with of a cumulative 14-day rate of new SARS-CoV-2 cases per 100,000 of 80 or more. ED, emergency department; GI, gastrointestinal; LRTI, lower respiratory tract infection; SARS-CoV-2, Severe Acute Respiratory Syndrome Coronavirus 2. (PDF) [file pmed.1003974.s022.pdf]
